# CLPTM1L induces estrogen receptor β signaling-mediated radioresistance in non-small cell lung cancer cells

**DOI:** 10.1186/s12964-020-00571-4

**Published:** 2020-09-17

**Authors:** Hang Li, Jun Che, Mian Jiang, Ming Cui, Guoxing Feng, Jiali Dong, Shuqin Zhang, Lu Lu, Weili Liu, Saijun Fan

**Affiliations:** 1 Tianjin Key Laboratory of Radiation Medicine and Molecular Nuclear Medicine, Institute of Radiation Medicine, Chinese Academy of Medical Sciences and Peking Union Medical College, 238 Bai-Di Road, Tianjin, 300192 P.R. China; 2 Department of Radiation Oncology, Affiliated Hospital of Jiangnan University, 200 Hui-He Road, Wuxi, 214062 Jiangsu P.R. China

**Keywords:** Radioresistance, Non-small cell lung cancer, CLPTM1L, ERβ, Radiotherapy

## Abstract

**Introduction:**

Radioresistance is a major challenge in lung cancer radiotherapy, and new radiosensitizers are urgently needed. Estrogen receptor β (ERβ) is involved in the progression of non-small cell lung cancer (NSCLC), however, the role of ERβ in the response to radiotherapy in lung cancer remains elusive. In the present study, we investigated the mechanism underlying ERβ-mediated transcriptional activation and radioresistance of NSCLC cells.

**Methods:**

Quantitative real-time PCR, western blot and immunohistochemistry were used to detect the expression of CLPTM1L, ERβ and other target genes. The mechanism of CLPTM1L in modulation of radiosensitivity was investigated by chromatin immunoprecipitation assay, luciferase reporter gene assay, immunofluorescence staining, confocal microscopy, coimmunoprecipitation and GST pull-down assays. The functional role of CLPTM1L was detected by function assays in vitro and in vivo.

**Results:**

CLPTM1L expression was negatively correlated with the radiosensitivity of NSCLC cell lines, and irradiation upregulated CLPTM1L in radioresistant (A549) but not in radiosensitive (H460) NSCLC cells. Meanwhile, IR induced the translocation of CLPTM1L from the cytoplasm into the nucleus in NSCLC cells. Moreover, CLPTM1L induced radioresistance in NSCLC cells. iTRAQ-based analysis and cDNA microarray identified irradiation-related genes commonly targeted by CLPTM1L and ERβ, and CLPTM1L upregulated ERβ-induced genes CDC25A, c-Jun, and BCL2. Mechanistically, CLPTM1L coactivated ERβ by directly interacting with ERβ through the LXXLL NR (nuclear receptor)-binding motif. Functionally, ERβ silencing was sufficient to block CLPTM1L-enhanced radioresistance of NSCLC cells in vitro. CLPTM1L shRNA treatment in combination with irradiation significantly inhibited cancer cell growth in NSCLC xenograft tumors in vivo.

**Conclusions:**

The present results indicate that CLPTM1L acts as a critical coactivator of ERβ to promote the transcription of its target genes and induce radioresistance of NSCLC cells, suggesting a new target for radiosensitization in NSCLC therapy.

Video Abstract

## Background

Non-small cell lung cancer (NSCLC) remains the leading cause of cancer-related death worldwide; more than 200,000 new cases are diagnosed annually, of which 16% of patients survive longer than 5 years [1, 2]. NSCLC is the most prevalent histological type of lung cancer, accounting for 85% of all cases; it comprises large cell carcinomas, squamous cell carcinomas, and adenocarcinomas [3, 4]. One reason for the low survival rate of this malignancy is the uncontrolled proliferation and metastatic potential of NSCLC cells [5, 6]. Radiotherapy (RT) is a powerful modality that is widely used in the clinical management of NSCLC [7, 8]. However, NSCLC cells exhibit intrinsic or acquired resistance to RT, which leads to treatment failure and induces local recurrence of NSCLC [9, 10]. Hence, the identification of new and more effective radiosensitizing approaches for the treatment of NSCLC is urgently needed to increase the survival rate of patients.

Cleft lip and palate transmembrane 1-like (CLPTM1L), also called cisplatin resistance-related gene 9 (CRR9), is a 62 kDa protein of 538 amino acids that was identified among the genes involved in resistance to the anticancer drug cisplatin in ovarian cancer cells [11]. It is located at the 5p15.33 locus near telomerase reverse transcriptase [12], which was identified as a susceptibility region for lung and several other cancers [13–19]. CLPTM1L is a predicted transmembrane protein that is expressed in normal and malignant tissues including the cervix, lung, breast, ovary, pancreatic and skin [12, 15, 20–22]. CLPTM1L is dysregulated in many human lung cancer tissues and cells [20, 23–28], particularly in NSCLC cells [20, 29–31]. Although CLPTM1L shows a strong genetic association with the development of NSCLC and is closely related to drug resistance in cancer cells, the role of CLPTM1L in the response to radiotherapy in NSCLC cells remains undetermined. Furthermore, the mechanism underlying the role of CLPTM1L in promoting NSCLC growth needs to be addressed.

Estrogen receptors (ERs) are ligand-dependent transcription factors that belong to the subfamily of steroid receptors [32]. The two isoforms, ERα and ERβ, are encoded by unique genes, although they share a common structural and functional organization [33]. As members of the nuclear receptor protein family, ERs are found mainly in the nucleus and can be activated by estrogen as well as other coactivators [34–36]. ERα and ERβ are detected in normal and tumor lung tissues [37–39], and affect survival in lung cancer patients through promoting the proliferation of cancer cells [40–42]. Recent evidence suggests that ERα and ERβ are expressed in NSCLC cell lines and tissues and play important roles during cancer development [37, 43–46]. In addition, ERβ is expressed at higher levels than ERα in NSCLC cells and plays a more important role [34, 47]. ERβ and its agonists are able to promote NSCLC progression through complicated molecular signaling networks [46, 48]. ERs mediate the radiosensitivity of triple-negative breast cancer cells [49]. However, whether ERs, especially ERβ, are involved in modulating the radiosensitivity of NSCLC cells remains poorly understood.

In the present study, we investigated the mechanism underlying ERβ-mediated transcriptional activation and radioresistance of NSCLC cells. The results show that CLPTM1L directly interacts with and acts as a coactivator of the transcription factor ERβ, leading to the activation of ERβ target genes in NSCLC cells and thereby promoting radioresistance. The present findings shed light on the mechanism underlying the function of ERβ in modulating the radiosensitivity of NSCLC cells, and suggest a new approach to NSCLC radiotherapy.

## Materials and methods

### Cell culture and treatment

Non-small cell lung cancer (NSCLC) cell lines H841, H23, H520, H460, H1299, A549 and H358 were cultured in RPMI 1640 (Gibco, USA). MDA-MB-231, and HEK293T cells stably transfected with pcDNA-ERβ were cultured in DMEM (Invitrogen, USA). All cell lines were supplemented with heat-inactivated 10% FBS (Gibco, USA), 100 units/ml penicillin, and 100 mg/ml streptomycin and grown at 5% CO_2_ and 37 °C. Cells were collected and seeded in 6-, 24-, or 96-well plates for 24 h and then transfected with corresponding plasmids or siRNAs using Lipofectamine 2000 (Invitrogen, USA) according to the manufacturer’s instructions.

### Irradiation studies

A Gammacell® 40 Exactor (Atomic Energy of Canada Limited, Chalk River, ON, Canada) was used for all experiments. For the relative experiments, the cells were exposed to ^137^Cs γ-ray irradiation (IR) at a dose rate of 1 Gy/min after 24 h of transfection.

### Total RNA isolation and real-time PCR

Total RNA was isolated from cells after 2 days of IR using TRIzol reagent (Invitrogen, USA) according to the instructions. First-strand cDNA was synthesized with the PrimeScript reverse transcriptase Kit (TaKaRa Bio, China). Real-time PCR was performed as described previously [50]. Primers used in the study were listed in Additional file 1: Table S1. All experiments were repeated 3 times.

### Plasmid construction and small interference RNA

The plasmids, such as pcDNA3.1, pCMV-Tag2B, pET28a, pGEX-4 T1, p*Silencer* 4.1-CMV neo vector, pGL3-Basic vector and pRL-TK plasmid (Promega, Madison, WI, USA) were kept in our laboratory. To construct a plasmid expressing CLPTM1L, the full-length cDNA of human CLPTM1L gene was cloned into pcDNA3.1 or pCMV-Tag2B to generate pcDNA-CLPTM1L or pCMV-CLPTM1L. The mutant sequence of CLPTM1L cDNA (with a mutated LXXLL motif) was cloned into pCMV-Tag2B or pcDNA3.1 to generate pCMV-CLPTM1L-mut or pcDNA-CLPTM1L-m. And the full-length cDNA of human ERβ gene was cloned into pcDNA3.1 to generate pcDNA-ERβ. The ERE luciferase reporter (ERE-LUC) was constructed by inserting estrogen response element (ERE) into the pGL3-Basic vector [51]. Mutant construct of ERE, carrying a substitution of 6 nucleotides within the core seed sequence of ERβ, was named ERE-LUC-mut. The resulting products were cloned into the multiple cloning sites of the vectors, such as pCMV-Tag2B, pET28a, pcDNA3.1 and pGEX-4 T1, respectively. The sequence of CLPTM1L shRNA (https://www.sigmaaldrich.com/china-mainland.html) was cloned into p*Silencer* vector to generate p*Silencer*-CLPTM1L (CLPTM1L shRNA). All the constructions were verified by sequence analysis. The primers for construction and siRNAs/shRNA used in this study were described in Additional file 1: Table S1.

### Western blot analysis

For western blot analysis, total protein lysate was extracted from tissues or cells with RIPA buffer (Solarbio) after 2 days of IR. For relative experiments, the nuclear or cytoplasmic protein was extracted from cells using Nuclear Protein Extraction Kit (Solarbio). The protein samples were subjected to SDS-PAGE and then transferred to a nitrocellulose membrane, blocked with 5% non-fat milk, and incubated with primary antibodies for 2 h at room temperature. Primary antibodies used were mouse anti-GAPDH (Proteintech, Cat NO.: HRP-60004), rabbit anti-CLPTM1L (ABclonal, Cat NO.: A10468), mouse anti-ERβ (Abcam, Cat NO.: ab288), rabbit anti-CDC25A (Proteintech, Cat NO.: 55031–1-AP), rabbit anti-c-Jun (Cell Signaling, Cat NO.: 60A8), mouse anti-BCL2 (Cell Signaling, Cat NO.: 15071), rabbit anti-Histone H3 (Proteintech, Cat NO.: 17168–1-AP) and mouse anti-Flag (ABclonal, Cat NO.: AE004). Then, treated with secondary antibody diluted in PBS at room temperature for 1 h. Membranes were washed in PBS-T and bound antibody was detected by enhanced chemiluminescence system Western Blotting Detection Reagents (Amersham Biosciences, Buckinghamshire, UK). All experiments were repeated 3 times.

### MTT assays

Cell growth assays were carried out using MTT (3-(4,5-dimethylthiazol-2-yl)-2,5-diph-enyltetrazolium bromide) reagent (Sigma, USA) as described previously [52]. In brief, transfected cells were trypsinized, counted, and plated into 96-well plates. After IR and incubating different time periods, MTT was added directly to each well, followed by incubation for 4 h, and then the supernatant was removed and 100 μl of dimethyl sulfoxide was added to stop the reaction. Absorbance at 490 nm was measured using an ELISA reader system (Labsystem, Multiskan Ascent). All experiments were performed in triplicate.

### EdU assays

Cell proliferation was determined after 2 days of IR by 5-ethynyl-2′-deoxyuridine (EdU) incorporation assay, which was carried out using the Cell-Light TM EdU imaging detection kit (RiboBio) according to the manufacturer’s instructions. All experiments were repeated 3 times.

### Colony formation assay

NSCLC cell lines to be tested were trypsinized and dissociated into single-cell suspensions for plating in 6-well plates (500–800 cells/well). IR (2, 4, 8 Gy) was delivered to the cells. Tissue culture medium was replenished every 3 days. After 12 days of IR, we rinsed the cells with cold phosphate-buffered saline (PBS), followed by fixation with methanol and staining with Giemsa (Sigma-Aldrich). Digital images of the plates were taken for permanent record of colony counting, using Image J software.

### Apoptosis assay

Cell apoptosis was determined by using an In Situ Cell Death Detection Kit based on labeling of DNA strand breaks (Roche, USA) according to manufacturer’s instructions. Analysis was performed after 3 days of IR by light microscopy and the result was quantified through Image J.

### The overlap of CLPTM1L-modulated genes, IR-related genes, and ERE containing genes

Compared with the control group, cells transfected with CLPTM1L were used to test the targets modulated by CLPTM1L. Then, 4161 CLPTM1L target genes were obtained from the analysis of iTRAQ-based study for CLPTM1L in A549 cells (Additional file 1: Table S2). Further screening for the genes was performed according to the *P*-value and fold changes in expression and 233 CLPTM1L-modulated genes [*P* < 0.05, log_10_(FC) < − 0.14 or log_10_(FC) > 0.14] (Additional file 1: Table S3) were obtained for the overlap. In addition, 936 irradiation-related genes [*P* < 0.05, log_10_(FC) < − 0.2 or log_10_(FC) > 0.2] (Additional file 1: Table S4) were obtained by gene expression (cDNA) microarray (The Beijing Genomics Institute, China). According to the GEO DataSets (https://www.ncbi.nlm.nih.gov/gds/?term=), 582 ERE-containing genes (ERβ-responsive genes, which contained EREs in the transcription regulatory region and could be regulated by 17β-estradiol in the ERβ wild-typed cells but couldn’t be regulated in the ERβ mutated cells) with a *P*-value cutoff were used for further overlapping (Additional file 1: Table S6). The datasets for combined analysis of CLPTM1L-modulated genes, IR-related genes, and ERE containing genes are provided in Additional file 1: Table S7.

### Chromatin immunoprecipitation assays

Chromatin immunoprecipitation (ChIP) assays were performed using the EpiQuik Chromatin Immunoprecipitation Kit from Epigentek Group Inc. A549 cells were lysed 48 h after transfection (24 h after 4 Gy IR). Protein/DNA complexes were immunoprecipitated by CLPTM1L or Flag antibodies, using normal rabbit IgG as a negative control. The primers used for PCR amplification were flanking the ERE in the promoters of CDC25A, BCL2, and c-Jun [53–55]. The antibodies used were rabbit anti-CLPTM1L (Abcam, Cat NO.: ab198862) and mouse anti-Flag (ABclonal, Cat NO.: AE004).

### Immunohistochemistry staining

NSCLC tissue microarrays were purchased from Xi’an Aomei Biotechnology. IHC staining was performed as described previously [56]. The negative control was performed as above protocol without using primary antibody. The staining level of CLPTM1L, CDC25A, BCL2, and c-Jun was classified into three groups using a modified scoring method based on the intensity of staining (0, negative; 1, low; 2, high) and the percentage of stained cells (0, 0% stained; 1, 1–49% stained; 2, 50–100% stained). A multiplied score (intensity scorepercentage score) lower than 1 was considered to be negative staining (−), 1 were considered to be moderate staining (+), and 2 was considered to be intense staining (++). Detailed information of the tissue microarray was shown in Additional file 1: Table S11. For animal assays, tumor tissue samples were harvested, fixed in 4% paraformaldehyde/PBS, dehydrated, embedded in paraffin blocks, and cut into 4-μm-thick sections. Deparaffinized tissue sections were rehydrated and stained using specific antibodies for CLPTM1L and Ki-67, before incubating and staining with biotinylated secondary antibodies. Signal intensity was determined with an avidin-biotin horseradish peroxidase complex and 3, 3′-diaminobenzidine (BD Biosciences, USA) as the chromogen. Representative photographs were taken using an Olympus CX31 microscope (Olympus America, Melville, NY, USA), after analyzing all slides. Primary antibodies used were rabbit anti-CLPTM1L (Abcam, Cat NO.: ab198862), rabbit anti-CDC25A (Proteintech, Cat NO.: 55031–1-AP), rabbit anti-c-Jun (Cell Signaling, Cat NO.: 60A8), mouse anti-BCL2 (Cell Signaling, Cat NO.: 15071) and rabbit anti-Ki67 (Proteintech, Cat NO.: 27309–1-AP).

### Luciferase reporter gene assay

Cells were plated into 24-well plates (3 × 10^4^ cells per well). After 24 h, the cells were co-transfected with the pRL-TK plasmids (50 ng per well) containing the Renilla luciferase gene (Promega, Madison, WI, USA) and ERE-LUC-wt or ERE-LUC-mut (100 ng per well). At 24 h post-transfection, the cells were exposed to 4 Gy IR. Then a standard dual luciferase reporter gene assay was performed after 24 h of IR, and the results were normalized using pRL-TK. All experiments were performed at least three times.

### Immunofluorescence staining and confocal microscopy

Immunofluorescence staining was performed as described previously [57]. After 48 h of transfection (24 h of 4 Gy IR), the cells were fixed with paraformaldehyde, and permeabilized with 0.1% Triton X-100 in PBS. After blocking in PBS containing 3%BSA, the cells were incubated with primary antibodies at room temperature. After washing with PBS, the cells were incubated with fluorophore-conjugated secondary antibody (R&D Systems, USA) and DAPI. After washing with PBS, slides were mounted with glycerol and observed under a confocal microscopy (Leica TCS SP5, Germany). Primary antibodies used were rabbit anti-CLPTM1L (Sigma, Cat NO.: HPA014791), mouse anti-ERβ (Abcam, Cat NO.: ab288) and mouse anti-Flag (ABclonal, Cat NO.: AE004).

### Coimmunoprecipitation (co-IP) assay and GST pull-down

The cells treated with transfection and 4 Gy IR were harvested and lysed in a lysis buffer (50 mmol/L Tris-HCl, pH 8.0, 100 mmol/L NaCl, 50 mmol/L sodium fluoride, 1% Nonidet P-40, 1 mmol/L dithiothreitol, 1 mmol/L Na_3_VO_4_, 1 mmol/L Microcystin-LR, 1 mmol/L phenylmethylsulfonyl fluoride, 10 mg/mL leupeptin, and 10 mg/mL aprotinin). The lysates were incubated with antibodies/protein G-conjugated agarose beads (Millipore, USA). The antibodies used were mouse anti-ERβ (Abcam, Cat NO.: ab288) and mouse anti-Flag (ABclonal, Cat NO.: AE004). The precipitates were washed six times with ice-cold lysis buffer, resuspended in PBS, followed by western blot analysis. For western blot analysis, the following antibodies were used: primary antibody is rabbit anti-ERβ (Proteintech, Cat NO.: 14007–1-AP) and rabbit anti-CLPTM1L (ABclonal, Cat NO.: A10468); secondary antibody is IPKine HRP mouse anti-rabbit IgG light chain (Abbkine, Cat NO.: A25022). The GST pull-down was performed according to published protocols [58]. Glutathione beads were recovered by a brief centrifugation and washed six times with lysis buffer, followed by western blot analysis.

### Animal models and treatment

Male athymic (nu/nu) BALB/c mice of 4–6 weeks of age with an average body weight of 18 g were obtained from Beijing HFK Bioscience Co., Ltd. (Beijing, China) and housed in a certified, specific pathogen-free level animal facility (with individually ventilated cages and independent ventilation system) at the Institute of Radiation Medicine (IRM), Chinese Academy of Medical Sciences (CAMS). All in vivo studies were approved by the Institutional Animal Care and Use Committee of IRM, CAMS under the Permit No. 1526, with an approval date of 16 September 2019. A total of 4 × 10^6^ radiosensitive H460 or radioresistant A549 cells after transfection were injected into the right anterior armpit of nude mice to establish lung cancer model according to previous research [59, 60]. IR treatment started when the tumors reached to an average volume of 200 mm^3^, as previously described [61]. All mice were randomized into four groups (5 mice per group, randomization was assigned by a computer-based, Excel-generated list of participating animals) as follows: (1) Control shRNA transfection; (2) CLPTM1L shRNA transfection; (3) IR + Control shRNA transfection; (4) IR + CLPTM1L shRNA transfection. IR was delivered using a lead shield so that one chest area was irradiated and the other chest area and the rest of the mouse were shielded. IR was delivered daily 2 Gy dose on days 10–14 after tumor inoculation (total of 5 days of treatment), as shown in relevant figure and legend. All experimental mice were euthanized by cervical dislocation under anesthesia at the completion of 30 days. Tumor samples were collected for pathological analysis. Tumor volume (V) was monitored by measuring the length (L) and width (W) using calipers and calculated according to the formula (L × W^2^) × 0.5.

### Statistical analysis

Each experiment was repeated at least three times. Statistical significance was assessed by comparing mean values (± SD) using a Student’s *t* test for independent groups and was assumed for *P* < 0.05 (*), *P* < 0.01 (**) and *P* < 0.001 (***).

## Results

### CLPTM1L induces the radioresistance of NSCLC cells

**Fig. 1:**
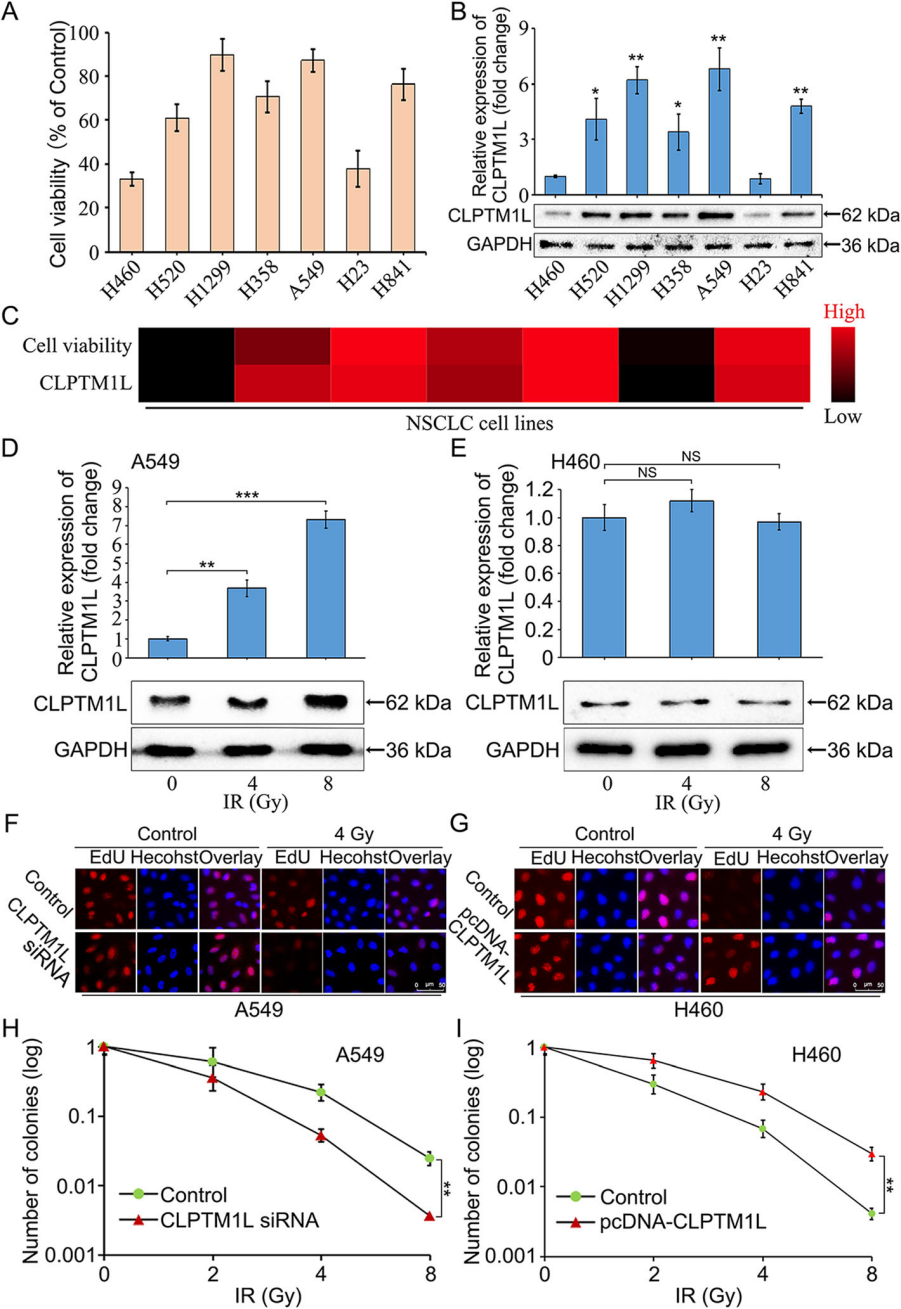
CLPTM1L induces the radioresistance of NSCLC cells. **a** The cell viabilities of different NSCLC cell lines exposed to 4 Gy of γ-ray irradiation (IR) were examined by colony formation assay after 12 days of IR and expressed as percent change compared with the control group (group without IR). **b** The expression levels of CLPTM1L in different NSCLC cell lines exposed to 4 Gy of IR were determined by western blot and real-time PCR analysis and expressed as fold change compared with the H460 group. **c** Heatmap showing the relationship between the expression level of CLPTM1L and the viability of NSCLC cells exposed to IR. **d**–**e** The expression levels of CLPTM1L in A549 and H460 cells exposed to various doses of γ-ray IR were detected by real-time PCR and western blot analysis. **f** The effect of CLPTM1L siRNA and/or 4 Gy of γ-ray IR on cell proliferation was measured by EdU assay in A549 cells. **g** The effect of CLPTM1L overexpression and/or 4 Gy of IR on cell proliferation was tested by EdU assay in H460 cells. **h**–**i** Cells (A549 and H460) pretreated with CLPTM1L siRNA or pcDNA-CLPTM1L (24 h) were exposed to IR (0, 2, 4, and 8 Gy). After 12 days, colonies were counted for quantification. Data are representative of three independent experiments; Student’s *t* test; * *P* < 0.05; ** *P* < 0.01; *** *P* < 0.001

CLPTM1L is dysregulated in many malignancies, especially in NSCLC, and promotes the development of NSCLC [20, 30, 62–65]. However, whether CLPTM1L is involved in the response to radiotherapy in NSCLC remains unexplored. To investigate the relationship between CLPTM1L and radiosensitivity in NSCLC cells, different NSCLC cells were exposed to 4 Gy of γ-ray irradiation (IR), then the viability of the cells and the expression levels of CLPTM1L in different cell lines were examined. The results showed that the expression of CLPTM1L was positively correlated with the viability of NSCLC cells exposed to IR (Fig. 1a–c). The differences in CLPTM1L expression among the cell lines with different radiosensitivities led us to hypothesize that CLPTM1L upregulation is positively associated with radioresistance. To test this hypothesis, we examined the relationship between IR and CLPTM1L levels. The results showed that γ-ray IR upregulated CLPTM1L in a dose- and time-dependent manner in radioresistant NSCLC cells (A549), whereas it had no significant effect in radiosensitive NSCLC cells (H460) (Fig. 1d–e and Additional file 1: Fig. S1A–B). These results identified CLPTM1L as a marker gene with a potential function in the regulation of NSCLC cell radiosensitivity. Additionally, exposure to IR resulted in CLPTM1L translocation from the cytoplasm into the nucleus in A549 and H460 cells (Additional file 1: Fig. S1C–F), suggesting that CLPTM1L was able to function in nucleus in NSCLC cells exposed to IR. The results of the EdU assay indicated that exposure to 4 Gy of γ-ray IR combined with CLPTM1L siRNA decreased A549 cell proliferation, whereas CLPTM1L overexpression promoted the growth of H460 cells exposed to IR (Fig. 1f–g). The results of the MTT assay demonstrated that CLPTM1L siRNA decreased the proliferation of IR-treated A549 cells in a time-dependent manner, whereas CLPTM1L siRNA alone or 4 Gy of γ-ray IR had no significant effect on cell growth (* *P* < 0.05, ** *P* < 0.01, Student’s *t* test, Additional file 1: Fig. S1G). However, overexpression of CLPTM1L abolished the effect of IR on suppressing H460 cell proliferation in a time-dependent manner (* *P* < 0.05, ** *P* < 0.01, Student’s *t* test, Additional file 1: Fig. S1H). CLPTM1L siRNA increased apoptosis in A549 cells exposed to IR, whereas CLPTM1L overexpression had the opposite effect in H460 cells (Additional file 1: Fig. S1I and J). The results of the clonogenic cell survival assay showed that CLPTM1L siRNA radiosensitized A549 cells (Fig. 1h), whereas CLPTM1L overexpression rendered H460 cells radioresistant (Fig. 1i). The interference efficiency of CLPTM1L siRNA and transfection efficiency of pcDNA-CLPTM1L were validated by western blot analysis in the cells (Additional file 1: Fig. S1K and L). These results strongly suggest that CLPTM1L is negatively correlated with the radiosensitivity of NSCLC cells and can induce radioresistance of the cells.

### Identification of candidate targets of CLPTM1L by iTRAQ-based analysis

**Fig. 2:**
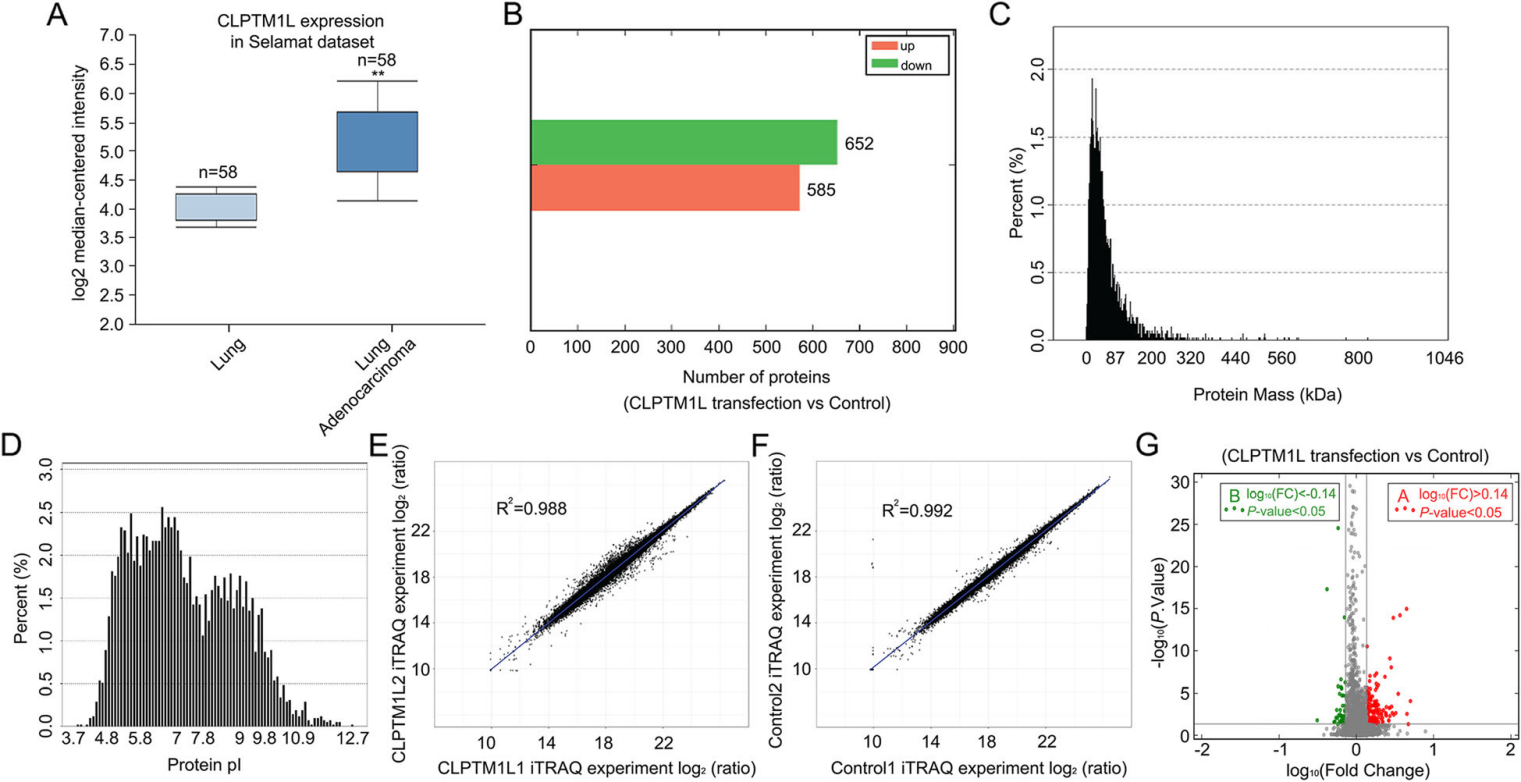
Identification of candidate targets of CLPTM1L by iTRAQ-based analysis. **a** Expression levels of CLPTM1L in lung adenocarcinoma compared with normal lung tissues obtained from public databases (Selamat dataset). Student’s *t* test; ** *P* < 0.01. **b** Proteins upregulated or downregulated by CLPTM1L in A549 cells exposed to IR were identified by iTRAQ-based analysis based on the *P*-value (*P* < 0.05). **c**–**d** Distribution of mass or isoelectric point of the 1237 proteins identified. **e**–**f** Analysis of parallelism between CLPTM1L overexpressing groups or control groups from iTRAQ-based analysis. **g** Volcano plot showing 233 CLPTM1L-modulated genes among the 1237 genes identified based on fold changes in expression [log_10_(FC) < − 0.14 or log_10_(FC) > 0.14]

Next, we examined the role of CLPTM1L in clinical samples. The expression level of CLPTM1L in lung cancer compared with other cancer types was shown in Additional file 1: Fig. S2A (Barretina dataset obtained from oncomine, https://www.oncomine.org/). Then, Selamat and TCGA datasets confirmed that CLPTM1L was expressed at higher levels in lung adenocarcinoma than in normal lung tissues and was positively expressed in various NSCLC tissues (Fig. 2a and Additional file 1: Fig. S2B). Therefore, iTRAQ-based analysis was performed to identify potential target genes modulated by CLPTM1L in NSCLC cells exposed to IR. Among 4161 CLPTM1L target genes identified in the iTRAQ-based study in A549 cells, 1237 genes were selected based on the *P*-value (*P* < 0.05) (Additional file 1: Table S2). Of these, 652 genes were upregulated and 585 genes were downregulated in CLPTM1L-overexpressing A549 cells (Fig. 2b). The distribution of mass and isoelectric point of the proteins is shown in Fig. 2c and d, and analysis of the parallelism between the groups confirmed the accuracy of above results (Fig. 2e–f). In addition, 233 CLPTM1L-modulated genes were further identified among the 1237 genes based on fold changes in expression [log_10_(FC) < − 0.14 or log_10_(FC) > 0.14] as indicated by the volcano plot in Fig. 2g and Additional file 1: Table S3. Taken together, the results indicate that CLPTM1L is positively correlated with the development of NSCLC, and 233 potential targets of CLPTM1L are identified by the iTRAQ-based analysis.

### CLPTM1L expression is positively associated with that of the ERβ-induced genes CDC25A, c-Jun, and BCL2 in NSCLC

**Fig. 3:**
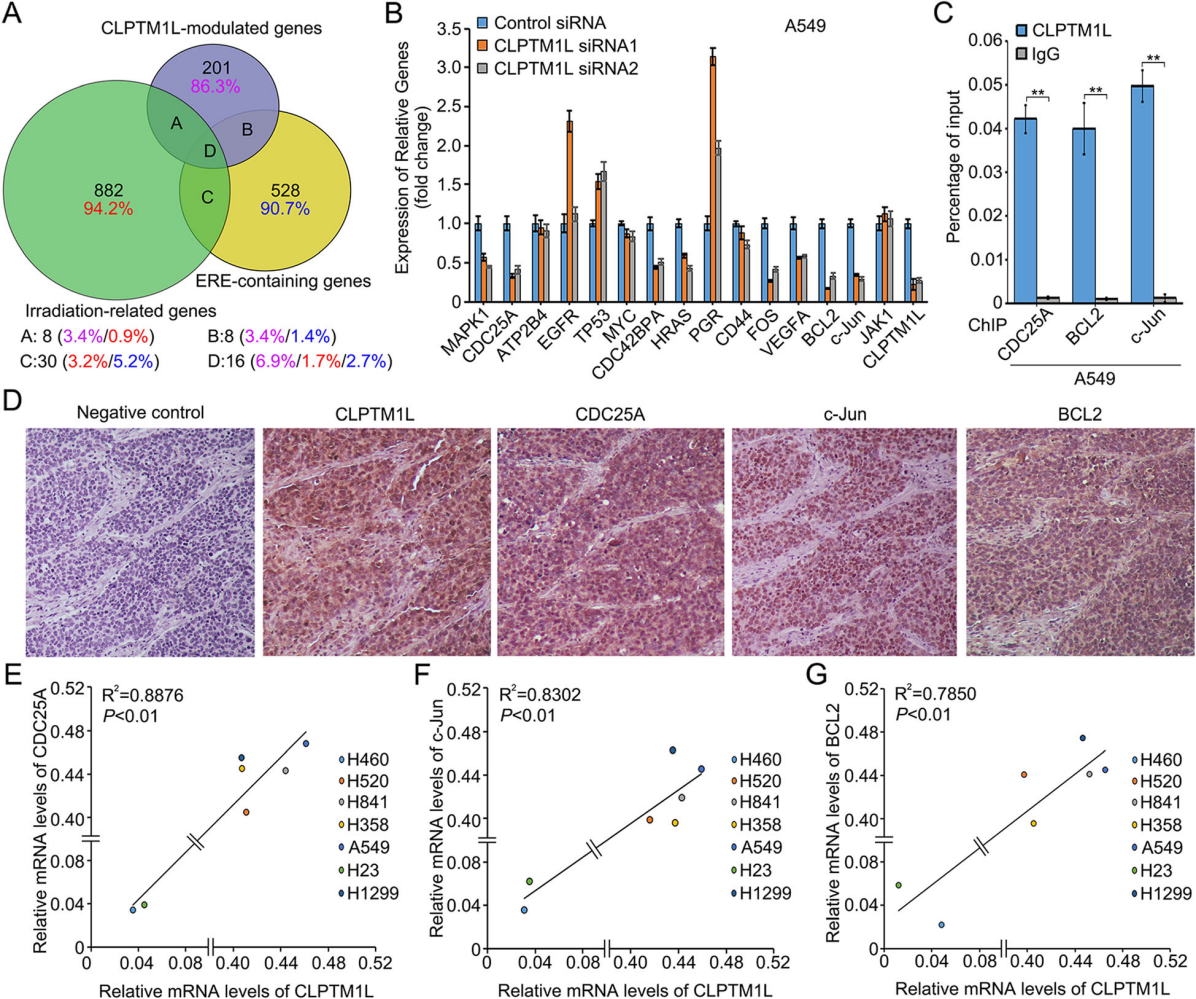
CLPTM1L expression is positively associated with the ERβ-induced genes in NSCLC. **a** Venn diagram showing the overlapping target genes of CLPTM1L, ERβ, and IR. **b** Real-time PCR analysis of the expression of 16 candidate target genes in A549 cells treated with CLPTM1L siRNAs/control siRNA and IR. **c** CLPTM1L binding to the promoters of CDC25A, c-Jun, and BCL2 was examined by ChIP-qPCR assay in A549 cells exposed to IR. **d** The expression levels of CLPTM1L, CDC25A, c-Jun, and BCL2 were detected by IHC staining in 110 cases of NSCLC tissue samples using tissue microarray. Representative images were taken from the same sample in 110 cases of the tissue microarray (see the No.55 case in Additional file 1: Table S11). **e**–**g** Real-time PCR analysis of the correlation between CLPTM1L and CDC25A (or c-Jun and BCL2) mRNA levels in seven NSCLC cell lines exposed to IR (CDC25A, *P* < 0.01, R^2^ = 0.8876; c-Jun, *P* < 0.01, R^2^ = 0.8302; BCL2, *P* < 0.01, R^2^ = 0.7850; Pearson’s correlation). All experiments were repeated at least three times. Statistically significant differences are indicated. Student’s *t* test; ** *P* < 0.01

Gene expression (cDNA) microarray analysis was performed to identify target genes associated with the radiosensitivity of A549 cells, and 936 IR-related genes were detected in the cells [*P* < 0.05, log_10_(FC) < − 0.2 or log_10_(FC) > 0.2] (Additional file 1: Table S4). The LXXLL motif (L, leucine; X, any amino acid) of CLPTM1L is an important domain that interacts with the ligand-binding domain of nuclear receptors [66, 67]. In addition, mass spectrographic analysis of CLPTM1L in A549 cells also indicated that CLPTM1L interacted with several nuclear receptors including RXRα, RXRβ, PPARα, and especially ERβ, the latter of which showed the highest score among the candidate nuclear receptors (Additional file 1: Table S5). This suggested that CLPTM1L acted as a transcriptional coregulator to activate ERβ target genes in NSCLC cells. Then, 582 estrogen response element (ERE)-containing genes (ERβ-responsive genes) (Additional file 1: Table S6) were obtained from the GEO dataset (https://www.ncbi.nlm.nih.gov/gds/?term=) and used for further analysis. Taken together with the data of CLPTM1L-modulated genes, IR-related genes, and ERβ-responsive genes, the analysis identified 16 potential target genes associated with CLPTM1L-induced radioresistance (Fig. 3a and Additional file 1: Table S7). The regulation of the 16 candidate genes by CLPTM1L was confirmed by real-time PCR in A549 cells, and three target genes (CDC25A, BCL2, and c-Jun) could be significantly downregulated by CLPTM1L siRNAs in the cells (Fig. 3b). Meanwhile, these genes were proved to be directly regulated by ERβ in A549 and H460 cells (Additional file 1: Fig. S3A–B). Hence, CDC25A, BCL2, and c-Jun were selected to investigate the effect of CLPTM1L on ERβ-induced gene transcription associated with radioresistance of NSCLC cells. ChIP assays confirmed the interaction of CLPTM1L with the promoters of the three genes in A549 cells (Fig. 3c). IHC staining showed that the level of CLPTM1L was positively associated with that of CDC25A (or BCL2 and c-Jun) in 110 cases of NSCLC tissue samples (pairing χ^2^ analysis; Fig. 3d and Additional file 1: Tables S8–S11). Real-time PCR revealed that CLPTM1L mRNA expression was positively related to CDC25A, BCL2, and c-Jun mRNA expression in different NSCLC cell lines (*P* < 0.01; Pearson’s correlation; Fig. 3e–g). Additionally, target proteins with high levels of expression were associated with poor overall survival according to the Kaplan-Meier plotter database and previous report [68, 69] (Additional file 1: Fig. S3C–D). Meanwhile, our previous study and public datasets (Bhattacharjee dataset obtained from oncomine, https://www.oncomine.org/) also confirmed the expression levels of the three targets in different lung cancer tissues [69] (Additional file 1: Fig. S3E–F). These data suggest that CLPTM1L expression is positively associated with that of the ERβ-induced genes CDC25A, c-Jun, and BCL2 in NSCLC.

### Irradiation increases the levels of CDC25A, c-Jun, and BCL2 by CLPTM1L in radioresistant NSCLC cells and CLPTM1L upregulates three target genes through ERβ

**Fig. 4:**
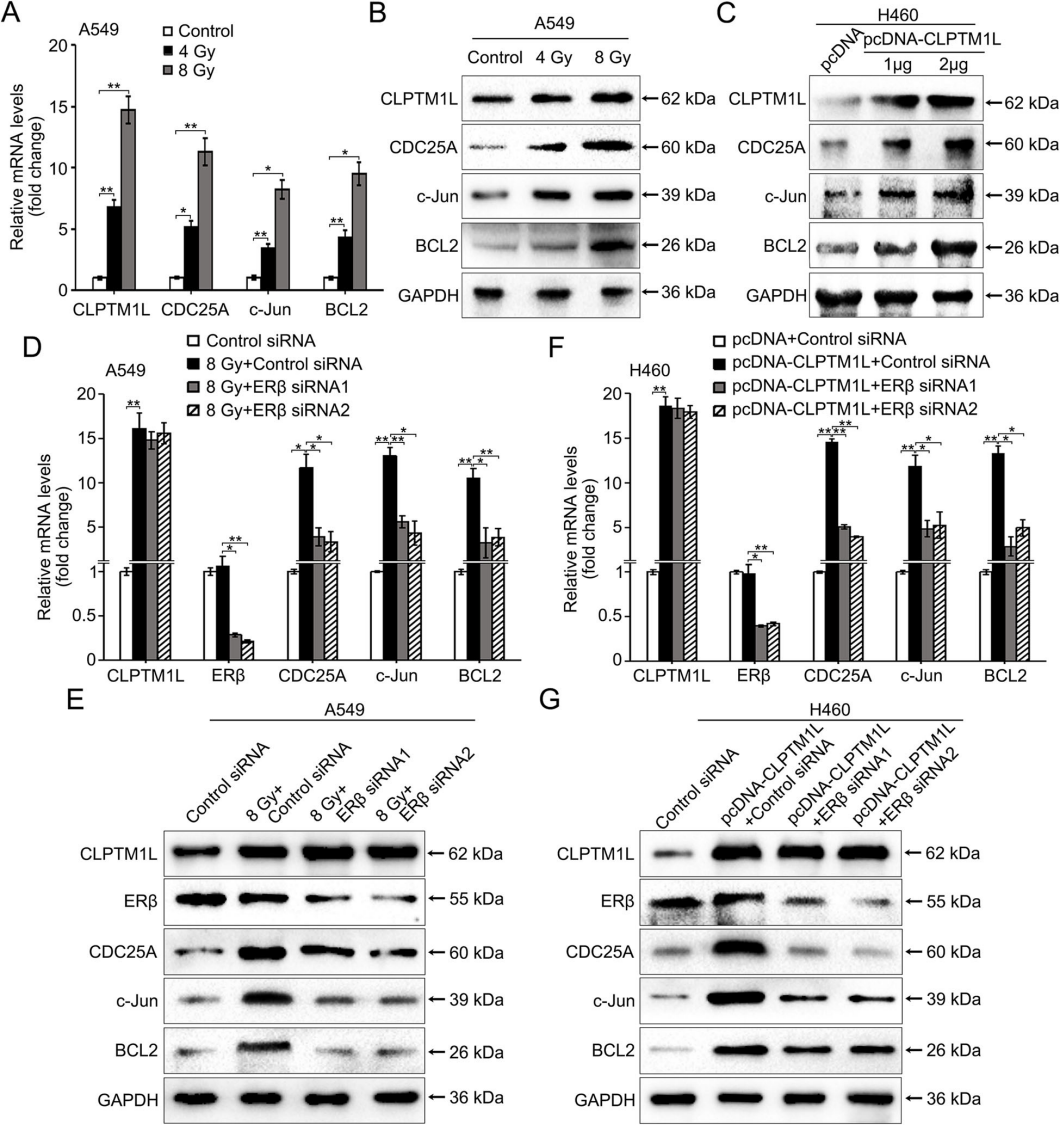
CLPTM1L upregulates the levels of CDC25A, c-Jun, and BCL2 through ERβ in NSCLC cells. **a** Real-time PCR analysis of the mRNA levels of CLPTM1L, CDC25A, c-Jun, and BCL2 in A549 cells exposed to different doses of γ-ray IR. **b** Western blot analysis of the protein levels of these genes in A549 cells. **c** Western blot analysis of the protein levels of these genes in H460 cells exposed to 4 Gy IR. **d**–**e** Real-time PCR and western blot analysis of the mRNA and protein levels of CLPTM1L, ERβ, CDC25A, c-Jun, and BCL2 in A549 cells treated with IR and siRNAs. **f**–**g** Real-time PCR and western blot analysis of the expression of these genes in H460 cells treated with the indicated plasmids/siRNAs and 4 Gy IR. All experiments were repeated at least three times. Statistically significant differences are indicated. Student’s *t* test; * *P* < 0.05; ** *P* < 0.01

IR increased CLPTM1L levels in radioresistant A549 cells, and CLPTM1L expression was positively associated with the levels of ERβ-induced genes in NSCLC cells. We therefore examined the effect of IR on the levels of the three target genes in A549 cells. IR upregulated CLPTM1L concomitant with the upregulation of CDC25A, c-Jun, and BCL2 in a dose-dependent manner in A549 cells, as determined by real-time PCR (Fig. 4a) and western blot analysis (Fig. 4b). Meanwhile, IR upregulated CLPTM1L protein expression in a time-dependent manner, leading to the upregulation of the three target genes in A549 cells (Additional file 1: Fig. S4A). Moreover, knockdown of CLPTM1L abolished the upregulation of target genes mediated by IR in A549 cells (Additional file 1: Fig. S4B), and IR had no effect on the three target genes in H460 cells (Additional file 1: Fig. S4C), suggesting that IR increased the CLPTM1L-induced upregulation of CDC25A, c-Jun, and BCL2 in radioresistant NSCLC cells. Next, we found that CLPTM1L overexpression upregulated CDC25A, c-Jun, and BCL2 at the mRNA and protein levels in radiosensitive H460 cells (Fig. 4c and Additional file 1: Fig. S4D). SiRNA-mediated silencing of ERβ abolished the IR-mediated upregulation of the three ERβ target genes at the mRNA and protein levels, whereas it had no effect on the modulation of CLPTM1L mediated by IR in A549 cells (Fig. 4d–e). SiRNA-mediated silencing of ERβ could also abolish the upregulation of the three target genes mediated by CLPTM1L in H460 cells (Fig. 4f–g), supporting that CLPTM1L upregulated the three target genes through ERβ. In addition, siRNA for CLPTM1L and ERβ overexpression could both regulate the levels of the three target genes in A549 cells (Additional file 1: Fig. S4E–H), further supported that the regulation of the target genes by CLPTM1L was dependent on ERβ and vice versa. Taken together, these results indicate that IR increases the levels of CDC25A, c-Jun, and BCL2 by CLPTM1L in radioresistant NSCLC cells, and CLPTM1L upregulates the three target genes through ERβ.

### CLPTM1L activates the promoter of ERβ-induced genes by stimulating the transcription factor ERβ in NSCLC cells

**Fig. 5:**
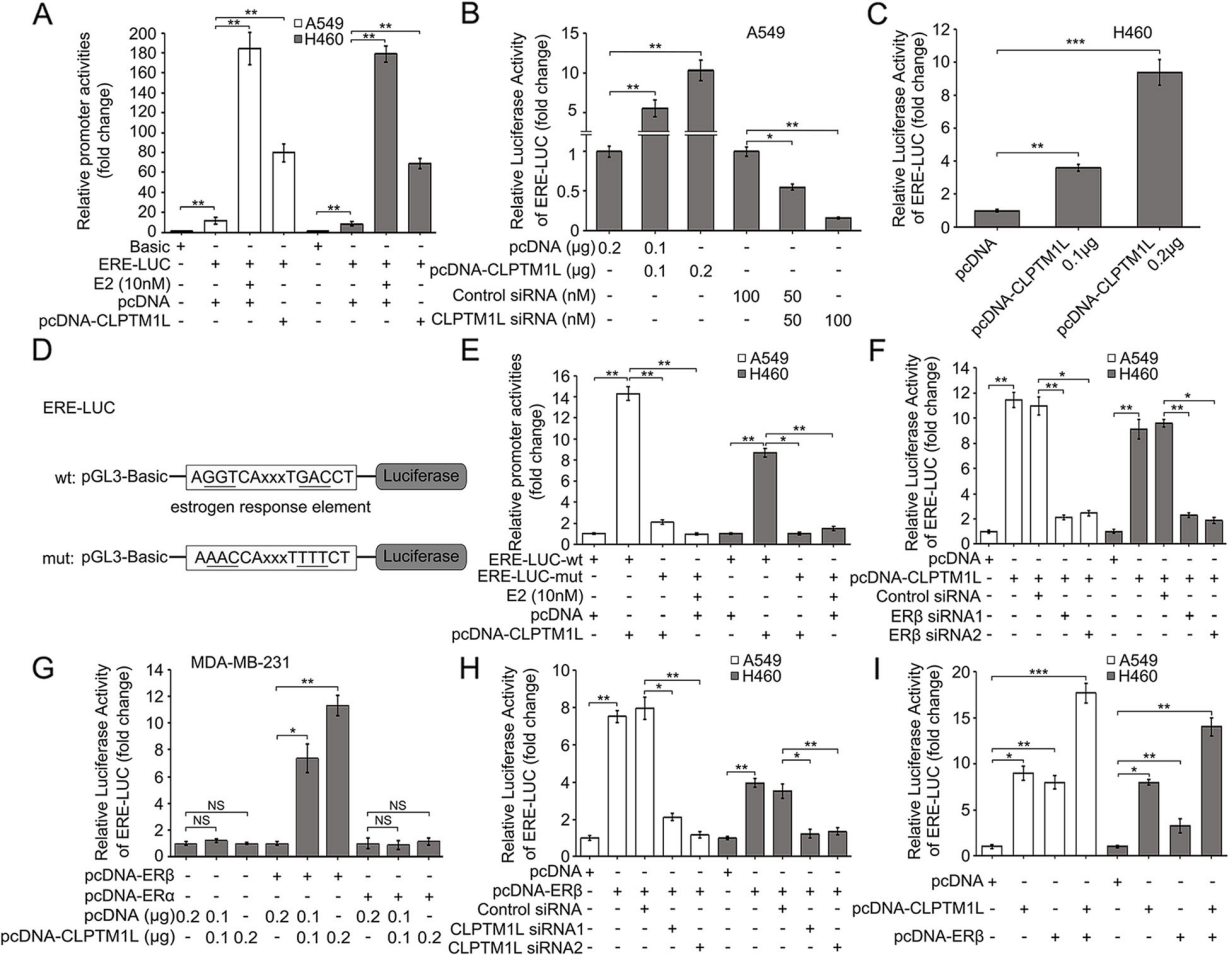
CLPTM1L activates the promoter of ERβ-induced genes by stimulating ERβ in NSCLC cells. **a–i** Cells were transfected with relative plasmids and siRNAs, exposed to 4 Gy IR, followed by luciferase reporter gene assays. **a** ERE-LUC activity was examined using luciferase reporter gene assays in A549 and H460 cells transfected with pcDNA-CLPTM1L plasmids (E2, also called 17β-estradiol, was used as the positive control). **b** ERE-LUC activity was examined using luciferase reporter gene assays in A549 cells transfected with pcDNA-CLPTM1L plasmids or CLPTM1L siRNA. **c** ERE-LUC activity was determined using luciferase reporter gene assays in H460 cells treated with various doses of pcDNA-CLPTM1L plasmids. **d** The mutation of the ERE is shown in the ERE-LUC construct. **e** Luciferase reporter gene assays in A549 and H460 cells transfected with ERE-LUC constructs with wild-type or mutant ERβ-binding sites (E2 was used as the positive control). **f** ERE-LUC activity was detected by luciferase reporter gene assays in A549 and H460 cells transfected with pcDNA-CLPTM1L and ERβ siRNAs. **g** The effect of CLPTM1L on ERE-LUC activity was tested by luciferase reporter gene assays in ER-negative cells (MDA-MB-231) transfected with or without pcDNA-ERβ/pcDNA-ERα. NS, not significant. **h** ERE-LUC activity was detected by luciferase reporter gene assays in A549 and H460 cells transfected with pcDNA-ERβ and CLPTM1L siRNAs. **i** ERE-LUC activity was examined by luciferase reporter gene assays in A549 and H460 cells transfected with pcDNA-CLPTM1L and/or pcDNA-ERβ plasmids. All experiments were repeated at least three times. Student’s *t* test; * *P* < 0.05; ** *P* < 0.01; *** *P* < 0.001

According to previous study, the ERβ-induced genes possess functional EREs (estrogen response elements) within the transcription regulatory region [51]. We therefore constructed an ERE luciferase reporter (ERE-LUC) to test the effect of CLPTM1L on ERβ-induced transcription. CLPTM1L promoted ERE-LUC activity in A549 and H460 cells (Fig. 5a), and the result was validated in HEK293T cells (Additional file 1: Fig. S5A). Overexpression of CLPTM1L increased ERE-LUC activity in a dose-dependent manner in NSCLC and HEK293T cells, whereas silencing of CLPTM1L decreased ERE-LUC activity in A549 cells (Fig. 5b–c and Additional file 1: Fig. S5B), suggesting that CLPTM1L activated EREs in the promoters of ERβ target genes in NSCLC cells. We next cloned an ERE-LUC-mut construct carrying a substitution of six nucleotides within the core seed sequence of ERβ (Fig. 5d), and observed that CLPTM1L failed to promote the activity of ERE-LUC-mut in NSCLC and HEK293T cells (Fig. 5e and Additional file 1: Fig. S5C). Moreover, ERβ knockdown markedly abolished the CLPTM1L-mediated increase in ERE-LUC activity (Fig. 5f and Additional file 1: Fig. S5D). CLPTM1L had no effect on ERE-LUC activity in ERβ-negative cells (MDA-MB-231), whereas it increased the activity in cells overexpressing ERβ (Fig. 5g). Interestingly, the regulation of ERE-LUC activity mediated by CLPTM1L was abolished in ERα-overexpressed cells (Fig. 5g), suggesting that only ERβ was required for the CLPTM1L-mediated activation of EREs in the promoters of target genes. Additionally, siRNA-mediated silencing of CLPTM1L attenuated ERE-LUC activation induced by ERβ (Fig. 5h and Additional file 1: Fig. S5E). Overexpression of CLPTM1L and/or ERβ increased ERE-LUC activity in NSCLC and HEK293T cells (Fig. 5i and Additional file 1: Fig. S5F). The interference efficiency of relative siRNAs and transfection efficiency of over-expression vectors were validated by qPCR analysis in the cells (Additional file 1: Fig. S5G–I). These results indicate that CLPTM1L activates the EREs in the promoters of ERβ-induced genes by stimulating the transcription factor ERβ in NSCLC cells.

### CLPTM1L directly interacts with and coactivates ERβ

**Fig. 6:**
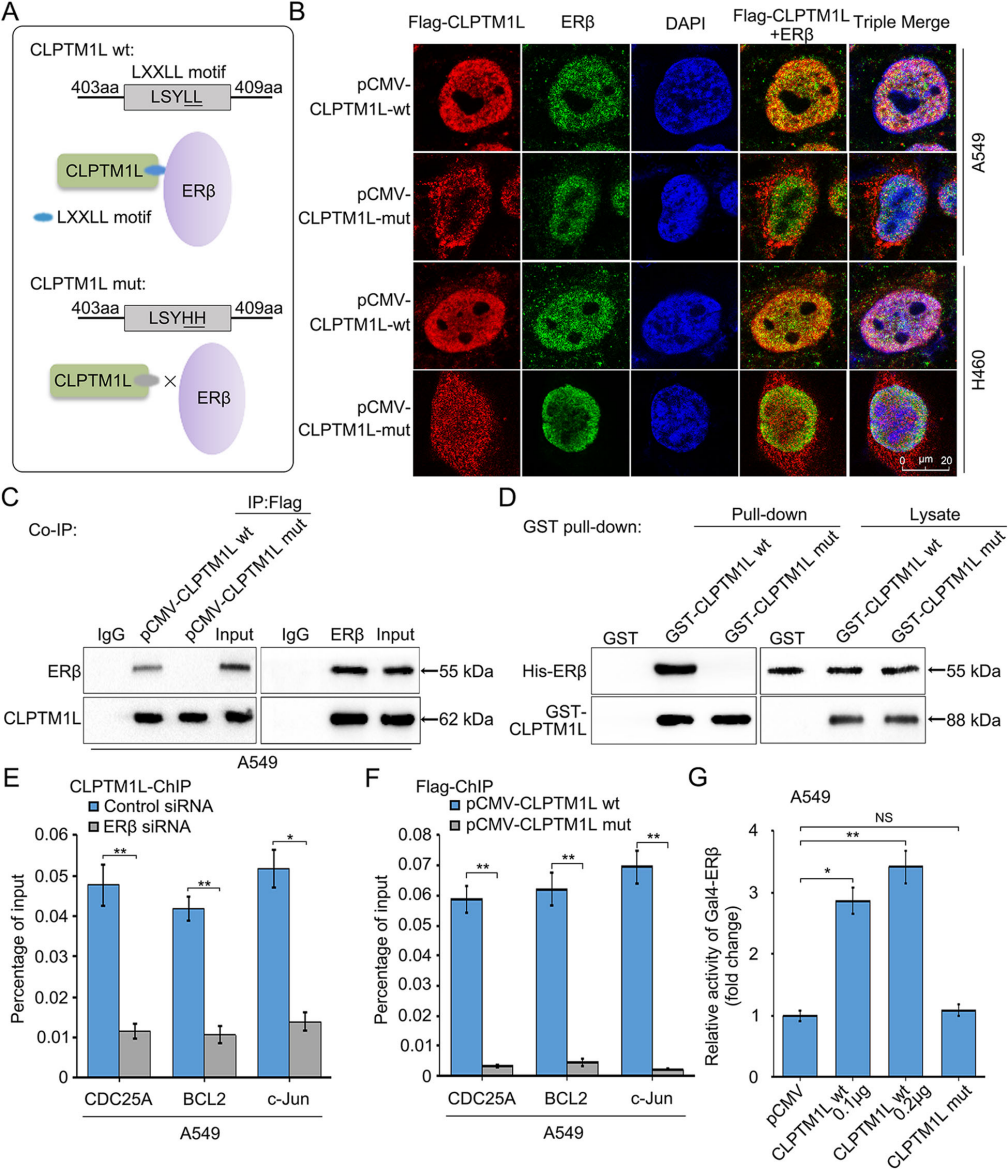
CLPTM1L directly interacts with and coactivates ERβ. **a** Model showing the effect of mutation of the LXXLL motif on abolishing the interaction between CLPTM1L and ERβ. **b** Confocal microscopy images of the localization of Flag-CLPTM1L (wt or mut) and ERβ in A549 and H460 cells exposed to 4 Gy IR. **c** The interaction of CLPTM1L (wt or mut) with ERβ was detected by Co-IP assays in A549 cells exposed to 4 Gy IR. **d** The direct interaction of recombinant GST-CLPTM1L (wt or mut) with His-ERβ was detected by GST pull-down assays and western blot analysis. **e**–**f** CLPTM1L binding to the promoters of CDC25A, c-Jun, and BCL2 was examined by ChIP-qPCR in A549 cells exposed to 4 Gy IR. **g** Luciferase activities of Gal4-ERβ were measured by luciferase reporter gene assays in A549 cells exposed to 4 Gy IR. NS, not significant. Each experiment was repeated at least three times. Student’s *t* test; * *P* < 0.05; ** *P* < 0.01

Next, we sought to investigate the mechanism by which CLPTM1L activates ERβ. Because CLPTM1L contains a LXXLL motif, a crucial domain for binding to nuclear receptors [70], we hypothesized that CLPTM1L interacts with ERβ through this motif. We therefore generated pCMV-CLPTM1L-mut (CLPTM1L containing a mutated LXXLL motif) (Fig. 6a). Confocal images showed that CLPTM1L interacted with ERβ, and mutation of the LXXLL motif abolished the interaction in A549 and H460 cells (Fig. 6b). According to the result, the nuclear localization of CLPTM1L in the cells was also abolished by the mutation, indicating that the LXXLL motif could be responsible for the translocation of CLPTM1L from the cytoplasm into the nucleus in NSCLC cells exposed to IR. Then the interaction between CLPTM1L with ERβ was confirmed using Co-IP assays and GST pull-down assays in vivo and in vitro (Fig. 6c–d), which showed that CLPTM1L bound directly to ERβ via the LXXLL motif. ChIP assays showed that both silencing of ERβ by siRNA and mutation of the LXXLL motif abolished binding of CLPTM1L to the promoters of the three ERβ target genes in A549 cells (Fig. 6e–f), indicating that CLPTM1L interacted with the target gene promoters through ERβ via the LXXLL motif. Finally, we constructed a plasmid encoding the ERβ protein fused to the Gal4 DNA-binding domain to test the interaction between CLPTM1L and ERβ. CLPTM1L significantly increased the luciferase activity of Gal4-ERβ in a dose-dependent manner, whereas the mutant CLPTM1L failed to activate it in A549 cells (Fig. 6g). This indicates that CLPTM1L directly interacts with ERβ via the LXXLL motif to coactivate ERβ in NSCLC cells.

### CLPTM1L induces the radioresistance of NSCLC cells by coactivating ERβ

**Fig. 7:**
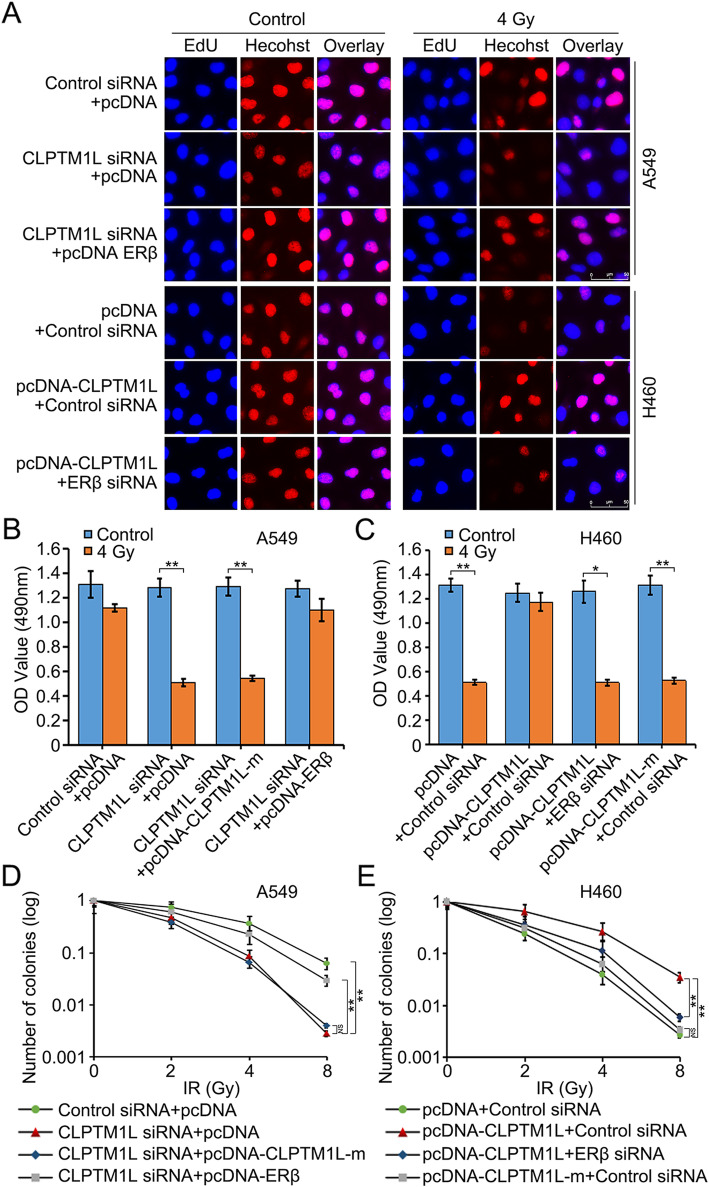
CLPTM1L induces radioresistance of NSCLC cells by coactivating ERβ. **a** Effect of CLPTM1L and ERβ on A549 and H460 cell proliferation after exposure to 0 or 4 Gy of IR as determined by the EdU assay. **b**–**c** MTT assays of the effect of ERβ/pcDNA-CLPTM1L-m (CLPTM1L containing a mutated LXXLL motif) on CLPTM1L-modulated radiosensitivity in A549 and H460 cells were performed after 3 days of IR. **d**–**e** A549/H460 cells pretreated with CLPTM1L siRNA/pcDNA-CLPTM1L and pcDNA-ERβ/ERβ siRNA/pcDNA-CLPTM1L-m were exposed to IR (0, 2, 4, and 8 Gy) and analyzed by clonogenic cell survival assay after 12 days of IR. The data presented are from three independent experiments; Student’s *t* test; * *P* < 0.05; ** *P* < 0.01; NS, not significant

Because ERβ contributed to the upregulation of target genes mediated by CLPTM1L and was coactivated by CLPTM1L in NSCLC cells, we tested the effects of ERβ silencing or overexpression on CLPTM1L-modulated cell radiosensitivity. The results of the EdU assay showed that treatment with 4 Gy of γ-ray IR combined with CLPTM1L siRNA decreased the proliferation of radioresistant A549 cells, and this effect was rescued by overexpression of ERβ (Fig. 7a). SiRNA-mediated silencing of ERβ attenuated CLPTM1L-induced radioresistance in radiosensitive H460 cells (Fig. 7A). This result was confirmed by MTT and apoptosis assays (Fig. 7b–c and Additional file 1: Fig. S6A–B). The clonogenic cell survival assay showed that ERβ overexpression blocked the radiosensitization caused by CLPTM1L interference in A549 cells (Fig. 7d), whereas ERβ silencing abolished the radioresistance induced by CLPTM1L in H460 cells (Fig. 7e). Moreover, the mutated CLPTM1L failed to modulate the radiosensitivity of NSCLC cells (Fig. 7b–e and Additional file 1: Fig. S6A–B). This suggested that CLPTM1L modulated the radiosensitivity of NSCLC cells through ERβ. Additionally, we confirmed that siRNA-mediated knockdown of CLPTM1L could be a new approach for sensitizing NSCLC cells to IR in vitro (Additional file 1: Fig. S6C–E). In conclusion, CLPTM1L induces radioresistance of NSCLC cells by coactivating ERβ.

### Silencing of CLPTM1L sensitizes xenograft NSCLC tumors to IR in an animal model

**Fig. 8:**
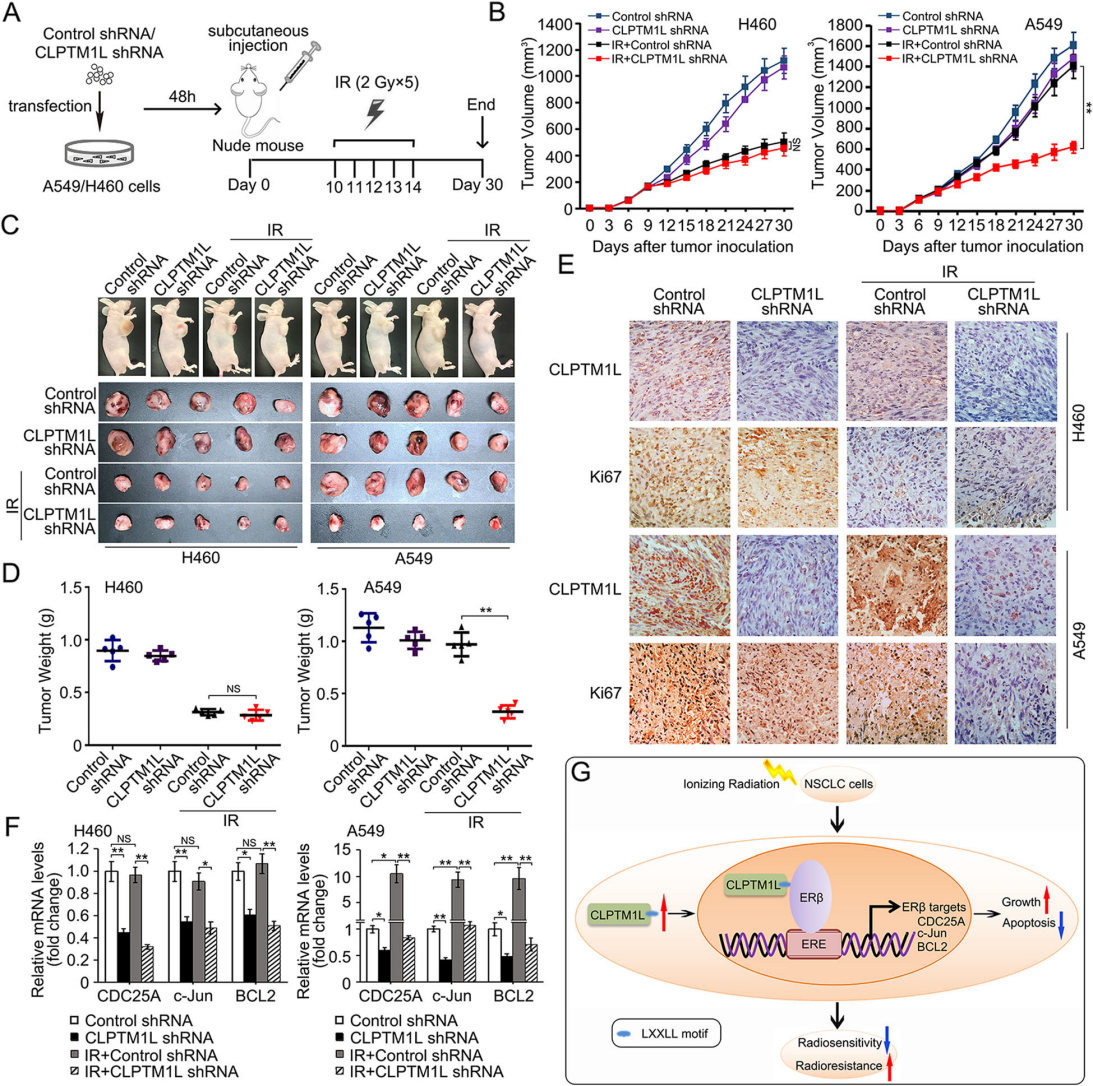
Silencing of CLPTM1L sensitizes xenograft NSCLC tumors to IR in an animal model. **a** Mice (*n* = 40) bearing NSCLC xenograft tumors developed from H460 or A549 cells transfected with control or CLPTM1L shRNA were treated with or without local IR, as illustrated in the diagram. **b** Growth of tumors generated by transplantation of NSCLC cells. **c** Images of dissected tumors from nude mice (*n* = 5/group). **d** Weight of resected xenograft tumors in nude mice. **e** Representative images (400× magnification) of H460 and A549 xenografts stained for CLPTM1L and Ki-67. **f** Real-time PCR analysis of the mRNA levels of CDC25A, c-Jun, and BCL2 in H460 and A549 xenografts. Statistically significant differences are indicated. NS, not significant. Student’s *t* test; * *P* < 0.05; ** *P* < 0.01. **g** The model shows that IR can increase the expression of CLPTM1L, and CLPTM1L upregulates the expression of ERβ-induced genes by coactivating ERβ via the LXXLL motif, leading to the radioresistance of NSCLC cells

To determine whether knockdown of CLPTM1L increases the radiosensitivity of NSCLC tumors in vivo, we generated a xenograft tumor model using male athymic (nu/nu) BALB/c mice (Fig. 8a). Local IR alone inhibited the growth of xenograft tumors from H460 cells, whereas IR alone had no significant inhibitory effect on the growth of A549 xenograft tumors (Fig. 8b–d). The combination of CLPTM1L shRNA and local IR had a greater effect on reducing A549 xenograft tumor size than treatment with local IR alone (Fig. 8b–d). These findings suggested that silencing of CLPTM1L could overcome acquired radioresistance in NSCLC tumors in vivo. Immunohistochemical staining validated the shRNA-mediated CLPTM1L knockdown in tumors and showed that IR increased the levels of CLPTM1L in A549 tumors, whereas it had no effect on H460 tumors (Fig. 8e). Assessment of the expression of the cell proliferation marker Ki-67 in tumors showed that combination of CLPTM1L shRNA and local IR markedly inhibited the expression levels of Ki-67 in A549 tumors (Fig. 8e). Finally, real-time PCR showed that local IR alone significantly upregulated ERβ-induced genes in A549 tumors compared with H460 tumors, whereas shRNA against CLPTM1L abolished the upregulation of these genes in A549 tumors (Fig. 8f). The interference efficiency of CLPTM1L shRNA in tumor cells was detected by western blot analysis in NSCLC tumors (Additional file 1: Fig. S7A). Collectively, the present results support that silencing of CLPTM1L sensitizes xenograft NSCLC tumors to IR in an animal model, suggesting a new strategy for increasing the radiosensitivity of NSCLC cells.

## Discussion

The acquisition of resistance to radiotherapy, which greatly increases patient morbidity and mortality, is a significant problem in the treatment of NSCLC [71, 72]. Therefore, the design of effective treatments capable of sensitizing radioresistant NSCLC to radiotherapy is an active research area. ERβ, an important member of the nuclear receptor protein family as well as a crucial transcription factor, is linked to the survival of breast cancer patients [73–75]. Recently, ERβ was shown to affect the progression of NSCLC by promoting the proliferation, invasion, and metastasis of NSCLC cells [38, 40, 48]. Many studies have focused on the pathological role of ERβ, which can be activated by estrogen or other ligands and coactivators, in the development of lung cancer, especially NSCLC [76–79]. However, whether ERβ can also function in modulating the radiosensitivity of NSCLC cells remains undetermined. The results of the present study suggest that ERβ signaling is involved in the radioresistance induced by CLPTM1L in NSCLC cells.

CLPTM1L is dysregulated in different NSCLC cell lines and closely related to the development of NSCLC [20, 30, 65]. In the present study, we showed that CLPTM1L was negatively correlated with NSCLC cell radiosensitivity, and γ-IR upregulated CLPTM1L in radioresistant NSCLC cells. The radioresistance of cancer cells is caused by a large number of signaling pathways, of which, the regulation of irradiation-related genes by IR plays an important role [80–82]. The expression levels of multiple irradiation-related genes are able to be regulated by IR in radioresistant cells, but not in radiosensitive cells [83, 84]. CLPTM1L acts as a marker gene, which can be upregulated by IR in radioresistant NSCLC cells, implies a potential function in the regulation of NSCLC cell radiosensitivity.

Next, our results suggested that CLPTM1L induced radioresistance in NSCLC, and silencing of CLPTM1L increased the therapeutic efficacy of IR in radioresistant NSCLC cells. iTRAQ-based analysis identified potential targets of CLPTM1L in NSCLC cells, and cDNA microarray analysis identified 936 IR-related genes in NSCLC cells. In addition, the LXXLL motif of CLPTM1L and mass spectrographic analysis of CLPTM1L in A549 cells suggested that the nuclear receptor ERβ interacted with CLPTM1L in the cells. ERα and ERβ affect the survival of lung cancer patients by promoting the proliferation of cancer cells [85, 86], and ERβ is expressed at higher levels than ERα in most NSCLC cell lines and tissues during cancer development [76, 87]. Meanwhile, ERβ showed the highest score among the candidate nuclear receptors interacting with CLPTM1L according to the mass spectrographic analysis. Hence, we focused on ERβ in the present study. Since ERβ target genes possess functional EREs within the transcription regulatory region [51], we identified 582 ERE-containing genes that could be regulated by ERβ as potential ERβ target genes. The results of combined analysis indicated that 16 IR-related genes could be regulated commonly by CLPTM1L and ERβ. Of the 16 genes, we identified CDC25A, c-Jun, and BCL2 as CLPTM1L-modulated ERβ-induced genes in NSCLC cells. We showed that the expression levels of CLPTM1L were significantly positively correlated with those of the three identified ERβ target genes in clinical NSCLC tissues and multiple NSCLC cell lines. These results suggest that CLPTM1L functions in modulating the transcription of ERβ target genes in association with the regulation of NSCLC cell radiosensitivity.

Next, we explored the mechanism by which CLPTM1L regulates ERβ target genes in NSCLC cells. We showed that IR increased the levels of ERβ target genes via CLPTM1L in radioresistant NSCLC cells, and CLPTM1L upregulated the target genes by stimulating the transcription factor ERβ and increasing the activities of target gene EREs in NSCLC cells. The LXXLL motif present in most coactivators is essential for binding to nuclear receptors and stimulating transcription factors [66, 88]. We therefore hypothesized that the fragment at aa 404–408 as the conserved LXXLL sequence of CLPTM1L was responsible for the interaction with ERβ. We showed that CLPTM1L directly interacted with ERβ via the LXXLL motif, and CLPTM1L interacted with the EREs in the promoters of ERβ-induced genes through ERβ. In addition, we found that ERα was useless for the regulation of EREs mediated by CLPTM1L, supporting that only ERβ was responsible for the CLPTM1L-mediated activation of EREs in the promoters of target genes. Taken together, the results indicate that CLPTM1L directly interacts with ERβ via the LXXLL motif to coactivate ERβ in NSCLC cells.

Increasing evidence supports the crucial roles of CDC25A, BCL2, and c-Jun in promoting the proliferation, apoptosis, and radioresistance of lung cancer cells [89–93]. Here, we provided further evidence that CLPTM1L induced radioresistance of NSCLC cells by coactivating ERβ and enhancing the expression of ERβ target genes. In vivo studies using radiosensitive H460 and radioresistant A549 xenografts demonstrated that silencing of CLPTM1L combined with local IR effectively overcame acquired radioresistance in animal models. The upregulation and nuclear translocation of CLPTM1L mediated by IR led to the radioresistance of NSCLC cells. Although siRNAs for CLPTM1L had a basal effect on growth of NSCLC cells in the absence of IR, the inhibition of CLPTM1L was still thought to be a considerable approach for sensitizing NSCLC cells exposed to IR. In addition, since little expression of CLPTM1L was observed in radiosensitive NSCLC cells, the nuclear translocation of CLPTM1L mediated by IR was not sufficient to induce the radioresistance of the cells. Therapeutically, our results provide a new target for improving the effect of NSCLC radiotherapy.

It has been reported that CLPTM1L functions mostly in cell cytoplasm during promoting the growth of cancer cells [63]. Here, we found that IR was able to induce the translocation of CLPTM1L from the cytoplasm into the nucleus in NSCLC cells. Moreover, the LXXLL motif and mass spectrographic analysis of CLPTM1L revealed a potential ability of CLPTM1L to interact with many other nuclear receptors, such as RXRα, RXRβ and PPARα. It has been reported that the nuclear receptors are involved in modulating the radiosensitivity of cancer cells during radiotherapy [94–96]. Above all, our results reveal a new character of CLPTM1L, which functions in the nucleus through interacting with nuclear receptors during NSCLC radiotherapy. Interestingly, there was a positive correlation between the expression levels of CLPTM1L and ERβ-induced genes in NSCLC tissues without IR (Fig. 3D), and siRNAs for CLPTM1L also had a weak effect on growth of NSCLC cells in the absence of IR, implying another mechanism of oncoprotein CLPTM1L in cytoplasm during the modulation of NSCLC development. This issue needs to be clarified in future research.

Many coactivators and steroids, especially estrogen, function in the activation of ERβ, which is a ligand-dependent transcription factor [33, 97]. In the present study, we showed that CLPTM1L induced ERβ target genes by acting as a coactivator of ERβ. We therefore investigated whether the activation of ERβ by CLPTM1L depended on the presence of estrogen in the cells. We found that the addition of 17β-estradiol had no effect on the interaction between CLPTM1L and ERβ or that of CLPTM1L with the EREs of ERβ target genes (Additional file 1: Fig. S7B–C). Moreover, overexpression of CLPTM1L and/or addition of 17β-estradiol increased ERE-LUC activity in NSCLC cells and promoted the proliferation of cells exposed to IR (Additional file 1 Fig. S7D–E). Hence, the coactivation of ERβ by CLPTM1L was independent of estrogen.

Although ERβ plays an important role in CLPTM1L-mediated radioresistance, knockdown of ERβ is not a feasible radiosensitizing method at the moment, because multiple functions of ERβ were still elusive in lung cancer. The present results and those of previous studies suggest that CLPTM1L expression is higher in radioresistant NSCLC cells than in radiosensitive cells, and it is considerably higher than that in normal cells [30, 98]. IR upregulated CLPTM1L only in radioresistant cells, whereas it had no effect in most radiosensitive cells. Thus, high expression of CLPTM1L in cells and IR-induced CLPTM1L upregulation are crucial mechanisms underlying CLPTM1L-induced radioresistance of NSCLC cells. This suggests that blocking CLPTM1L can markedly affect radioresistant NSCLC cells, whereas it has a weak effect on other cancer cells or normal cells because of the low levels of CLPTM1L in these cells. This result supports that inhibitors of CLPTM1L could be potent candidate radiosensitizers without significant toxicities toward normal tissues and cells.

## Conclusions

The results of the present study suggest a model, in which IR upregulates CLPTM1L and induces the translocation of CLPTM1L into the nucleus, which in turn upregulates the expression of ERβ-induced genes by coactivating ERβ through the LXXLL motif, leading to the radioresistance of NSCLC cells (Fig. 8g). CLPTM1L is therefore a promising target for overcoming radioresistance and restoring radiosensitivity in radioresistant NSCLC. An increasing number of studies are searching for effective approaches to sensitize radioresistant NSCLC to radiotherapy, and the present findings suggest a new target as well as a new strategy for increasing the radiosensitivity of NSCLC.

## Supplementary information

**Additional file 1: Fig. S1.** CLPTM1L induces the radioresistance of NSCLC cells. **Fig. S2.** Positive correlation of CLPTM1L with the development of NSCLC. **Fig. S3.** High-expressed CLPTM1L target genes are associated with poor patient overall survival. **Fig. S4.** CLPTM1L upregulates the levels of CDC25A, c-Jun, and BCL2 through ERβ in NSCLC cells. **Fig. S5.** CLPTM1L activates the promoter of ERβ-induced genes by stimulating ERβ in HEK293T cells. **Fig. S6.** CLPTM1L induces radioresistance of NSCLC cells by coactivating ERβ. **Fig. S7.** CLPTM1L coactivates ERβ independent of estrogen. **Table S1.** Primers and siRNAs using for relative experiments. **Table S2.** CLPTM1L target genes. CLPTM1L target genes after screening. **Table S4.** Irradiation-related genes. **Table S5.** Mass spectrographic analysis of CLPTM1L. **Table S6.** ERE-containing genes (ERβ-responsive genes). **Table S7.** Combined analysis of CLPTM1L-modulated genes, IR-related genes, and ERE containing genes. **Table S8.** Cross tabulation analysis of CLPTM1L and CDC25A in NSCLC tissues. **Table S9.** Cross tabulation analysis of CLPTM1L and c-Jun in NSCLC tissues. **Table S10.** Cross tabulation analysis of CLPTM1L and BCL2 in NSCLC tissues. **Table S11.** Non-small cell lung carcinoma & Normal TMA.

## Data Availability

All data generated or analysed during this study are included in this published article (and its supplementary information files).

## Abbreviations

NSCLC: Non-small cell lung cancer
DMEM: Dulbecco’s modified Eagle’s media
FBS: Fetal bovine serum
IHC: Immunohistochemistry
RT: Radiotherapy
IR: Irradiation
CLPTM1L: Cleft lip and palate transmembrane 1-like
ERβ: Estrogen Receptor β
CDC25A: Cell division cycle 25A
c-Jun: AP-1 transcription factor subunit
BCL2: B cell leukemia/lymphoma 2
E2: 17β-estradiol
GAPDH: Glyceraldehyde-3-phosphate dehydrogenase

## References

[1] Nagaraj AS, Lahtela J, Hemmes A (2017). Cell of origin links Histotype Spectrum to immune microenvironment diversity in non-small-cell lung Cancer driven by mutant Kras and loss of Lkb1. Cell Rep.

[2] McCann AP, Smyth P, Cogo F (2018). USP17 is required for trafficking and oncogenic signaling of mutant EGFR in NSCLC cells. Cell Commun Signal..

[3] Cooper WA, Lam DC, O'Toole SA (2013). Molecular biology of lung cancer. J Thorac Dis.

[4] Althammer S, Tan TH, Spitzmuller A (2019). Automated image analysis of NSCLC biopsies to predict response to anti-PD-L1 therapy. J Immunother Cancer.

[5] Ciaramella V, Sasso FC, Di Liello R (2019). Activity and molecular targets of pioglitazone via blockade of proliferation, invasiveness and bioenergetics in human NSCLC. J Exp Clin Cancer Res.

[6] Szymura SJ, Zaemes JP, Allison DF (2019). NF-kappaB upregulates glutamine-fructose-6-phosphate transaminase 2 to promote migration in non-small cell lung cancer. Cell Commun Signal.

[7] Gong HY, Wang Y, Han G (2019). Radiotherapy for oligometastatic tumor improved the prognosis of patients with non-small cell lung cancer (NSCLC). Thorac Cancer.

[8] Dunne V, Ghita M, Small DM (2017). Inhibition of ataxia telangiectasia related-3 (ATR) improves therapeutic index in preclinical models of non-small cell lung cancer (NSCLC) radiotherapy. Radiother Oncol.

[9] Klein C, Dokic I, Mairani A (2017). Overcoming hypoxia-induced tumor radioresistance in non-small cell lung cancer by targeting DNA-dependent protein kinase in combination with carbon ion irradiation. Radiat Oncol.

[10] Son B, Jun SY, Seo H (2016). Inhibitory effect of traditional oriental medicine-derived monoamine oxidase B inhibitor on radioresistance of non-small cell lung cancer. Sci Rep.

[11] Yamamoto K, Okamoto A, Isonishi S (2001). A novel gene, CRR9, which was up-regulated in CDDP-resistant ovarian tumor cell line, was associated with apoptosis. Biochem Biophys Res Commun.

[12] Wang S, Wu J, Hu L (2012). Common genetic variants in TERT contribute to risk of cervical cancer in a Chinese population. Mol Carcinog.

[13] Kote-Jarai Z, Saunders EJ, Leongamornlert DA (2013). Fine-mapping identifies multiple prostate cancer risk loci at 5p15, one of which associates with TERT expression. Hum Mol Genet.

[14] Wang Z, Zhu B, Zhang M (2014). Imputation and subset-based association analysis across different cancer types identifies multiple independent risk loci in the TERT-CLPTM1L region on chromosome 5p15.33. Hum Mol Genet.

[15] Ge M, Shi M, An C (2016). Functional evaluation of TERT-CLPTM1L genetic variants associated with susceptibility of papillary thyroid carcinoma. Sci Rep.

[16] Dong J, Cheng Y, Zhu M (2017). Fine mapping of chromosome 5p15.33 identifies novel lung cancer susceptibility loci in Han Chinese. Int J Cancer.

[17] Baron AE, Kako S, Feser WJ (2017). Clinical utility of chromosomal Aneusomy in individuals at high risk of lung Cancer. J Thorac Oncol.

[18] Kachuri L, Latifovic L, Liu G (2016). Systematic review of genetic variation in chromosome 5p15.33 and telomere length as predictive and prognostic biomarkers for lung Cancer. Cancer Epidemiol Biomark Prev.

[19] Mobuchon L, Battistella A, Bardel C, et al. A GWAS in uveal melanoma identifies risk polymorphisms in the CLPTM1L locus. NPJ Genom Med. 2017;2:5. 10.1038/s41525-017-0008-5. Epub 2017 Mar 10. 10.1038/s41525-017-0008-5 PMC5542017 28781888

[20] Ni Z, Chen Q, Lai Y (2016). Prognostic significance of CLPTM1L expression and its effects on migration and invasion of human lung cancer cells. Cancer Biomark.

[21] Clarke WR, Amundadottir L, James MA (2019). CLPTM1L/CRR9 ectodomain interaction with GRP78 at the cell surface signals for survival and chemoresistance upon ER stress in pancreatic adenocarcinoma cells. Int J Cancer.

[22] Yang YC, Fu WP, Zhang J (2018). rs401681 and rs402710 confer lung cancer susceptibility by regulating TERT expression instead of CLPTM1L in east Asian populations. Carcinogenesis..

[23] Jin T, Li B, He N (2016). CLPTM1L polymorphism as a protective factor for lung cancer: a case-control study in southern Chinese population. Tumour Biol.

[24] Zhao MM, Zhang Y, Shen L (2014). Genetic variations in TERT-CLPTM1L genes and risk of lung cancer in a Chinese population. Asian Pac J Cancer Prev.

[25] Zhang Y, Zhao M, Shen L (2014). Genetic polymorphisms of TERT and CLPTM1L and risk of lung cancer: a case-control study in northeast Chinese male population. Med Oncol.

[26] Xun X, Wang H, Yang H, et al: CLPTM1L genetic polymorphisms and interaction with smoking and alcohol drinking in lung cancer risk: a case-control study in the Han population from northwest China. Medicine (Baltimore). 2014; 93:e289. 10.1097/MD.0000000000000289 PMC4603120 25526467

[27] James MA, Vikis HG, Tate E (2014). CRR9/CLPTM1L regulates cell survival signaling and is required for Ras transformation and lung tumorigenesis. Cancer Res.

[28] Hung RJ, Spitz MR, Houlston RS (2019). Lung Cancer risk in never smokers of European descent is associated with genetic variation in the 5P15.33 TERT-CLPTM1L region. J Thorac Oncol.

[29] Lee DH, Heo YR, Park WJ (2017). A TERT-CLPTM1 locus polymorphism (rs401681) is associated with EGFR mutation in non-small cell lung cancer. Pathol Res Pract.

[30] Chen Z, Wang J, Bai Y (2017). The associations of TERT-CLPTM1L variants and TERT mRNA expression with the prognosis of early stage non-small cell lung cancer. Cancer Gene Ther.

[31] Sun Y, Zhang YJ, Kong XM (2013). No association of XRCC1 and CLPTM1L polymorphisms with non-small cell lung cancer in a non-smoking Han Chinese population. Asian Pac J Cancer Prev.

[32] Vahrenkamp JM, Yang CH, Rodriguez AC (2018). Clinical and genomic crosstalk between glucocorticoid receptor and estrogen receptor alpha in endometrial Cancer. Cell Rep.

[33] Magne Nde CB, Casas Gimeno G, Docanto M (2018). Timeless is a novel estrogen receptor co-activator involved in multiple signaling pathways in MCF-7 cells. J Mol Biol.

[34] Jia M, Dahlman-Wright K, Gustafsson JA (2015). Estrogen receptor alpha and beta in health and disease. Best Pract Res Clin Endocrinol Metab.

[35] Yi P, Wang Z, Feng Q (2017). Structural and functional impacts of ER Coactivator sequential recruitment. Mol Cell.

[36] Lecomte S, Chalmel F, Ferriere F (2017). Glyceollins trigger anti-proliferative effects through estradiol-dependent and independent pathways in breast cancer cells. Cell Commun Signal..

[37] Zhang J, Guan X, Liang N (2018). Estrogen-related receptor alpha triggers the proliferation and migration of human non-small cell lung cancer via interleukin-6. Cell Biochem Funct.

[38] Yu W, Ding J, He M (2018). Estrogen receptor beta promotes the vasculogenic mimicry (VM) and cell invasion via altering the lncRNA-MALAT1/miR-145-5p/NEDD9 signals in lung cancer. Oncogene..

[39] Yu N, Dou L, Li Y, et al: Roles of ERalpha and ERbeta in estrogen-induced DDP chemoresistance in non-small cell lung cancer. Genet Mol Res. 2016;15(3). 10.4238/gmr.15038995. 10.4238/gmr.15038995 27706665

[40] Pelekanou V, Anastasiou E, Bakogeorgou E (2019). Estrogen receptor-alpha isoforms are the main estrogen receptors expressed in non-small cell lung carcinoma. Steroids..

[41] Lund-Iversen M, Scott H, Strom EH (2018). Expression of estrogen receptor-alpha and survival in advanced-stage non-small cell lung Cancer. Anticancer Res.

[42] Cheng TD, Darke AK, Redman MW (2018). Smoking, sex, and non-Small cell lung Cancer: steroid hormone receptors in tumor tissue (S0424). J Natl Cancer Inst.

[43] Li W, Tse LA, Wang F (2015). Prognostic value of estrogen receptors mRNA expression in non-small cell lung cancer: a systematic review and meta-analysis. Steroids..

[44] Ma L, Zhan P, Liu Y (2016). Prognostic value of the expression of estrogen receptor beta in patients with non-small cell lung cancer: a meta-analysis. Transl Lung Cancer Res.

[45] Nikolos F, Thomas C, Bado I (2018). ERbeta sensitizes NSCLC to chemotherapy by regulating DNA damage response. Mol Cancer Res.

[46] Gao X, Cai Y, Wang Z (2019). Estrogen receptors promote NSCLC progression by modulating the membrane receptor signaling network: a systems biology perspective. J Transl Med.

[47] Shen L, Li Z, Shen S (2012). The synergistic effect of EGFR tyrosine kinase inhibitor gefitinib in combination with aromatase inhibitor anastrozole in non-small cell lung cancer cell lines. Lung Cancer.

[48] Huang Q, Zhang Z, Liao Y (2018). 17beta-estradiol upregulates IL6 expression through the ERbeta pathway to promote lung adenocarcinoma progression. J Exp Clin Cancer Res.

[49] Chen X, Ma N, Zhou Z (2017). Estrogen receptor mediates the Radiosensitivity of triple-negative breast Cancer cells. Med Sci Monit.

[50] Russell JO, Lu WY, Okabe H (2019). Hepatocyte-specific beta-catenin deletion during severe liver injury provokes cholangiocytes to differentiate into hepatocytes. Hepatology..

[51] Ikeda K, Horie-Inoue K, Inoue S (2015). Identification of estrogen-responsive genes based on the DNA binding properties of estrogen receptors using high-throughput sequencing technology. Acta Pharmacol Sin.

[52] Gade AB, Bagle PN, Shinde PS (2018). Catalytic Enantioselective 1,3-alkyl shift in alkyl aryl ethers: efficient synthesis of optically active 3,3′-Diaryloxindoles. Angew Chem Int Ed Engl.

[53] Lau KM, Chan QK, Pang JC (2012). Overexpression of HMGA1 deregulates tumor growth via cdc25A and alters migration/invasion through a cdc25A-independent pathway in medulloblastoma. Acta Neuropathol.

[54] Kendrick S, Kang HJ, Alam MP (2016). Correction to "the dynamic character of the BCL2 promoter i-motif provides a mechanism for modulation of gene expression by compounds that bind selectively to the alternative DNA hairpin structure". J Am Chem Soc.

[55] Hyder SM, Stancel GM, Nawaz Z (1992). Identification of an estrogen response element in the 3′-flanking region of the murine c-fos protooncogene. J Biol Chem.

[56] Liu Q, Bai X, Li H (2013). The oncoprotein HBXIP upregulates Lin28B via activating TF II D to promote proliferation of breast cancer cells. Int J Cancer.

[57] Wilson KE, Bachawal SV, Willmann JK (2018). Intraoperative resection guidance with Photoacoustic and fluorescence molecular imaging using an anti-B7-H3 antibody-Indocyanine green dual contrast agent. Clin Cancer Res.

[58] Fu Q, Song X, Liu Z (2017). miRomics and proteomics reveal a miR-296-3p/PRKCA/FAK/Ras/c-Myc feedback loop modulated by HDGF/DDX5/beta-catenin complex in lung adenocarcinoma. Clin Cancer Res.

[59] Ling C, Xie Y, Zhao D (2012). Enhanced radiosensitivity of non-small-cell lung cancer (NSCLC) by adenovirus-mediated ING4 gene therapy. Cancer Gene Ther.

[60] Qian H, Chen L, Huang J (2018). The lncRNA MIR4435-2HG promotes lung cancer progression by activating beta-catenin signalling. J Mol Med (Berl).

[61] Fan S, Meng Q, Xu J (2013). DIM (3,3′-diindolylmethane) confers protection against ionizing radiation by a unique mechanism. Proc Natl Acad Sci U S A.

[62] Haiman CA, Chen GK, Vachon CM (2011). A common variant at the TERT-CLPTM1L locus is associated with estrogen receptor-negative breast cancer. Nat Genet.

[63] Jia J, Bosley AD, Thompson A (2014). CLPTM1L promotes growth and enhances aneuploidy in pancreatic cancer cells. Cancer Res.

[64] Zhang M, Wu X, Lu W (2015). Rs401681 polymorphism in TERT-CLPTM1L was associated with bladder cancer risk: a meta-analysis. Iran J Basic Med Sci.

[65] Azad AK, Qiu X, Boyd K, et al: A genetic sequence variant (GSV) at susceptibility loci of 5p15.33 (TERT-CLPTM1L) is associated with survival outcome in locally advanced and metastatic non-small-cell lung cancer (NSCLC). Lung Cancer. 2014; 84:289–294. 10.1016/j.lungcan.2014.03.008 24679952

[66] Adamidou T, Arvaniti KO, Glykos NM (2018). Folding simulations of a nuclear receptor box-containing peptide demonstrate the structural persistence of the LxxLL motif even in the absence of its cognate receptor. J Phys Chem B.

[67] Paramanik V, Krishnan H, Kumar Thakur M (2018). Estrogen receptor alpha- and beta-interacting proteins contain consensus secondary structures: An Insilico study. Ann Neurosci.

[68] Gyorffy B, Lanczky A, Eklund AC (2010). An online survival analysis tool to rapidly assess the effect of 22,277 genes on breast cancer prognosis using microarray data of 1,809 patients. Breast Cancer Res Treat.

[69] Li H, Jiang M, Cui M (2019). et al. MiR-365 enhances the radiosensitivity of non-small cell lung cancer cells through targeting CDC25A. Biochem Biophys Res Commun.

[70] Nguyen HD, Phan TT, Carraz M (2012). Estrogen receptor alpha/beta-cofactor motif interactions; interplay of tyrosine 537/488 phosphorylation and LXXLL motifs. Mol BioSyst.

[71] Brower V (2017). Adding radiotherapy to chemotherapy in advanced NSCLC. Lancet Oncol.

[72] Dai Y, Wei Q, Schwager C (2018). Oncogene addiction and radiation oncology: effect of radiotherapy with photons and carbon ions in ALK-EML4 translocated NSCLC. Radiat Oncol.

[73] Ma R, Karthik GM, Lovrot J (2017). Estrogen receptor beta as a therapeutic target in breast Cancer stem cells. J Natl Cancer Inst.

[74] Tarallo R, Giurato G, Bruno G (2017). The nuclear receptor ERbeta engages AGO2 in regulation of gene transcription. RNA splicing and RISC loading Genome Biol.

[75] Andersson S, Sundberg M, Pristovsek N (2017). Insufficient antibody validation challenges oestrogen receptor beta research. Nat Commun.

[76] Verma MK, Miki Y, Abe K (2012). Cytoplasmic estrogen receptor beta as a potential marker in human non-small cell lung carcinoma. Expert Opin Ther Targets.

[77] Fang F, Zheng Q, Zhang J (2013). Testicular orphan nuclear receptor 4-associated protein 16 promotes non-small cell lung carcinoma by activating estrogen receptor beta and blocking testicular orphan nuclear receptor 2. Oncol Rep.

[78] Zhao G, Zhao S, Wang T (2011). Estrogen receptor beta signaling regulates the progression of Chinese non-small cell lung cancer. J Steroid Biochem Mol Biol.

[79] Wang Z, Li Z, Ding X (2015). ERbeta localization influenced outcomes of EGFR-TKI treatment in NSCLC patients with EGFR mutations. Sci Rep.

[80] Ma S, Zhang WL, Leckey BD (2018). X-ray irradiation induced Disabled-2 gene promoter de-methylation enhances radiosensitivity of non-small-cell lung carcinoma cells. J Exp Clin Cancer Res.

[81] Feng X, Lv W (2018). Wang S, et al: miR495 enhances the efficacy of radiotherapy by targeting GRP78 to regulate EMT in nasopharyngeal carcinoma cells. Oncol Rep.

[82] Jun S, Jung YS, Suh HN (2016). LIG4 mediates Wnt signalling-induced radioresistance. Nat Commun.

[83] Zhang P, Wang L, Rodriguez-Aguayo C (2014). miR-205 acts as a tumour radiosensitizer by targeting ZEB1 and Ubc13. Nat Commun.

[84] Lin J, Liu Z, Liao S, et al. Elevation of long non-coding RNA GAS5 and knockdown of microRNA-21 up-regulate RECK expression to enhance esophageal squamous cell carcinoma cell radio-sensitivity after radiotherapy. Genomics. 2019;112:2173–85. 10.1016/j.ygeno.2019.12.013 31866421

[85] Lim VW, Lim WY, Zhang Z (2014). Serum estrogen receptor beta mediated bioactivity correlates with poor outcome in lung cancer patients. Lung Cancer.

[86] Tang H, Liao Y, Zhang C (2014). Fulvestrant-mediated inhibition of estrogen receptor signaling slows lung cancer progression. Oncol Res.

[87] Skjefstad K, Grindstad T, Khanehkenari MR (2016). Prognostic relevance of estrogen receptor alpha, beta and aromatase expression in non-small cell lung cancer. Steroids..

[88] Khurana S, Chakraborty S, Zhao X (2012). Identification of a novel LXXLL motif in alpha-actinin 4-spliced isoform that is critical for its interaction with estrogen receptor alpha and co-activators. J Biol Chem.

[89] Hu B, Wang X (2017). Hu S, et al: miR-21-mediated Radioresistance occurs via promoting repair of DNA double Strand breaks. J Biol Chem.

[90] Fu S, Jin L, Gong T (2018). Effect of sinomenine hydrochloride on radiosensitivity of esophageal squamous cell carcinoma cells. Oncol Rep.

[91] Li CH, Lim SH, Ryu HH (2016). Enhancement of radiosensitivity by inhibition of c-Jun N-terminal kinase activity in a Lewis lung carcinomabearing subcutaneous tumor mouse model. Oncol Rep.

[92] Liang J, Cao R, Wang X (2017). Mitochondrial PKM2 regulates oxidative stress-induced apoptosis by stabilizing Bcl2. Cell Res.

[93] Edison N, Curtz Y, Paland N (2017). Degradation of Bcl-2 by XIAP and ARTS promotes apoptosis. Cell Rep.

[94] Seo JH, Lee KN, Park SH (2001). Retinoic acid as a Radiosensitizer on the head and neck squamous cell carcinoma cell lines. Cancer Res Treat.

[95] Xue J, Zhu W, Song J (2018). Activation of PPARalpha by clofibrate sensitizes pancreatic cancer cells to radiation through the Wnt/beta-catenin pathway. Oncogene..

[96] Chen L, Zhu Z, Gao W (2017). Systemic analysis of different colorectal cancer cell lines and TCGA datasets identified IGF-1R/EGFR-PPAR-CASPASE axis as important indicator for radiotherapy sensitivity. Gene..

[97] Yang Y, Leonard M, Zhang Y (2018). HER2-driven breast tumorigenesis relies upon interactions of the estrogen receptor with Coactivator MED1. Cancer Res.

[98] Li Z, Pu Z, Fan J (2018). Fine mapping in TERT-CLPTM1L region identified three independent lung cancer susceptibility signals: a large-scale multi-ethnic population study. Mol Carcinog.

