# Supplementary material for: CLPTM1L induces estrogen receptor β signaling-mediated radioresistance in non-small cell lung cancer cells
Source: Cell Commun Signal. 2020 Sep 17;18:152. doi: 10.1186/s12964-020-00571-4 (PMC7499972; doi:10.1186/s12964-020-00571-4)
Supplement: Supplementary file 2 — Additional file 1: Fig. S1. CLPTM1L induces the radioresistance of NSCLC cells. Fig. S2. Positive correlation of CLPTM1L with the development of NSCLC. Fig. S3. High-expressed CLPTM1L target genes are associated with poor patient overall survival. Fig. S4. CLPTM1L upregulates the levels of CDC25A, c-Jun, and BCL2 through ERβ in NSCLC cells. Fig. S5. CLPTM1L activates the promoter of ERβ-induced genes by stimulating ERβ in HEK293T cells. Fig. S6. CLPTM1L induces radioresistance of NSCLC cells by coactivating ERβ. Fig. S7. CLPTM1L coactivates ERβ independent of estrogen. Table S1. Primers and siRNAs using for relative experiments. Table S2. CLPTM1L target genes. CLPTM1L target genes after screening. Table S4. Irradiation-related genes. Table S5. Mass spectrographic analysis of CLPTM1L. Table S6. ERE-containing genes (ERβ-responsive genes). Table S7. Combined analysis of CLPTM1L-modulated genes, IR-related genes, and ERE containing genes. Table S8. Cross tabulation analysis of CLPTM1L and CDC25A in NSCLC tissues. Table S9. Cross tabulation analysis of CLPTM1L and c-Jun in NSCLC tissues. Table S10. Cross tabulation analysis of CLPTM1L and BCL2 in NSCLC tissues. Table S11. Non-small cell lung carcinoma & Normal TMA. [file 12964_2020_571_MOESM2_ESM.doc]

**Supplementary materials and methods.** Materials and Methods for iTRAQ-based analysis, Gene expression (cDNA) microarray analysis, Mass spectrographic analysis

*iTRAQ-based analysis*

The A549 cells were divided into four groups and treated with 4 Gy IR: (1) pcDNA transfection 1 (control1); (2) pcDNA transfection 2 (control2); (3) pcDNA-CLPTM1L transfection 1 (CLPTM1L1); (4) pcDNA-CLPTM1L transfection 2 (CLPTM1L2). The analysis of iTRAQ-based study for CLPTM1L in A549 cells was performed as follows (The Beijing Genomics Institute, China):

1. The lysis buffer was added to the cell sample (control1; control2; CLPTM1L1; CLPTM1L2). After ultrasonic treatment for 5 min and centrifugation for 30 minutes, supernatant was taken for next step;
2. Protein was quantified by bradford method;
3. The protein sample was added to the 10K ultrafiltration tube, centrifuged at 14000 g, 4 °C for 40 min, and the waste liquid was discarded;
4. 50% TEAB was added to the sample, centrifuged at 14000 g, 4 °C for 40 min, then the waste liquid was discarded;
5. Added trypsin to the sample, 37 °C water bath for 24 h, freeze-dried the solution and TEAB buffer was used to dissolve the peptide;
6. Balanced the labeling reagent to room temperature, and added isopropanol to the reagent for centrifugation;
7. Mixed labeling reagents with the peptide, and different samples were labeled with different sizes of isotopes;
8. The labeling sample was diluted 10 times with solution A, centrifuged at 15000 g for 10 min to obtaine the supernatant;
9. Ran the sample according to the supposed separation gradient inm order to establish the pre-separated chromatogram. The purified peptides were used for mass spectrographic analysis;
10. Desalted peptide mixture were loaded onto a Acclaim PePmap C18-reversed phase column （75 μm×2 cm, 3μm, 100 Ǻ thermo scientific) and separated with reversed phase C18 column (75 μm×10 cm, 5 μm, 300 Ǻ, Agela Technologies) mounted on a Dionex ultimate 3000 nano LC system. Peptides were eluted using a gradient of 5–80% (v/v) acetonitrile in 0.1% formic acid over 45 min at a flow rate of 300 nL min-1 combined with a Q Exactive mass spectrometer (Thermo Fisher Scientific, MA, USA). The eluates were directly entered Q－Exactive MS (Thermo Fisher Scientific, Waltham, MA, USA), setting in positive ion mode and data-dependent manner with full MS scan from 350-2000 m/z, full scan resolution at 70,000, MS/MS scan resoltion at 17,500. MS/MS scan with minimum signal threshold 1E+5, isolation width at 2 Da. To evaluate the performance of this mass spectrometry on the iTRAQ labeled samples, two MS/MS acquisition modes, higher collision energy dissociation (HCD) was employed. And to optimize the MS/MS acquisition efficiency of HCD, normalized collision energy (NCE) was systemically examined 28, steped 20%.

*Gene expression (cDNA) microarray analysis*

The A549 cells were divided into two groups: (1) control group (cells without IR): (2) IR group (cells exposed to 4 Gy IR). Total RNA was isolated from the cells and cleaned-up for the following analysis:

1. Denatured the primer and the template by incubating the reaction at 65 °C in a circulating water bath for 10 min, and placed the reactions on ice and incubated for 5 min;
2. Added cDNA Master Mix to each sample tube and mixed by pipetting up and down;
3. Incubated samples in a 40 °C circulating water bath for 2 h, 70 °C circulating water bath for 15 min. Then moved samples to ice for 5 min;
4. Added Transcription Master Mix to each sample tube. Incubated samples in a circulating water bath at 40 °C for 2 h to obtaine the labeled cRNA;
5. Purified the labeled cRNA using relative Kit;
6. Added nuclease-free water to the vial containing lyophilized 10x Blocking Agent. Mixed by gently vortexing and incubated at 60 °C for exactly 30 min to fragment RNA;
7. Added 2x GEx Hybridization Buffer to the sample and mixed well;
8. The two groups of reactions were loaded on the arrays in a hybridization oven set to 65 °C and rotated at 10 rpm. Then hybridized at 65 °C for 17 h;
9. Washed the microarrays using relative buffer and scanned arrays with Agilent Microarray Scanner;
10. Extracted the data of irradiation-related genes (differentially expressed genes between the control group and IR group) using Agilent Feature Extraction software;
11. Experiments were repeated three times.

*Mass spectrographic analysis*

Co-IP assay was performed using A549 cells treated with 4 Gy IR. The antibody used was rabbit anti-CLPTM1L (Abcam, Cat NO.: ab198862). The precipitates were washed, resuspended and subjected to SDS-PAGE. The Mass spectrographic analysis was performed as follows (The Beijing Genomics Institute, China):

1. Cut off the glue spots that need to be analyzed, put them into the centrifuge tube, and numbered them;

2. Added 1 ml water to each centrifuge tube, washed for 10 minutes, removed the water and repeated;

3. Added 1ml glue to each centrifuge tube to digest the decolorizing solution, washed for 10 minutes, and moved the decolorizing solution;

4. Added acetonitrile to dehydrate until the colloidal particles were completely whitened and vacuum dried acetonitrile;

5. Added 10 mM DTT to allow the colloidal particles to be absorbed completely, put them in a 56 °C water bath and incubated for 1 h;

6. After incubation, removed excess DTT liquid and added 55 mM IAM, darkroom to incubate at room temperature for 45 min;

7. After incubation, removed the excess IAM liquid, added 25 mM ammonium bicarbonate, washed for 10 min and repeated the cleaning;

8. Removed ammonium bicarbonate, added decolorizing solution and washed for 10 minutes, and repeated;

9. Added acetonitrile to dehydrate until the colloidal particles were completely whitened, the acetonitrile was dried in vacuum;

10. The enzyme storage solution was added to the dehydrated colloidal particles so that the colloidal particles could be fully absorbed. Then added 25 mM ammonium bicarbonate and put it in a 37 °C water bath and digested overnight;

11. After overnight, the FA kit was added to terminate the digestion;

12. Sample was loaded on the machine for spectrographic analysis;

13. The spectrographic analysis was performed according to the 10th step of iTRAQ-based analysis.

**Supplementary** **Figure 1** CLPTM1L induces the radioresistance of NSCLC cells.

(A and B) The protein levels of CLPTM1L in A549 and H460 cells after γ-ray IR was detected by western blot analysis.

(C and D) The expression level of CLPTM1L in cytoplasm (C) and nuclei (D) of NSCLC cells exposed to IR or not was detected by western blot analysis.

(E and F) Representative immunofluorescence images of the localization of CLPTM1L in A549 and H460 cells exposed to IR or not.

(G) The effect of CLPTM1L siRNA and/or 4 Gy of IR on the cell proliferation was tested by MTT in A549 cells.

(H) The effect of CLPTM1L and/or 4 Gy of IR on the cell proliferation was measured by MTT in H460 cells.

(I and J) Cell apoptosis level was examined using apoptosis assay and quantified by Image J software.

(K) The interference efficiency of CLPTM1L siRNA was detected by western blot analysis in A549 cells exposed to IR or not.

(L) The transfection efficiency of pcDNA-CLPTM1L was tested by western blot analysis in H460 cells exposed to IR or not. The data presented are from three independent experiments; Student’s *t* test; * *P* < 0.05; ** *P* < 0.01.

**Supplementary** **Figure 2** Positive correlation of CLPTM1L with the development of NSCLC.

(A) CLPTM1L expression levels from different tumor types plotted using RNA-seq data from Barretina dataset.

(B) Expression level of CLPTM1L in different types of NSCLC obtained from public databases (TCGA dataset).

**Supplementary** **Figure 3** High-expressed CLPTM1L target genes are associated with poor patient overall survival.

(A and B) Real-time PCR analysis of the mRNA levels of ERβ, CDC25A, c-Jun, and BCL2 in A549 and H460 cells treated with pcDNA- ERβ or/and E2 (17β-estradiol). All experiments were repeated at least three times. Statistically significant differences are indicated. Student’s *t* test; * *P* < 0.05; ** *P* < 0.01.

(C–D) Kaplan-Meier plots of overall survival of patients, stratified by expressions of c-Jun and BCL2. Data was obtained from the Kaplan-Meier plotter database.

(E–F) The levels of c-Jun and BCL2 in different lung cancer tissues were obtained using public datasets (Bhattacharjee dataset).

**Supplementary** **Figure 4** CLPTM1L upregulates the levels of CDC25A, c-Jun, and BCL2 through ERβ in NSCLC cells.

(A) The protein levels of CLPTM1L, CDC25A, c-Jun, and BCL2 were tested by western blot analysis in A549 cells.

(B) The mRNA levels of above genes were detected by real-time PCR analysis in A549 cells.

(C) The protein levels of above genes were examined using western blot analysis in H460 cells.

(D) The mRNA levels of above genes were detected by real-time PCR analysis in H460 cells exposed to 4 Gy IR.

(E–H) The mRNA and protein levels of CLPTM1L, ERβ, CDC25A, c-Jun, and BCL2 were measured by real-time PCR and western blot analysis in A549 cells treated with relative siRNAs/plasmids and exposed to 4 Gy IR. All experiments were repeated at least three times. Statistically significant differences are indicated. Student’s *t* test; * *P* < 0.05; ** *P* < 0.01.

**Supplementary** **Figure 5** CLPTM1L activates the promoter of ERβ-induced genes by stimulating ERβ in HEK293T cells.

(A–I) Cells were transfected with relative plasmids and siRNAs, exposed to 4 Gy IR, followed by relative analysis.

(A) The activities of ERE-LUC were examined by luciferase reporter gene assays in HEK293T cells transfected with pcDNA-CLPTM1L plasmids (E2 was used as the positive control).

(B) The ERE-LUC activities were determined using luciferase reporter gene assays in HEK293T cells treated with various doses of pcDNA-CLPTM1L plasmids.

(C) The luciferase activities were examined by luciferase reporter gene assays in the ERE-LUC with wild-type or mutant binding sites of ERβ in HEK293T cells (E2 was used as the positive control).

(D) The luciferase activities of the ERE-LUC were detected by luciferase reporter gene assays in HEK293T cells transfected with pcDNA-CLPTM1L and ERβ siRNAs.

(E) The luciferase activities of ERE-LUC were detected by luciferase reporter gene assays in HEK293T cells transfected with pcDNA-ERβ and CLPTM1L siRNAs.

(F) The activities of ERE-LUC were examined by luciferase reporter gene assays in HEK293T cells transfected with pcDNA-CLPTM1L and/or pcDNA-ERβ plasmids.

(G–I) The interference efficiency of relative siRNAs and transfection efficiency of relative plasmids were detected by qPCR analysis in A549, H460 and HEK293T cells. All experiments were repeated at least three times. Student’s *t* test; * *P* < 0.05; ** *P* < 0.01; *** *P* < 0.001.

**Supplementary** **Figure 6** CLPTM1L induces radioresistance of NSCLC cells by coactivating ERβ.

(A and B) Cell apoptosis level was examined using apoptosis assay and quantified by Image J software.

(C) Effect of CLPTM1L siRNAs on cell proliferation after 0 or 4 Gy of IR was examined by EdU assay in A549 cells.

(D) The effect of CLPTM1L siRNAs on radiosensitivities of A549 cells was measured by MTT experiments after 3 days of IR.

(E) The effect of CLPTM1L siRNAs on radiosensitivities of A549 cells was tested by clonogenic cell survival assay after 12 days of IR. The data presented are from three independent experiments; Student’s *t* test; * *P* < 0.05; ** *P* < 0.01.

**Supplementary** **Figure 7** CLPTM1L coactivates ERβ independent of estrogen.

(A) The interference efficiency of CLPTM1L shRNA in tumor cells was detected by western blot analysis in resected xenograft tumors of nude mice.

(B) The interaction of CLPTM1L with ERβ was detected by Co-IP assays in A549 cells exposed to 4 Gy IR.

(C) The occupancy of CLPTM1L in the promoters of CDC25A, c-Jun, and BCL2 was examined by ChIP-qPCR in A549 cells treated with or without 17β-estradiol exposed to 4 Gy IR.

(D) The activities of ERE-LUC were examined by luciferase reporter gene assays in A549 and H460 cells treated with pcDNA-CLPTM1L and/or 17β-estradiol and exposed to 4 Gy IR.

(E) The proliferation level of H460 cells after 4 Gy of IR was tested using clonogenic cell survival assay in the cells treated with pcDNA-CLPTM1L and/or 17β-estradiol after 12 days of IR. The data presented are from three independent experiments; NS, not significant. Student’s *t* test; ** *P* < 0.01; *** *P* < 0.001.


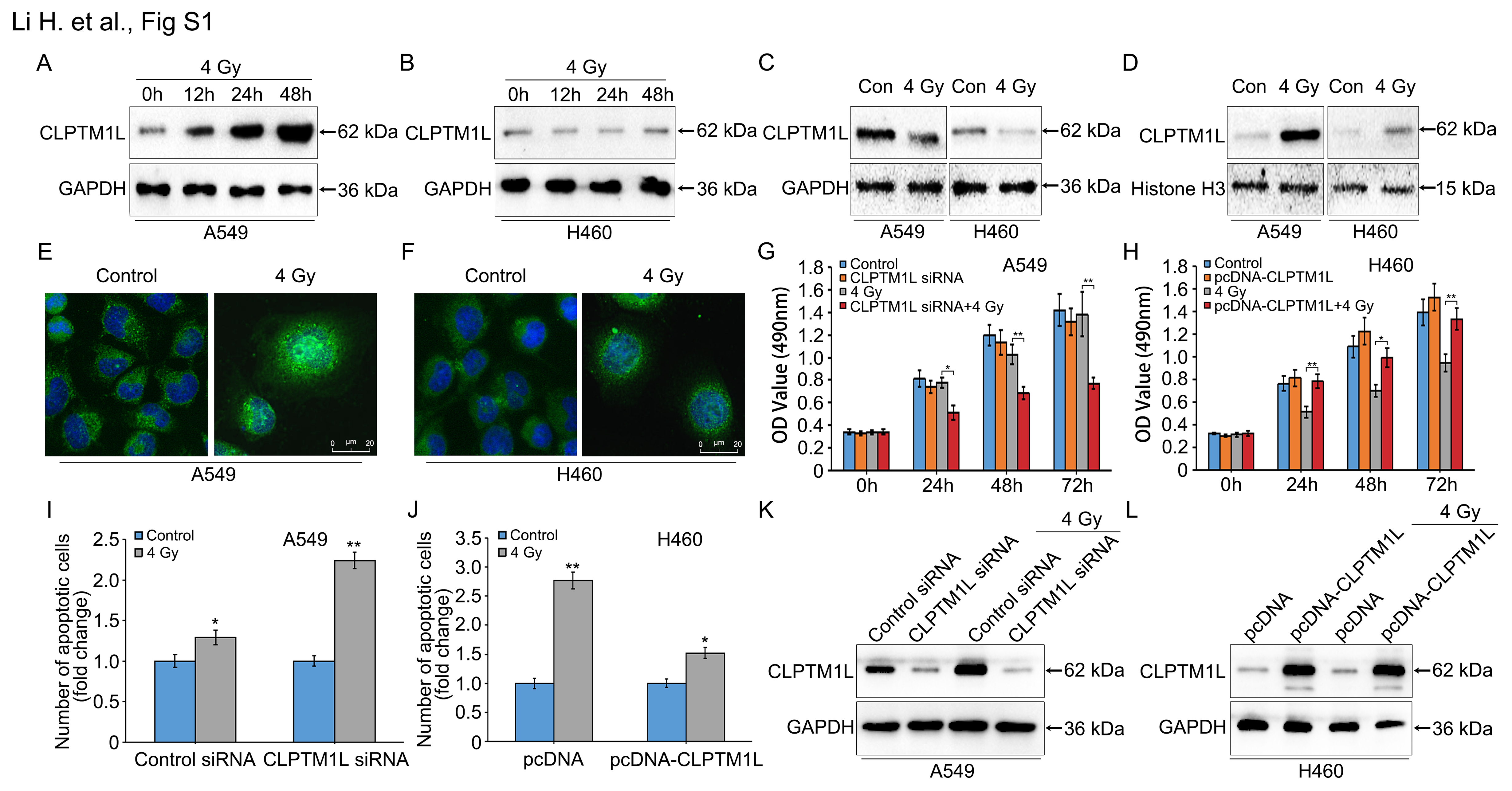


**
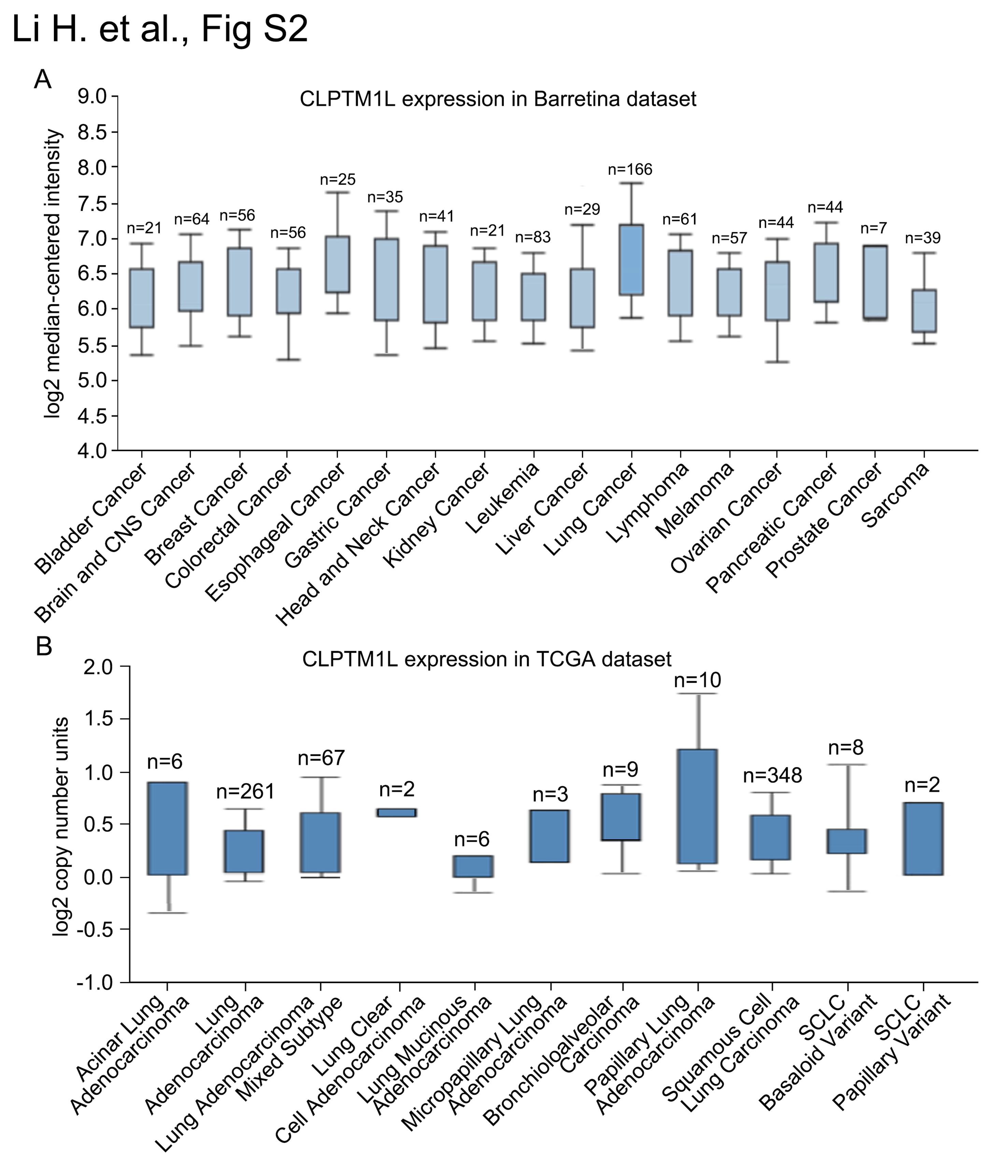
**

**
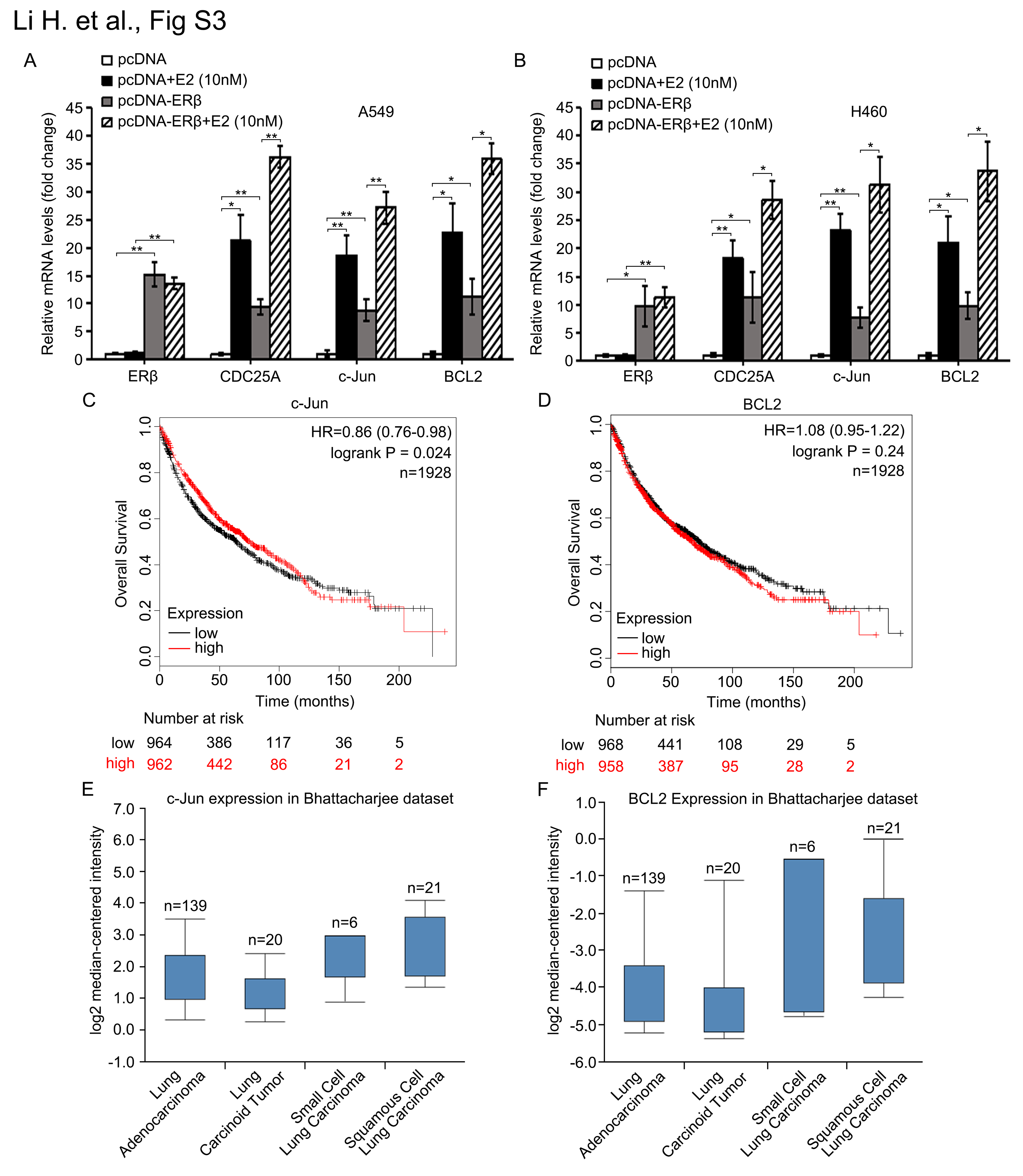
**

**
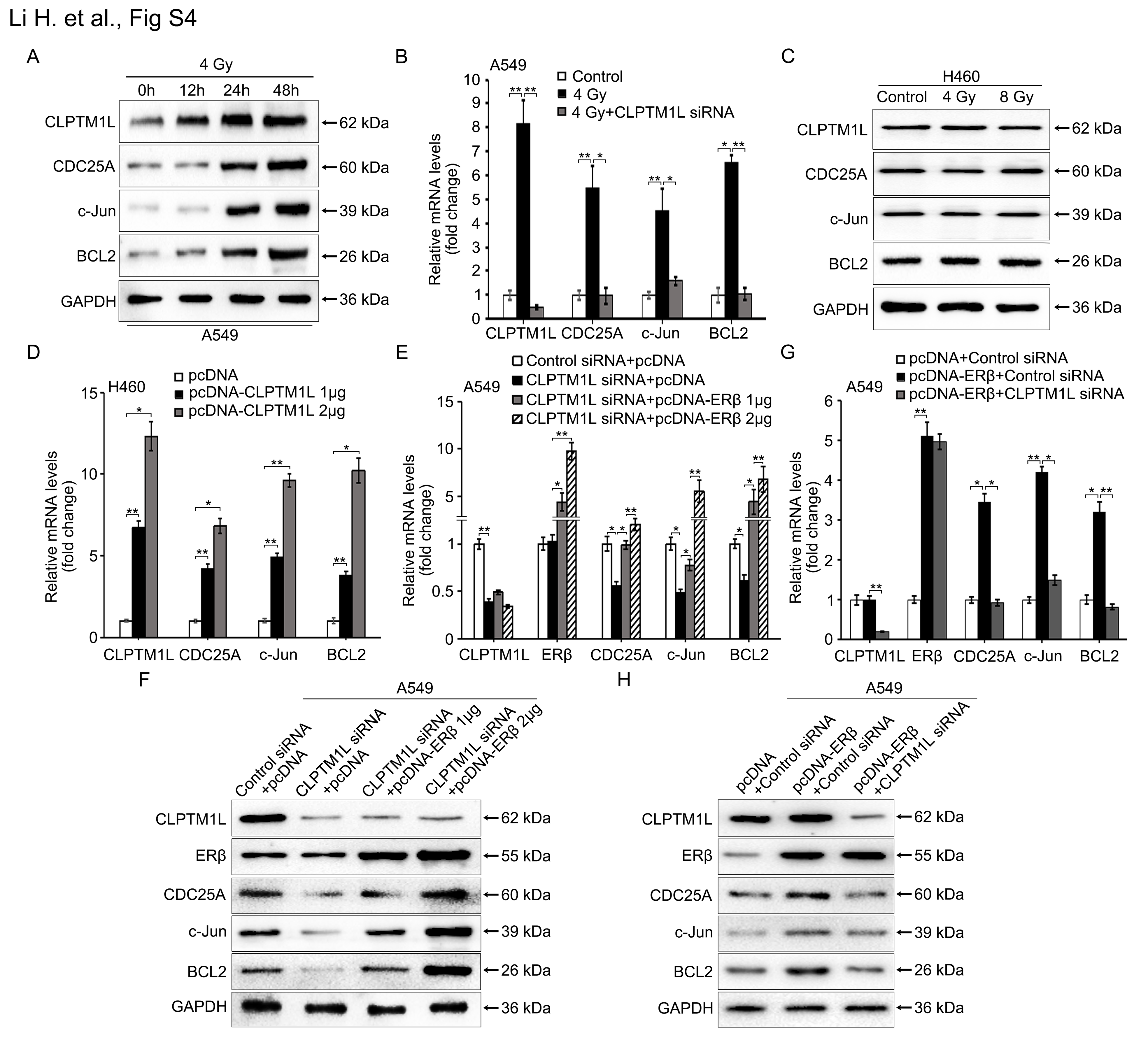
**

**
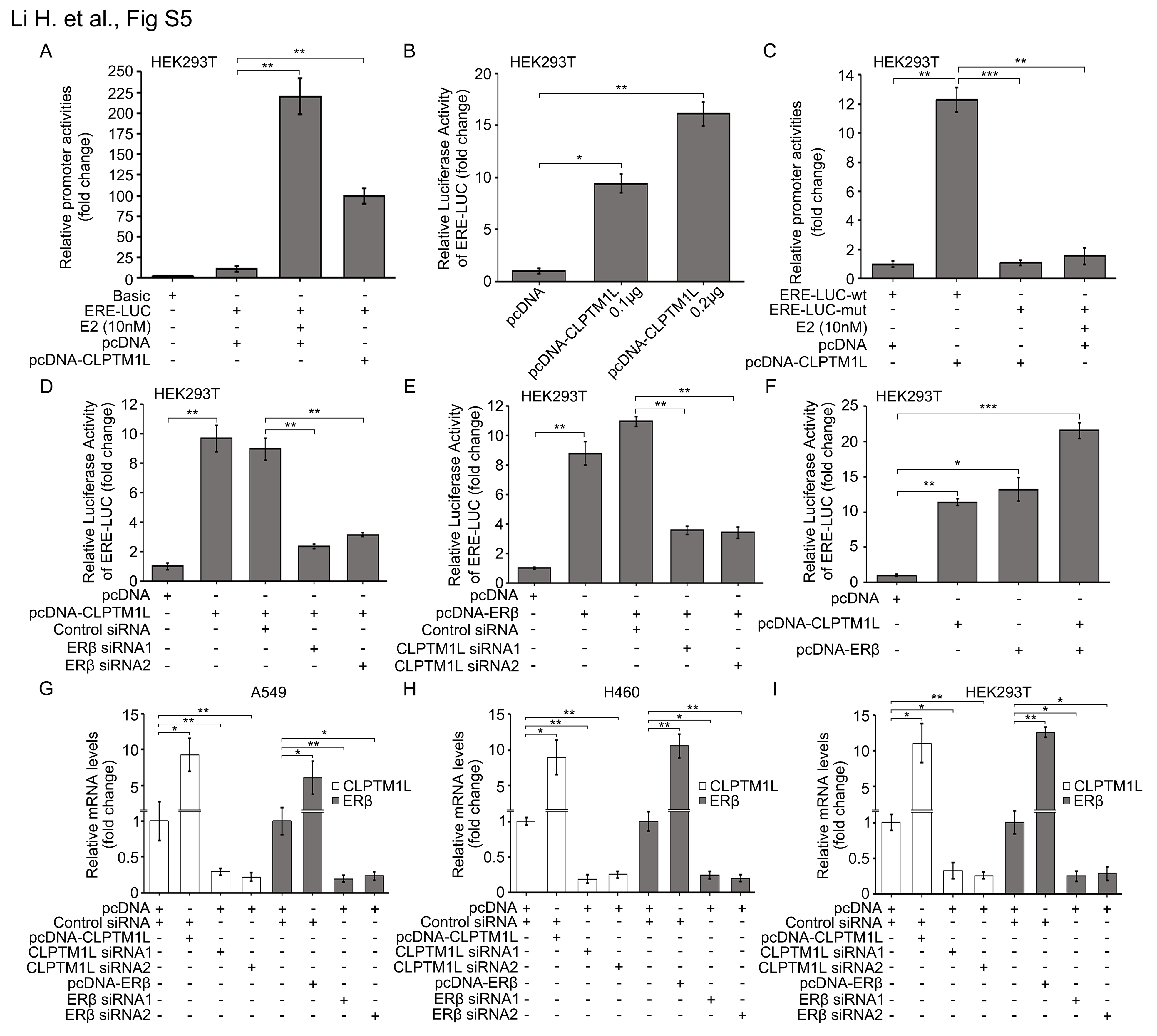
**

**
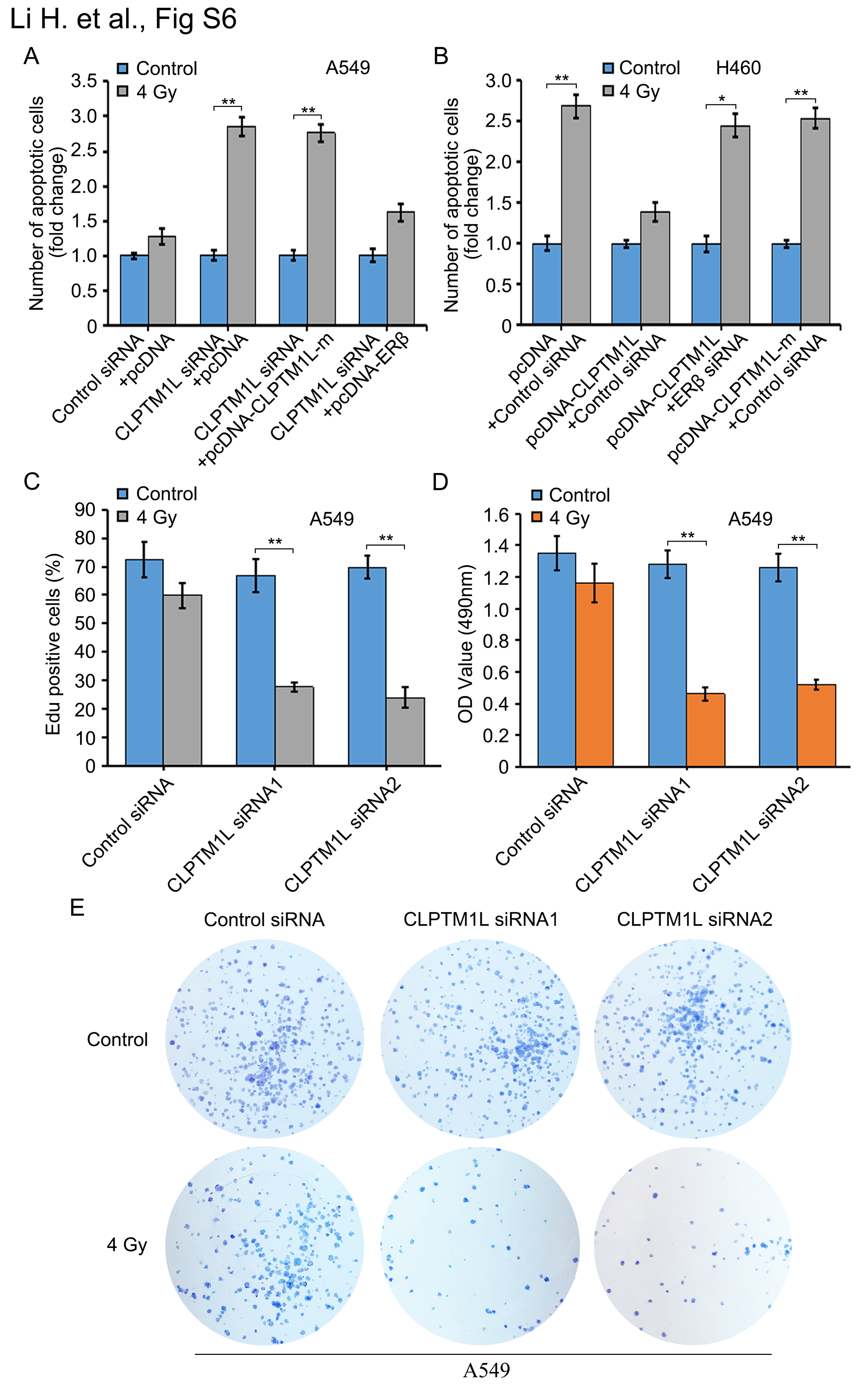
**

**
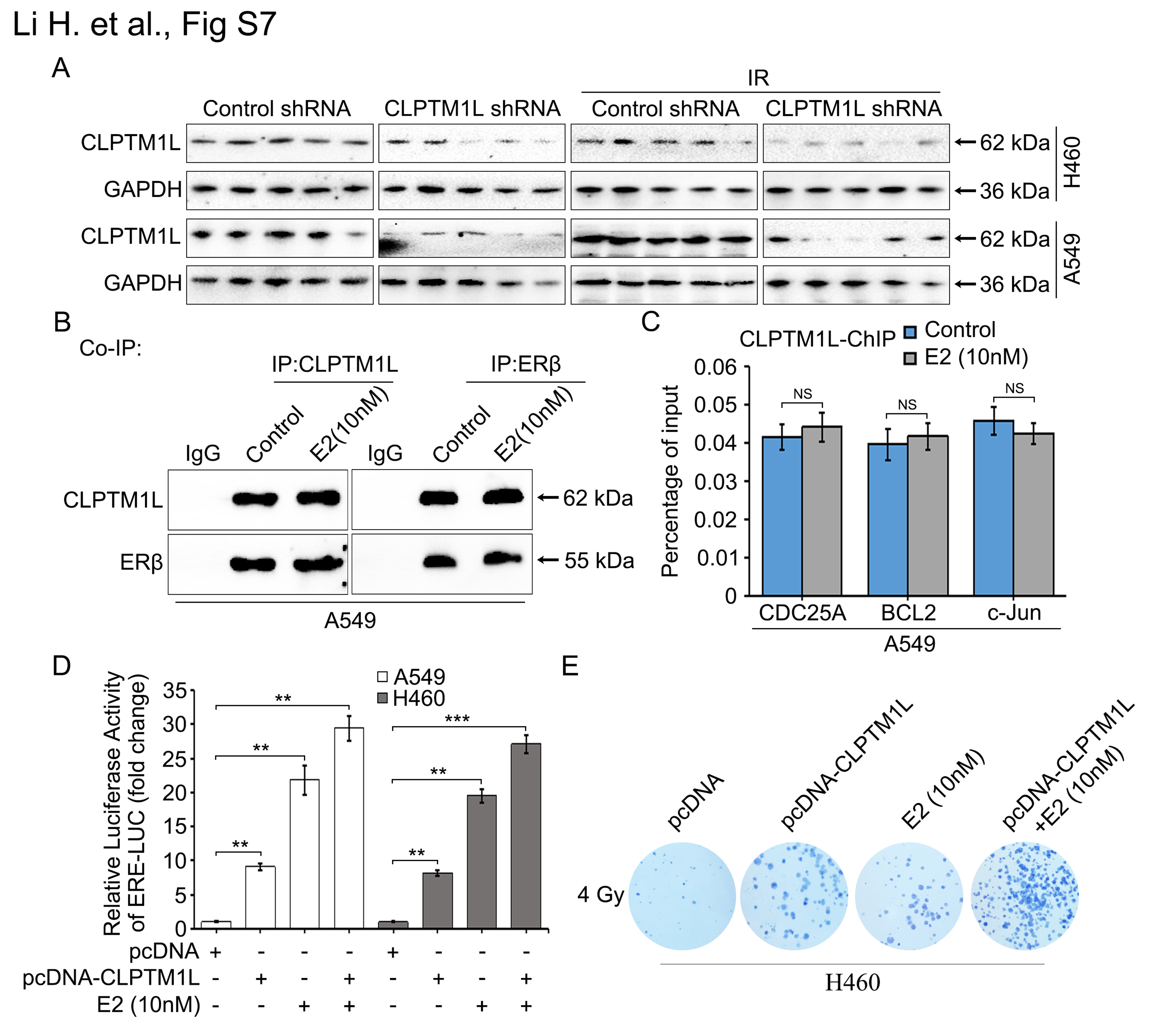
**

**Supplementary** **Table 1.** Primers and siRNAs using for relative experiments

| **Genes** | **Forward (5' to 3')** | **Reverse (5' to 3')** |
| --- | --- | --- |
| *CLPTM1L*  *(qRT)*  *MAPK1*  *(qRT)* | TGGCACTTACAGCGAATC  ACCATCGAGCAAATGAAA | GAGGAAACCAAAGGCATAG  GGTGTTGAGCAGCAGGTT |
| *CDC25A*  *(qRT)* | AACCTTGACAACCGATGC | CAGGGATAAAGACTGATGAA |
| *ATP2B4*  *(qRT)*  *EGFR*  *(qRT)*  *TP53*  *(qRT)*  *MYC*  *(qRT)*  *CDC42BPA (qRT)*  *HRAS*  *(qRT)*  *PGR*  *(qRT)*  *CD44*  *(qRT)*  *FOS*  *(qRT)*  *VEGFA*  *(qRT)*  *BCL2*  *(qRT)*  *c-Jun*  *(qRT)*  *JAK1*  *(qRT)*  *CLPTM1L*  *(wt clone )*  *CLPTM1L*  *(mut clone )*  *CLPTM1L siRNA1*  *CLPTM1L siRNA2*  *CLPTM1L*  *shRNA*    *ERβ*  *siRNA1*  *ERβ*  *siRNA2* | CCACTTCTGTTCCTGCTGT  TGCCCACTACATTGACGG  GTTTCCGTCTGGGCTTCT  GTATTTCTACTGCGACGAGG  GGAACGGTTCAGTCCTCA  GCCATCAACAACACCAAG  GCGTTCCTACCTTGTGGC  ACCAAGAGGCAAGAAACC  TCCGAAGGGAAAGGAATAA  GACGGACAGACAGACAGACA  TGGTCCACCTGACCCTC  CATCGCTGCCTCCAAGT  CTGCCCAAGGACATCAG  CGGGGTACCATGTGGAGCGGCCG  TACCACCACTACCCTCTCTGTGTCG  CGTGACCGATGCTTTGGTT  GCGATTACGCATCGGGATA  CCGGGATTTCCATTTCAGGTGGTTTCTCGAGAAACCACCTGAAATGGAAATCTTTTTTG  GCTTTATCCTGTGGATAAA  GCACTTACAGCGAATCTGA | TTCCTCCTCTTCCTCATCC  GGGATCTTAGGCCCATTC  CAACCTCCGTCATGTGCT  CGAAGGGAGAAGGGTGT  ATGTCTCCACCAGCGATT  GGCATCCTCCACTCCCT  CGCCCTCCGCTTTGTA  AAATGGTGCTGGAGATAAA  CCCAGGTCATCAGGGATC  CCGAAGCGAGAACAGC  CCCACCGAACTCAAAGAA  TGCTGCGTTAGCATGAGTT  TTTCCAAGGTAGCCAAG  CCGGAATTCTCAGTCCGTGTGGGGC  GGTAGTGGTGGTATGACAAGTACTT |

| **Supplementary** **Table 2.** CLPTM1L target genes | | | |  |  |
| --- | --- | --- | --- | --- | --- |
| Gene name | Score | # AAs | MW [kDa] | calc. pI | p_value |
| KRT8 | 2711.12 | 483 | 53.7 | 5.59 | 8.61E-125 |
| HIST1H4A | 1096.62 | 103 | 11.4 | 11.36 | 3.87E-103 |
| ENO1 | 4149.41 | 434 | 47.1 | 7.39 | 4.14E-84 |
| PKM | 3911.09 | 531 | 57.9 | 7.84 | 4.14E-76 |
| RPL15 | 144.17 | 204 | 24.1 | 11.62 | 3.75E-75 |
| AHNAK | 1290.1 | 5890 | 628.7 | 6.15 | 2.73E-68 |
| FLNA | 3914.47 | 2647 | 280.6 | 6.06 | 1.49E-67 |
| KRT19 | 1866.61 | 400 | 44.1 | 5.14 | 1.07E-65 |
| PPP1CB | 601.26 | 149 | 16.8 | 4.22 | 2.37E-65 |
| RPS9 | 276.01 | 194 | 22.6 | 10.65 | 3.86E-64 |
| BASP1 | 353.79 | 227 | 22.7 | 4.63 | 2.36E-61 |
| CFL1 | 705.63 | 166 | 18.5 | 8.09 | 2.63E-56 |
| GAPDH | 5086.65 | 335 | 36 | 8.46 | 3.13E-54 |
| C1QBP | 392.5 | 282 | 31.3 | 4.84 | 3.68E-53 |
| CANX | 744.75 | 592 | 67.5 | 4.6 | 8.99E-51 |
| RPL13A | 146.63 | 203 | 23.6 | 10.93 | 1.25E-49 |
| KRT18 | 1025.98 | 430 | 48 | 5.45 | 1.45E-49 |
| HSPD1 | 3488.45 | 573 | 61 | 5.87 | 4.02E-49 |
| HIST1H2AB | 800.99 | 130 | 14.1 | 11.05 | 1.75E-46 |
| NAMPT | 1098.44 | 491 | 55.5 | 7.15 | 6.61E-46 |
| HIST1H3A | 362.05 | 136 | 15.4 | 11.12 | 5.98E-45 |
| ANXA2 | 2223.88 | 339 | 38.6 | 7.75 | 6.13E-45 |
| RPL6 | 173.39 | 288 | 32.7 | 10.58 | 1.22E-44 |
| RPL18A | 141.87 | 176 | 20.7 | 10.71 | 7.9E-42 |
| PTMA | 349.74 | 110 | 12.1 | 3.79 | 3E-39 |
| NCL | 1477.88 | 710 | 76.6 | 4.7 | 9.08E-39 |
| RPL4 | 586.01 | 427 | 47.7 | 11.06 | 5.95E-38 |
| HSPE1 | 331.92 | 102 | 10.9 | 8.92 | 1.5E-36 |
| HSPA8 | 3621.79 | 646 | 70.9 | 5.52 | 1.15E-35 |
| NQO1 | 1275.94 | 240 | 27.3 | 8.72 | 2.03E-34 |
| PGD | 1009.39 | 470 | 51.8 | 7.44 | 5.7E-33 |
| HSP90AA1 | 4422.65 | 732 | 84.6 | 5.02 | 2.83E-32 |
| RPLP0 | 694.28 | 317 | 34.3 | 5.97 | 3.04E-30 |
| EEF2 | 2062.79 | 858 | 95.3 | 6.83 | 1.26E-29 |
| RPL23 | 395.02 | 140 | 14.9 | 10.51 | 1.37E-29 |
| HNRNPC | 754.03 | 293 | 32.3 | 5.08 | 8.53E-28 |
| YBX1 | 419.96 | 324 | 35.9 | 9.88 | 9.68E-28 |
| VIM | 2656.54 | 466 | 53.6 | 5.12 | 1.46E-27 |
| HSPA5 | 3193.07 | 654 | 72.3 | 5.16 | 5.42E-27 |
| TKT | 1453.71 | 623 | 67.8 | 7.66 | 1.16E-26 |
| RPL7 | 288.58 | 248 | 29.2 | 10.65 | 6.52E-26 |
| PRDX1 | 1781.06 | 199 | 22.1 | 8.13 | 1.28E-25 |
| S100A10 | 80.69 | 97 | 11.2 | 7.37 | 2.64E-25 |
| SET | 233.53 | 266 | 31.1 | 4.23 | 4.31E-25 |
| ACTB | 6135.33 | 375 | 41.7 | 5.48 | 4.55E-25 |
| PPIB | 653.4 | 216 | 23.7 | 9.41 | 1.14E-24 |
| RPS16 | 318.8 | 146 | 16.4 | 10.21 | 1.23E-24 |
| NPM1 | 1586.88 | 294 | 32.6 | 4.78 | 1.65E-24 |
| EEF1A1 | 2107.56 | 462 | 50.1 | 9.01 | 4.6E-23 |
| PGK1 | 1571.74 | 417 | 44.6 | 8.1 | 1.36E-22 |
| ALDOA | 2637.75 | 364 | 39.4 | 8.09 | 2.16E-22 |
| MKI67 | 284.46 | 3256 | 358.5 | 9.45 | 2.35E-22 |
| RPL38 | 138.55 | 70 | 8.2 | 10.1 | 6.37E-22 |
| RPL23A | 270.83 | 156 | 17.7 | 10.45 | 8.64E-22 |
| RPL24 | 85.42 | 157 | 17.8 | 11.25 | 9.09E-21 |
| YWHAE | 1167.21 | 255 | 29.2 | 4.74 | 3.43E-20 |
| RPL28 | 150.75 | 137 | 15.7 | 12.02 | 4.33E-20 |
| PPA1 | 365.18 | 289 | 32.6 | 5.86 | 1.11E-19 |
| RPS12 | 368.5 | 132 | 14.5 | 7.21 | 1.38E-19 |
| GOT2 | 545.93 | 430 | 47.5 | 9.01 | 1.68E-19 |
| LMNA | 2692.44 | 664 | 74.1 | 7.02 | 2.45E-19 |
| GSTM1 | 151.02 | 218 | 25.7 | 6.7 | 9E-19 |
| EIF5A | 534.19 | 154 | 16.8 | 5.24 | 1.72E-18 |
| RPS20 | 148.54 | 119 | 13.4 | 9.94 | 2.1E-18 |
| SUB1 | 151.89 | 127 | 14.4 | 9.6 | 2.88E-18 |
| XRCC6 | 679.16 | 609 | 69.8 | 6.64 | 3.71E-18 |
| RPS17 | 253.18 | 135 | 15.5 | 9.85 | 3.81E-18 |
| RPL18 | 134.03 | 188 | 21.6 | 11.72 | 4.84E-18 |
| LDHB | 839.44 | 334 | 36.6 | 6.05 | 1.19E-17 |
| HSP90B1 | 1324.18 | 803 | 92.4 | 4.84 | 1.94E-17 |
| RPLP2 | 416.05 | 115 | 11.7 | 4.54 | 2.01E-17 |
| ACTN4 | 1586.73 | 911 | 104.8 | 5.44 | 4.64E-17 |
| SLC25A5 | 1047.82 | 298 | 32.8 | 9.69 | 5.49E-17 |
| MYOF | 2090.63 | 2048 | 233.3 | 6.21 | 8.24E-17 |
| RPS8 | 517.21 | 208 | 24.2 | 10.32 | 1.21E-16 |
| SERBP1 | 398.04 | 387 | 42.4 | 8.44 | 1.31E-16 |
| PDIA4 | 765.92 | 645 | 72.9 | 5.07 | 2.74E-16 |
| TPT1 | 168.06 | 172 | 19.6 | 4.93 | 4.68E-16 |
| PGAM1 | 516.45 | 254 | 28.8 | 7.18 | 5.35E-16 |
| KRT10 | 102.13 | 584 | 58.8 | 5.21 | 1.01E-15 |
| AKR1B10 | 1064.02 | 316 | 36 | 7.84 | 1.05E-15 |
| YWHAZ | 926.4 | 245 | 27.7 | 4.79 | 1.2E-15 |
| DDX21 | 588.07 | 783 | 87.3 | 9.28 | 1.52E-15 |
| EEF1B2 | 286.45 | 225 | 24.7 | 4.67 | 3.48E-15 |
| RPS15 | 112.36 | 145 | 17 | 10.39 | 3.86E-15 |
| KRT9 | 70.28 | 623 | 62 | 5.24 | 6.11E-15 |
| HMGB1 | 235.52 | 215 | 24.9 | 5.74 | 7.23E-15 |
| HIST1H2BK | 1305.51 | 126 | 13.9 | 10.32 | 1.04E-14 |
| RPL14 | 116.52 | 215 | 23.4 | 10.93 | 1.07E-14 |
| CAPZA1 | 167.33 | 286 | 32.9 | 5.69 | 1.21E-14 |
| KRT1 | 236.11 | 644 | 66 | 8.12 | 1.3E-14 |
| CAV1 | 79.05 | 178 | 20.5 | 6.02 | 1.75E-14 |
| RPS24 | 125.81 | 130 | 15.1 | 10.89 | 1.99E-14 |
| PSMA4 | 303.09 | 261 | 29.5 | 7.72 | 2.43E-14 |
| CALU | 190.77 | 315 | 37.1 | 4.64 | 2.99E-14 |
| CSRP1 | 353.85 | 193 | 20.6 | 8.57 | 3.74E-14 |
| TALDO1 | 652.53 | 337 | 37.5 | 6.81 | 6.68E-14 |
| EIF4A1 | 1213.38 | 406 | 46.1 | 5.48 | 7.04E-14 |
| CPS1 | 1690.77 | 1500 | 164.8 | 6.74 | 8.8E-14 |
| HSP90AB1 | 3961.16 | 724 | 83.2 | 5.03 | 9.87E-14 |
| RPS7 | 314.51 | 194 | 22.1 | 10.1 | 1.82E-13 |
| P4HB | 1034.4 | 508 | 57.1 | 4.87 | 2.6E-13 |
| DPYSL2 | 555.4 | 572 | 62.3 | 6.38 | 2.64E-13 |
| G6PD | 2146.42 | 515 | 59.2 | 6.84 | 3.58E-13 |
| RPN1 | 434.26 | 607 | 68.5 | 6.38 | 7.59E-13 |
| PRDX6 | 746.99 | 224 | 25 | 6.38 | 2.77E-12 |
| RPS28 | 174.32 | 69 | 7.8 | 10.7 | 3.98E-12 |
| ATP2A2 | 371.68 | 1042 | 114.7 | 5.34 | 4.43E-12 |
| HMGN2 | 89.57 | 90 | 9.4 | 9.99 | 4.49E-12 |
| TPM3 | 1070.74 | 248 | 29 | 4.78 | 6.39E-12 |
| NUDC | 260.38 | 331 | 38.2 | 5.38 | 1.73E-11 |
| FLNB | 2987.58 | 2633 | 281.5 | 5.71 | 1.78E-11 |
| LGALS3 | 1385.09 | 250 | 26.1 | 8.56 | 2.19E-11 |
| RPL26 | 94.35 | 145 | 17.2 | 10.55 | 2.49E-11 |
| RPL27 | 203.94 | 136 | 15.8 | 10.56 | 3E-11 |
| LRPPRC | 1023.03 | 1394 | 157.8 | 6.13 | 3.52E-11 |
| HMGB2 | 42.12 | 209 | 24 | 7.81 | 3.99E-11 |
| RPS3A | 682.07 | 264 | 29.9 | 9.73 | 4.24E-11 |
| KYNU | 250.71 | 465 | 52.3 | 7.03 | 4.94E-11 |
| EEF1A2 | 1405.91 | 463 | 50.4 | 9.03 | 6.14E-11 |
| ANXA1 | 853.11 | 346 | 38.7 | 7.02 | 7.01E-11 |
| ARHGDIA | 112.73 | 204 | 23.2 | 5.11 | 8.65E-11 |
| PDIA3 | 1205.43 | 505 | 56.7 | 6.35 | 1.58E-10 |
| EIF4B | 211.63 | 611 | 69.1 | 5.73 | 1.95E-10 |
| MYH9 | 1836.84 | 1960 | 226.4 | 5.6 | 2.57E-10 |
| PDAP1 | 58.42 | 181 | 20.6 | 8.87 | 3.75E-10 |
| FH | 2484.01 | 641 | 70 | 5.66 | 3.91E-10 |
| MIF | 106.54 | 115 | 12.5 | 7.88 | 8.58E-10 |
| RPL7A | 270.77 | 266 | 30 | 10.61 | 9.54E-10 |
| PFKM | 191.7 | 780 | 85.1 | 7.99 | 1.15E-09 |
| GPI | 615.46 | 558 | 63.1 | 8.32 | 1.27E-09 |
| NDC1 | 5.12 | 542 | 61 | 8.85 | 1.29E-09 |
| RPL9 | 258.83 | 192 | 21.8 | 9.95 | 1.43E-09 |
| PFN1 | 1252.37 | 140 | 15 | 8.27 | 1.44E-09 |
| CALR | 418.65 | 417 | 48.1 | 4.44 | 1.82E-09 |
| HSPA9 | 1805.92 | 679 | 73.6 | 6.16 | 1.87E-09 |
| HDGF | 207.67 | 240 | 26.8 | 4.73 | 2.54E-09 |
| RPS27A | 485.35 | 156 | 18 | 9.64 | 3.97E-09 |
| XRCC5 | 464.7 | 732 | 82.7 | 5.81 | 4.45E-09 |
| PUM2 | 2.62 | 987 | 106.1 | 6.86 | 4.55E-09 |
| TUBB | 3958.56 | 444 | 49.6 | 4.89 | 5.01E-09 |
| ACTN1 | 1345.24 | 892 | 103 | 5.41 | 6.38E-09 |
| GSTP1 | 750.02 | 210 | 23.3 | 5.64 | 6.65E-09 |
| AZIN1 | 0 | 448 | 49.5 | 4.78 | 9.52E-09 |
| INTS7 | 4.12 | 913 | 101.1 | 7.8 | 9.73E-09 |
| HMGN1 | 53.53 | 100 | 10.7 | 9.6 | 1.05E-08 |
| RPLP1 | 226.3 | 114 | 11.5 | 4.32 | 1.05E-08 |
| CAST | 468.82 | 750 | 80.2 | 5.15 | 1.54E-08 |
| ATPIF1 | 27.05 | 106 | 12.2 | 9.35 | 1.54E-08 |
| S100A6 | 336.14 | 90 | 10.2 | 5.48 | 1.83E-08 |
| PSMA1 | 347.2 | 263 | 29.5 | 6.61 | 2.13E-08 |
| HNRNPF | 369.22 | 415 | 45.6 | 5.58 | 0.000000026 |
| TXNRD1 | 1007.06 | 551 | 60.4 | 6.68 | 3.14E-08 |
| SLC3A2 | 341.98 | 529 | 57.9 | 5.35 | 3.31E-08 |
| YWHAB | 849.92 | 244 | 27.8 | 4.83 | 3.47E-08 |
| RPL13 | 234.95 | 211 | 24.2 | 11.65 | 3.55E-08 |
| JAK1 | 2.23 | 1154 | 133.2 | 7.55 | 0.000000036 |
| STMN1 | 218.87 | 149 | 17.3 | 5.97 | 3.78E-08 |
| SERPINB5 | 336.61 | 375 | 42.1 | 6.05 | 4.92E-08 |
| CLCC1 | 3.75 | 366 | 39.8 | 5.74 | 4.95E-08 |
| PSMC1 | 202.07 | 440 | 49.2 | 6.21 | 5.42E-08 |
| SKP1 | 88.88 | 163 | 18.6 | 4.54 | 5.42E-08 |
| GDI2 | 444.63 | 445 | 50.6 | 6.47 | 5.51E-08 |
| RTN4 | 210.59 | 373 | 40.3 | 4.78 | 5.63E-08 |
| DBI | 111.58 | 87 | 10 | 6.57 | 6.09E-08 |
| CGGBP1 | 0 | 167 | 18.8 | 8.95 | 6.21E-08 |
| RPS14 | 306.68 | 151 | 16.3 | 10.05 | 7.17E-08 |
| IKBIP | 35.09 | 377 | 43.1 | 4.92 | 7.19E-08 |
| PA2G4 | 820.46 | 394 | 43.8 | 6.55 | 7.28E-08 |
| PPP2R5A | 2.74 | 486 | 56.2 | 6.71 | 8.42E-08 |
| PRDX3 | 351.62 | 238 | 25.8 | 7.46 | 8.57E-08 |
| MICAL3 | 2.08 | 2002 | 224.2 | 5.55 | 9.28E-08 |
| TUBA1A | 2989.93 | 416 | 46.3 | 5.08 | 9.51E-08 |
| EFHD2 | 377.41 | 240 | 26.7 | 5.2 | 9.52E-08 |
| SERPINB2 | 563.65 | 415 | 46.6 | 5.63 | 0.000000102 |
| HSPH1 | 600.06 | 814 | 92.1 | 5.55 | 0.000000109 |
| PCNA | 352.36 | 261 | 28.8 | 4.69 | 0.000000111 |
| COX7B | 0 | 80 | 9.2 | 10.27 | 0.000000112 |
| ZBED1 | 0 | 694 | 78.1 | 6.1 | 0.000000121 |
| CDC37 | 108.42 | 378 | 44.4 | 5.25 | 0.000000134 |
| TPI1 | 1124.38 | 249 | 26.7 | 6.9 | 0.000000135 |
| HSPA4 | 966.25 | 840 | 94.3 | 5.19 | 0.000000138 |
| ANP32A | 258.9 | 249 | 28.6 | 4.09 | 0.000000142 |
| ILF2 | 202.42 | 390 | 43 | 5.26 | 0.000000151 |
| PTGES3 | 248.79 | 139 | 16.5 | 4.81 | 0.000000166 |
| MRPS34 | 28.45 | 218 | 25.6 | 9.98 | 0.000000206 |
| SSBP1 | 81.35 | 148 | 17.2 | 9.6 | 0.000000215 |
| MARK3 | 2.07 | 659 | 74.1 | 9.85 | 0.00000024 |
| NDUFA8 | 38.87 | 172 | 20.1 | 7.65 | 0.000000257 |
| SSB | 372.36 | 408 | 46.8 | 7.12 | 0.000000269 |
| ALDH3A1 | 903.32 | 453 | 50.4 | 6.54 | 0.000000277 |
| TUFM | 844.58 | 452 | 49.5 | 7.61 | 0.000000322 |
| RPS11 | 182.73 | 158 | 18.4 | 10.3 | 0.000000336 |
| MSN | 1360.25 | 577 | 67.8 | 6.4 | 0.000000338 |
| SOD1 | 226.85 | 154 | 15.9 | 6.13 | 0.000000362 |
| BIN1 | 4.6 | 409 | 45.5 | 5.49 | 0.000000391 |
| ARF3 | 491.41 | 181 | 20.6 | 7.43 | 0.000000441 |
| MANF | 70.6 | 182 | 20.7 | 8.69 | 0.000000455 |
| TUBAL3 | 256.25 | 406 | 45.5 | 6.32 | 0.000000463 |
| BCAP31 | 182.75 | 246 | 28 | 8.44 | 0.000000495 |
| VDAC2 | 439.09 | 283 | 30.4 | 7.2 | 0.000000515 |
| ACLY | 966.45 | 1101 | 120.8 | 7.33 | 0.000000529 |
| CRIP1 | 65.23 | 77 | 8.5 | 8.75 | 0.000000545 |
| AGTRAP | 5.23 | 152 | 16.7 | 6.28 | 0.000000545 |
| PTMS | 98.42 | 102 | 11.5 | 4.16 | 0.000000605 |
| PARG | 3.08 | 894 | 102.2 | 5.83 | 0.000000612 |
| HNRNPA1 | 1559.76 | 320 | 34.2 | 9.23 | 0.000000726 |
| YWHAQ | 924.36 | 245 | 27.7 | 4.78 | 0.000000731 |
| HNRNPA3 | 729.88 | 378 | 39.6 | 9.01 | 0.000000778 |
| NUDT1 | 3.94 | 156 | 17.9 | 5.08 | 0.000000827 |
| EIF4H | 195.82 | 248 | 27.4 | 7.23 | 0.000000867 |
| MARCKS | 31.39 | 332 | 31.5 | 4.45 | 0.000000887 |
| CACYBP | 208.21 | 228 | 26.2 | 8.25 | 0.000000907 |
| PRDM15 | 0 | 1507 | 169.2 | 8.24 | 0.000000984 |
| RARS | 402.8 | 660 | 75.3 | 6.68 | 0.00000101 |
| CISD1 | 33.14 | 108 | 12.2 | 9.09 | 0.00000102 |
| KAT7 | 2.58 | 501 | 58.1 | 9.01 | 0.00000103 |
| BTAF1 | 2.71 | 1849 | 206.8 | 6.52 | 0.00000112 |
| AP4B1 | 2.62 | 302 | 34.4 | 7.55 | 0.00000114 |
| PPP2R1A | 261.73 | 589 | 65.3 | 5.11 | 0.00000115 |
| FUS | 287.97 | 525 | 53.3 | 9.36 | 0.0000012 |
| PSPH | 2.56 | 225 | 25 | 5.69 | 0.00000124 |
| PPP2R1B | 75.14 | 601 | 66.2 | 4.94 | 0.00000129 |
| H2AFZ | 350.45 | 128 | 13.5 | 10.58 | 0.00000131 |
| TP53RK | 0 | 253 | 28.1 | 9.54 | 0.00000138 |
| NME1 | 651.1 | 152 | 17.1 | 6.19 | 0.00000145 |
| GFPT1 | 248.93 | 681 | 76.7 | 6.84 | 0.00000154 |
| MTRR | 3.12 | 698 | 77.6 | 6.49 | 0.00000157 |
| EIF5 | 203.07 | 431 | 49.2 | 5.58 | 0.00000158 |
| CYB5B | 32.85 | 146 | 16.3 | 4.97 | 0.0000016 |
| RPF2 | 8.47 | 306 | 35.6 | 9.99 | 0.0000016 |
| PLEKHG3 | 0 | 1163 | 128.2 | 6.76 | 0.00000197 |
| PARK7 | 406.71 | 189 | 19.9 | 6.79 | 0.00000199 |
| PARP4 | 3.05 | 1724 | 192.5 | 5.66 | 0.000002 |
| SSR3 | 24.23 | 185 | 21.1 | 9.61 | 0.00000201 |
| ARFGAP2 | 5.29 | 414 | 44.5 | 8.4 | 0.00000208 |
| GDA | 535.91 | 454 | 51 | 5.68 | 0.0000022 |
| VPS53 | 4.86 | 673 | 76.5 | 5.78 | 0.00000227 |
| PSMC6 | 257.98 | 389 | 44.1 | 7.49 | 0.00000231 |
| RPL27A | 171.34 | 148 | 16.6 | 11 | 0.00000234 |
| SUGP2 | 2.72 | 1030 | 115.1 | 8.63 | 0.00000245 |
| PSMA7 | 270.6 | 248 | 27.9 | 8.46 | 0.00000273 |
| NME2 | 871.97 | 267 | 30.1 | 8.92 | 0.00000304 |
| YTHDC2 | 5.2 | 1430 | 160.1 | 8.4 | 0.0000031 |
| RANGRF | 2.37 | 146 | 16.1 | 5.25 | 0.00000319 |
| PARP1 | 371.04 | 1014 | 113 | 8.88 | 0.00000351 |
| OSBPL9 | 2.44 | 719 | 81.1 | 5.82 | 0.00000402 |
| UBA1 | 987.5 | 1018 | 113.7 | 5.6 | 0.00000403 |
| GSTK1 | 26.87 | 226 | 25.5 | 8.41 | 0.00000478 |
| RRBP1 | 332.93 | 1410 | 152.4 | 8.6 | 0.00000483 |
| UBA52 | 384.54 | 128 | 14.7 | 9.83 | 0.00000485 |
| UNC45A | 41.52 | 929 | 101.6 | 6.06 | 0.00000552 |
| PINX1 | 2.67 | 328 | 37 | 9.6 | 0.00000568 |
| TIMM21 | 2.81 | 248 | 28.2 | 9.7 | 0.00000585 |
| CYB5R1 | 12.65 | 305 | 34.1 | 9.38 | 0.00000587 |
| DDX28 | 2.27 | 540 | 59.5 | 10.42 | 0.00000604 |
| LUZP1 | 2.46 | 1026 | 114.5 | 8.88 | 0.00000607 |
| CCT8 | 669.18 | 548 | 59.6 | 5.6 | 0.00000611 |
| CDC42EP4 | 6.05 | 356 | 38 | 5.19 | 0.00000644 |
| SLC25A3 | 266.18 | 361 | 39.9 | 9.36 | 0.0000067 |
| CASP6 | 3.9 | 204 | 22.6 | 7.15 | 0.00000674 |
| TTI1 | 9.05 | 1089 | 122 | 5.97 | 0.00000686 |
| MRPL10 | 4.27 | 261 | 29.3 | 9.58 | 0.00000708 |
| HNRNPU | 1029.09 | 825 | 90.5 | 6 | 0.00000731 |
| EIF1 | 89.82 | 113 | 12.7 | 7.44 | 0.00000748 |
| ZNF572 | 0 | 529 | 61.2 | 8 | 0.00000753 |
| LGMN | 11.4 | 372 | 42 | 6.2 | 0.00000796 |
| OGDH | 210.99 | 1019 | 115.4 | 7.02 | 0.00000805 |
| MAPK3 | 13.7 | 335 | 38.3 | 6.2 | 0.00000817 |
| APPL1 | 7.65 | 709 | 79.6 | 5.41 | 0.00000902 |
| RPL22 | 127.85 | 128 | 14.8 | 9.19 | 0.00000912 |
| BUD23 | 13.64 | 281 | 31.9 | 8.73 | 0.00000922 |
| SLC38A5 | 3.44 | 421 | 45.9 | 8.34 | 0.00000922 |
| RBBP7 | 158.89 | 425 | 47.8 | 5.05 | 0.00000943 |
| PRIM2 | 3.13 | 509 | 58.8 | 7.91 | 0.0000101 |
| RAB5C | 226.64 | 216 | 23.5 | 8.41 | 0.0000105 |
| NT5C3B | 3.01 | 292 | 33.5 | 6.68 | 0.0000106 |
| QTRT1 | 4.94 | 403 | 44 | 7.23 | 0.000011 |
| DDT | 38.08 | 118 | 12.7 | 7.3 | 0.000012 |
| ABHD14B | 41.61 | 210 | 22.3 | 6.4 | 0.0000122 |
| TXN | 843.78 | 105 | 11.7 | 4.92 | 0.0000124 |
| GINS3 | 2.38 | 138 | 15.6 | 4.93 | 0.0000124 |
| ATAD1 | 48.76 | 361 | 40.7 | 6.9 | 0.0000131 |
| MRPS7 | 30.11 | 242 | 28.1 | 9.99 | 0.0000132 |
| CAPRIN1 | 165.84 | 694 | 76.8 | 5.12 | 0.0000147 |
| ACOX1 | 8.44 | 622 | 70.1 | 7.61 | 0.0000148 |
| COMMD2 | 2.43 | 169 | 19.1 | 6.28 | 0.0000148 |
| UQCRFS1 | 68.52 | 274 | 29.6 | 8.32 | 0.0000153 |
| MDH2 | 1104.57 | 338 | 35.5 | 8.68 | 0.0000156 |
| MRPL41 | 20.07 | 137 | 15.4 | 9.57 | 0.0000156 |
| DOHH | 4.09 | 302 | 32.9 | 4.83 | 0.0000162 |
| SBDS | 77.82 | 250 | 28.7 | 8.75 | 0.0000162 |
| HYPK | 27.9 | 129 | 14.7 | 4.93 | 0.0000167 |
| HPRT1 | 161.62 | 218 | 24.6 | 6.68 | 0.0000169 |
| MVK | 2.46 | 396 | 42.4 | 6.46 | 0.000017 |
| EPHX1 | 428.26 | 455 | 52.9 | 7.25 | 0.0000177 |
| TFRC | 487.09 | 760 | 84.8 | 6.61 | 0.0000182 |
| RPS2 | 413.31 | 293 | 31.3 | 10.24 | 0.0000182 |
| POP1 | 2.9 | 1024 | 114.6 | 9.22 | 0.0000185 |
| NDUFB1 | 3.2 | 58 | 7 | 8.92 | 0.0000186 |
| UEVLD | 0 | 215 | 24.2 | 7.88 | 0.0000195 |
| NEU1 | 0 | 415 | 45.4 | 5.88 | 0.0000196 |
| AFG1L | 2.1 | 481 | 54.8 | 7.27 | 0.00002 |
| CDC16 | 2.24 | 568 | 65.8 | 6.07 | 0.0000201 |
| CCT2 | 716.92 | 535 | 57.5 | 6.46 | 0.0000203 |
| NDUFAF2 | 31.41 | 169 | 19.8 | 8.97 | 0.0000207 |
| COX7A2 | 25.37 | 83 | 9.4 | 9.76 | 0.0000211 |
| RPS29 | 25.21 | 56 | 6.7 | 10.13 | 0.0000213 |
| AP2B1 | 164.5 | 937 | 104.5 | 5.38 | 0.0000215 |
| AVL9 | 3.64 | 591 | 65.3 | 6 | 0.0000226 |
| EIF3F | 148.26 | 357 | 37.5 | 5.45 | 0.0000228 |
| IQGAP1 | 680.17 | 1657 | 189.1 | 6.48 | 0.000023 |
| GOLT1B | 36.48 | 138 | 15.4 | 10.36 | 0.0000233 |
| EIF5B | 167.31 | 1220 | 138.7 | 5.49 | 0.000024 |
| DAZAP1 | 78.29 | 378 | 40.5 | 8.28 | 0.000024 |
| PC | 45.35 | 1178 | 129.6 | 6.84 | 0.0000243 |
| ARFIP1 | 8.67 | 341 | 38.6 | 6.55 | 0.0000246 |
| ABCC4 | 2.08 | 1278 | 144.1 | 8.19 | 0.0000255 |
| TEAD1 | 3.56 | 357 | 40 | 8.32 | 0.0000256 |
| HMG20A | 8.36 | 347 | 40.1 | 6.49 | 0.0000257 |
| TMSB10 | 82.26 | 44 | 5 | 5.36 | 0.0000262 |
| ST13 | 195.9 | 369 | 41.3 | 5.27 | 0.0000275 |
| RPL34 | 57.39 | 117 | 13.3 | 11.47 | 0.0000283 |
| RBM8A | 43.26 | 174 | 19.9 | 5.72 | 0.0000285 |
| BRIX1 | 53.87 | 353 | 41.4 | 9.92 | 0.000029 |
| AKAP9 | 2.23 | 1643 | 191.2 | 4.89 | 0.0000292 |
| TAGLN2 | 585.76 | 199 | 22.4 | 8.25 | 0.0000296 |
| CLTC | 1849.84 | 1639 | 187.8 | 5.69 | 0.0000296 |
| GNPAT | 2.73 | 619 | 69.9 | 6.4 | 0.0000311 |
| STIP1 | 598.42 | 543 | 62.6 | 6.8 | 0.0000321 |
| NIT1 | 5.22 | 291 | 31.8 | 6.87 | 0.0000323 |
| ZNF207 | 31.59 | 463 | 49.7 | 9.1 | 0.0000325 |
| HYKK | 0 | 220 | 24.9 | 8.97 | 0.0000336 |
| MRPL57 | 0 | 102 | 12.3 | 11.44 | 0.0000397 |
| HNRNPR | 347.06 | 633 | 70.9 | 8.13 | 0.0000399 |
| MED15 | 18.88 | 677 | 75.9 | 9.29 | 0.0000404 |
| UBR5 | 18.45 | 2798 | 309 | 5.85 | 0.0000411 |
| NUBP1 | 19.41 | 309 | 33.4 | 5.24 | 0.0000422 |
| GANAB | 434.95 | 944 | 106.8 | 6.14 | 0.0000424 |
| GPATCH8 | 7.84 | 1424 | 155.1 | 8.82 | 0.0000435 |
| USP3 | 7.26 | 476 | 53.7 | 8.1 | 0.0000439 |
| POTEI | 896.73 | 1075 | 121.2 | 6.21 | 0.0000453 |
| RAPH1 | 27.09 | 1250 | 135.2 | 8.85 | 0.0000473 |
| ADD2 | 3.2 | 559 | 62.4 | 6.49 | 0.0000478 |
| ADSS | 185.04 | 456 | 50.1 | 6.55 | 0.0000491 |
| MRPS21 | 0 | 87 | 10.7 | 10.21 | 0.0000499 |
| PRMT1 | 181.33 | 343 | 39.6 | 5.6 | 0.0000501 |
| CAP1 | 181.55 | 474 | 51.8 | 8.06 | 0.0000511 |
| NAP1L1 | 328.24 | 368 | 42.7 | 4.55 | 0.0000517 |
| PAWR | 8.19 | 340 | 36.5 | 5.41 | 0.000052 |
| TMEM33 | 123.84 | 247 | 28 | 9.7 | 0.0000573 |
| GLRX | 118.55 | 106 | 11.8 | 8.09 | 0.000063 |
| RP2 | 5.48 | 350 | 39.6 | 5.12 | 0.0000641 |
| EIF1AY | 97.73 | 144 | 16.4 | 5.24 | 0.0000645 |
| SLC25A6 | 954.66 | 298 | 32.8 | 9.74 | 0.0000656 |
| SRPRB | 75.89 | 271 | 29.7 | 9.04 | 0.0000663 |
| RRP7A | 2.72 | 280 | 32.3 | 9.58 | 0.000067 |
| LRP8 | 3.56 | 700 | 77.8 | 5.01 | 0.0000681 |
| SLC39A14 | 11.94 | 481 | 52.8 | 5.77 | 0.000069 |
| RPS27L | 62.14 | 84 | 9.5 | 9.45 | 0.00007 |
| SLAMF1 | 0 | 298 | 33.3 | 8.85 | 0.0000722 |
| CSTB | 44.1 | 98 | 11.1 | 7.56 | 0.000074 |
| DPCD | 2.21 | 203 | 23.2 | 9.03 | 0.0000741 |
| CLU | 12.49 | 416 | 48.8 | 6.71 | 0.0000748 |
| DARS | 241.84 | 501 | 57.1 | 6.55 | 0.0000757 |
| ACTR3 | 196.6 | 418 | 47.3 | 5.88 | 0.000076 |
| ARHGAP35 | 0 | 1499 | 170.4 | 6.64 | 0.0000764 |
| RAB1A | 438.95 | 205 | 22.7 | 6.21 | 0.0000804 |
| KIDINS220 | 0 | 1031 | 115.2 | 7.74 | 0.0000811 |
| BAZ2A | 3.48 | 1878 | 208.3 | 6.6 | 0.0000845 |
| PEX3 | 6.49 | 373 | 42.1 | 8.15 | 0.0000891 |
| MYDGF | 26.68 | 173 | 18.8 | 6.68 | 0.0000903 |
| CYB5R3 | 65.61 | 279 | 31.7 | 7.58 | 0.0000909 |
| VPS28 | 8.14 | 221 | 25.4 | 5.54 | 0.0000914 |
| PAK4 | 2.98 | 426 | 47.9 | 9.47 | 0.000094 |
| GOLGA4 | 7.81 | 2223 | 260.2 | 5.4 | 0.0000941 |
| TSPAN13 | 6.7 | 204 | 22.1 | 7.93 | 0.0000959 |
| POTEF | 1486.85 | 1075 | 121.4 | 6.2 | 0.0000982 |
| RANBP1 | 166.58 | 200 | 23.2 | 5.29 | 0.000105276 |
| RTCA | 3.42 | 366 | 39.3 | 7.85 | 0.000108122 |
| RAB18 | 72.7 | 206 | 23 | 5.24 | 0.000108879 |
| RBBP5 | 6.83 | 500 | 55 | 4.96 | 0.000109045 |
| ZNF330 | 0 | 320 | 36.2 | 6.16 | 0.000111055 |
| MSTO1 | 2.36 | 99 | 10.9 | 5.8 | 0.00011142 |
| PNMA1 | 2.27 | 353 | 39.7 | 4.83 | 0.000111754 |
| HSBP1 | 2.57 | 76 | 8.5 | 4.36 | 0.00011504 |
| RAB31 | 4.91 | 194 | 21.6 | 7.06 | 0.00011766 |
| APEX1 | 196.77 | 318 | 35.5 | 8.12 | 0.000117989 |
| AHSA1 | 204.48 | 338 | 38.3 | 5.53 | 0.000118753 |
| DCTN4 | 14.79 | 460 | 52.3 | 7.34 | 0.000122235 |
| HARS2 | 95.55 | 481 | 54.1 | 8.02 | 0.000124876 |
| DYNLL1 | 70.14 | 89 | 10.4 | 7.4 | 0.000126309 |
| DDX20 | 0 | 824 | 92.2 | 6.95 | 0.000131741 |
| UTP23 | 7.32 | 145 | 16.7 | 9.04 | 0.000131771 |
| HMGA1 | 39.3 | 107 | 11.7 | 10.32 | 0.000133108 |
| PRKD3 | 3.86 | 890 | 100.4 | 6.87 | 0.000139444 |
| PCYOX1L | 2.12 | 494 | 54.6 | 7.31 | 0.000140952 |
| ERO1A | 53.9 | 468 | 54.4 | 5.68 | 0.000142925 |
| HSPB1 | 272.13 | 205 | 22.8 | 6.4 | 0.000144269 |
| EEF1G | 518.06 | 437 | 50.1 | 6.67 | 0.000145551 |
| DVL2 | 2.04 | 736 | 78.9 | 6.02 | 0.000153285 |
| SURF2 | 0 | 256 | 29.6 | 9.22 | 0.000155841 |
| TUBA4A | 2521.7 | 433 | 48.3 | 5.01 | 0.000160292 |
| SFN | 633.24 | 216 | 24.3 | 4.82 | 0.000161992 |
| CHRAC1 | 2.06 | 131 | 14.7 | 5.1 | 0.000162158 |
| C8orf33 | 4.74 | 229 | 25 | 9.95 | 0.000162651 |
| MED8 | 2.94 | 179 | 19 | 9.09 | 0.000169465 |
| IGBP1 | 14.96 | 339 | 39.2 | 5.38 | 0.000178099 |
| FBL | 120.01 | 321 | 33.8 | 10.18 | 0.000180116 |
| DNAJA1 | 129.46 | 397 | 44.8 | 7.08 | 0.000180888 |
| PAK1 | 8.59 | 545 | 60.6 | 5.76 | 0.000196664 |
| MLLT1 | 2.22 | 559 | 62 | 8.59 | 0.000203129 |
| AHCY | 537.55 | 432 | 47.7 | 6.34 | 0.000212034 |
| PDE3B | 0 | 1061 | 118.7 | 5.8 | 0.000219284 |
| HINT3 | 0 | 182 | 20.3 | 6.6 | 0.000220785 |
| LCLAT1 | 9.4 | 376 | 44.5 | 8.59 | 0.000224079 |
| DDX17 | 417.94 | 650 | 72.3 | 8.59 | 0.000225738 |
| PSMA5 | 287.38 | 241 | 26.4 | 4.79 | 0.00022776 |
| CBX3 | 208.21 | 183 | 20.8 | 5.33 | 0.000230027 |
| XRCC4 | 9.88 | 310 | 35.3 | 5.25 | 0.00023003 |
| HMBS | 13.7 | 344 | 37.7 | 7.39 | 0.000234073 |
| C11orf54 | 3.08 | 204 | 23.2 | 6.79 | 0.00024384 |
| CANT1 | 0 | 350 | 38.9 | 5.33 | 0.000243903 |
| PAIP2 | 4.06 | 127 | 15 | 4.12 | 0.000244378 |
| COA7 | 2.67 | 231 | 25.7 | 6.02 | 0.000245469 |
| RPL7L1 | 3.56 | 153 | 18 | 11.06 | 0.000246733 |
| TMEM56 | 0 | 263 | 30 | 9.33 | 0.000253755 |
| MT-ND2 | 8.57 | 347 | 38.9 | 9.83 | 0.000257431 |
| NARS | 251.17 | 548 | 62.9 | 6.25 | 0.00026085 |
| UBE2O | 47.93 | 1292 | 141.2 | 5.12 | 0.000264126 |
| RDX | 625.35 | 583 | 68.5 | 6.37 | 0.000265553 |
| ZC3HAV1L | 7.16 | 300 | 32.9 | 8.13 | 0.000282692 |
| THOC2 | 37.28 | 1593 | 182.7 | 8.44 | 0.000285695 |
| VPS13B | 0 | 1427 | 159.5 | 5.68 | 0.000286619 |
| HMGN5 | 13.85 | 282 | 31.5 | 4.55 | 0.000296049 |
| RBM17 | 11.01 | 401 | 44.9 | 5.97 | 0.000298993 |
| PTCD1 | 2.3 | 700 | 78.8 | 8.59 | 0.000303539 |
| FAM210B | 6.13 | 192 | 20.4 | 10.43 | 0.000306085 |
| VDAC1 | 666.13 | 283 | 30.8 | 8.54 | 0.000309823 |
| C16orf62 | 0 | 813 | 93 | 6.83 | 0.000310966 |
| CENPX | 2.5 | 81 | 9 | 5.9 | 0.000321457 |
| MAN2B1 | 4.08 | 1010 | 113.5 | 7.28 | 0.000322952 |
| WDR62 | 3.3 | 1518 | 165.8 | 5.91 | 0.000323482 |
| PTBP1 | 340.73 | 531 | 57.2 | 9.17 | 0.000325378 |
| FAM32A | 2.1 | 92 | 11 | 10.11 | 0.000325479 |
| METTL13 | 3.22 | 396 | 44.5 | 5.63 | 0.000339513 |
| ISOC1 | 11.59 | 298 | 32.2 | 7.39 | 0.000345032 |
| MFGE8 | 4.17 | 312 | 35.2 | 8.85 | 0.000346013 |
| ACTR2 | 149.95 | 394 | 44.7 | 6.74 | 0.000349452 |
| TRIM28 | 406.74 | 835 | 88.5 | 5.77 | 0.000358541 |
| NLN | 83.37 | 704 | 80.6 | 6.64 | 0.000362503 |
| FAM83E | 0 | 478 | 51.7 | 9.35 | 0.000377633 |
| MTDH | 78.76 | 582 | 63.8 | 9.32 | 0.000400202 |
| PSMD3 | 178.12 | 534 | 60.9 | 8.44 | 0.000404396 |
| FAM45A | 6.11 | 349 | 39.8 | 6.8 | 0.000415736 |
| TSN | 97.63 | 228 | 26.2 | 6.44 | 0.00041983 |
| S100A4 | 295.57 | 101 | 11.7 | 6.11 | 0.000424791 |
| MVB12A | 7.3 | 233 | 24.5 | 8.31 | 0.000426418 |
| RCN1 | 87.57 | 331 | 38.9 | 5 | 0.000429114 |
| INTS5 | 3.47 | 1019 | 107.9 | 7.05 | 0.000434308 |
| P4HA1 | 157.4 | 534 | 60.9 | 6.01 | 0.000447077 |
| CXorf38 | 2.74 | 200 | 23.3 | 4.77 | 0.000448136 |
| USP6 | 2.46 | 1089 | 121.9 | 7.46 | 0.000450823 |
| GALNT14 | 2.11 | 519 | 60.5 | 7.4 | 0.000465665 |
| SPRYD7 | 2.45 | 196 | 21.7 | 6.7 | 0.000472036 |
| MTPN | 51.38 | 118 | 12.9 | 5.52 | 0.000476544 |
| NHEJ1 | 4.05 | 299 | 33.3 | 5.97 | 0.000487119 |
| PLEKHA5 | 6.22 | 1060 | 121.2 | 8.07 | 0.000488761 |
| ADPRHL2 | 0 | 363 | 38.9 | 5.07 | 0.000505431 |
| CAMK1 | 5.33 | 370 | 41.3 | 5.29 | 0.000517612 |
| SAAL1 | 0 | 474 | 53.5 | 4.5 | 0.000526474 |
| PISD | 0 | 375 | 43 | 9.7 | 0.000538991 |
| RCN2 | 35.63 | 317 | 36.9 | 4.4 | 0.000538994 |
| RAN | 634.68 | 216 | 24.4 | 7.49 | 0.000539801 |
| HNRNPA2B1 | 1748.57 | 353 | 37.4 | 8.95 | 0.000563828 |
| TSEN15 | 3.27 | 171 | 18.6 | 4.58 | 0.000576804 |
| SRP72 | 109.41 | 671 | 74.6 | 9.26 | 0.000580185 |
| SYAP1 | 8.63 | 352 | 39.9 | 4.53 | 0.000580875 |
| UQCRH | 56.68 | 91 | 10.7 | 4.44 | 0.000584004 |
| ATXN2 | 22.06 | 995 | 106 | 9.67 | 0.00058983 |
| CCDC51 | 2.34 | 302 | 33.6 | 6.96 | 0.000604198 |
| DNAJC19 | 1.87 | 91 | 10.1 | 10.05 | 0.000645298 |
| MAFF | 2.14 | 135 | 14.6 | 10.15 | 0.000646637 |
| DIEXF | 9.9 | 756 | 87 | 5.88 | 0.000649016 |
| NAXE | 149.38 | 288 | 31.7 | 7.66 | 0.000649676 |
| HNRNPL | 362.01 | 589 | 64.1 | 8.22 | 0.000650689 |
| EIF2B4 | 13.77 | 523 | 57.5 | 9.38 | 0.000651338 |
| PPP4R3A | 21.06 | 820 | 93.8 | 4.89 | 0.000651468 |
| RSL24D1 | 8.31 | 163 | 19.6 | 9.98 | 0.000656301 |
| OPA3 | 2.3 | 179 | 20 | 8.91 | 0.000666455 |
| ANP32E | 116.8 | 268 | 30.7 | 3.85 | 0.00066703 |
| STIM1 | 2.35 | 540 | 62.1 | 6.48 | 0.000691932 |
| LTA4H | 246.8 | 611 | 69.2 | 6.18 | 0.000699019 |
| RXRA | 3.33 | 365 | 41 | 7.77 | 0.000704634 |
| CDC42 | 162.55 | 191 | 21.2 | 6.55 | 0.000706883 |
| FUBP1 | 384.18 | 644 | 67.5 | 7.61 | 0.000711755 |
| MRPL14 | 3.47 | 145 | 15.9 | 10.24 | 0.000717462 |
| SCD | 12.14 | 359 | 41.5 | 9 | 0.000743507 |
| CAPN1 | 50.45 | 714 | 81.8 | 5.67 | 0.000751095 |
| LDHA | 853.73 | 332 | 36.7 | 8.27 | 0.000779695 |
| MARS | 151.98 | 900 | 101.1 | 6.16 | 0.00078352 |
| G3BP1 | 197.22 | 466 | 52.1 | 5.52 | 0.000789248 |
| ALB | 218.17 | 609 | 69.3 | 6.28 | 0.000791357 |
| SLC47A1 | 0 | 570 | 61.9 | 7.58 | 0.000805728 |
| RPS3 | 978.75 | 243 | 26.7 | 9.66 | 0.000808465 |
| ARFGAP3 | 2.92 | 472 | 52 | 6.11 | 0.000823857 |
| GTPBP6 | 5.37 | 516 | 56.8 | 9.42 | 0.000833771 |
| RPL19 | 116.76 | 196 | 23.5 | 11.47 | 0.000837704 |
| GXYLT1 | 5.58 | 409 | 46.8 | 8.95 | 0.000840987 |
| EIF2A | 48.14 | 560 | 62.2 | 8.91 | 0.000848605 |
| RPL8 | 199.37 | 257 | 28 | 11.03 | 0.00086467 |
| RPL17 | 246.28 | 184 | 21.4 | 10.17 | 0.000878111 |
| RPS10 | 394.6 | 165 | 18.9 | 10.15 | 0.000885261 |
| SLC12A4 | 5.63 | 1054 | 117 | 6.8 | 0.000891319 |
| EXOSC4 | 12.85 | 245 | 26.4 | 6.52 | 0.000894026 |
| MYD88 | 2.3 | 146 | 15.8 | 8.24 | 0.000900876 |
| TMEM165 | 0 | 261 | 28.4 | 6.54 | 0.000902126 |
| ENOPH1 | 40.52 | 261 | 28.9 | 4.78 | 0.000910397 |
| UBP1 | 7 | 504 | 56.4 | 6.29 | 0.000916324 |
| SF3B1 | 213.62 | 1304 | 145.7 | 7.09 | 0.000918459 |
| LRRFIP1 | 120.83 | 752 | 82.6 | 4.61 | 0.000929573 |
| DLG2 | 11.52 | 334 | 38.4 | 6.81 | 0.000935968 |
| NDUFB6 | 19.2 | 128 | 15.5 | 9.63 | 0.00094418 |
| RMDN1 | 5.09 | 284 | 32.3 | 8.94 | 0.000945582 |
| LGALS1 | 570.76 | 135 | 14.7 | 5.5 | 0.000951899 |
| PYGL | 403.37 | 813 | 93.1 | 7.3 | 0.000959477 |
| RHOA | 234.25 | 193 | 21.8 | 6.1 | 0.000967839 |
| SLC52A2 | 2.03 | 445 | 45.7 | 7.15 | 0.00097159 |
| TPM4 | 652.33 | 248 | 28.5 | 4.69 | 0.000981086 |
| CD14 | 0 | 375 | 40.1 | 6.23 | 0.000983474 |
| SPC25 | 10.44 | 224 | 26.1 | 8 | 0.000991021 |
| ASF1A | 5.31 | 204 | 23 | 4.41 | 0.000991036 |
| UBAC2 | 4.28 | 157 | 18 | 8.03 | 0.000993352 |
| NCBP1 | 101.98 | 790 | 91.8 | 6.43 | 0.001004276 |
| FKBP11 | 32.39 | 146 | 15.8 | 6.16 | 0.001005074 |
| RNH1 | 275.2 | 461 | 49.9 | 4.82 | 0.001011054 |
| STK38 | 0 | 465 | 54.2 | 7.15 | 0.001024205 |
| MANBAL | 2.74 | 85 | 9.5 | 9.16 | 0.001028414 |
| ETFA | 252.96 | 333 | 35.1 | 8.38 | 0.001036133 |
| KIF3C | 10.78 | 793 | 89.4 | 8.22 | 0.001039488 |
| GDAP2 | 2.03 | 496 | 56 | 6.16 | 0.001047695 |
| PPCS | 2.71 | 138 | 15.6 | 5.88 | 0.001062997 |
| TUBB4B | 3754.85 | 445 | 49.8 | 4.89 | 0.001087241 |
| POLR2M | 0 | 363 | 40.7 | 8.38 | 0.00108907 |
| EIF4G1 | 377.25 | 1559 | 171.4 | 5.31 | 0.001103832 |
| UBE2L3 | 86.3 | 154 | 17.9 | 8.51 | 0.001111053 |
| HNRNPDL | 296.39 | 244 | 27.2 | 8.65 | 0.001117442 |
| APRT | 92.24 | 180 | 19.6 | 6.02 | 0.001149148 |
| DNAJC3 | 6.12 | 504 | 57.5 | 6.15 | 0.001152177 |
| KDSR | 2.66 | 268 | 29.1 | 5.33 | 0.001155082 |
| TRIO | 5.1 | 2563 | 287.2 | 6.34 | 0.001160912 |
| SPAG7 | 2.33 | 227 | 26 | 7.91 | 0.001161135 |
| GCLM | 116.45 | 274 | 30.7 | 6.02 | 0.001180877 |
| SNAPIN | 2.92 | 136 | 14.9 | 9.31 | 0.001181604 |
| RPS19 | 474.22 | 145 | 16.1 | 10.32 | 0.0011874 |
| STEAP3 | 2.66 | 456 | 50.5 | 7.87 | 0.001194373 |
| GNS | 15.92 | 532 | 59.9 | 8.12 | 0.001205436 |
| RPS26 | 83.63 | 115 | 13 | 11 | 0.001219739 |
| NOP56 | 113.41 | 594 | 66 | 9.19 | 0.00122954 |
| SELENOI | 5.08 | 397 | 45.2 | 6.6 | 0.001234197 |
| RAB34 | 6.34 | 198 | 21.1 | 12.16 | 0.001251442 |
| MRPS33 | 2.98 | 106 | 12.6 | 10.11 | 0.001276607 |
| STRBP | 81.07 | 658 | 71.9 | 8.65 | 0.001300327 |
| NPM3 | 66.15 | 178 | 19.3 | 4.63 | 0.001309595 |
| SCML2 | 2.3 | 700 | 77.2 | 8.54 | 0.001318448 |
| SLC22A18 | 2.69 | 424 | 44.8 | 9.57 | 0.001320417 |
| UCHL1 | 687.51 | 223 | 24.8 | 5.48 | 0.001347109 |
| SLC30A7 | 2.45 | 376 | 41.6 | 6.95 | 0.001347473 |
| SRGAP3 | 0 | 1075 | 121.7 | 6.58 | 0.001363638 |
| RNF25 | 4.28 | 459 | 51.2 | 6.54 | 0.00136546 |
| ASAH1 | 3.23 | 389 | 44 | 7.62 | 0.001383914 |
| DR1 | 4 | 176 | 19.4 | 4.75 | 0.001416533 |
| NOLC1 | 301.09 | 700 | 73.7 | 9.47 | 0.001423515 |
| CKS1B | 3.95 | 79 | 9.7 | 8.94 | 0.001436723 |
| NGDN | 10.42 | 311 | 35.2 | 9.33 | 0.001443129 |
| ITGB5 | 39.39 | 799 | 88 | 6.06 | 0.001463641 |
| MTFR1L | 3.02 | 280 | 30.8 | 6.32 | 0.001465963 |
| PGR | 5.74 | 933 | 99 | 6.09 | 0.00146987 |
| TXN2 | 21.04 | 166 | 18.4 | 8.29 | 0.001470948 |
| LYAR | 3.03 | 379 | 43.6 | 9.54 | 0.001478507 |
| PCYT2 | 0 | 311 | 35.2 | 6.47 | 0.001482154 |
| UNC93B1 | 2.48 | 597 | 66.6 | 6.96 | 0.00148327 |
| RNF40 | 18.09 | 838 | 94.7 | 5.58 | 0.001488711 |
| NBAS | 2.12 | 2251 | 254.7 | 5.94 | 0.001492503 |
| SPCS1 | 3.39 | 102 | 11.8 | 9.31 | 0.001494395 |
| VBP1 | 80.1 | 197 | 22.6 | 7.11 | 0.001578124 |
| RFX5 | 0 | 576 | 60.5 | 9.57 | 0.001642134 |
| RTN1 | 139.99 | 776 | 83.6 | 4.69 | 0.001644643 |
| EPB41L3 | 458.51 | 865 | 96.5 | 5.43 | 0.001650984 |
| RPS6 | 347.54 | 249 | 28.7 | 10.84 | 0.001673788 |
| KIRREL1 | 4.83 | 757 | 83.5 | 5.73 | 0.001678126 |
| GRB2 | 57.17 | 217 | 25.2 | 6.32 | 0.001685821 |
| CPT2 | 9.76 | 658 | 73.7 | 8.18 | 0.001712169 |
| CSNK1G1 | 2.72 | 393 | 45.4 | 9.16 | 0.001732863 |
| PHF6 | 13.34 | 310 | 35.2 | 9.04 | 0.001736434 |
| COX7C | 62.97 | 63 | 7.2 | 10.27 | 0.001740688 |
| ZC3HC1 | 8.93 | 431 | 47.7 | 5.19 | 0.001759254 |
| AHCYL2 | 46.31 | 508 | 56.7 | 8.09 | 0.001763465 |
| SNRPB | 151.84 | 231 | 23.6 | 10.9 | 0.001770673 |
| FSTL1 | 6.43 | 273 | 31.2 | 5.59 | 0.001793443 |
| POLR3E | 2.41 | 672 | 75.7 | 6.42 | 0.001808832 |
| FKBP4 | 245.52 | 459 | 51.8 | 5.43 | 0.001814211 |
| CMPK1 | 49.67 | 196 | 22.2 | 5.57 | 0.001815003 |
| COPZ1 | 34.95 | 177 | 20.2 | 4.81 | 0.001824174 |
| DNAJC10 | 37.89 | 747 | 86.1 | 7.39 | 0.001831552 |
| LUC7L3 | 20.94 | 432 | 51.4 | 9.79 | 0.001837753 |
| ARHGAP17 | 3.35 | 226 | 25.7 | 7.15 | 0.00183856 |
| HNRNPK | 829.1 | 440 | 48.5 | 5.54 | 0.001861758 |
| MRPS18A | 16.8 | 196 | 22.2 | 10.33 | 0.001884839 |
| CRTAP | 43.94 | 401 | 46.5 | 5.73 | 0.001893286 |
| AHCTF1 | 16.43 | 2266 | 252.3 | 6.6 | 0.001903132 |
| ATPAF2 | 3.83 | 289 | 32.8 | 7.09 | 0.001910812 |
| EMC8 | 10.9 | 210 | 23.8 | 6.4 | 0.001911706 |
| TRIM26 | 0 | 539 | 62.1 | 5.03 | 0.001920832 |
| SUPT16H | 275.78 | 1047 | 119.8 | 5.66 | 0.001960048 |
| SPTBN1 | 1401.4 | 2364 | 274.4 | 5.57 | 0.001971569 |
| MAPRE1 | 107.93 | 268 | 30 | 5.14 | 0.001996299 |
| KCMF1 | 5.62 | 381 | 41.9 | 5.66 | 0.00208832 |
| CTNNBL1 | 29.4 | 563 | 65.1 | 5.05 | 0.002121885 |
| MTO1 | 0 | 595 | 66.8 | 8.35 | 0.002143495 |
| PITPNB | 31.45 | 271 | 31.5 | 6.87 | 0.002160744 |
| ERBIN | 2.38 | 1302 | 146 | 5.29 | 0.002213436 |
| ERCC6 | 3.43 | 1493 | 168.3 | 8.09 | 0.002219299 |
| TACC2 | 4.93 | 571 | 64.1 | 5.33 | 0.00223444 |
| KRT2 | 57.84 | 639 | 65.4 | 8 | 0.002240147 |
| PSMD12 | 68 | 456 | 52.9 | 7.65 | 0.002252152 |
| CDK5RAP1 | 2.52 | 426 | 48 | 8.18 | 0.00226263 |
| GGA3 | 0 | 651 | 70.1 | 6.25 | 0.00227153 |
| PABPC1 | 625.14 | 547 | 61.1 | 9.07 | 0.002297745 |
| TCP1 | 390.18 | 556 | 60.3 | 6.11 | 0.002298677 |
| SOGA1 | 3.82 | 1661 | 183.7 | 6.52 | 0.002305702 |
| PDLIM1 | 81.1 | 329 | 36 | 7.02 | 0.002311012 |
| OTUB1 | 107.33 | 271 | 31.3 | 4.94 | 0.002315108 |
| SOX9 | 3.2 | 509 | 56.1 | 6.81 | 0.002325731 |
| ARMC10 | 2.74 | 225 | 24.7 | 7.46 | 0.002351871 |
| USP14 | 172.48 | 494 | 56 | 5.3 | 0.00235874 |
| SMS | 80.5 | 366 | 41.2 | 5.02 | 0.002379157 |
| WNK1 | 0 | 1975 | 206.5 | 6.15 | 0.002397564 |
| DSTN | 322.48 | 165 | 18.5 | 7.85 | 0.002457252 |
| DECR1 | 61.94 | 326 | 35 | 8.9 | 0.002475867 |
| DYNLT1 | 7.97 | 113 | 12.4 | 5.08 | 0.002485654 |
| LRRFIP2 | 10.46 | 400 | 45.4 | 5.53 | 0.002500938 |
| HIST1H1C | 252.4 | 213 | 21.4 | 10.93 | 0.002502005 |
| NXF1 | 8.22 | 619 | 70.1 | 8.51 | 0.002507827 |
| CHMP2A | 2.41 | 222 | 25.1 | 5.97 | 0.002514607 |
| GNG12 | 53.89 | 72 | 8 | 8.97 | 0.002519253 |
| EIF2B1 | 27.91 | 305 | 33.7 | 7.33 | 0.002538067 |
| ILF3 | 393.44 | 698 | 75.5 | 8.41 | 0.002578361 |
| GNL1 | 15.63 | 607 | 68.6 | 5.8 | 0.002590018 |
| EGFR | 51.36 | 1091 | 120.7 | 6.37 | 0.002596 |
| HERC4 | 14 | 947 | 106.6 | 6.47 | 0.002637877 |
| TMED2 | 57.05 | 201 | 22.7 | 5.17 | 0.002685104 |
| CHST14 | 2.69 | 376 | 43 | 9.48 | 0.002724982 |
| LANCL1 | 87.14 | 399 | 45.3 | 7.75 | 0.002725626 |
| STAM | 10.77 | 403 | 44.9 | 4.87 | 0.002777813 |
| ZPR1 | 18.82 | 459 | 50.9 | 4.73 | 0.002807461 |
| FASN | 2420.8 | 2511 | 273.3 | 6.44 | 0.002838101 |
| COPS2 | 50.96 | 443 | 51.6 | 5.53 | 0.002843557 |
| RAP1B | 121.11 | 165 | 18.8 | 8.53 | 0.002851878 |
| MT-ND6 | 8.07 | 174 | 18.6 | 4.22 | 0.002871102 |
| PSMA3 | 212.26 | 248 | 27.6 | 5.33 | 0.002878139 |
| PPIA | 1250.04 | 165 | 18 | 7.81 | 0.002901616 |
| NOC2L | 61.49 | 749 | 84.9 | 5.62 | 0.002917116 |
| DCP1A | 2.67 | 582 | 63.3 | 6.25 | 0.002927743 |
| CDK7 | 3.79 | 346 | 39 | 8.47 | 0.002930953 |
| LSG1 | 2.18 | 658 | 75.2 | 6.38 | 0.003037767 |
| PSMG2 | 37.93 | 233 | 26.3 | 7.96 | 0.003089937 |
| DLD | 249.83 | 509 | 54.1 | 7.85 | 0.003091401 |
| PPME1 | 55.71 | 386 | 42.3 | 5.97 | 0.003092894 |
| SPRYD4 | 2.21 | 207 | 23.1 | 6.93 | 0.003096806 |
| MT1L | 0 | 61 | 6.1 | 7.96 | 0.003122981 |
| UROD | 28.44 | 367 | 40.8 | 6.14 | 0.003141771 |
| NRBP1 | 17.72 | 535 | 59.8 | 5.08 | 0.003161904 |
| DNASE2 | 2.34 | 305 | 33.6 | 8.32 | 0.003243729 |
| ATP5F1B | 1350.5 | 529 | 56.5 | 5.4 | 0.003257971 |
| UBE2M | 48.96 | 183 | 20.9 | 7.69 | 0.003303014 |
| RNASEH2A | 16.41 | 299 | 33.4 | 5.25 | 0.003306651 |
| ATP5F1A | 1092.6 | 553 | 59.7 | 9.13 | 0.003329646 |
| CFDP1 | 0 | 217 | 24 | 4.49 | 0.003337637 |
| HAGH | 15.63 | 260 | 28.8 | 7.33 | 0.003353076 |
| SMC2 | 81.19 | 1099 | 124.8 | 8.25 | 0.003355421 |
| TPD52L1 | 2.79 | 102 | 11.7 | 8.94 | 0.003365026 |
| SLC7A5 | 46.61 | 507 | 55 | 7.72 | 0.003377972 |
| MAP4 | 200.38 | 1152 | 120.9 | 5.43 | 0.003378011 |
| CWC22 | 2.86 | 908 | 105.4 | 7.03 | 0.003385946 |
| TMEM70 | 9.35 | 161 | 18.6 | 6.8 | 0.003435879 |
| ABRACL | 13.37 | 81 | 9.1 | 6.29 | 0.003435881 |
| TRABD | 4.19 | 330 | 37.1 | 7.44 | 0.003492377 |
| SRGAP2 | 2.85 | 1071 | 120.8 | 6.7 | 0.00353 |
| OTUD7B | 4.28 | 814 | 88.9 | 6.68 | 0.003587773 |
| CDK1 | 103.97 | 297 | 34.1 | 8.4 | 0.003641832 |
| SLC9A1 | 3.95 | 815 | 90.7 | 7.21 | 0.003689194 |
| ESYT1 | 128.26 | 1104 | 122.8 | 5.83 | 0.003720242 |
| P4HA2 | 0 | 533 | 60.6 | 5.71 | 0.003731734 |
| CD44 | 178.28 | 340 | 37.3 | 5.52 | 0.003756934 |
| MED23 | 0 | 1359 | 155.5 | 7.4 | 0.003827892 |
| JPT1 | 128.68 | 154 | 16 | 5.6 | 0.003889248 |
| CHMP1B | 20.89 | 199 | 22.1 | 8.1 | 0.003946014 |
| N/A | 2.4 | 355 | 38 | 5.26 | 0.003955904 |
| PML | 19.24 | 560 | 62 | 6.42 | 0.004037199 |
| CARS | 26.91 | 726 | 82.8 | 7.02 | 0.004092802 |
| SLC25A11 | 77.04 | 314 | 34 | 9.91 | 0.004116339 |
| MYC | 366.12 | 453 | 50.4 | 5.28 | 0.004125 |
| PEF1 | 13.92 | 284 | 30.4 | 6.54 | 0.004151173 |
| PSMD14 | 52.38 | 310 | 34.6 | 6.52 | 0.004226402 |
| MRPL48 | 0 | 194 | 21.7 | 8.62 | 0.004287443 |
| RPL5 | 350.81 | 297 | 34.3 | 9.72 | 0.004351349 |
| TRMT61A | 12.84 | 289 | 31.4 | 7.36 | 0.004424569 |
| CC2D1A | 6.9 | 950 | 103.9 | 8.09 | 0.004524658 |
| YWHAG | 969.23 | 247 | 28.3 | 4.89 | 0.004598559 |
| IDH3G | 41.55 | 380 | 41.4 | 8.82 | 0.004609432 |
| SAP30BP | 3.27 | 292 | 32 | 5.07 | 0.004614443 |
| DNM1L | 24.67 | 699 | 78.1 | 6.81 | 0.004642174 |
| MRPL24 | 12.12 | 216 | 24.9 | 9.29 | 0.004644146 |
| HSP90AB3P | 1311.03 | 597 | 68.3 | 4.79 | 0.004646422 |
| RACGAP1 | 17.55 | 632 | 71 | 8.88 | 0.00468032 |
| SNAT2 | 0 | 406 | 45.2 | 8.89 | 0.00469742 |
| ANKRD12 | 0 | 2039 | 232.9 | 7.06 | 0.004756851 |
| KIF5B | 206.48 | 963 | 109.6 | 6.51 | 0.00477719 |
| ITGA3 | 2.8 | 1051 | 116.5 | 6.77 | 0.004805751 |
| GNA13 | 35.42 | 377 | 44 | 8 | 0.004817815 |
| MMAB | 11.29 | 250 | 27.4 | 8.6 | 0.004841921 |
| EDF1 | 45.06 | 148 | 16.4 | 9.95 | 0.004875807 |
| MARS2 | 3.33 | 593 | 66.5 | 8.09 | 0.004878799 |
| PYCR3 | 6.63 | 274 | 28.6 | 7.5 | 0.004954508 |
| GIPC1 | 2 | 236 | 26.1 | 5.57 | 0.005007568 |
| L1RE1 | 3.2 | 338 | 40 | 9.51 | 0.005046608 |
| TRIP6 | 107.05 | 476 | 50.3 | 7.37 | 0.00508603 |
| SLC25A17 | 10.59 | 307 | 34.5 | 10.08 | 0.005089174 |
| WARS | 331.5 | 471 | 53.1 | 6.23 | 0.005095796 |
| CASP2 | 2.6 | 313 | 34.9 | 6.52 | 0.005205913 |
| RPE | 12.23 | 199 | 21.7 | 5.81 | 0.005229245 |
| EIF3J | 76.09 | 258 | 29 | 4.83 | 0.005263138 |
| PPFIA2 | 3.19 | 1152 | 131.5 | 6.65 | 0.005271597 |
| EZR | 1161.05 | 586 | 69.4 | 6.27 | 0.005295044 |
| SSR2 | 0 | 183 | 20.1 | 8.35 | 0.005363726 |
| UGP2 | 128.1 | 508 | 56.9 | 8.15 | 0.005391163 |
| CNOT10 | 5.04 | 695 | 76.9 | 7.56 | 0.00540298 |
| CDC42BPA | 66.46 | 1741 | 197.8 | 6.06 | 0.005413699 |
| DNAJC12 | 0 | 107 | 12.4 | 5.11 | 0.005439148 |
| COX5A | 135.59 | 150 | 16.8 | 6.79 | 0.005449764 |
| SEPT2 | 233.54 | 361 | 41.5 | 6.6 | 0.005452357 |
| CD44 | 88.46 | 742 | 81.5 | 5.13 | 0.005476 |
| YBX3 | 178.97 | 372 | 40.1 | 9.77 | 0.005519885 |
| ZC3H15 | 57.38 | 426 | 48.6 | 5.31 | 0.005630171 |
| MRPL47 | 23.36 | 230 | 27.1 | 10.35 | 0.005633441 |
| ZDHHC5 | 0 | 662 | 71.9 | 8.9 | 0.00567113 |
| ZNF550 | 0 | 390 | 44.8 | 8.62 | 0.005805479 |
| RAD23B | 147.15 | 409 | 43.1 | 4.84 | 0.0058174 |
| OAS3 | 11.6 | 1087 | 121.1 | 8.4 | 0.005844947 |
| EIF3D | 134.95 | 499 | 58.1 | 5.71 | 0.005905269 |
| DCAF8 | 3.04 | 597 | 66.8 | 5.39 | 0.005913785 |
| PRDX2 | 559.19 | 198 | 21.9 | 5.97 | 0.006013013 |
| SLC25A18 | 11.63 | 315 | 33.8 | 9.25 | 0.006036453 |
| CKAP4 | 700.61 | 602 | 66 | 5.92 | 0.006219634 |
| RAB6A | 149.09 | 208 | 23.6 | 5.54 | 0.006282358 |
| PAFAH1B2 | 55.83 | 229 | 25.6 | 5.92 | 0.006427838 |
| JAK1 | 2.44 | 1154 | 133.3 | 7.48 | 0.006489315 |
| ALDH7A1 | 47.71 | 511 | 55.3 | 6.86 | 0.006508286 |
| BCLAF1 | 21.83 | 869 | 100.2 | 9.95 | 0.006511219 |
| TMED4 | 52.02 | 227 | 25.9 | 8.28 | 0.006523584 |
| TXNDC12 | 68.96 | 172 | 19.2 | 5.4 | 0.006547813 |
| LMNB1 | 467.38 | 586 | 66.4 | 5.16 | 0.006592606 |
| CCT6A | 278.4 | 531 | 58 | 6.68 | 0.006594086 |
| ADO | 6.02 | 270 | 29.7 | 6.04 | 0.006626515 |
| PTDSS1 | 18.52 | 327 | 38.2 | 8.7 | 0.006637851 |
| RNF113A | 7.81 | 343 | 38.8 | 5.69 | 0.006639189 |
| LMAN2L | 6.47 | 214 | 24.7 | 8.44 | 0.006731065 |
| YME1L1 | 19.97 | 683 | 75.9 | 8.57 | 0.006786209 |
| PRKCA | 2.16 | 672 | 76.7 | 7.05 | 0.006809251 |
| MLEC | 54.58 | 292 | 32.2 | 5.41 | 0.00687663 |
| NSDHL | 50.03 | 373 | 41.9 | 8.06 | 0.00687913 |
| KLHL38 | 2.04 | 581 | 65.5 | 7.93 | 0.006882195 |
| THYN1 | 2.96 | 225 | 25.7 | 9.25 | 0.007025147 |
| VDAC3 | 248.3 | 283 | 30.6 | 8.66 | 0.007175867 |
| MRPS5 | 5.36 | 430 | 48 | 9.92 | 0.007198903 |
| TOP2A | 243.83 | 1531 | 174.3 | 8.72 | 0.007215688 |
| TSPYL1 | 2.37 | 437 | 49.2 | 5.45 | 0.007226387 |
| GLUD1 | 106.55 | 558 | 61.4 | 7.8 | 0.007307747 |
| FLII | 125.35 | 1269 | 144.7 | 6.05 | 0.007338445 |
| HINT1 | 209.09 | 126 | 13.8 | 6.95 | 0.007339674 |
| SLK | 25.98 | 1204 | 138.9 | 5.1 | 0.007352009 |
| SCAMP1 | 4.83 | 338 | 37.9 | 7.42 | 0.007393351 |
| ACAA2 | 119.04 | 397 | 41.9 | 8.09 | 0.007404962 |
| COPS8 | 41.34 | 160 | 17.9 | 7.25 | 0.007411608 |
| MCM7 | 156.94 | 719 | 81.3 | 6.46 | 0.007460586 |
| ORC3 | 4.02 | 568 | 65.9 | 7.62 | 0.007517209 |
| EMC4 | 4.54 | 136 | 15.1 | 9.1 | 0.00752604 |
| PSME3 | 158.18 | 254 | 29.5 | 5.95 | 0.00758996 |
| ERCC6L | 12.24 | 1250 | 141 | 5.31 | 0.007694871 |
| ABCB4 | 86.14 | 1349 | 147.8 | 8.93 | 0.00769845 |
| GUK1 | 2.44 | 197 | 21.7 | 6.55 | 0.007740704 |
| PPP5C | 45.95 | 499 | 56.8 | 6.28 | 0.007763085 |
| RECQL | 74.77 | 649 | 73.4 | 7.88 | 0.00779108 |
| HEBP1 | 4 | 189 | 21.1 | 5.8 | 0.00785186 |
| TROVE2 | 132.14 | 538 | 60.6 | 8.03 | 0.007874632 |
| TOMM22 | 56.06 | 142 | 15.5 | 4.34 | 0.007883644 |
| BLVRB | 242.52 | 206 | 22.1 | 7.65 | 0.007993747 |
| AGPAT1 | 6.13 | 283 | 31.7 | 9.38 | 0.00802784 |
| MATR3 | 395.29 | 847 | 94.6 | 6.25 | 0.008061017 |
| C7orf50 | 31.81 | 194 | 22.1 | 9.64 | 0.008112121 |
| MUC16 | 0 | 22152 | 2351.2 | 6 | 0.008117524 |
| GLRX3 | 162.87 | 335 | 37.4 | 5.39 | 0.008180979 |
| FOXS1 | 2.69 | 330 | 35.4 | 9.07 | 0.008267072 |
| EIF6 | 125.86 | 245 | 26.6 | 4.68 | 0.0083176 |
| PPP4R1 | 9.45 | 933 | 105.1 | 4.81 | 0.008332332 |
| BPNT1 | 57.58 | 272 | 29.2 | 8.28 | 0.008410788 |
| PCYT1A | 31.35 | 367 | 41.7 | 7.25 | 0.00845902 |
| NBEAL2 | 1.7 | 2570 | 282.7 | 6.54 | 0.008549896 |
| RBM39 | 122.55 | 524 | 58.6 | 10.13 | 0.00856262 |
| ERH | 68.63 | 104 | 12.3 | 5.92 | 0.008626645 |
| ROCK2 | 12.82 | 1388 | 160.8 | 6.02 | 0.008642868 |
| HRAS | 41.64 | 189 | 21.3 | 5.16 | 0.00864631 |
| HIST1H1E | 274.55 | 219 | 21.9 | 11.03 | 0.00866412 |
| RPL31 | 228.7 | 125 | 14.5 | 10.54 | 0.008731925 |
| PDXP | 2.83 | 296 | 31.7 | 6.55 | 0.008743446 |
| WDR92 | 3.52 | 288 | 32.1 | 8.02 | 0.008757469 |
| ABCF2 | 69.62 | 623 | 71.2 | 7.37 | 0.008779881 |
| ITGA1 | 2.25 | 1179 | 130.8 | 6.29 | 0.008819033 |
| KPNB1 | 731.17 | 876 | 97.1 | 4.78 | 0.008955518 |
| SRGN | 6.93 | 158 | 17.6 | 4.96 | 0.00897612 |
| ACBD6 | 37.47 | 282 | 31.1 | 5.11 | 0.009163524 |
| ACYP1 | 12.31 | 99 | 11.3 | 9.31 | 0.009179347 |
| NOL9 | 2.51 | 702 | 79.3 | 9.13 | 0.009235503 |
| CDC25A | 981.72 | 524 | 59.1 | 6.49 | 0.00924646 |
| CAVIN2 | 234.22 | 425 | 47.1 | 5.21 | 0.009272387 |
| OSGEPL1 | 3.71 | 414 | 45.1 | 8.56 | 0.009281426 |
| VTA1 | 13.88 | 222 | 24.5 | 5.29 | 0.00929325 |
| FARSB | 239.18 | 589 | 66.1 | 6.84 | 0.009333763 |
| UBE2R2 | 6.55 | 238 | 27.1 | 4.42 | 0.009353825 |
| TUBG1 | 29.12 | 451 | 51.1 | 6.14 | 0.009435855 |
| SRSF1 | 305.5 | 248 | 27.7 | 10.36 | 0.009479957 |
| PLCD3 | 8.09 | 789 | 89.2 | 6.98 | 0.009484737 |
| CUTC | 0 | 273 | 29.3 | 8.18 | 0.009499932 |
| MYO5A | 0 | 1828 | 212.1 | 8.6 | 0.009502003 |
| ARHGEF2 | 32.39 | 958 | 108.2 | 6.87 | 0.009518041 |
| EIF2B4 | 13.08 | 522 | 57.4 | 9.38 | 0.009537342 |
| REPS1 | 2.8 | 705 | 76.8 | 5.83 | 0.009632046 |
| PHB2 | 498.74 | 299 | 33.3 | 9.83 | 0.009714399 |
| MRRF | 13.81 | 201 | 22.4 | 9.72 | 0.009717356 |
| NME4 | 17.42 | 187 | 20.6 | 10.29 | 0.009741902 |
| RHOT1 | 11.3 | 247 | 28.2 | 5.08 | 0.009786065 |
| NIFK | 71.55 | 293 | 34.2 | 9.88 | 0.00983298 |
| REEP5 | 24.2 | 189 | 21.5 | 8.1 | 0.009889367 |
| GTF3C2 | 2.53 | 585 | 64 | 6.54 | 0.00995913 |
| PDCD4 | 30.33 | 458 | 50.5 | 5.48 | 0.009983318 |
| NDUFS4 | 4.61 | 175 | 20.1 | 10.3 | 0.010030367 |
| OXSR1 | 68.95 | 527 | 58 | 6.43 | 0.010332002 |
| AKAP1 | 2.53 | 593 | 62.9 | 4.84 | 0.010447931 |
| PSMC3 | 219.25 | 439 | 49.2 | 5.24 | 0.010450502 |
| MPC2 | 0 | 127 | 14.3 | 10.43 | 0.010450667 |
| CBX5 | 69.56 | 191 | 22.2 | 5.86 | 0.010545968 |
| EAPP | 3.16 | 285 | 32.7 | 5.12 | 0.010678783 |
| MP68 | 2.35 | 58 | 6.7 | 10.08 | 0.010680557 |
| RAB3B | 145.01 | 219 | 24.7 | 5.02 | 0.010727851 |
| CLUH | 149.39 | 1309 | 146.6 | 6.13 | 0.010731152 |
| EPHB4 | 5.27 | 987 | 108.2 | 6.9 | 0.010802511 |
| CDK4 | 22.17 | 303 | 33.7 | 7.01 | 0.010816219 |
| GCN1 | 373.29 | 2671 | 292.6 | 7.47 | 0.01092721 |
| TPM2 | 535.23 | 284 | 33 | 4.67 | 0.010988727 |
| GALNT1 | 4.87 | 559 | 64.2 | 7.72 | 0.01100496 |
| CPSF7 | 12.99 | 462 | 51.1 | 7.78 | 0.011188059 |
| PSMF1 | 10.93 | 271 | 29.8 | 5.74 | 0.011265095 |
| KIF2C | 2.72 | 671 | 75.5 | 7.56 | 0.011288342 |
| METTL1 | 13.78 | 276 | 31.5 | 7.64 | 0.011322931 |
| ISG15 | 4.49 | 165 | 17.9 | 7.44 | 0.011356307 |
| SNRPD2 | 169.92 | 118 | 13.5 | 9.91 | 0.011372223 |
| DPH5 | 5.53 | 234 | 26.1 | 5.02 | 0.011521087 |
| ILKAP | 63.49 | 392 | 42.9 | 7.09 | 0.011580103 |
| CRYAB | 2.73 | 175 | 20.1 | 7.33 | 0.011612297 |
| RBMX | 409.65 | 391 | 42.3 | 10.05 | 0.011724967 |
| GBF1 | 63.41 | 1859 | 206.3 | 5.73 | 0.011740913 |
| UQCRB | 44.86 | 111 | 13.5 | 8.78 | 0.011834147 |
| MFSD5 | 4.17 | 450 | 49.7 | 7.8 | 0.011877539 |
| USP4 | 15.15 | 916 | 103.9 | 5.52 | 0.011907543 |
| SMARCA1 | 18.42 | 1042 | 121.1 | 8.18 | 0.011931221 |
| PRKAA1 | 9.07 | 559 | 64 | 8.12 | 0.011951796 |
| COG5 | 0 | 823 | 90.8 | 6.51 | 0.011955268 |
| ZFAND5 | 2.78 | 213 | 23.1 | 8.51 | 0.012029122 |
| SRP54 | 57.89 | 504 | 55.7 | 8.75 | 0.012083329 |
| UBL4A | 7.35 | 157 | 17.8 | 8.66 | 0.012128644 |
| PIK3CB | 2.45 | 1070 | 122.7 | 7.09 | 0.012193763 |
| ZNF667 | 0 | 610 | 70.1 | 9.73 | 0.012265871 |
| CHORDC1 | 79.73 | 332 | 37.5 | 7.87 | 0.012418217 |
| FOXRED1 | 3.03 | 472 | 52.1 | 6.61 | 0.012494452 |
| ATP6V1E1 | 17.95 | 204 | 23.6 | 8.98 | 0.012551003 |
| CTSD | 91.07 | 412 | 44.5 | 6.54 | 0.01256294 |
| HLA-B | 132.85 | 362 | 40.5 | 6.46 | 0.012642639 |
| ARL2 | 18.84 | 157 | 18 | 5.94 | 0.012647189 |
| SPDL1 | 3.24 | 506 | 58.5 | 5.72 | 0.012800992 |
| RIN1 | 4.53 | 721 | 77.5 | 7.2 | 0.012838557 |
| POLD1 | 29.11 | 1107 | 123.6 | 7.03 | 0.012844029 |
| RPL39 | 19.55 | 51 | 6.4 | 12.56 | 0.012874894 |
| PGAM5 | 76.34 | 289 | 32 | 8.68 | 0.012965719 |
| EIF2S1 | 265.48 | 315 | 36.1 | 5.08 | 0.013010214 |
| FN3KRP | 2.09 | 309 | 34.4 | 7.33 | 0.013047527 |
| ZNFX1 | 2.88 | 1918 | 220.1 | 7.3 | 0.01306857 |
| MYL12A | 256.17 | 171 | 19.8 | 4.81 | 0.013146678 |
| DAGLB | 2.5 | 391 | 43.2 | 6.83 | 0.013161357 |
| ZMPSTE24 | 44.11 | 475 | 54.8 | 7.49 | 0.013206616 |
| USP16 | 2.65 | 408 | 46.6 | 7.27 | 0.01332375 |
| CHD9 | 8.27 | 2881 | 323.9 | 6.92 | 0.013525103 |
| NT5C3A | 4.8 | 285 | 32.5 | 5.72 | 0.013547693 |
| ALG3 | 2.68 | 438 | 50.1 | 9.44 | 0.01362125 |
| RRAS | 15.19 | 218 | 23.5 | 6.93 | 0.013629254 |
| CASP9 | 0 | 333 | 36.5 | 6.43 | 0.013705371 |
| VAPA | 54.37 | 249 | 27.9 | 8.62 | 0.013995851 |
| TIMM8B | 2.91 | 83 | 9.3 | 5.12 | 0.014042304 |
| SAFB | 68.81 | 848 | 95.1 | 6.46 | 0.014086793 |
| MYL6 | 317.93 | 151 | 16.9 | 4.65 | 0.014201526 |
| NDUFAB1 | 71.46 | 156 | 17.4 | 4.93 | 0.014328995 |
| HNRNPU | 990.18 | 806 | 88.9 | 5.78 | 0.014342838 |
| SOD2 | 112.98 | 176 | 19.7 | 8.03 | 0.014351479 |
| NPC2 | 17.08 | 125 | 13.4 | 8.34 | 0.014399031 |
| ATP5G1 | 22.6 | 136 | 14.3 | 9.74 | 0.014399209 |
| ESF1 | 3.55 | 851 | 98.7 | 5.11 | 0.01445365 |
| CDR2L | 0 | 465 | 53 | 6.01 | 0.014460195 |
| RBM6 | 6.37 | 601 | 69.1 | 8.73 | 0.014502753 |
| CYFIP1 | 59.13 | 1253 | 145.1 | 6.9 | 0.014566558 |
| COX20 | 9.98 | 118 | 13.3 | 8.76 | 0.014645019 |
| ARPC2 | 148.33 | 300 | 34.3 | 7.36 | 0.014655349 |
| TFF1 | 41.5 | 84 | 9.1 | 4.29 | 0.0146845 |
| GEMIN5 | 71.22 | 1508 | 168.5 | 6.62 | 0.014728981 |
| CSE1L | 452.59 | 945 | 107.7 | 5.82 | 0.014730271 |
| MRFAP1 | 5.1 | 127 | 14.6 | 4.7 | 0.014854146 |
| EIF4EBP2 | 3.31 | 120 | 12.9 | 6.67 | 0.014922111 |
| GDAP1L1 | 0 | 367 | 41.9 | 6.6 | 0.01494289 |
| SLC39A11 | 3.55 | 335 | 34.7 | 5.38 | 0.014992834 |
| LETM1 | 97.81 | 739 | 83.3 | 6.7 | 0.015022911 |
| PTS | 2.78 | 145 | 16.4 | 6.68 | 0.01510568 |
| RALA | 14.88 | 206 | 23.6 | 7.11 | 0.01523177 |
| SNRPF | 31.85 | 86 | 9.7 | 4.67 | 0.015253816 |
| API5 | 46.31 | 445 | 50.3 | 5.45 | 0.015305311 |
| MRPS24 | 3.01 | 167 | 19 | 9.38 | 0.015349929 |
| TOM1L2 | 2.12 | 462 | 50.5 | 4.73 | 0.015503975 |
| ODF2 | 0 | 657 | 75.6 | 7.62 | 0.01559727 |
| ZC3H14 | 8.77 | 711 | 80 | 7.31 | 0.015727246 |
| WDR43 | 35.55 | 677 | 74.8 | 5.57 | 0.015727568 |
| RUVBL1 | 325.41 | 456 | 50.2 | 6.42 | 0.015753945 |
| LSMEM1 | 0 | 59 | 6.5 | 8.02 | 0.015759682 |
| ADNP | 2.29 | 1102 | 123.5 | 7.34 | 0.015793782 |
| COQ8A | 2.14 | 163 | 18.9 | 6.68 | 0.015851337 |
| EIF3B | 327.9 | 814 | 92.4 | 5 | 0.015987053 |
| TRIM27 | 3.55 | 358 | 41.4 | 5.82 | 0.015991759 |
| MMS19 | 14.15 | 1030 | 113.2 | 6.35 | 0.016095968 |
| RDH11 | 28.96 | 318 | 35.4 | 8.82 | 0.01613609 |
| GTF2H2 | 5.65 | 395 | 44.4 | 6.76 | 0.016203153 |
| HNRNPAB | 430.14 | 285 | 30.6 | 7.91 | 0.016279451 |
| PTPRJ | 2.94 | 539 | 57.2 | 5.6 | 0.016282185 |
| LARP4B | 3.03 | 738 | 80.5 | 6.92 | 0.016357739 |
| WDR44 | 5.34 | 905 | 100.3 | 5.45 | 0.016447285 |
| TSR3 | 4.11 | 312 | 33.6 | 6.87 | 0.016448502 |
| ATP5O | 138.62 | 213 | 23.3 | 9.96 | 0.016456983 |
| TXNL4A | 16.22 | 142 | 16.8 | 5.85 | 0.016475191 |
| NUDT4 | 7.97 | 180 | 20.3 | 6.35 | 0.016475837 |
| PLEKHA4 | 0 | 583 | 64.2 | 10.07 | 0.016495078 |
| SNX8 | 3.05 | 465 | 52.5 | 7.39 | 0.016577741 |
| SPATA20 | 6.74 | 742 | 83.3 | 6.83 | 0.016648562 |
| FAM136A | 6.82 | 138 | 15.6 | 7.61 | 0.016788909 |
| CCNT1 | 0 | 726 | 80.6 | 8.78 | 0.016806329 |
| CCT7 | 517.54 | 543 | 59.3 | 7.65 | 0.016820241 |
| PAPSS2 | 551.03 | 614 | 69.5 | 8.03 | 0.016929044 |
| OGT | 8.39 | 1046 | 116.8 | 6.7 | 0.016958275 |
| SAR1A | 108.83 | 198 | 22.4 | 6.68 | 0.016999672 |
| HIST2H2BD | 158.79 | 164 | 18 | 10.58 | 0.017107859 |
| QARS | 139.97 | 764 | 86.5 | 7.05 | 0.017141231 |
| SLC16A3 | 131.28 | 465 | 49.4 | 7.96 | 0.017188473 |
| ATP5L | 70.19 | 103 | 11.4 | 9.64 | 0.017249808 |
| ATIC | 507.3 | 592 | 64.6 | 6.71 | 0.017428659 |
| MT-CYB | 2.93 | 380 | 42.7 | 8.05 | 0.017446414 |
| SFXN2 | 2.23 | 322 | 36.2 | 9.41 | 0.017593377 |
| EIF4A3 | 354.15 | 411 | 46.8 | 6.73 | 0.017664156 |
| RTF1 | 8.03 | 710 | 80.3 | 8.15 | 0.017668521 |
| RAB32 | 29.87 | 225 | 25 | 6.54 | 0.017831752 |
| CPNE9 | 17.28 | 553 | 61.8 | 5.34 | 0.017902367 |
| GOT1 | 155.94 | 413 | 46.2 | 7.01 | 0.017916878 |
| NUDT21 | 52.06 | 227 | 26.2 | 8.82 | 0.018045647 |
| PNISR | 2.16 | 413 | 47.4 | 5.17 | 0.018067792 |
| CNPY2 | 74.77 | 182 | 20.6 | 4.92 | 0.018213866 |
| GSTM2 | 59.01 | 191 | 22.6 | 5.31 | 0.018259347 |
| EIF2S3 | 251.74 | 472 | 51.1 | 8.4 | 0.018371952 |
| MYO18A | 6.37 | 2002 | 226.5 | 6.2 | 0.018386221 |
| ASNA1 | 40.11 | 348 | 38.8 | 4.91 | 0.018413636 |
| MAU2 | 0 | 189 | 21.3 | 6.71 | 0.018457189 |
| PSMG4 | 3.27 | 104 | 11.2 | 6.51 | 0.018483872 |
| GSS | 93.03 | 474 | 52.4 | 5.92 | 0.018543661 |
| TAF4 | 2.18 | 1085 | 110 | 9.94 | 0.018692251 |
| EPB41 | 15.92 | 566 | 63.2 | 6.67 | 0.018760396 |
| MRPL38 | 33.78 | 380 | 44.6 | 7.53 | 0.018772688 |
| PPP2R5C | 20.42 | 540 | 62.7 | 8.7 | 0.01878083 |
| MRPS12 | 4.7 | 138 | 15.2 | 10.29 | 0.018843348 |
| CYP1B1 | 24.26 | 543 | 60.8 | 8.98 | 0.019065817 |
| USP36 | 2.78 | 1121 | 122.6 | 9.67 | 0.019076108 |
| MRPL2 | 5.08 | 305 | 33.3 | 11.3 | 0.019128252 |
| FAM20B | 25.05 | 409 | 46.4 | 6.87 | 0.019166217 |
| STAG2 | 20.99 | 1231 | 141.2 | 5.43 | 0.019238057 |
| FDFT1 | 28.33 | 306 | 35.1 | 5.85 | 0.0192929 |
| TOLLIP | 4.73 | 274 | 30.3 | 5.97 | 0.019361325 |
| CIAO1 | 2.66 | 339 | 37.8 | 4.97 | 0.01945891 |
| TCEAL1 | 0 | 159 | 18.6 | 5.02 | 0.019667911 |
| POLK | 0 | 371 | 42.3 | 6.65 | 0.019744616 |
| EIF3I | 143.1 | 325 | 36.5 | 5.64 | 0.019772687 |
| SREK1IP1 | 6.82 | 155 | 18.2 | 9.85 | 0.019820657 |
| C8orf82 | 3.58 | 208 | 22.8 | 11.02 | 0.019881329 |
| RPS25 | 198.99 | 125 | 13.7 | 10.11 | 0.020007019 |
| TECR | 60.57 | 308 | 36 | 9.45 | 0.020027595 |
| GOLGA5 | 6.75 | 691 | 78.1 | 5.54 | 0.020070623 |
| LRRC20 | 2.88 | 184 | 20.5 | 6.55 | 0.020084942 |
| NDUFS1 | 121.3 | 670 | 73.5 | 5.87 | 0.020250244 |
| COX6C | 17.93 | 75 | 8.8 | 10.39 | 0.020368326 |
| ORC2 | 3.75 | 577 | 65.9 | 6.51 | 0.020381248 |
| FDPS | 145.93 | 353 | 40.5 | 5.17 | 0.020621539 |
| SMAP1 | 3.25 | 436 | 47.8 | 8.43 | 0.020627976 |
| DOCK1 | 2.36 | 1865 | 215.2 | 7.56 | 0.020721769 |
| NDUFA2 | 14.52 | 99 | 10.9 | 9.57 | 0.020834054 |
| MDP1 | 2.66 | 122 | 14 | 5.81 | 0.021000297 |
| PRKCB | 4.28 | 671 | 76.8 | 7.01 | 0.021048985 |
| NDUFAF5 | 2.04 | 317 | 36.1 | 8.87 | 0.021249547 |
| FAM107B | 2.56 | 131 | 15.5 | 8.29 | 0.021320615 |
| SFXN1 | 158.73 | 322 | 35.6 | 9.07 | 0.021385161 |
| TPD52L2 | 85.96 | 206 | 22.2 | 5.36 | 0.021633769 |
| LSM4 | 5.23 | 139 | 15.3 | 9.99 | 0.021700072 |
| FAHD1 | 8.93 | 224 | 24.8 | 7.39 | 0.021757971 |
| WDR77 | 58.5 | 342 | 36.7 | 5.17 | 0.021823235 |
| RDH13 | 7.91 | 331 | 35.9 | 8.1 | 0.022133596 |
| ZCCHC3 | 2.57 | 404 | 43.6 | 8.53 | 0.022136531 |
| NDUFA12 | 4.3 | 145 | 17.1 | 9.63 | 0.022181927 |
| PDS5B | 5.57 | 1391 | 158.2 | 8.59 | 0.022250415 |
| TRMT2A | 2.91 | 562 | 61.9 | 7.97 | 0.022320974 |
| SRSF2 | 107.04 | 221 | 25.5 | 11.85 | 0.022572195 |
| PPP1R3D | 2.15 | 299 | 32.5 | 8.07 | 0.022608457 |
| CNN3 | 61.07 | 283 | 31.4 | 6.55 | 0.02280846 |
| FAM129A | 6.13 | 928 | 103.1 | 4.78 | 0.022931128 |
| STK4 | 3.21 | 462 | 52.3 | 5 | 0.02296238 |
| MCFD2 | 3.63 | 94 | 10.7 | 4.41 | 0.022996253 |
| UBE2B | 1.72 | 152 | 17.3 | 5.01 | 0.023047994 |
| NCAPG | 52.45 | 1015 | 114.3 | 5.59 | 0.023368979 |
| EPS15L1 | 11.39 | 601 | 66.4 | 5.26 | 0.023601613 |
| ILVBL | 14.65 | 632 | 67.8 | 8.15 | 0.023601804 |
| IKBIP | 18.19 | 350 | 39.3 | 9.17 | 0.02370202 |
| GTPBP10 | 2.44 | 308 | 34.6 | 8.98 | 0.023719804 |
| RNPEP | 281.25 | 650 | 72.5 | 5.74 | 0.023826478 |
| GPX8 | 16.25 | 209 | 23.9 | 9.35 | 0.023930151 |
| FADS2 | 3.07 | 386 | 45.5 | 7.47 | 0.024000097 |
| CDIPT | 5.01 | 168 | 18.6 | 8.21 | 0.024080543 |
| UBE2I | 52.37 | 158 | 18 | 8.66 | 0.024225155 |
| EVI5L | 0 | 794 | 91.3 | 5.34 | 0.024371291 |
| FAH | 50 | 419 | 46.3 | 6.95 | 0.024434446 |
| PRPF4 | 37.36 | 521 | 58.3 | 7.42 | 0.024440268 |
| HIST1H1B | 95.21 | 226 | 22.6 | 10.92 | 0.024565219 |
| JUN | 28.47 | 331 | 35.7 | 8.9 | 0.024569878 |
| NUMB | 0 | 592 | 64.5 | 8.37 | 0.024708103 |
| DYNC1I2 | 102.54 | 612 | 68.4 | 5.29 | 0.024713309 |
| PRSS3P2 | 10.62 | 247 | 26.5 | 6.01 | 0.024866618 |
| STUB1 | 83.06 | 303 | 34.8 | 5.87 | 0.024875473 |
| CPSF3 | 12.62 | 684 | 77.4 | 5.6 | 0.025073102 |
| YIPF4 | 0 | 244 | 27.1 | 4.65 | 0.025158082 |
| SMYD5 | 9.07 | 418 | 47.3 | 5.05 | 0.025258484 |
| TPX2 | 4.7 | 747 | 85.6 | 9.23 | 0.02531245 |
| TOR4A | 0 | 423 | 46.9 | 9.94 | 0.025593772 |
| TIMM8A | 33.47 | 97 | 11 | 5.16 | 0.025765392 |
| SCARB1 | 55.8 | 474 | 53.5 | 7.25 | 0.025974019 |
| NANS | 33.2 | 359 | 40.3 | 6.74 | 0.025996441 |
| ECSIT | 9.55 | 296 | 33.2 | 9.13 | 0.026053659 |
| DHX30 | 94.2 | 1155 | 129.4 | 8.53 | 0.026214078 |
| TRAPPC8 | 0 | 1359 | 152.9 | 6.79 | 0.02635745 |
| UBE2V2 | 97.59 | 145 | 16.4 | 8.09 | 0.026380198 |
| VPS26A | 35.66 | 327 | 38.1 | 6.57 | 0.026390737 |
| BCL2 | 272.44 | 239 | 26.3 | 6.75 | 0.02648134 |
| CRLF3 | 3.22 | 442 | 49.7 | 5.14 | 0.026610999 |
| EXOC3 | 2.66 | 641 | 73.8 | 7.15 | 0.026616709 |
| ULBP2 | 2.32 | 246 | 27.3 | 7.3 | 0.026689886 |
| ERG28 | 2.34 | 140 | 15.9 | 9.83 | 0.026760888 |
| MRPL42 | 3.84 | 142 | 16.7 | 8.35 | 0.026844358 |
| ZNHIT2 | 4.01 | 403 | 42.9 | 5.99 | 0.02690495 |
| KARS | 155.38 | 597 | 68 | 6.35 | 0.026946944 |
| WDR13 | 6.17 | 393 | 43 | 7.46 | 0.027049282 |
| NEB | 0 | 6669 | 772.4 | 9.07 | 0.027068216 |
| PFDN1 | 10.24 | 122 | 14.2 | 6.81 | 0.027089398 |
| CHAMP1 | 9.36 | 812 | 89 | 8.44 | 0.027123178 |
| GSTM3 | 295.53 | 225 | 26.5 | 5.54 | 0.027142402 |
| DCAF13 | 19.79 | 445 | 51.4 | 9.19 | 0.0271502 |
| SF3B3 | 308.89 | 1217 | 135.5 | 5.26 | 0.027383224 |
| FMNL1 | 11.3 | 1100 | 121.8 | 5.72 | 0.027448804 |
| SARS2 | 11.16 | 518 | 58.2 | 8.13 | 0.027615086 |
| ARG1 | 0 | 330 | 35.6 | 7.21 | 0.027831032 |
| SEPT11 | 113.85 | 429 | 49.4 | 6.81 | 0.027840419 |
| VEGFA | 11.145 | 412 | 45.5 | 9.22 | 0.027843156 |
| WDR26 | 8.58 | 645 | 70.4 | 6.15 | 0.028091567 |
| CCDC59 | 9.77 | 241 | 28.7 | 9.88 | 0.028106176 |
| TLE3 | 3.71 | 760 | 82.2 | 7.2 | 0.028231685 |
| SDF4 | 15.72 | 362 | 41.8 | 4.86 | 0.028464091 |
| PPIP5K2 | 3.6 | 1222 | 138 | 8.06 | 0.028526907 |
| STRAP | 128.02 | 350 | 38.4 | 5.12 | 0.028737913 |
| PDHB | 59.53 | 341 | 37.2 | 5.9 | 0.028877333 |
| NOL7 | 0 | 145 | 16.3 | 4.87 | 0.029154733 |
| CAPZA2 | 105.47 | 286 | 32.9 | 5.85 | 0.029509485 |
| COMMD4 | 3.51 | 199 | 21.8 | 7.31 | 0.029524745 |
| RABEPK | 2.02 | 321 | 34.8 | 6 | 0.029549384 |
| PRKAG1 | 2.76 | 299 | 34.1 | 8.46 | 0.029722069 |
| ENSA | 25.69 | 117 | 13 | 8.27 | 0.029801487 |
| RBM45 | 8.73 | 474 | 53.3 | 7.17 | 0.030029835 |
| ATP10B | 0 | 1461 | 165.3 | 6.89 | 0.030081992 |
| SRSF9 | 78.02 | 221 | 25.5 | 8.65 | 0.030132866 |
| CHTOP | 6.02 | 202 | 21.9 | 11.97 | 0.030294686 |
| EPS15 | 0 | 896 | 98.6 | 4.64 | 0.030605372 |
| NUDCD1 | 19.24 | 496 | 56.6 | 5.11 | 0.030743908 |
| TMEM120A | 0 | 322 | 35.9 | 8.43 | 0.030989971 |
| RIC8A | 19.42 | 530 | 59.6 | 5.33 | 0.031176344 |
| NUCKS1 | 78.04 | 243 | 27.3 | 5.08 | 0.031263503 |
| TAMM41 | 0 | 316 | 35.9 | 8.75 | 0.031337043 |
| PRDX4 | 327.65 | 271 | 30.5 | 6.29 | 0.031477925 |
| SND1 | 670.44 | 910 | 101.9 | 7.17 | 0.031532512 |
| METTL5 | 2.41 | 209 | 23.7 | 6.68 | 0.031858776 |
| RHBDD2 | 3.95 | 364 | 39.2 | 9.32 | 0.031882899 |
| ADI1 | 8.99 | 173 | 20.3 | 6.29 | 0.031916514 |
| PSMA2 | 151.36 | 234 | 25.9 | 7.43 | 0.032093567 |
| SYDE1 | 2.33 | 668 | 72.9 | 8.72 | 0.032346652 |
| RAB14 | 178.97 | 215 | 23.9 | 6.21 | 0.032377866 |
| RBM12 | 93.37 | 932 | 97.3 | 8.63 | 0.032431643 |
| SCO1 | 13.13 | 301 | 33.8 | 8.88 | 0.032549083 |
| GIT1 | 13.84 | 761 | 84.3 | 6.8 | 0.032684557 |
| BOD1L1 | 0 | 3051 | 330.3 | 5.08 | 0.032863241 |
| BCL7B | 5.63 | 145 | 16 | 5.15 | 0.033007102 |
| HEXB | 40.35 | 556 | 63.1 | 6.76 | 0.033237499 |
| ATXN10 | 59.89 | 475 | 53.5 | 5.25 | 0.033594693 |
| AFG3L2 | 60.48 | 797 | 88.5 | 8.66 | 0.03364339 |
| VTI1B | 0 | 232 | 26.7 | 9.04 | 0.033986421 |
| PPP6R3 | 30.44 | 791 | 88.9 | 4.58 | 0.034007993 |
| C5orf22 | 0 | 442 | 49.9 | 4.78 | 0.034567696 |
| MAPK1 | 32.14 | 360 | 41.4 | 6.5 | 0.03489 |
| LPGAT1 | 10.15 | 370 | 43.1 | 8.92 | 0.034904576 |
| NDUFB3 | 2.02 | 98 | 11.4 | 9.2 | 0.034941582 |
| MPDU1 | 57.85 | 186 | 19.7 | 8.41 | 0.03497114 |
| MED12 | 0 | 2176 | 242.8 | 7.05 | 0.035019456 |
| STK24 | 22.58 | 431 | 47.9 | 5.43 | 0.035034747 |
| CLPTM1L | 69.81 | 502 | 58.3 | 8.82 | 0.035281401 |
| STK17A | 0 | 414 | 46.5 | 5.15 | 0.03532259 |
| SDHB | 65.49 | 280 | 31.6 | 8.76 | 0.035533711 |
| SLITRK5 | 3.44 | 717 | 79.7 | 8.15 | 0.035614947 |
| CCDC50 | 4.14 | 306 | 35.8 | 6.65 | 0.035629017 |
| MYCBP | 6.31 | 103 | 12 | 5.91 | 0.035711673 |
| RCOR1 | 7.49 | 482 | 53 | 7.03 | 0.035729195 |
| PLBD2 | 3.22 | 557 | 61.8 | 6.8 | 0.03625146 |
| PTRH1 | 7.96 | 214 | 22.9 | 10.56 | 0.036306813 |
| ARMC6 | 2.46 | 476 | 51.5 | 6.05 | 0.036362316 |
| RBBP6 | 0 | 1758 | 197.2 | 9.63 | 0.036421144 |
| SAMM50 | 36.99 | 469 | 51.9 | 6.9 | 0.036448184 |
| SLC38A1 | 0 | 487 | 54 | 7.02 | 0.036549393 |
| BAP18 | 10.85 | 172 | 17.9 | 7.33 | 0.036571241 |
| ELOB | 59.55 | 118 | 13.1 | 4.88 | 0.036787363 |
| NONO | 591.55 | 471 | 54.2 | 8.95 | 0.037076271 |
| RACK1 | 1011.04 | 317 | 35.1 | 7.69 | 0.037113705 |
| PNPLA6 | 0 | 1300 | 143.3 | 7.74 | 0.037267578 |
| NDUFB2 | 4.6 | 105 | 12.1 | 5.74 | 0.037355369 |
| TMEM14C | 12.97 | 112 | 11.6 | 9.88 | 0.037430592 |
| PPP2R5E | 16.05 | 462 | 54 | 6.57 | 0.037555242 |
| HIST2H2AB | 259.81 | 130 | 14 | 10.89 | 0.037573857 |
| OSTC | 39.12 | 149 | 16.8 | 9.13 | 0.037590617 |
| FNBP4 | 2.14 | 1017 | 110.2 | 4.74 | 0.037672853 |
| LRRC59 | 296.99 | 307 | 34.9 | 9.57 | 0.037712905 |
| A2M | 1.71 | 1474 | 163.2 | 6.46 | 0.037743832 |
| HS3ST6 | 0 | 342 | 37.2 | 10.78 | 0.037765795 |
| NADK2 | 2.3 | 279 | 31.7 | 6.68 | 0.037803035 |
| RAB2A | 111.87 | 212 | 23.5 | 6.54 | 0.038277887 |
| CCT4 | 552.17 | 539 | 57.9 | 7.83 | 0.03838242 |
| HNRNPK | 838.14 | 464 | 51 | 5.33 | 0.03878844 |
| SF1 | 78.05 | 548 | 59.7 | 9.5 | 0.038833855 |
| MYO1C | 213.41 | 1044 | 119.6 | 9.45 | 0.039509014 |
| TACC3 | 3.01 | 838 | 90.3 | 5.05 | 0.039764987 |
| AKR1B1 | 275.62 | 316 | 35.8 | 6.98 | 0.039910713 |
| SNRPD3 | 212.27 | 120 | 13.3 | 8.91 | 0.040154766 |
| LYRM7 | 6.55 | 104 | 11.9 | 9.66 | 0.040242769 |
| TIGAR | 10.89 | 270 | 30 | 7.69 | 0.040256428 |
| ORC5 | 9.26 | 324 | 37.4 | 6.46 | 0.040299972 |
| NUP188 | 29.29 | 1638 | 182.2 | 6.83 | 0.04046731 |
| KHSRP | 334.59 | 711 | 73.1 | 7.3 | 0.04062697 |
| ATP2B4 | 1.24 | 1170 | 129.4 | 7.45 | 0.04137649 |
| MORF4L2 | 43.26 | 288 | 32.3 | 9.72 | 0.041960228 |
| SNX6 | 38.97 | 406 | 46.6 | 6.16 | 0.042060904 |
| SDC1 | 19.91 | 310 | 32.4 | 4.63 | 0.042190837 |
| ZFP36L2 | 5.44 | 494 | 51 | 8.16 | 0.042527365 |
| APEH | 55.75 | 732 | 81.2 | 5.48 | 0.042586874 |
| AP1B1 | 89.77 | 939 | 103.5 | 5.16 | 0.04301684 |
| VPS29 | 79.31 | 186 | 20.9 | 7.05 | 0.043226843 |
| BRCA1 | 2.47 | 1863 | 207.7 | 5.29 | 0.04348795 |
| LIN7A | 16.93 | 233 | 26 | 8.72 | 0.043770325 |
| YRDC | 4.09 | 279 | 29.3 | 8.57 | 0.043986026 |
| MRPS18B | 16.35 | 258 | 29.4 | 9.38 | 0.044093838 |
| TSEN34 | 4.8 | 310 | 33.6 | 8.43 | 0.044128303 |
| ERP44 | 123.28 | 406 | 46.9 | 5.26 | 0.044179554 |
| TRIR | 14.4 | 176 | 18.4 | 9.44 | 0.04438448 |
| GPAT3 | 31.41 | 434 | 48.7 | 8.87 | 0.044388002 |
| SRRT | 104.21 | 871 | 100.1 | 6.01 | 0.044731904 |
| APMAP | 144.11 | 416 | 46.5 | 6.16 | 0.045013179 |
| NDUFC2 | 3.18 | 96 | 11.4 | 8.15 | 0.045122925 |
| PRIM1 | 10.5 | 420 | 49.9 | 8.21 | 0.045157571 |
| C9orf64 | 33.82 | 341 | 39 | 5.88 | 0.045169864 |
| MT-ND1 | 3.53 | 318 | 35.6 | 6.55 | 0.045372859 |
| TMEM11 | 5.25 | 192 | 21.5 | 7.36 | 0.045852154 |
| NDUFAF1 | 6.11 | 327 | 37.7 | 7.64 | 0.045852583 |
| FAM98A | 21.81 | 518 | 55.2 | 8.95 | 0.045977098 |
| NMT1 | 89.58 | 496 | 56.8 | 7.8 | 0.045992361 |
| SPART | 17.48 | 666 | 72.8 | 5.91 | 0.046267789 |
| HAUS5 | 0 | 290 | 32.6 | 7.56 | 0.046274086 |
| PNPT1 | 94.18 | 783 | 85.9 | 7.77 | 0.046275333 |
| TCIRG1 | 3.21 | 830 | 92.9 | 7.12 | 0.046290442 |
| GORASP2 | 24.18 | 384 | 39.7 | 4.63 | 0.046293292 |
| WDFY1 | 3.4 | 410 | 46.3 | 7.33 | 0.046990878 |
| ANXA11 | 166.59 | 472 | 51.2 | 7.66 | 0.047032527 |
| HLA-A | 129.46 | 365 | 40.9 | 6.7 | 0.047089142 |
| FOS | 19.5 | 380 | 40.7 | 4.77 | 0.047156846 |
| DYRK1A | 2.94 | 529 | 60.3 | 9.13 | 0.047202399 |
| NEFL | 81.78 | 543 | 61.5 | 4.65 | 0.047300346 |
| PDS5A | 51.81 | 1337 | 150.7 | 7.91 | 0.047321538 |
| WASHC2C | 7.2 | 1245 | 136.5 | 4.74 | 0.047432849 |
| RHEB | 78.49 | 184 | 20.5 | 5.92 | 0.047602249 |
| CUL1 | 36.25 | 776 | 89.6 | 8 | 0.047846593 |
| UQCC2 | 3.82 | 126 | 14.9 | 7.37 | 0.047995586 |
| OPA1 | 35.24 | 960 | 111.6 | 7.87 | 0.048124267 |
| MBOAT7 | 12.73 | 344 | 38.4 | 8.87 | 0.048182905 |
| IGFBP2 | 41.72 | 325 | 34.8 | 7.5 | 0.048227892 |
| TP53 | 4.03 | 393 | 43.7 | 6.33 | 0.04826479 |
| ZNF586 | 0 | 210 | 22.8 | 9.99 | 0.048357397 |
| MYBBP1A | 217.66 | 1328 | 148.8 | 9.28 | 0.048405728 |
| MRPS14 | 6.59 | 128 | 15.1 | 11.41 | 0.048612282 |
| NME2P1 | 341.86 | 137 | 15.5 | 8.57 | 0.04866753 |
| RBX1 | 2.72 | 108 | 12.3 | 6.96 | 0.048686687 |
| PKMYT1 | 5.54 | 430 | 47.3 | 6.06 | 0.048877909 |
| IMPDH2 | 393.53 | 514 | 55.8 | 6.9 | 0.048878947 |
| UACA | 5.17 | 1403 | 161.4 | 6.93 | 0.048906491 |
| KTN1 | 100.39 | 1357 | 156.2 | 5.64 | 0.049181782 |
| ALDH1B1 | 21.09 | 517 | 57.2 | 6.8 | 0.049384178 |
| CDS2 | 4.18 | 445 | 51.4 | 7.09 | 0.049790732 |
| PRNP | 9.27 | 246 | 26.9 | 9.11 | 0.049963946 |
| BZW1 | 247 | 419 | 48 | 5.92 | 0.049975633 |
| CD70 | 20.39 | 193 | 21.1 | 8.53 | 0.049998102 |
| CRAT | 13.05 | 544 | 61.8 | 8.94 | 0.050287233 |
| KIN | 2.43 | 374 | 43.2 | 8.9 | 0.050630578 |
| HSPG2 | 2.45 | 4391 | 468.5 | 6.51 | 0.051103801 |
| GPS1 | 39.76 | 487 | 55.1 | 6.61 | 0.051223985 |
| UCK2 | 52.49 | 261 | 29.3 | 6.7 | 0.051241302 |
| BCL2L13 | 18.4 | 485 | 52.7 | 4.44 | 0.051528983 |
| EIF3A | 404 | 1382 | 166.5 | 6.79 | 0.05173694 |
| SEH1L | 10.58 | 360 | 39.6 | 8.09 | 0.05196777 |
| EXOSC3 | 2.51 | 164 | 17.2 | 9.28 | 0.052072381 |
| FAM177A1 | 4.1 | 213 | 23.7 | 4.45 | 0.052289059 |
| EEF1D | 311.74 | 281 | 31.1 | 5.01 | 0.052378789 |
| GZMB | 0 | 247 | 27.7 | 9.57 | 0.052398481 |
| VCPIP1 | 3.13 | 1222 | 134.2 | 7.2 | 0.052423416 |
| MZT2B | 24.24 | 158 | 16.2 | 10.15 | 0.053358178 |
| PAIP1 | 10.51 | 367 | 42 | 4.51 | 0.053379966 |
| WTAP | 0 | 151 | 17.8 | 5.47 | 0.053410706 |
| CD63 | 12.54 | 156 | 17.3 | 7.88 | 0.053561749 |
| ADPGK | 29.99 | 496 | 53.9 | 6.2 | 0.053857731 |
| CNOT7 | 3.02 | 244 | 28.2 | 4.92 | 0.054071618 |
| CLIC1 | 354.2 | 241 | 26.9 | 5.17 | 0.054485478 |
| LMNB2 | 172.7 | 600 | 67.6 | 5.35 | 0.05474861 |
| CRNKL1 | 3.85 | 687 | 83.1 | 6.76 | 0.055059999 |
| BCKDK | 7.99 | 335 | 37.7 | 9.33 | 0.055227354 |
| TAX1BP3 | 2.83 | 124 | 13.7 | 8.48 | 0.055402998 |
| PRPF8 | 276.37 | 2335 | 273.4 | 8.84 | 0.055918857 |
| FLVCR1 | 2.56 | 555 | 59.8 | 5.97 | 0.055964637 |
| CCT5 | 640.4 | 541 | 59.6 | 5.66 | 0.056246395 |
| CNDP2 | 143.45 | 475 | 52.8 | 5.97 | 0.056300651 |
| EEF2K | 2.46 | 725 | 82.1 | 5.33 | 0.056345805 |
| RFC3 | 11.48 | 356 | 40.5 | 8.34 | 0.056630919 |
| WDR1 | 101.81 | 606 | 66.2 | 6.65 | 0.056986989 |
| PSMD8 | 64.97 | 350 | 39.6 | 9.7 | 0.057013421 |
| RPN2 | 223.31 | 631 | 69.2 | 5.69 | 0.057051746 |
| TMX4 | 36.66 | 349 | 38.9 | 4.37 | 0.057250051 |
| DBNL | 59.49 | 430 | 48.2 | 5.05 | 0.057260279 |
| S100A11 | 234.75 | 105 | 11.7 | 7.12 | 0.057435525 |
| LUC7L | 94.14 | 325 | 38.4 | 10.04 | 0.057730975 |
| AP3B1 | 99.11 | 1045 | 116.1 | 6.3 | 0.057783418 |
| DOCK5 | 2.09 | 1870 | 215.2 | 7.96 | 0.057823314 |
| ADAM9 | 10.42 | 819 | 90.5 | 7.52 | 0.058028447 |
| CSTF1 | 14.68 | 431 | 48.3 | 6.58 | 0.058410711 |
| MRPS30 | 0 | 439 | 50.3 | 7.97 | 0.058422969 |
| AGR2 | 581.83 | 175 | 20 | 9 | 0.05854093 |
| SOAT1 | 15.53 | 550 | 64.7 | 8.94 | 0.058805903 |
| BCKDHB | 2.06 | 218 | 23.5 | 8.1 | 0.05942501 |
| CLN8 | 0 | 286 | 32.8 | 8.21 | 0.059480133 |
| FLYWCH2 | 5.15 | 140 | 14.6 | 8.46 | 0.059617622 |
| STX4 | 12.28 | 297 | 34.2 | 6.28 | 0.059627948 |
| POLR2C | 12.22 | 275 | 31.4 | 4.92 | 0.059664385 |
| GNE | 8.73 | 648 | 71.2 | 6.81 | 0.060709459 |
| RPAP1 | 0 | 1315 | 143.5 | 6.47 | 0.0610711 |
| PWP1 | 6.26 | 501 | 55.8 | 4.77 | 0.061165687 |
| SLC9A3R1 | 42.84 | 358 | 38.8 | 5.77 | 0.061218819 |
| EIF3G | 120.87 | 320 | 35.6 | 6.13 | 0.061411257 |
| PDIA6 | 514.26 | 437 | 47.8 | 5.08 | 0.061744394 |
| PFN2 | 166.4 | 140 | 15.1 | 6.1 | 0.061896908 |
| SRBD1 | 2.1 | 620 | 69.7 | 8.81 | 0.062080208 |
| ATP5J | 86.81 | 108 | 12.6 | 9.52 | 0.062253594 |
| RPS6KA1 | 24.69 | 643 | 72.7 | 8.25 | 0.062350549 |
| CDKN2AIP | 2.22 | 580 | 61.1 | 9.01 | 0.062661747 |
| SHC1 | 71.28 | 473 | 51.6 | 7.18 | 0.062781875 |
| TLK1 | 7.11 | 549 | 64 | 8.48 | 0.062810381 |
| POLR2E | 24.36 | 210 | 24.5 | 5.95 | 0.063262084 |
| ATP5F1D | 63.77 | 168 | 17.5 | 5.49 | 0.063279569 |
| RABGAP1 | 0 | 599 | 67.1 | 6.04 | 0.063365224 |
| SNRPE | 76.34 | 92 | 10.8 | 9.44 | 0.063418353 |
| PPIG | 13.34 | 754 | 88.6 | 10.29 | 0.06342673 |
| PRKCSH | 228.83 | 525 | 59.1 | 4.42 | 0.063528443 |
| FKBP3 | 79.6 | 224 | 25.2 | 9.28 | 0.064039007 |
| CCDC115 | 4.92 | 180 | 19.7 | 6.95 | 0.064212302 |
| FAM49B | 17.18 | 324 | 36.7 | 6.06 | 0.064416464 |
| CTIF | 2.02 | 598 | 67.5 | 6.54 | 0.06441771 |
| NASP | 179.69 | 788 | 85.2 | 4.3 | 0.064448625 |
| SPTAN1 | 1777.11 | 2452 | 282.1 | 5.34 | 0.06454573 |
| RAB35 | 234.54 | 201 | 23 | 8.29 | 0.064624125 |
| PAG1 | 15.04 | 432 | 47 | 4.65 | 0.06486601 |
| BOLA2 | 120.85 | 86 | 10.1 | 6.52 | 0.065080733 |
| PNKP | 3.81 | 482 | 53 | 8.53 | 0.065325659 |
| PPP6C | 7.24 | 283 | 32.5 | 6.04 | 0.065442349 |
| URM1 | 17.18 | 63 | 7.1 | 5.34 | 0.065509214 |
| FAM192A | 13.21 | 254 | 28.9 | 5.45 | 0.065564692 |
| RGS10 | 3.76 | 167 | 19.6 | 5.87 | 0.06587549 |
| PRPF40A | 45.68 | 930 | 105.9 | 7.99 | 0.066007081 |
| CARM1 | 21.13 | 585 | 63.4 | 6.57 | 0.066385379 |
| COG7 | 8.21 | 770 | 86.3 | 5.47 | 0.066559743 |
| FNDC3A | 2.93 | 1142 | 125.7 | 7.08 | 0.066574411 |
| MNAT1 | 19.19 | 267 | 31.1 | 5.74 | 0.066788932 |
| UFM1 | 31.57 | 85 | 9.1 | 9.31 | 0.067065028 |
| SLC16A1 | 7.56 | 430 | 46.2 | 9.23 | 0.067099025 |
| DHX8 | 5 | 1220 | 139.2 | 8.32 | 0.067135929 |
| HEATR5A | 3.51 | 1965 | 213.9 | 6.52 | 0.06719375 |
| ACTC1 | 3704.14 | 377 | 42 | 5.39 | 0.067199214 |
| TUBB6 | 1163.28 | 446 | 49.8 | 4.88 | 0.067834284 |
| GPD1L | 2.68 | 351 | 38.4 | 7.02 | 0.067945861 |
| KHDRBS1 | 56.24 | 443 | 48.2 | 8.66 | 0.067986579 |
| PSMC4 | 97.53 | 387 | 43.5 | 5.26 | 0.067998755 |
| ATF7 | 3.13 | 307 | 33.7 | 7.18 | 0.068086123 |
| EPDR1 | 35.16 | 224 | 25.4 | 6.6 | 0.068394192 |
| CSRP2 | 3.43 | 193 | 20.9 | 8.62 | 0.068462421 |
| CLASP1 | 0 | 1471 | 162 | 8.81 | 0.06855682 |
| FXN | 2.48 | 171 | 19.1 | 8.84 | 0.06863806 |
| RPA1 | 105.37 | 616 | 68.1 | 7.21 | 0.068694405 |
| THG1L | 5.23 | 298 | 34.8 | 8 | 0.068867409 |
| D2HGDH | 2.44 | 521 | 56.4 | 7.99 | 0.069048598 |
| CARS2 | 2.56 | 564 | 62.2 | 8.34 | 0.069139381 |
| ASPH | 255.52 | 729 | 83.2 | 4.92 | 0.06916815 |
| GNG5 | 2.11 | 68 | 7.3 | 9.85 | 0.069247238 |
| HADHB | 127.21 | 474 | 51.3 | 9.41 | 0.069458736 |
| IFT27 | 3 | 185 | 20.4 | 5.41 | 0.069529874 |
| TTC39A | 0 | 221 | 25.1 | 7.91 | 0.069533533 |
| VCP | 715.49 | 806 | 89.3 | 5.26 | 0.069714484 |
| NCEH1 | 145.2 | 408 | 45.8 | 7.23 | 0.069724247 |
| RSPRY1 | 4.21 | 117 | 12.7 | 7.4 | 0.069786287 |
| PRPF6 | 64.64 | 941 | 106.9 | 8.25 | 0.069854516 |
| USP8 | 9.92 | 1012 | 115 | 8.46 | 0.069856608 |
| GSR | 359.42 | 479 | 51.7 | 7.71 | 0.070189161 |
| MRPS16 | 3.05 | 123 | 13.8 | 9.07 | 0.070409442 |
| MAP3K20 | 4.55 | 455 | 51.5 | 5.21 | 0.07066355 |
| USP11 | 9.8 | 963 | 109.7 | 5.45 | 0.070680205 |
| HDHD2 | 0 | 259 | 28.5 | 6.24 | 0.070784983 |
| DUS3L | 3.28 | 408 | 46.1 | 7.58 | 0.070804581 |
| C1orf123 | 17.28 | 160 | 18 | 5.01 | 0.070961426 |
| RPL30 | 335.93 | 115 | 12.8 | 9.63 | 0.071047691 |
| PAF1 | 26.88 | 485 | 55.4 | 5.36 | 0.071475623 |
| SFPQ | 487.73 | 707 | 76.1 | 9.44 | 0.071590128 |
| AP2S1 | 55.05 | 142 | 17 | 6.18 | 0.071736682 |
| ABCB6 | 33.17 | 796 | 88.5 | 8.69 | 0.072035362 |
| NAA10 | 13.02 | 220 | 24.8 | 5.52 | 0.07227591 |
| MISP | 3.19 | 679 | 75.3 | 6.83 | 0.072306372 |
| ENO3 | 955.46 | 391 | 42.2 | 7.97 | 0.072581602 |
| MYO1G | 0 | 230 | 26.1 | 8.84 | 0.072679897 |
| DHX33 | 2.3 | 534 | 60.4 | 8.34 | 0.072781648 |
| SLC4A1AP | 5 | 796 | 88.8 | 5.19 | 0.073059506 |
| COX7A2L | 14.36 | 114 | 12.6 | 9.42 | 0.073679044 |
| SPCS3 | 33.57 | 180 | 20.3 | 8.62 | 0.073681051 |
| SLITRK4 | 13.09 | 837 | 94.3 | 7.8 | 0.074096138 |
| CHST12 | 0 | 414 | 48.4 | 9.32 | 0.074116011 |
| LTBP4 | 5.24 | 1587 | 169.4 | 5.29 | 0.074201098 |
| TRIP12 | 68.48 | 1722 | 192 | 6.49 | 0.074239736 |
| SLC16A7 | 0 | 478 | 52.2 | 9.31 | 0.074368646 |
| ARL8A | 26.94 | 186 | 21.4 | 7.77 | 0.074382155 |
| FASTK | 2.43 | 522 | 58.1 | 9.86 | 0.074641731 |
| KPNA6 | 53.47 | 536 | 60 | 4.98 | 0.074673451 |
| RING1 | 2.22 | 406 | 42.4 | 5.62 | 0.075271398 |
| PIH1D1 | 6.08 | 290 | 32.3 | 5.14 | 0.075442442 |
| STXBP6 | 12.95 | 210 | 23.5 | 9.04 | 0.075577968 |
| PTP4A1 | 8.43 | 173 | 19.8 | 8.97 | 0.075653059 |
| UBE2Q1 | 0 | 422 | 46.1 | 5.1 | 0.075772981 |
| DDX19B | 36.46 | 448 | 50.5 | 6.14 | 0.075826371 |
| CLTB | 77.74 | 211 | 23.2 | 4.69 | 0.076113281 |
| NPTX1 | 0 | 432 | 47.1 | 6.55 | 0.076281222 |
| EPB41L5 | 2.37 | 505 | 57.8 | 9.1 | 0.076366026 |
| PUS7 | 15.93 | 661 | 75 | 6.37 | 0.076432527 |
| PER2 | 2.4 | 404 | 45.1 | 6.29 | 0.076604645 |
| GPR108 | 2.44 | 543 | 60.6 | 8.69 | 0.076919561 |
| SNRPD1 | 27.2 | 119 | 13.3 | 11.56 | 0.077006542 |
| SUCLG1 | 73.02 | 346 | 36.2 | 8.79 | 0.077375983 |
| RRP15 | 18.11 | 282 | 31.5 | 5.52 | 0.077450065 |
| POLE4 | 24.77 | 117 | 12.2 | 4.92 | 0.077851577 |
| TRIM47 | 17.08 | 638 | 69.5 | 6.44 | 0.077976344 |
| MRM3 | 12.21 | 420 | 47 | 8.73 | 0.077979545 |
| DUSP12 | 2.48 | 340 | 37.7 | 6.84 | 0.077997534 |
| WRAP53 | 3.8 | 548 | 59.3 | 4.58 | 0.078682003 |
| ACBD3 | 24.41 | 528 | 60.6 | 5.06 | 0.078796596 |
| TFB2M | 3.3 | 396 | 45.3 | 9.19 | 0.079452258 |
| PAPSS1 | 53.47 | 624 | 70.8 | 6.86 | 0.079722341 |
| ALCAM | 18.64 | 570 | 63.6 | 7.3 | 0.079982912 |
| MRPL50 | 17.19 | 158 | 18.3 | 7.88 | 0.080444285 |
| GNL2 | 28.11 | 731 | 83.6 | 9.25 | 0.080473122 |
| NCAPG2 | 5.04 | 1143 | 130.9 | 6.87 | 0.081172435 |
| RSF1 | 6.32 | 1410 | 160 | 5.01 | 0.081234762 |
| DDX3X | 603.66 | 662 | 73.2 | 7.18 | 0.081530876 |
| FKBP1A | 44.31 | 108 | 11.9 | 8.16 | 0.081532067 |
| HNRNPUL2 | 40.47 | 747 | 85.1 | 4.91 | 0.081747726 |
| TTC4 | 14.23 | 387 | 44.7 | 5.6 | 0.081866278 |
| CTSZ | 53.42 | 303 | 33.8 | 7.11 | 0.081950172 |
| NUP133 | 31.63 | 1156 | 128.9 | 5.1 | 0.082007054 |
| ANXA3 | 104.42 | 323 | 36.4 | 5.92 | 0.082017682 |
| SLC25A13 | 105.55 | 675 | 74.1 | 8.62 | 0.082088609 |
| C1orf122 | 6.12 | 110 | 11.5 | 6.73 | 0.082477287 |
| PLEC | 1788.14 | 4570 | 517.7 | 5.74 | 0.08270128 |
| CCDC22 | 1.61 | 627 | 70.7 | 6.74 | 0.082736315 |
| NIPSNAP3A | 11.31 | 247 | 28.4 | 9.16 | 0.082865809 |
| MIPEP | 10.12 | 713 | 80.6 | 7.05 | 0.083065428 |
| FRYL | 3.25 | 403 | 45.6 | 4.5 | 0.083306578 |
| ERAL1 | 4.36 | 276 | 30 | 9.28 | 0.083547397 |
| ITGAV | 58.38 | 1002 | 111.1 | 5.62 | 0.083972639 |
| SMARCA4 | 12.14 | 1613 | 181.1 | 7.99 | 0.084100375 |
| OSBP | 18.02 | 807 | 89.4 | 7.3 | 0.084377778 |
| RAVER1 | 15.23 | 739 | 77.8 | 8.84 | 0.084404508 |
| NUDT3 | 4.96 | 172 | 19.5 | 6.34 | 0.084717103 |
| SCAMP4 | 1.87 | 195 | 22 | 9.13 | 0.084930011 |
| MAP2K6 | 5.07 | 334 | 37.5 | 7.39 | 0.084936664 |
| DNAJC8 | 42.98 | 253 | 29.8 | 9.06 | 0.085157153 |
| ARL2BP | 11.13 | 152 | 17.7 | 4.5 | 0.085224478 |
| AGL | 0 | 1515 | 172.5 | 6.71 | 0.085488119 |
| RAB3C | 167.79 | 227 | 25.9 | 5.24 | 0.085530801 |
| TAF9B | 7.09 | 251 | 27.6 | 9.55 | 0.085773486 |
| GALK2 | 5.27 | 447 | 49.2 | 6.37 | 0.085994882 |
| ESD | 50.69 | 282 | 31.4 | 7.02 | 0.086015394 |
| YY1 | 21.56 | 414 | 44.7 | 6.25 | 0.086812017 |
| SRSF5 | 88.71 | 272 | 31.2 | 11.59 | 0.0869296 |
| PABPN1 | 53.68 | 296 | 31.5 | 4.96 | 0.086958187 |
| TOR1A | 6.18 | 332 | 37.8 | 6.99 | 0.08715318 |
| MYO1B | 12.8 | 1078 | 124.9 | 9.2 | 0.08739824 |
| DPP10 | 0 | 746 | 85.4 | 6.27 | 0.08770952 |
| SAE1 | 67.79 | 346 | 38.4 | 5.3 | 0.088033215 |
| H2AFY | 179.38 | 371 | 39.5 | 9.77 | 0.088125478 |
| CS | 439.22 | 466 | 51.7 | 8.32 | 0.088557586 |
| DLG3 | 10.44 | 366 | 42.1 | 8.59 | 0.08863533 |
| POLR3A | 5.33 | 1390 | 155.5 | 8.48 | 0.088702922 |
| TMX2 | 11.91 | 258 | 29.6 | 8.65 | 0.089225657 |
| ARL6IP5 | 34.69 | 188 | 21.6 | 9.77 | 0.089335465 |
| HDHD5 | 34.53 | 393 | 43.6 | 6.9 | 0.090235818 |
| HBS1L | 3.16 | 642 | 70.6 | 7.17 | 0.090561793 |
| ITPR3 | 19.73 | 2671 | 303.9 | 6.48 | 0.090653487 |
| GPD2 | 205.45 | 727 | 80.8 | 7.69 | 0.090733761 |
| RIOX1 | 6.68 | 641 | 71 | 6.46 | 0.091005297 |
| PRPF38A | 8.85 | 312 | 37.5 | 9.96 | 0.091183637 |
| EEF1E1 | 24.38 | 139 | 15.5 | 8.34 | 0.091250083 |
| CNP | 41.47 | 401 | 45.1 | 8.53 | 0.092232257 |
| GNAS | 72.53 | 379 | 44.2 | 6.29 | 0.092304453 |
| ALDH18A1 | 200.96 | 793 | 87 | 7.12 | 0.092529909 |
| HADHA | 403.72 | 763 | 82.9 | 9.04 | 0.092767784 |
| PHAX | 8.51 | 394 | 44.4 | 5.4 | 0.093412273 |
| B2M | 28.1 | 119 | 13.7 | 6.52 | 0.093456133 |
| RTN4IP1 | 7.07 | 225 | 24.9 | 9.58 | 0.094268756 |
| TCEAL4 | 22.66 | 215 | 24.6 | 5.2 | 0.094397128 |
| SLC25A20 | 2.95 | 301 | 32.9 | 9.41 | 0.094549029 |
| APOA1 | 11.44 | 267 | 30.8 | 5.76 | 0.094583319 |
| HDAC1 | 26.75 | 482 | 55.1 | 5.48 | 0.094789559 |
| HACD3 | 58.78 | 362 | 43.1 | 8.94 | 0.095111136 |
| SYPL1 | 3.84 | 241 | 26.4 | 7.23 | 0.095192972 |
| HDLBP | 279.75 | 1268 | 141.4 | 6.87 | 0.09541895 |
| DRG2 | 20.62 | 364 | 40.7 | 8.88 | 0.095645079 |
| TBL2 | 27.36 | 447 | 49.8 | 9.44 | 0.096340702 |
| TYMP | 15.4 | 482 | 49.9 | 5.53 | 0.096402702 |
| FIP1L1 | 7.83 | 378 | 40.8 | 4.69 | 0.096477183 |
| CBL | 2.22 | 906 | 99.6 | 6.54 | 0.096885062 |
| KIF1BP | 12.2 | 621 | 71.8 | 5.49 | 0.097087516 |
| CKB | 381.99 | 381 | 42.6 | 5.59 | 0.097667172 |
| UQCRQ | 19.44 | 82 | 9.9 | 10.08 | 0.097984872 |
| NCK1 | 2.2 | 313 | 35.5 | 5.72 | 0.098055095 |
| SMN1 | 44.45 | 250 | 27.1 | 5.25 | 0.098145179 |
| NOLC1 | 298.73 | 699 | 73.6 | 9.47 | 0.09821461 |
| UBE2L6 | 0 | 87 | 10.1 | 7.09 | 0.098374191 |
| DCTD | 5.41 | 178 | 20 | 7.56 | 0.099510759 |
| TARS | 370.31 | 723 | 83.4 | 6.67 | 0.100068545 |
| SEPT5 | 3.44 | 369 | 42.7 | 6.67 | 0.100219567 |
| NRCAM | 6 | 1180 | 130.6 | 6.04 | 0.100312666 |
| DDX54 | 30.86 | 881 | 98.5 | 10.02 | 0.100344264 |
| COX6B1 | 79.91 | 86 | 10.2 | 7.05 | 0.100376933 |
| ORC4 | 2.59 | 352 | 40.8 | 6.54 | 0.100946838 |
| ELL | 3.1 | 621 | 68.2 | 9.33 | 0.101151612 |
| COX4I1 | 196.21 | 169 | 19.6 | 9.51 | 0.101542776 |
| GNB2 | 149.85 | 340 | 37.3 | 6 | 0.101740416 |
| ERP29 | 98.84 | 261 | 29 | 7.31 | 0.102109458 |
| B4GAT1 | 2.79 | 415 | 47.1 | 7.2 | 0.10219831 |
| SPTLC2 | 9.17 | 562 | 62.9 | 7.78 | 0.10232359 |
| CNOT11 | 2.26 | 510 | 55.2 | 6.4 | 0.102658467 |
| LNPEP | 14.35 | 1006 | 115 | 5.78 | 0.102862656 |
| AP4E1 | 2.71 | 1062 | 119.4 | 5.85 | 0.103180975 |
| AIFM2 | 42.96 | 373 | 40.5 | 9.11 | 0.103532143 |
| ECD | 3.57 | 601 | 67.7 | 4.75 | 0.103558602 |
| RBM19 | 2.92 | 960 | 107.3 | 6.54 | 0.104336092 |
| PSMC5 | 237.48 | 398 | 44.8 | 8.18 | 0.104613638 |
| DNAJC2 | 15.43 | 621 | 72 | 8.7 | 0.104618939 |
| G6PC3 | 8.24 | 346 | 38.7 | 8.21 | 0.105255371 |
| RPL35A | 123.69 | 110 | 12.5 | 11.06 | 0.105319379 |
| ANKRD17 | 36.56 | 2352 | 246.6 | 6.68 | 0.105408612 |
| ATP6V1G1 | 4.61 | 118 | 13.7 | 8.79 | 0.105775865 |
| URI1 | 3.49 | 459 | 51.2 | 5.01 | 0.10602774 |
| MAGEA1 | 14.39 | 309 | 34.3 | 4.86 | 0.10629506 |
| MRPL17 | 12.32 | 175 | 20 | 10.11 | 0.106599467 |
| AARSD1 | 17.75 | 412 | 45.5 | 6.42 | 0.106769637 |
| NUP107 | 24.38 | 925 | 106.3 | 5.43 | 0.106860096 |
| ATP5H | 98.09 | 161 | 18.5 | 5.3 | 0.107442444 |
| ALG5 | 2.93 | 324 | 36.9 | 9.28 | 0.107902026 |
| THEM4 | 2.46 | 240 | 27.1 | 8.28 | 0.108925973 |
| NHSL1 | 6.06 | 1606 | 170.4 | 7.55 | 0.109188243 |
| BCL9 | 0 | 1426 | 149.2 | 8.91 | 0.109738687 |
| SPG7 | 7.78 | 795 | 88.2 | 8.69 | 0.109813975 |
| EXOC6 | 7.97 | 799 | 93.3 | 6.04 | 0.110071072 |
| UBR1 | 2.78 | 803 | 93.1 | 5.59 | 0.110124072 |
| MAP7D1 | 2.9 | 377 | 42.1 | 9.2 | 0.110232085 |
| ANO2 | 0 | 999 | 113.5 | 6.42 | 0.110666516 |
| CTNNB1 | 85.11 | 781 | 85.4 | 5.86 | 0.11093869 |
| RPL35 | 36.68 | 123 | 14.5 | 11.05 | 0.111029991 |
| XPO7 | 15.32 | 1087 | 123.8 | 6.32 | 0.11133187 |
| DNM1L | 27.43 | 710 | 79.4 | 6.93 | 0.111471507 |
| OCRL | 5.08 | 893 | 103.2 | 6.77 | 0.111524393 |
| FSCN1 | 351.93 | 493 | 54.5 | 7.24 | 0.11179352 |
| TTLL12 | 101.01 | 644 | 74.4 | 5.53 | 0.111872879 |
| WDR48 | 6.14 | 668 | 75.3 | 7.15 | 0.111939794 |
| BTF3 | 96.95 | 162 | 17.7 | 7.5 | 0.111955727 |
| VMP1 | 9.19 | 406 | 46.2 | 6.95 | 0.1125279 |
| SARNP | 19.1 | 210 | 23.7 | 6.42 | 0.113011563 |
| TXNDC17 | 143.35 | 123 | 13.9 | 5.52 | 0.113082692 |
| EGFR | 56.45 | 1210 | 134.2 | 6.68 | 0.11327394 |
| PTPN1 | 9.07 | 435 | 49.9 | 6.27 | 0.11336246 |
| NDUFA13 | 32.91 | 144 | 16.7 | 8.43 | 0.113539818 |
| XRCC1 | 3.09 | 633 | 69.4 | 6.39 | 0.114253419 |
| LAMTOR3 | 2.86 | 117 | 12.9 | 8.13 | 0.11478044 |
| HLA-G | 45.82 | 338 | 38.2 | 5.69 | 0.114968138 |
| ATG12 | 3.35 | 140 | 15.1 | 5.1 | 0.115230468 |
| ZYX | 133.15 | 572 | 61.2 | 6.67 | 0.115928052 |
| MCU | 22.47 | 302 | 35.1 | 7.49 | 0.115952579 |
| RANGAP1 | 93.97 | 587 | 63.5 | 4.68 | 0.115991755 |
| L1CAM | 9.1 | 1248 | 138.8 | 6.21 | 0.116038675 |
| DCAF11 | 2.99 | 446 | 50.6 | 7.12 | 0.116437207 |
| AP2M1 | 35.71 | 433 | 49.4 | 9.54 | 0.116488666 |
| IKBKB | 2.19 | 697 | 79.5 | 5.5 | 0.11651244 |
| PDLIM5 | 357.55 | 596 | 63.9 | 8.21 | 0.116696809 |
| SYNCRIP | 437.23 | 623 | 69.6 | 8.59 | 0.117364835 |
| TRADD | 4.32 | 252 | 27.9 | 7.68 | 0.117785817 |
| ARIH2 | 0 | 493 | 57.8 | 5.63 | 0.117922635 |
| PLCG1 | 6.16 | 1290 | 148.4 | 6.05 | 0.118168583 |
| ANKRD61 | 1.99 | 418 | 46.1 | 8.85 | 0.118194321 |
| SEC62 | 3.2 | 399 | 45.8 | 7.12 | 0.118515855 |
| MICU1 | 3.78 | 403 | 45.9 | 9 | 0.118720887 |
| MAPRE2 | 4.57 | 274 | 30.7 | 5.81 | 0.118759241 |
| LIMA1 | 16.34 | 599 | 67.1 | 5.88 | 0.118875945 |
| HSPA1L | 1301.12 | 641 | 70.3 | 6.02 | 0.119538405 |
| ARF5 | 287.34 | 180 | 20.5 | 6.79 | 0.119766052 |
| NAPA | 20.44 | 295 | 33.2 | 5.36 | 0.119836047 |
| RSL1D1 | 90.63 | 490 | 54.9 | 10.13 | 0.120511813 |
| SRFBP1 | 2.79 | 429 | 48.6 | 9.58 | 0.120567262 |
| SUCLA2 | 38.59 | 441 | 48 | 7.08 | 0.120817003 |
| L3HYPDH | 5.99 | 354 | 38.1 | 6.68 | 0.120933302 |
| ATP5J2 | 64.68 | 49 | 5.7 | 9.7 | 0.120972121 |
| HMGN3 | 4.41 | 99 | 10.7 | 9.66 | 0.121031643 |
| EIF3E | 156.02 | 445 | 52.2 | 6.04 | 0.121317177 |
| PFDN4 | 18.21 | 134 | 15.3 | 4.53 | 0.121407343 |
| MRPL19 | 22.81 | 292 | 33.5 | 9.5 | 0.121544587 |
| BID | 16.83 | 195 | 22 | 5.44 | 0.122540147 |
| SGPL1 | 7.23 | 568 | 63.5 | 9.16 | 0.123013762 |
| RPL37A | 73.05 | 92 | 10.3 | 10.43 | 0.123276297 |
| TSG101 | 2.8 | 285 | 31.7 | 5.33 | 0.123453404 |
| PPP1R9B | 15.48 | 815 | 89.1 | 4.97 | 0.123534451 |
| WRB | 2.68 | 140 | 16 | 9.79 | 0.123971521 |
| MYO18B | 0 | 2567 | 285 | 6.9 | 0.124026658 |
| RPL36A | 83.34 | 106 | 12.4 | 10.58 | 0.124263263 |
| MAP2K1 | 45.51 | 393 | 43.4 | 6.62 | 0.124333277 |
| ATP6AP1 | 12.1 | 470 | 52 | 6.14 | 0.124706824 |
| FAM129B | 58.85 | 733 | 82.6 | 6.15 | 0.124895868 |
| FKBP7 | 3.61 | 221 | 25.7 | 6.32 | 0.125144093 |
| EXOC2 | 8.44 | 924 | 104 | 6.9 | 0.125505869 |
| TELO2 | 12.27 | 837 | 91.7 | 5.76 | 0.126171719 |
| TXNDC5 | 331.23 | 432 | 47.6 | 5.97 | 0.126242438 |
| TRAPPC5 | 8.81 | 188 | 20.8 | 9.66 | 0.1265836 |
| LDAH | 9.31 | 195 | 22.6 | 5.9 | 0.127174537 |
| OGFR | 36.65 | 657 | 71.4 | 4.88 | 0.127302571 |
| GADD45GIP1 | 21.08 | 222 | 25.4 | 10.02 | 0.127428329 |
| AIP | 54.12 | 330 | 37.6 | 6.29 | 0.127982821 |
| ZMIZ2 | 2.93 | 862 | 91.1 | 7.23 | 0.128084191 |
| BSG | 72.14 | 269 | 29.2 | 5.64 | 0.128215697 |
| TAF15 | 131.14 | 589 | 61.5 | 8.02 | 0.128538658 |
| EIF3M | 105.85 | 374 | 42.5 | 5.63 | 0.128729253 |
| CIT | 2.86 | 1544 | 176.9 | 6.58 | 0.129007148 |
| BUB3 | 130.97 | 326 | 36.9 | 6.84 | 0.13000149 |
| PTPN11 | 60.55 | 593 | 68 | 7.3 | 0.130041188 |
| LAMA1 | 3.13 | 3075 | 336.9 | 6.35 | 0.130058544 |
| CLCN7 | 2.51 | 781 | 86 | 8.73 | 0.130097877 |
| PDE4D | 2.12 | 507 | 57.8 | 4.86 | 0.13053293 |
| SLC35B2 | 2.48 | 339 | 37.2 | 9.6 | 0.13060266 |
| PRMT5 | 80.98 | 637 | 72.6 | 6.29 | 0.130867451 |
| UTP20 | 56.6 | 2785 | 318.2 | 7.39 | 0.131933898 |
| ZBTB38 | 0 | 1195 | 134.2 | 8.03 | 0.132303235 |
| NSUN2 | 114.41 | 767 | 86.4 | 6.77 | 0.132912592 |
| UBQLN1 | 143.48 | 589 | 62.5 | 5.11 | 0.132934551 |
| RAP1GDS1 | 34.08 | 607 | 66.3 | 5.31 | 0.133075514 |
| NUCB2 | 22.05 | 420 | 50.2 | 5.12 | 0.133672964 |
| UBAC1 | 3.64 | 405 | 45.3 | 4.92 | 0.13396275 |
| CDC23 | 7.56 | 479 | 55.8 | 7.42 | 0.134173729 |
| FLAD1 | 15.66 | 446 | 49.2 | 5.96 | 0.134547452 |
| CYCS | 305.62 | 105 | 11.7 | 9.57 | 0.134553975 |
| PCBP1 | 719.29 | 356 | 37.5 | 7.09 | 0.13477513 |
| PCBP2 | 549.22 | 335 | 35.3 | 8 | 0.135111213 |
| ADGRG6 | 0 | 1193 | 133.6 | 7.74 | 0.13518459 |
| RNF14 | 1.8 | 348 | 39.6 | 4.88 | 0.135500343 |
| RPL12 | 467.72 | 165 | 17.8 | 9.42 | 0.135576144 |
| POLR3C | 5.9 | 534 | 60.6 | 7.31 | 0.13576967 |
| EXOSC1 | 15.39 | 195 | 21.4 | 8.24 | 0.135797216 |
| STAM2 | 8 | 525 | 58.1 | 5.07 | 0.136220319 |
| SPP1 | 18.73 | 292 | 33 | 4.74 | 0.136459655 |
| ANXA5 | 447.05 | 320 | 35.9 | 5.05 | 0.136611874 |
| MRI1 | 7.16 | 369 | 39.1 | 6.3 | 0.136733537 |
| DUT | 252.69 | 164 | 17.7 | 6.57 | 0.136977371 |
| SRSF4 | 99.33 | 494 | 56.6 | 11.52 | 0.1373134 |
| MIB1 | 0 | 1006 | 110.1 | 6.92 | 0.137494155 |
| PRMT6 | 2.41 | 375 | 41.9 | 5.44 | 0.137649709 |
| MRPS31 | 16.54 | 395 | 45.3 | 9.29 | 0.137677535 |
| FTSJ1 | 2.38 | 327 | 35.8 | 5.88 | 0.137756521 |
| RAC1 | 167.23 | 192 | 21.4 | 8.5 | 0.138102687 |
| ERCC4 | 0 | 916 | 104.4 | 6.93 | 0.138162796 |
| RER1 | 15.05 | 196 | 22.9 | 9.54 | 0.138251394 |
| TK1 | 24.67 | 234 | 25.5 | 8.51 | 0.138412296 |
| HSDL2 | 33.46 | 418 | 45.4 | 7.99 | 0.13857414 |
| AHSG | 51.03 | 367 | 39.3 | 5.72 | 0.138755899 |
| NDUFB10 | 33.18 | 172 | 20.8 | 8.48 | 0.139298688 |
| GCC2 | 9.24 | 1684 | 195.8 | 5.14 | 0.139764572 |
| GRWD1 | 24.25 | 446 | 49.4 | 4.92 | 0.140271388 |
| SMCHD1 | 17 | 2005 | 226.2 | 7.3 | 0.140286179 |
| MYH10 | 228.31 | 1976 | 228.9 | 5.54 | 0.140364084 |
| CARD10 | 2.02 | 1032 | 115.9 | 5.95 | 0.141146751 |
| NDUFB11 | 6.6 | 153 | 17.3 | 5.22 | 0.142205332 |
| MORF4L1 | 4.74 | 235 | 26.7 | 9.19 | 0.142701419 |
| ARF6 | 70.8 | 175 | 20.1 | 8.95 | 0.143228752 |
| CPSF1 | 4.91 | 1443 | 160.8 | 6.4 | 0.143248802 |
| PYGB | 836.93 | 843 | 96.6 | 6.86 | 0.143334671 |
| MTERF3 | 2.21 | 296 | 34.3 | 8.44 | 0.143703755 |
| RNF126 | 2.12 | 311 | 33.8 | 5.47 | 0.143925675 |
| ARRB2 | 4.85 | 394 | 44.4 | 7.88 | 0.14404388 |
| IDH2 | 76.34 | 452 | 50.9 | 8.69 | 0.144417439 |
| P3H1 | 33.16 | 736 | 83.3 | 5.14 | 0.144433244 |
| MARCKSL1 | 19.58 | 195 | 19.5 | 4.67 | 0.145094158 |
| LLGL2 | 2.86 | 356 | 39.6 | 7.59 | 0.145569714 |
| COMMD8 | 22.56 | 183 | 21.1 | 5.43 | 0.145760647 |
| MAP2K3 | 43.65 | 318 | 36.1 | 6.25 | 0.145770666 |
| NEK7 | 29.46 | 302 | 34.5 | 8.25 | 0.146056273 |
| HBD | 24.62 | 147 | 16 | 8.05 | 0.146326172 |
| RCC2 | 101.04 | 522 | 56 | 8.78 | 0.146553937 |
| SOWAHC | 16.38 | 525 | 55.6 | 7.03 | 0.146577153 |
| CD55 | 151.93 | 364 | 39.7 | 7.61 | 0.146645341 |
| NACC2 | 3.64 | 587 | 62.8 | 5.9 | 0.146739939 |
| MON2 | 3.33 | 1675 | 185.7 | 6.18 | 0.146883115 |
| CUEDC2 | 3.01 | 287 | 32 | 4.81 | 0.146997079 |
| PIBF1 | 0 | 757 | 89.7 | 6.02 | 0.14798712 |
| PRPS2 | 179.95 | 318 | 34.7 | 6.61 | 0.147995936 |
| SEC24B | 19.24 | 1233 | 133.5 | 7.39 | 0.148094005 |
| PRRC1 | 32.59 | 445 | 46.7 | 5.83 | 0.148401889 |
| TWF2 | 42.53 | 349 | 39.5 | 6.84 | 0.148512974 |
| EPB41L3 | 413.39 | 1087 | 120.6 | 5.19 | 0.148558052 |
| NUP35 | 5.8 | 191 | 21.1 | 8.97 | 0.149035822 |
| SMAP | 30.83 | 183 | 20.3 | 4.72 | 0.149090089 |
| KIF11 | 11.02 | 1056 | 119.1 | 5.64 | 0.149941864 |
| NOL6 | 35.92 | 1143 | 127.2 | 7.64 | 0.150333344 |
| PSME1 | 196.76 | 249 | 28.7 | 6.02 | 0.150458098 |
| ALDH3A2 | 88.68 | 485 | 54.8 | 7.88 | 0.150867965 |
| IDH3A | 134.85 | 366 | 39.6 | 6.92 | 0.150891597 |
| LEO1 | 2.24 | 666 | 75.4 | 4.51 | 0.151628666 |
| NDUFV2 | 36.23 | 249 | 27.4 | 8.06 | 0.151630878 |
| PTPN12 | 21.32 | 780 | 88.1 | 5.62 | 0.15227218 |
| DIS3L2 | 4.97 | 885 | 99.2 | 6.1 | 0.152710023 |
| GRHPR | 61.24 | 328 | 35.6 | 7.39 | 0.152979307 |
| BTF3 | 112.5 | 206 | 22.2 | 9.38 | 0.153238683 |
| NUB1 | 7.77 | 601 | 69.1 | 5.97 | 0.153368307 |
| FRG1 | 3.93 | 258 | 29.2 | 9.01 | 0.153750086 |
| OSGEP | 7.01 | 335 | 36.4 | 6.35 | 0.153938759 |
| AATF | 22.3 | 560 | 63.1 | 4.94 | 0.154054163 |
| TFB1M | 13.23 | 346 | 39.5 | 9.26 | 0.154450382 |
| RPS13 | 295.63 | 151 | 17.2 | 10.54 | 0.154573236 |
| POLR2K | 8.92 | 58 | 7 | 9.06 | 0.155079627 |
| TBCC | 2.65 | 346 | 39.2 | 5.71 | 0.155084582 |
| SNRPA1 | 65.4 | 255 | 28.4 | 8.62 | 0.155146487 |
| MT-CO2 | 114.45 | 227 | 25.5 | 4.82 | 0.155250107 |
| RSU1 | 10.65 | 224 | 25.5 | 8.72 | 0.155298387 |
| CDC27 | 3.11 | 824 | 91.8 | 7.02 | 0.155404753 |
| ATRAID | 2.03 | 171 | 18.6 | 7.25 | 0.155543521 |
| SCCPDH | 152.47 | 429 | 47.1 | 9.14 | 0.155758821 |
| PRCP | 9.48 | 496 | 55.8 | 7.21 | 0.156404514 |
| SERPINB10 | 0 | 397 | 45.4 | 6.16 | 0.156446357 |
| SHTN1 | 104.9 | 631 | 71.6 | 5.33 | 0.156753744 |
| USP5 | 114.1 | 835 | 93.2 | 5.08 | 0.157089664 |
| NEDD4 | 19.17 | 900 | 104.2 | 5.97 | 0.157950012 |
| SYNJ2BP | 21.98 | 145 | 15.9 | 6.3 | 0.159102273 |
| MOB1B | 24.16 | 216 | 25.1 | 6.73 | 0.159107808 |
| COPG2 | 68.35 | 726 | 81.4 | 6.58 | 0.159184154 |
| NENF | 39.13 | 172 | 18.8 | 5.69 | 0.159384779 |
| ADH5 | 62.68 | 374 | 39.7 | 7.49 | 0.159586022 |
| MAP7D3 | 5.35 | 835 | 93.7 | 9.28 | 0.159921024 |
| DHODH | 3.68 | 395 | 42.8 | 9.67 | 0.16035378 |
| ATOX1 | 27.65 | 68 | 7.4 | 7.24 | 0.160435283 |
| PREPL | 21.15 | 661 | 76.3 | 6.2 | 0.16048543 |
| INTS1 | 5.16 | 2190 | 244.1 | 6.13 | 0.160681865 |
| TMA7 | 10.78 | 64 | 7.1 | 9.99 | 0.160838622 |
| NFKB1 | 3.08 | 788 | 85.5 | 5.08 | 0.16085525 |
| RFTN1 | 0 | 578 | 63.1 | 5.67 | 0.161305996 |
| MAP1B | 391.38 | 2468 | 270.5 | 4.81 | 0.161370068 |
| SRP68 | 89.06 | 589 | 66.1 | 7.2 | 0.161487799 |
| IMP4 | 18.34 | 291 | 33.7 | 9.47 | 0.161520606 |
| NOB1 | 6.58 | 412 | 46.6 | 7.18 | 0.161610942 |
| SGMS1 | 2.37 | 419 | 49.2 | 8.4 | 0.161828366 |
| NMRAL1 | 2.71 | 299 | 33.3 | 7.52 | 0.16191714 |
| SH3GLB1 | 8.26 | 265 | 29.3 | 6.37 | 0.162289013 |
| COX5B | 116.57 | 129 | 13.7 | 8.81 | 0.162755781 |
| SPG21 | 0 | 281 | 31.6 | 6.43 | 0.163422524 |
| TOMM34 | 58.03 | 309 | 34.5 | 8.98 | 0.163476106 |
| SNRPA | 116.65 | 282 | 31.3 | 9.83 | 0.164000748 |
| SMIM20 | 2.25 | 67 | 7.7 | 9.86 | 0.164021483 |
| CHAC2 | 7.57 | 184 | 20.9 | 5.43 | 0.164233024 |
| INF2 | 167.73 | 1240 | 134.5 | 5.53 | 0.164427424 |
| IDE | 85.36 | 1019 | 117.9 | 6.61 | 0.164885994 |
| PELO | 5.73 | 385 | 43.3 | 6.34 | 0.165031899 |
| CDKN1A | 3.23 | 164 | 18.1 | 8.37 | 0.165370317 |
| BRI3BP | 29.42 | 251 | 27.8 | 9.44 | 0.165391937 |
| POP7 | 4.39 | 140 | 15.6 | 8.94 | 0.165747525 |
| PTEN | 3.12 | 403 | 47.1 | 6.37 | 0.165811369 |
| DIXDC1 | 2.14 | 218 | 24.3 | 9.67 | 0.166186098 |
| ZCCHC8 | 8.23 | 469 | 51.1 | 4.88 | 0.166935374 |
| ANK3 | 35.96 | 1861 | 202.5 | 6.68 | 0.167957187 |
| INTS14 | 6.45 | 439 | 48.4 | 5.11 | 0.167977353 |
| RABGGTB | 8.59 | 331 | 36.9 | 5.03 | 0.168733542 |
| ATP5F1C | 149.68 | 298 | 33 | 9.22 | 0.16881667 |
| ETFDH | 18.72 | 570 | 62.8 | 6.87 | 0.169259039 |
| SEMA6D | 2.05 | 998 | 111.7 | 8.66 | 0.16949493 |
| RPL10A | 212.76 | 217 | 24.8 | 9.94 | 0.169703817 |
| PHPT1 | 19.81 | 125 | 13.8 | 6.07 | 0.169705792 |
| THRAP3 | 90.06 | 955 | 108.6 | 10.15 | 0.169957394 |
| USP28 | 2.06 | 1045 | 119 | 5.16 | 0.170096579 |
| MTX2 | 12.24 | 253 | 28.8 | 6.54 | 0.170402778 |
| FAHD2A | 12.87 | 314 | 34.6 | 8.24 | 0.170640917 |
| RIOX2 | 5.81 | 211 | 23.9 | 5.22 | 0.170721447 |
| SLC51A | 0 | 340 | 37.7 | 8.54 | 0.17073279 |
| NUP37 | 16.95 | 326 | 36.7 | 5.92 | 0.171709141 |
| WDR12 | 26.37 | 423 | 47.7 | 5.9 | 0.17219674 |
| IMUP | 2.47 | 85 | 8.5 | 10.59 | 0.172536686 |
| PLOD1 | 29.52 | 727 | 83.5 | 6.95 | 0.172595568 |
| JAGN1 | 3.22 | 183 | 21.1 | 9.73 | 0.173466252 |
| TRIP4 | 0 | 581 | 66.1 | 7.85 | 0.173660112 |
| VWA3A | 1.79 | 1184 | 133.9 | 8.46 | 0.17484965 |
| SLC1A5 | 218.29 | 541 | 56.6 | 5.48 | 0.175041079 |
| ABHD11 | 3.6 | 308 | 33.8 | 9.25 | 0.175258971 |
| ATP1B1 | 36.84 | 301 | 34.9 | 8.65 | 0.175627452 |
| PTGR1 | 200.8 | 301 | 32.9 | 7.2 | 0.17563194 |
| HEXA | 6.8 | 529 | 60.7 | 5.16 | 0.175935182 |
| PSMG3 | 22.48 | 122 | 13.1 | 7.88 | 0.175942911 |
| CYBRD1 | 3.04 | 228 | 25.1 | 8.44 | 0.175999099 |
| GFM1 | 18.4 | 751 | 83.4 | 7.01 | 0.17615138 |
| NEMF | 2.15 | 1034 | 118 | 6.16 | 0.176261245 |
| VAMP7 | 9.07 | 179 | 20.1 | 9.29 | 0.176362209 |
| PSMG1 | 20.93 | 267 | 30.3 | 7.77 | 0.176724362 |
| DDX52 | 8.8 | 599 | 67.5 | 9.67 | 0.176783806 |
| RAP2C | 16.37 | 183 | 20.7 | 4.94 | 0.17742059 |
| ACADSB | 18.23 | 330 | 36 | 6.24 | 0.178321278 |
| LSM8 | 25.98 | 96 | 10.4 | 4.48 | 0.178477327 |
| PRKRA | 9.01 | 288 | 31.6 | 8.43 | 0.178629982 |
| EIF1AD | 5.54 | 165 | 19 | 5.21 | 0.178769332 |
| SLC7A6 | 10.85 | 515 | 56.8 | 5.88 | 0.178920092 |
| ADAM15 | 8.01 | 633 | 68.2 | 6.62 | 0.17934024 |
| CWF19L1 | 4.89 | 401 | 45.5 | 7.66 | 0.179707159 |
| GSTO1 | 335.95 | 241 | 27.5 | 6.6 | 0.179980775 |
| SPATS2L | 20.49 | 489 | 54.1 | 9.63 | 0.180589438 |
| UBE2N | 153.22 | 152 | 17.1 | 6.57 | 0.180688037 |
| CCNYL1 | 2.2 | 289 | 33.4 | 6.93 | 0.180722415 |
| CUL3 | 30.37 | 702 | 81 | 8.51 | 0.180788334 |
| KIF4A | 19.19 | 1232 | 139.8 | 6.27 | 0.180896787 |
| PLSCR3 | 4.19 | 295 | 31.6 | 6.65 | 0.180900371 |
| TRIM4 | 2.52 | 474 | 54.1 | 7.99 | 0.181198141 |
| ARF4 | 356.53 | 180 | 20.5 | 7.14 | 0.1816646 |
| DPY30 | 10.92 | 99 | 11.2 | 4.88 | 0.181911984 |
| RPS6KA3 | 63.48 | 740 | 83.7 | 6.89 | 0.182255754 |
| HMOX2 | 45.44 | 287 | 32.8 | 5.91 | 0.182287889 |
| TRIM2 | 2.49 | 744 | 81.5 | 6.96 | 0.182378229 |
| BRAT1 | 13.54 | 821 | 88.1 | 5.27 | 0.182378502 |
| S100P | 69.33 | 95 | 10.4 | 4.88 | 0.18271922 |
| EARS2 | 8.71 | 523 | 58.7 | 8.76 | 0.182983158 |
| WDR6 | 2.46 | 1121 | 121.6 | 6.87 | 0.183597927 |
| TRIM32 | 4.98 | 653 | 71.9 | 6.98 | 0.18361018 |
| NLE1 | 12.73 | 485 | 53.3 | 7.34 | 0.18400455 |
| TRRAP | 2.29 | 3830 | 434.1 | 8.22 | 0.184971153 |
| EMG1 | 22.44 | 244 | 26.7 | 9.17 | 0.185011436 |
| MIF4GD | 2.78 | 222 | 25.4 | 5.33 | 0.18562042 |
| AK3 | 2.9 | 158 | 18.3 | 8.78 | 0.186019922 |
| COQ9 | 21.01 | 318 | 35.5 | 5.94 | 0.186075633 |
| ABHD12 | 8.47 | 360 | 41.4 | 8.81 | 0.186369203 |
| FANCI | 5.7 | 1267 | 142.3 | 6.81 | 0.186409014 |
| GSN | 2.87 | 731 | 80.6 | 5.85 | 0.186496063 |
| CIP2A | 9.11 | 746 | 84.5 | 6.48 | 0.186549211 |
| ABLIM1 | 2.15 | 401 | 46.1 | 8.38 | 0.186707403 |
| RAP2B | 16.98 | 183 | 20.5 | 4.81 | 0.186919916 |
| TXLNA | 25.59 | 546 | 61.9 | 6.52 | 0.18742431 |
| RILPL1 | 4.9 | 362 | 42.2 | 5.2 | 0.187693637 |
| UHRF1 | 15.42 | 793 | 89.8 | 7.56 | 0.188080105 |
| TTC5 | 2.65 | 440 | 48.9 | 6.48 | 0.188208637 |
| PPA2 | 143.57 | 334 | 37.9 | 7.39 | 0.188230905 |
| CERS2 | 32.21 | 380 | 44.8 | 8.98 | 0.188362353 |
| CD81 | 17.64 | 236 | 25.8 | 5.29 | 0.188518037 |
| PEBP1 | 155.51 | 187 | 21 | 7.53 | 0.18964699 |
| TOMM7 | 2.82 | 55 | 6.2 | 10.29 | 0.189660769 |
| IKBKG | 11.78 | 419 | 48.2 | 5.71 | 0.190550939 |
| TOP1 | 146.37 | 765 | 90.7 | 9.31 | 0.19078691 |
| TRMT5 | 9.48 | 509 | 58.2 | 8.62 | 0.190815949 |
| LRRC47 | 21.78 | 583 | 63.4 | 8.28 | 0.191062885 |
| EIF4A2 | 685.3 | 407 | 46.4 | 5.48 | 0.191460246 |
| CTNNA1 | 266.94 | 906 | 100 | 6.29 | 0.191466346 |
| C1orf174 | 2.85 | 243 | 26 | 6.9 | 0.191894833 |
| TREX1 | 7.07 | 304 | 32.3 | 7.93 | 0.192020513 |
| PRKACA | 12.99 | 351 | 40.6 | 8.79 | 0.192551775 |
| AKR1C3 | 792.16 | 323 | 36.8 | 7.94 | 0.192902808 |
| SRP14 | 31.6 | 136 | 14.6 | 10.04 | 0.192902825 |
| ATP1A1 | 364.17 | 1023 | 112.8 | 5.49 | 0.19295012 |
| ACOT7 | 156.82 | 329 | 36.5 | 7.78 | 0.193012566 |
| NOM1 | 3.29 | 860 | 96.2 | 8.1 | 0.193367351 |
| CSNK1A1 | 14.6 | 337 | 38.9 | 9.57 | 0.193937367 |
| MT-ND5 | 3.08 | 603 | 67 | 9.03 | 0.194020674 |
| YWHAH | 588.34 | 246 | 28.2 | 4.84 | 0.19406155 |
| CDK9 | 26.89 | 372 | 42.8 | 8.79 | 0.194204613 |
| HSPB8 | 26.5 | 196 | 21.6 | 5.12 | 0.195016148 |
| STAT3 | 11.07 | 722 | 83.1 | 7.12 | 0.195292784 |
| ADAM10 | 2.38 | 447 | 49 | 8.03 | 0.19531003 |
| HERC1 | 0 | 4861 | 531.9 | 6.04 | 0.195955795 |
| NCOA5 | 19.23 | 579 | 65.5 | 9.6 | 0.196291411 |
| UBR7 | 31.61 | 425 | 48 | 4.81 | 0.196345297 |
| SUPT5H | 22.01 | 1083 | 120.4 | 5.1 | 0.196745879 |
| IFT22 | 2.28 | 108 | 12.4 | 5.74 | 0.197061721 |
| EIF4G3 | 73.65 | 1305 | 146.8 | 6.06 | 0.197906365 |
| IRF2BP1 | 0 | 584 | 61.6 | 8.18 | 0.198230546 |
| ACOT13 | 16.46 | 117 | 12.4 | 8.5 | 0.19823634 |
| SSR1 | 67.33 | 286 | 32.2 | 4.49 | 0.198764734 |
| PPM1G | 145.24 | 546 | 59.2 | 4.36 | 0.198776889 |
| MRPS7 | 3.67 | 133 | 15.2 | 5.8 | 0.199676768 |
| ALYREF | 95.3 | 257 | 26.9 | 11.15 | 0.199987277 |
| NCLN | 28.26 | 562 | 62.8 | 6.89 | 0.200272971 |
| VPS25 | 12.13 | 176 | 20.7 | 6.34 | 0.20052846 |
| ECH1 | 79.04 | 328 | 35.8 | 8 | 0.200679156 |
| HFM1 | 0 | 1435 | 162.5 | 7.09 | 0.200814085 |
| CCT3 | 700.95 | 545 | 60.5 | 6.49 | 0.20117386 |
| NAGK | 7.95 | 344 | 37.4 | 6.24 | 0.202225334 |
| MIC13 | 9.69 | 118 | 13.1 | 9.42 | 0.202394359 |
| PRSS1 | 16.2 | 247 | 26.5 | 6.51 | 0.203151627 |
| DIMT1 | 14.55 | 313 | 35.2 | 9.99 | 0.203235924 |
| THOC1 | 5.98 | 657 | 75.6 | 4.98 | 0.203503246 |
| IPO5 | 255.24 | 1097 | 123.5 | 4.94 | 0.203994691 |
| RPS21 | 129.69 | 83 | 9.1 | 8.5 | 0.204250974 |
| MCL1 | 2.08 | 271 | 28.6 | 6.58 | 0.204377426 |
| PEPD | 31.28 | 429 | 47.2 | 6.07 | 0.204900282 |
| EXOSC5 | 9.66 | 235 | 25.2 | 7.59 | 0.205477809 |
| MAPK1 | 39 | 316 | 36.4 | 6.52 | 0.205754123 |
| PGRMC2 | 62.66 | 223 | 23.8 | 4.88 | 0.205996207 |
| B4GALNT1 | 2.04 | 478 | 52.8 | 8.88 | 0.206260991 |
| GABPA | 5.79 | 454 | 51.3 | 4.97 | 0.206837623 |
| ERMP1 | 2.27 | 904 | 100.2 | 7.52 | 0.207106098 |
| C2orf69 | 3.62 | 385 | 43.4 | 8.09 | 0.207220683 |
| SCFD1 | 18.19 | 642 | 72.3 | 6.27 | 0.207526425 |
| TECTA | 0 | 2155 | 239.4 | 5.4 | 0.207554524 |
| PXN | 3.26 | 557 | 60.9 | 6.35 | 0.207589847 |
| WASHC5 | 24.37 | 1159 | 134.2 | 6.98 | 0.207815652 |
| BCAT2 | 26.25 | 300 | 33.8 | 7.72 | 0.207921779 |
| CASC4 | 4.91 | 380 | 43.3 | 6.65 | 0.20835151 |
| NDUFS8 | 22.7 | 210 | 23.7 | 6.34 | 0.209631954 |
| CLINT1 | 23.31 | 625 | 68.2 | 6.42 | 0.209742816 |
| IARS2 | 299.03 | 1012 | 113.7 | 7.2 | 0.21039085 |
| DIABLO | 20.72 | 186 | 21.2 | 4.81 | 0.210613329 |
| SHMT2 | 452.25 | 483 | 53.4 | 8.15 | 0.211335924 |
| ABHD10 | 2.83 | 149 | 16.9 | 6.73 | 0.211640041 |
| PSMD5 | 92.14 | 461 | 51.3 | 5.44 | 0.212039393 |
| CNKSR2 | 0 | 849 | 96.3 | 6.61 | 0.212059129 |
| LBR | 71.8 | 615 | 70.7 | 9.36 | 0.212126677 |
| RPA3 | 52.72 | 121 | 13.6 | 5.08 | 0.213060269 |
| PTGES2 | 57.79 | 377 | 41.9 | 9.16 | 0.213561171 |
| WIPF2 | 7.44 | 440 | 46.3 | 10.93 | 0.214002455 |
| SCLY | 5.61 | 445 | 48.1 | 7.12 | 0.214109436 |
| UBE4A | 7.53 | 1066 | 122.5 | 5.24 | 0.214322905 |
| PEA15 | 51.61 | 130 | 15 | 5.02 | 0.214555265 |
| RRP36 | 4.01 | 253 | 29.1 | 10.01 | 0.214965098 |
| POLR2B | 16.12 | 1174 | 133.8 | 6.87 | 0.215066728 |
| UBTF | 36.6 | 727 | 84.9 | 5.55 | 0.215724691 |
| RPL29 | 115.57 | 159 | 17.7 | 11.66 | 0.215823845 |
| MTHFD2 | 71.6 | 350 | 37.9 | 8.73 | 0.215958467 |
| GNL3L | 2.31 | 582 | 65.5 | 8.44 | 0.216289212 |
| PALLD | 132.47 | 1001 | 108.6 | 7.27 | 0.216607697 |
| ARAF | 10.22 | 606 | 67.5 | 9.01 | 0.216943394 |
| REM1 | 12.6 | 298 | 32.9 | 8.79 | 0.217425379 |
| NOP14 | 9.42 | 806 | 91.3 | 6.58 | 0.217483194 |
| CTPS1 | 157.23 | 591 | 66.6 | 6.46 | 0.218126556 |
| HMGN4 | 5.28 | 90 | 9.5 | 10.48 | 0.218388262 |
| RSRC2 | 5.71 | 386 | 44.9 | 11.33 | 0.218531497 |
| DARS2 | 73.08 | 645 | 73.5 | 8.02 | 0.21868894 |
| GNAQ | 29.13 | 359 | 42.1 | 5.68 | 0.219051301 |
| DCTN2 | 126.25 | 401 | 44.2 | 5.21 | 0.219209619 |
| CAMSAP2 | 2.35 | 1462 | 165 | 6.71 | 0.219619576 |
| LYSMD2 | 5.22 | 215 | 23.4 | 5.35 | 0.219713536 |
| SNX5 | 21.95 | 404 | 46.8 | 6.76 | 0.219798337 |
| ARMCX3 | 9.69 | 379 | 42.5 | 8.37 | 0.219875064 |
| TIMM13 | 59.03 | 95 | 10.5 | 8.18 | 0.220168588 |
| RHOC | 190.62 | 193 | 22 | 6.58 | 0.220284479 |
| DSCR3 | 3.37 | 270 | 29.9 | 7.12 | 0.220432247 |
| TCERG1 | 73.03 | 1077 | 121.6 | 8.54 | 0.220454736 |
| SCAMP3 | 77.92 | 347 | 38.3 | 7.64 | 0.22096882 |
| GSAP | 8.46 | 248 | 28.5 | 7.43 | 0.22191583 |
| RHOG | 37.93 | 191 | 21.3 | 8.12 | 0.221923724 |
| ERGIC1 | 45.02 | 198 | 22.1 | 9.16 | 0.223107136 |
| CA12 | 16.67 | 343 | 38.4 | 7.23 | 0.223248114 |
| KANK1 | 5.83 | 1194 | 129.7 | 5.14 | 0.223846645 |
| PARVA | 10.65 | 372 | 42.2 | 5.95 | 0.224010858 |
| ACTBL2 | 1743.18 | 376 | 42 | 5.59 | 0.224129457 |
| MT-ATP6 | 24.38 | 226 | 24.8 | 10.1 | 0.224804251 |
| LTN1 | 6.42 | 1766 | 200.4 | 6.25 | 0.225603033 |
| MBNL1 | 11.8 | 302 | 33 | 8.82 | 0.225889575 |
| SNCA | 95.82 | 112 | 11.4 | 8.29 | 0.225977682 |
| GLRX5 | 46.49 | 157 | 16.6 | 6.79 | 0.226039244 |
| MRPL39 | 16.21 | 338 | 38.7 | 7.65 | 0.226502967 |
| STXBP3 | 7.22 | 592 | 67.7 | 7.8 | 0.226709874 |
| CDK5RAP3 | 16.28 | 506 | 56.9 | 4.75 | 0.226713542 |
| COTL1 | 25.18 | 142 | 15.9 | 5.67 | 0.226820858 |
| NDUFAF4 | 7.46 | 175 | 20.3 | 8.82 | 0.226854106 |
| RAD23A | 30.5 | 308 | 33.4 | 4.72 | 0.227502549 |
| SUGT1 | 48.92 | 333 | 37.8 | 5.21 | 0.228206567 |
| TARDBP | 44.72 | 414 | 44.7 | 6.19 | 0.228218291 |
| PDCD2 | 2.81 | 311 | 35.3 | 5.29 | 0.22856419 |
| FIS1 | 16.4 | 152 | 16.9 | 8.79 | 0.228605908 |
| NAA15 | 85.46 | 866 | 101.2 | 7.42 | 0.229135768 |
| LDLR | 10.59 | 692 | 76.8 | 5.45 | 0.229371407 |
| ATG9A | 9.96 | 778 | 87.3 | 6.92 | 0.229460715 |
| CHCHD10 | 3.19 | 142 | 14.1 | 7.84 | 0.230138651 |
| ZNF598 | 22.51 | 896 | 97.7 | 8.28 | 0.230256809 |
| CLPX | 35.88 | 633 | 69.2 | 7.58 | 0.230430749 |
| MTHFD1 | 478.01 | 935 | 101.5 | 7.3 | 0.230490644 |
| DHRS7 | 36.11 | 289 | 32.2 | 8.87 | 0.230627847 |
| EFEMP1 | 40.62 | 485 | 53.7 | 5.02 | 0.230652383 |
| KIF20A | 2.8 | 890 | 100.2 | 6.92 | 0.23171431 |
| PDCD6 | 75.53 | 191 | 21.9 | 5.4 | 0.232188934 |
| CPD | 14.49 | 1380 | 152.8 | 6.05 | 0.232377278 |
| CAPN2 | 160.95 | 700 | 79.9 | 4.98 | 0.232574928 |
| GGPS1 | 2.36 | 246 | 28.4 | 5.81 | 0.232588948 |
| EPN3 | 4.16 | 208 | 23.6 | 9.42 | 0.232906913 |
| TGFBR1 | 13.54 | 503 | 55.9 | 7.55 | 0.233556874 |
| ATP6V0D1 | 32.8 | 351 | 40.3 | 5 | 0.234139601 |
| ACIN1 | 47.36 | 1328 | 150.5 | 6.43 | 0.234224113 |
| DDX27 | 89.85 | 796 | 89.8 | 9.28 | 0.234503628 |
| TARS2 | 15.06 | 718 | 81 | 7.3 | 0.234781697 |
| C1orf131 | 2.64 | 292 | 32.5 | 9.88 | 0.23507262 |
| FABP5 | 223.42 | 135 | 15.2 | 7.01 | 0.235804034 |
| CCDC12 | 2.23 | 166 | 19.2 | 7.34 | 0.236177573 |
| PKN2 | 8.46 | 658 | 75.1 | 5.41 | 0.236828699 |
| COPG1 | 226.28 | 874 | 97.7 | 5.47 | 0.237216662 |
| RHBDF2 | 0 | 827 | 93.3 | 8.59 | 0.237406639 |
| TBC1D4 | 6.02 | 1235 | 139.5 | 6.93 | 0.237703776 |
| DDX51 | 6.04 | 666 | 72.4 | 8.16 | 0.238049344 |
| LAMTOR5 | 10.4 | 91 | 9.6 | 4.87 | 0.238349171 |
| RAB4A | 107.51 | 218 | 24.4 | 6.07 | 0.238380645 |
| VWA8 | 6.09 | 1039 | 116.9 | 8.15 | 0.238406627 |
| AQR | 21.01 | 1485 | 171.2 | 6.37 | 0.238688018 |
| ANKRD44 | 3.94 | 919 | 99.7 | 6.34 | 0.239168018 |
| TMEM126A | 2.81 | 125 | 13.8 | 8.92 | 0.239301938 |
| PSMD11 | 162.94 | 422 | 47.4 | 6.48 | 0.240249634 |
| IDH1 | 358.29 | 414 | 46.6 | 7.01 | 0.240350686 |
| EFTUD2 | 265.77 | 937 | 105.3 | 5.22 | 0.240666168 |
| ANP32B | 277.83 | 195 | 22.3 | 4.3 | 0.240699124 |
| PLP2 | 57.26 | 152 | 16.7 | 7.24 | 0.240750534 |
| CTNND1 | 146.24 | 933 | 104.1 | 6.95 | 0.240857059 |
| SLC12A2 | 99.36 | 1196 | 129.6 | 6.46 | 0.240914987 |
| FHL1 | 9.58 | 194 | 22 | 8.46 | 0.24095184 |
| WDR11 | 9.07 | 1224 | 136.6 | 6.92 | 0.241122549 |
| PPIC | 12.68 | 212 | 22.7 | 8.4 | 0.242623843 |
| DECR2 | 5.98 | 211 | 23.1 | 8.81 | 0.242695849 |
| PDCL | 2.93 | 218 | 24.9 | 4.82 | 0.24443415 |
| DNAJB6 | 8.75 | 241 | 26.9 | 7.55 | 0.244599342 |
| MRPS26 | 5.1 | 205 | 24.2 | 10.39 | 0.244649326 |
| FLOT1 | 28.44 | 427 | 47.3 | 7.49 | 0.245049354 |
| FADD | 5.7 | 208 | 23.3 | 5.69 | 0.245349926 |
| URGCP | 2.75 | 856 | 96.6 | 7.3 | 0.246023073 |
| PHKA1 | 0 | 1151 | 129.3 | 5.96 | 0.246413503 |
| RNF114 | 9.02 | 228 | 25.7 | 7.25 | 0.246553771 |
| VCL | 770.03 | 1066 | 116.6 | 6.09 | 0.246899556 |
| PCBD1 | 63.06 | 104 | 12 | 6.8 | 0.247427775 |
| TMED10 | 168.66 | 219 | 25 | 7.44 | 0.24757126 |
| SART3 | 30.06 | 963 | 109.9 | 5.57 | 0.24762691 |
| CGREF1 | 3.27 | 318 | 33.4 | 4.39 | 0.247749216 |
| RIPOR1 | 3.21 | 1219 | 131.8 | 6.28 | 0.24830217 |
| SLC25A22 | 18.75 | 323 | 34.4 | 9.29 | 0.249392422 |
| MAT2B | 30.41 | 306 | 34.6 | 6.54 | 0.249440071 |
| FAM120A | 65.62 | 1118 | 121.8 | 8.88 | 0.250126959 |
| TM9SF4 | 17.22 | 642 | 74.5 | 6.54 | 0.250266314 |
| SCRN3 | 8.19 | 417 | 47.6 | 5.95 | 0.250312185 |
| WBP11 | 4.52 | 641 | 70 | 8.38 | 0.251042206 |
| TNFRSF10B | 3.31 | 411 | 45.1 | 5.66 | 0.25109285 |
| ADD3 | 0 | 674 | 75.6 | 6.81 | 0.251125692 |
| WDR18 | 15.18 | 432 | 47.4 | 6.7 | 0.25159677 |
| SRSF6 | 161.07 | 335 | 38.4 | 11 | 0.252045611 |
| SLIRP | 102.13 | 107 | 12.1 | 10.24 | 0.252951902 |
| CAMK2D | 11.98 | 478 | 54.1 | 7.12 | 0.252963636 |
| SEPT6 | 71.68 | 427 | 48.8 | 6.81 | 0.253576976 |
| GNAI3 | 76.05 | 354 | 40.5 | 5.69 | 0.2545462 |
| COQ5 | 3.22 | 246 | 27.9 | 9.14 | 0.254579498 |
| SRXN1 | 175.53 | 137 | 14.3 | 8.19 | 0.254723596 |
| EPB41L2 | 26.16 | 852 | 95.5 | 5.77 | 0.255727984 |
| PSMB3 | 178.16 | 205 | 22.9 | 6.55 | 0.255983306 |
| NDUFA9 | 39.6 | 377 | 42.5 | 9.8 | 0.256172911 |
| CASP3 | 5.33 | 277 | 31.6 | 6.54 | 0.256798334 |
| GTF2E2 | 8.74 | 291 | 33 | 9.66 | 0.256822164 |
| XPO5 | 57.63 | 1204 | 136.2 | 5.8 | 0.25708102 |
| AK6 | 7.47 | 169 | 19.8 | 4.74 | 0.257738834 |
| MTCH2 | 25.89 | 303 | 33.3 | 7.97 | 0.257812482 |
| ATP6V1B2 | 30.09 | 511 | 56.5 | 5.81 | 0.258487119 |
| GNAI2 | 118.35 | 355 | 40.4 | 5.54 | 0.25858254 |
| EML4 | 49.59 | 981 | 108.8 | 6.4 | 0.258707068 |
| CEP85L | 0 | 805 | 91.8 | 6.33 | 0.259065202 |
| RUVBL2 | 344.1 | 463 | 51.1 | 5.64 | 0.259088114 |
| MMGT1 | 3.78 | 131 | 14.7 | 9.16 | 0.259278863 |
| SAMD4A | 0 | 617 | 67.9 | 8.72 | 0.259650119 |
| NUP85 | 32.94 | 656 | 75 | 5.55 | 0.259898146 |
| AARS2 | 8.91 | 985 | 107.3 | 6.27 | 0.260086814 |
| MRPL18 | 5.36 | 180 | 20.6 | 9.54 | 0.26031162 |
| SPNS1 | 3.4 | 454 | 49 | 7.75 | 0.26089609 |
| MED14 | 2.32 | 1454 | 160.5 | 8.73 | 0.260946186 |
| LAMP1 | 8.89 | 417 | 44.9 | 8.75 | 0.260970786 |
| GOLM1 | 10.79 | 391 | 44.2 | 4.91 | 0.260982489 |
| HMGB3 | 34.2 | 200 | 23 | 8.37 | 0.261256092 |
| PSMD4 | 96.21 | 377 | 40.7 | 4.79 | 0.261332896 |
| SENP3 | 14.59 | 574 | 65 | 8.56 | 0.261539468 |
| GOLGB1 | 17.26 | 3259 | 375.8 | 5 | 0.261837059 |
| HARS | 274.65 | 509 | 57.4 | 5.88 | 0.261962613 |
| ABCF3 | 23.86 | 703 | 78.9 | 6.25 | 0.262724344 |
| RPL36AL | 75.52 | 106 | 12.5 | 10.65 | 0.262793608 |
| AP3S1 | 9.7 | 193 | 21.7 | 5.39 | 0.262868649 |
| TTC33 | 2.37 | 262 | 29.4 | 5.44 | 0.2628908 |
| PSAP | 200.99 | 524 | 58.1 | 5.17 | 0.263178314 |
| RPS5 | 231.62 | 204 | 22.9 | 9.72 | 0.263185328 |
| ZCCHC6 | 3.69 | 412 | 47.9 | 8.91 | 0.263748914 |
| DDX10 | 4.51 | 875 | 100.8 | 8.63 | 0.263809911 |
| TAP2 | 1.96 | 653 | 72 | 7.84 | 0.263838931 |
| SF3B6 | 36.72 | 125 | 14.6 | 9.38 | 0.264289289 |
| DNPH1 | 12.35 | 174 | 19.1 | 5.05 | 0.264482026 |
| H2AFX | 324.48 | 143 | 15.1 | 10.74 | 0.265242257 |
| RBM28 | 38.98 | 759 | 85.7 | 9.22 | 0.265244 |
| MRM1 | 6.54 | 353 | 38.6 | 7.94 | 0.266753731 |
| FDXR | 66.64 | 491 | 53.8 | 8.44 | 0.266881356 |
| WBP2 | 8.41 | 261 | 28.1 | 5.91 | 0.267361084 |
| TNS3 | 25.89 | 1205 | 129 | 7.2 | 0.267535423 |
| ANTXR1 | 39.12 | 564 | 62.7 | 7.61 | 0.26766889 |
| SLC25A40 | 2.06 | 338 | 38.1 | 9.35 | 0.268140986 |
| ARHGAP18 | 9.85 | 618 | 70.1 | 6.11 | 0.268212322 |
| NDUFB8 | 11.31 | 155 | 18.5 | 5.54 | 0.26971046 |
| DEK | 35.34 | 375 | 42.6 | 8.56 | 0.269991003 |
| RPL11 | 180.17 | 177 | 20.1 | 9.6 | 0.270139188 |
| CETN2 | 10.55 | 172 | 19.7 | 5 | 0.270846408 |
| MAP1LC3B2 | 5.23 | 125 | 14.6 | 8.68 | 0.271232039 |
| BLMH | 66.31 | 455 | 52.5 | 6.27 | 0.271293692 |
| C9orf78 | 12.89 | 289 | 33.7 | 6.74 | 0.2713149 |
| AURKB | 3.11 | 142 | 16.2 | 10.05 | 0.27183375 |
| LMAN2 | 165.31 | 356 | 40.2 | 6.95 | 0.272558239 |
| PEX11B | 9.71 | 245 | 26.7 | 9.91 | 0.272751529 |
| PSMB5 | 186.72 | 263 | 28.5 | 6.92 | 0.272838642 |
| HNRNPA0 | 266.26 | 305 | 30.8 | 9.29 | 0.273093313 |
| TXNIP | 2.6 | 336 | 37.3 | 8.05 | 0.273582918 |
| SSR4 | 68.16 | 173 | 19 | 6.15 | 0.273754409 |
| FAM98B | 44.41 | 330 | 37.2 | 6.29 | 0.273771228 |
| TNFAIP2 | 6.23 | 654 | 72.6 | 6.46 | 0.274025557 |
| MRPL30 | 4.78 | 131 | 15.2 | 10.32 | 0.274438272 |
| VAMP3 | 33.18 | 100 | 11.3 | 8.79 | 0.274515535 |
| CHMP3 | 6.1 | 182 | 20.8 | 4.77 | 0.274771272 |
| ARHGAP1 | 3.52 | 439 | 50.4 | 6.29 | 0.27490976 |
| PUM3 | 24.36 | 648 | 73.5 | 9.64 | 0.275101266 |
| PRTFDC1 | 4.09 | 190 | 21.5 | 7.03 | 0.275334835 |
| TKFC | 26.62 | 575 | 58.9 | 7.49 | 0.275346093 |
| GOSR1 | 0 | 185 | 21.2 | 9.61 | 0.275415389 |
| NUMA1 | 72.04 | 2101 | 236.4 | 5.8 | 0.275611296 |
| PRPF31 | 15.53 | 499 | 55.4 | 5.78 | 0.275928765 |
| ECHDC1 | 3.33 | 226 | 24.8 | 7.11 | 0.276329451 |
| NFKB2 | 26.57 | 899 | 96.6 | 6.25 | 0.276447286 |
| DNM2 | 22.54 | 866 | 97.6 | 7.64 | 0.276521015 |
| ANXA7 | 195.89 | 466 | 50.3 | 6.61 | 0.277014548 |
| XRN2 | 56.31 | 950 | 108.5 | 7.47 | 0.277267228 |
| ACSL1 | 15.39 | 688 | 76.8 | 6.99 | 0.277847889 |
| MAZ | 8.54 | 454 | 46.3 | 8.92 | 0.27796016 |
| GALC | 20.51 | 685 | 77 | 6.64 | 0.278557971 |
| LMO7 | 3.39 | 1349 | 153.6 | 7.75 | 0.278646158 |
| MRPL4 | 33.81 | 263 | 29.5 | 10.13 | 0.278913333 |
| RAD21 | 16.25 | 631 | 71.6 | 4.65 | 0.279204566 |
| RNASEH1 | 6.47 | 286 | 32 | 9.16 | 0.279510173 |
| NDUFB9 | 10.56 | 179 | 21.8 | 8.38 | 0.279675338 |
| PTP4A2 | 27.88 | 167 | 19.1 | 8.37 | 0.279961217 |
| SMAP2 | 3.05 | 349 | 37.7 | 9.11 | 0.281224537 |
| PGRMC1 | 126.15 | 195 | 21.7 | 4.7 | 0.281882954 |
| MAN2C1 | 0 | 941 | 104.6 | 6.46 | 0.282011377 |
| CSK | 19.41 | 450 | 50.7 | 7.06 | 0.282025593 |
| TLCD1 | 7.87 | 247 | 28.5 | 9.47 | 0.282068733 |
| CTBP1 | 11.94 | 429 | 46.4 | 6.65 | 0.282788314 |
| GMDS | 12.18 | 342 | 39 | 7.08 | 0.282799202 |
| PNO1 | 16.4 | 252 | 27.9 | 9.73 | 0.283309969 |
| RBM15 | 3.15 | 913 | 99.6 | 9.92 | 0.283438958 |
| BCL10 | 2.04 | 233 | 26.2 | 5.74 | 0.284323312 |
| PBK | 78.06 | 322 | 36.1 | 5.12 | 0.284678856 |
| PSMD7 | 129.2 | 324 | 37 | 6.77 | 0.284689027 |
| ACTA1 | 3642.75 | 377 | 42 | 5.39 | 0.284827541 |
| TCOF1 | 91.87 | 1488 | 152 | 9.04 | 0.284871038 |
| ECI1 | 64.6 | 285 | 30.9 | 8.9 | 0.28539178 |
| MRPL43 | 20.97 | 159 | 17.8 | 9.88 | 0.285514927 |
| TOR1B | 8.97 | 336 | 38 | 8.54 | 0.285639237 |
| RAB3GAP1 | 16.58 | 981 | 110.5 | 5.55 | 0.286187055 |
| PPP1R10 | 15.96 | 940 | 99 | 9.17 | 0.286545413 |
| COPS7A | 2.63 | 275 | 30.3 | 8.22 | 0.28669098 |
| DDOST | 147.73 | 456 | 50.8 | 6.55 | 0.287180842 |
| MAIP1 | 5.39 | 291 | 32.5 | 9.17 | 0.287681188 |
| FAM114A1 | 25.06 | 356 | 40 | 4.98 | 0.287826534 |
| SSRP1 | 198.87 | 709 | 81 | 6.87 | 0.287862326 |
| PYM1 | 9.92 | 203 | 22.7 | 9.44 | 0.288127549 |
| C1orf21 | 2.95 | 121 | 13.9 | 5.22 | 0.288362017 |
| PIR | 72.53 | 290 | 32.1 | 6.92 | 0.288547346 |
| VPS26B | 10.44 | 336 | 39.1 | 7.36 | 0.288583912 |
| MED30 | 2.77 | 143 | 16.3 | 9.2 | 0.289020342 |
| ATP2C1 | 8.36 | 888 | 96.9 | 6.32 | 0.289478469 |
| DNAJB11 | 46.3 | 358 | 40.5 | 6.18 | 0.289566445 |
| PPP1R14B | 8.7 | 147 | 15.9 | 4.86 | 0.289754598 |
| NELFB | 8.89 | 580 | 65.7 | 6.13 | 0.290251931 |
| BANF1 | 63.28 | 89 | 10.1 | 6.09 | 0.29041302 |
| DCTN1 | 79.32 | 1236 | 136.7 | 5.4 | 0.290419573 |
| MRPL3 | 14.42 | 348 | 38.6 | 9.48 | 0.29049365 |
| RAB10 | 332.62 | 200 | 22.5 | 8.38 | 0.29090071 |
| BLVRA | 103.12 | 296 | 33.4 | 6.44 | 0.291064768 |
| MAP7 | 44.13 | 712 | 79.9 | 9.57 | 0.291489701 |
| ZMAT2 | 5.31 | 199 | 23.6 | 9.01 | 0.293068916 |
| IFRD1 | 3.17 | 451 | 50.2 | 7.18 | 0.293169191 |
| CLIC4 | 25.01 | 253 | 28.8 | 5.59 | 0.293185792 |
| HNRNPM | 848.63 | 730 | 77.5 | 8.7 | 0.293564388 |
| MYEF2 | 9.26 | 576 | 61.9 | 8.62 | 0.293635042 |
| EIF4E2 | 8.74 | 234 | 27 | 7.99 | 0.293920838 |
| SERF2 | 0 | 45 | 5.2 | 10.07 | 0.293921267 |
| PAFAH1B1 | 75 | 410 | 46.6 | 7.37 | 0.29426977 |
| ALDH9A1 | 134.33 | 494 | 53.8 | 5.87 | 0.294891695 |
| UBR3 | 0 | 1888 | 212.3 | 6.1 | 0.29526052 |
| IAH1 | 0 | 135 | 15.2 | 4.64 | 0.295788007 |
| TTC37 | 19.91 | 1564 | 175.4 | 7.53 | 0.296271472 |
| ANKHD1 | 29.19 | 2542 | 269.3 | 5.73 | 0.296902432 |
| MRE11 | 29.41 | 680 | 77.6 | 5.82 | 0.296910242 |
| DNAH10 | 0 | 4471 | 514.5 | 5.88 | 0.297141191 |
| BZW2 | 113.22 | 419 | 48.1 | 6.68 | 0.297308482 |
| IGF2BP3 | 81 | 579 | 63.7 | 8.87 | 0.297392406 |
| PPIF | 174.74 | 207 | 22 | 9.38 | 0.298153754 |
| RAB13 | 150.02 | 203 | 22.8 | 9.19 | 0.298192249 |
| ABCC1 | 41.76 | 1475 | 165.5 | 6.92 | 0.298424414 |
| IRAK1 | 3.47 | 633 | 68 | 6.43 | 0.298703629 |
| ATR | 4.91 | 2580 | 294 | 7.43 | 0.298944163 |
| FEN1 | 65.07 | 380 | 42.6 | 8.62 | 0.299169286 |
| RRM1 | 103.13 | 792 | 90 | 7.15 | 0.299404554 |
| CD109 | 2.73 | 665 | 74.4 | 7.4 | 0.299562277 |
| CFL2 | 231.58 | 166 | 18.7 | 7.88 | 0.299746554 |
| YTHDC1 | 2.7 | 709 | 82.6 | 5.96 | 0.300259744 |
| CTH | 2.44 | 373 | 41.2 | 6.86 | 0.300559821 |
| NOP2 | 87.11 | 812 | 89.2 | 9.23 | 0.300744015 |
| QRICH1 | 4.7 | 776 | 86.4 | 5.87 | 0.300947569 |
| TIA1 | 33.85 | 375 | 41.8 | 7.74 | 0.301275695 |
| WDR3 | 35.66 | 943 | 106 | 6.64 | 0.301576194 |
| PRKAR1A | 155.71 | 381 | 43 | 5.35 | 0.302377794 |
| MT-ND4 | 2.69 | 459 | 51.5 | 9.35 | 0.302829254 |
| SGPP1 | 5.14 | 441 | 49.1 | 8.82 | 0.303120911 |
| NT5DC1 | 8.29 | 455 | 51.8 | 6.35 | 0.303208783 |
| EBP | 10.95 | 230 | 26.3 | 7.9 | 0.303532516 |
| RCC1 | 136.84 | 421 | 44.9 | 7.52 | 0.303736402 |
| CEP44 | 2.54 | 390 | 44.1 | 5.21 | 0.304444524 |
| S100A13 | 30.98 | 98 | 11.5 | 6.16 | 0.304567887 |
| HDGFL2 | 26.98 | 670 | 74.2 | 7.49 | 0.304633231 |
| ZNF800 | 2.57 | 664 | 75.2 | 9.47 | 0.305693434 |
| WWTR1 | 2.78 | 400 | 44.1 | 5.82 | 0.305828684 |
| EMD | 69.34 | 254 | 29 | 5.5 | 0.306111059 |
| ARHGEF1 | 28.38 | 879 | 98.7 | 5.57 | 0.306140708 |
| PSMC2 | 232.77 | 433 | 48.6 | 5.95 | 0.307349367 |
| IPO11 | 12.68 | 975 | 112.5 | 5.25 | 0.307932309 |
| ECE1 | 16.07 | 754 | 85.5 | 5.8 | 0.308314735 |
| NMD3 | 6.03 | 503 | 57.6 | 7.14 | 0.308378416 |
| MCM2 | 178.03 | 904 | 101.8 | 5.52 | 0.309650844 |
| L2HGDH | 3.07 | 441 | 48.5 | 8.65 | 0.309939554 |
| ALDH1A1 | 165.62 | 501 | 54.8 | 6.73 | 0.31013566 |
| EMC2 | 21.43 | 297 | 34.8 | 6.57 | 0.310256358 |
| PKP3 | 2.58 | 797 | 87 | 9.32 | 0.310703459 |
| ARPC1A | 17.03 | 370 | 41.5 | 8.18 | 0.311014866 |
| EIF2S2 | 89.63 | 333 | 38.4 | 5.8 | 0.311198148 |
| RNPS1 | 36.87 | 268 | 30.3 | 11.85 | 0.311353032 |
| FBXO7 | 3.19 | 522 | 58.5 | 6.55 | 0.311579866 |
| PTRHD1 | 10.09 | 140 | 15.8 | 9.1 | 0.31171813 |
| GALNT2 | 99.84 | 571 | 64.7 | 8.35 | 0.311751697 |
| GAK | 2.69 | 1232 | 134.4 | 5.8 | 0.312016214 |
| NT5DC2 | 10.71 | 390 | 46 | 8.07 | 0.312030948 |
| CHCHD5 | 11.89 | 110 | 12.4 | 6.73 | 0.312312169 |
| MRPL58 | 20.77 | 206 | 23.6 | 10.07 | 0.312610698 |
| NUTF2 | 120.69 | 127 | 14.5 | 5.38 | 0.313000713 |
| ACAD9 | 8.72 | 621 | 68.7 | 7.96 | 0.313838765 |
| DERL1 | 18.24 | 231 | 26.4 | 9.54 | 0.314018538 |
| DERA | 42.19 | 318 | 35.2 | 8.94 | 0.314141529 |
| AGPS | 105.56 | 658 | 72.9 | 7.34 | 0.314195667 |
| UBE2S | 15.06 | 222 | 23.8 | 8.38 | 0.314317277 |
| FXR1 | 59.66 | 621 | 69.7 | 6.15 | 0.314342048 |
| DAG1 | 7.8 | 895 | 97.4 | 8.56 | 0.314413283 |
| BAG2 | 69.93 | 211 | 23.8 | 6.7 | 0.314500432 |
| ALDH6A1 | 2.59 | 522 | 56.2 | 8.37 | 0.315114557 |
| TRAP1 | 733.29 | 651 | 74.2 | 7.87 | 0.315538047 |
| EHBP1L1 | 5.18 | 1523 | 161.8 | 4.83 | 0.315614465 |
| MAP1A | 163.43 | 2803 | 305.3 | 4.92 | 0.315682859 |
| MAD1L1 | 2.93 | 626 | 72.2 | 6.81 | 0.315694523 |
| FTO | 4.18 | 505 | 58.2 | 5.22 | 0.315921254 |
| PSMB7 | 139.97 | 277 | 29.9 | 7.68 | 0.315961885 |
| SF3A2 | 21.03 | 464 | 49.2 | 9.64 | 0.31623456 |
| ME2 | 86.01 | 584 | 65.4 | 7.61 | 0.316482882 |
| SELENBP1 | 11.23 | 472 | 52.4 | 6.37 | 0.316756587 |
| GLOD4 | 70.03 | 298 | 33.2 | 5.6 | 0.316933759 |
| PNPO | 2.94 | 166 | 19.5 | 7.5 | 0.317730274 |
| DDB1 | 183.62 | 1140 | 126.9 | 5.26 | 0.318364512 |
| GDPGP1 | 0 | 385 | 42.3 | 6.47 | 0.318804153 |
| RNF213 | 17.41 | 5207 | 591 | 6.48 | 0.319614426 |
| ENY2 | 2.39 | 96 | 11 | 9.13 | 0.319717385 |
| PPIH | 34.18 | 177 | 19.2 | 8.07 | 0.321576952 |
| TRAPPC3 | 28.21 | 180 | 20.3 | 4.96 | 0.321744376 |
| POLR3B | 0 | 1075 | 121 | 8.56 | 0.322281662 |
| DDI2 | 11.48 | 399 | 44.5 | 5.05 | 0.322568546 |
| SLC39A7 | 5.56 | 469 | 50.1 | 6.87 | 0.32261791 |
| NXT2 | 3.46 | 197 | 22.7 | 7.11 | 0.323584149 |
| GAA | 6.99 | 952 | 105.3 | 6 | 0.32363378 |
| MAPK9 | 9.35 | 382 | 44 | 6.39 | 0.324131495 |
| RMND1 | 2.66 | 238 | 27.8 | 6.18 | 0.324238361 |
| RHOB | 64.86 | 196 | 22.1 | 5.24 | 0.324374403 |
| SACM1L | 7.08 | 587 | 66.9 | 7.12 | 0.325099557 |
| HSP90AB4P | 359.08 | 505 | 58.2 | 4.73 | 0.325830747 |
| ANKRD52 | 3.67 | 1076 | 115 | 6.48 | 0.326114677 |
| CLNS1A | 19.32 | 237 | 26.2 | 4.11 | 0.327347956 |
| MAP2K4 | 2.54 | 399 | 44.3 | 8.07 | 0.32747439 |
| MT-ATP8 | 0 | 68 | 8 | 9.91 | 0.327718792 |
| MDH1 | 275.72 | 334 | 36.4 | 7.36 | 0.327922188 |
| NFATC2IP | 3.04 | 419 | 45.8 | 6.6 | 0.328138181 |
| DTYMK | 56.65 | 188 | 21.1 | 8.75 | 0.328301867 |
| UGGT1 | 99.6 | 1531 | 174.9 | 5.6 | 0.328480211 |
| RAD51C | 3.36 | 376 | 42.2 | 6.74 | 0.329213816 |
| MRPS36 | 15.49 | 103 | 11.5 | 9.99 | 0.329424382 |
| HSD17B12 | 94.94 | 312 | 34.3 | 9.32 | 0.329433407 |
| FOXK1 | 3.01 | 570 | 59.7 | 9.32 | 0.329464029 |
| MAGED1 | 21.94 | 778 | 86.1 | 5.83 | 0.331028098 |
| ECHS1 | 116.78 | 290 | 31.4 | 8.07 | 0.331614348 |
| SNX4 | 3.47 | 305 | 35.6 | 6.95 | 0.331818744 |
| SRI | 71.98 | 180 | 20 | 5.63 | 0.331841767 |
| RABGGTA | 18.05 | 567 | 65 | 5.67 | 0.331957602 |
| FAF1 | 26.79 | 650 | 73.9 | 4.88 | 0.331963409 |
| TSPO | 19.43 | 169 | 18.8 | 9.36 | 0.331995559 |
| PUS1 | 22.7 | 399 | 44.3 | 7.71 | 0.332120009 |
| CDK2 | 46.87 | 298 | 33.9 | 8.68 | 0.332130081 |
| ARCN1 | 157.35 | 511 | 57.2 | 6.21 | 0.332894784 |
| SIGMAR1 | 11.42 | 192 | 21.5 | 5.99 | 0.333298805 |
| CCDC124 | 38.25 | 223 | 25.8 | 9.54 | 0.33333246 |
| LSM2 | 33.42 | 95 | 10.8 | 6.52 | 0.333923395 |
| KRTCAP2 | 3.67 | 136 | 14.7 | 9.61 | 0.33485588 |
| ETFB | 202.57 | 255 | 27.8 | 8.1 | 0.334985297 |
| COPS5 | 27.4 | 334 | 37.6 | 6.54 | 0.336028396 |
| SLFN11 | 13.96 | 901 | 102.8 | 7.77 | 0.336056918 |
| CCDC88A | 5.18 | 1796 | 207.7 | 5.97 | 0.336154083 |
| RPL10 | 237.01 | 214 | 24.6 | 10.08 | 0.336282753 |
| ACTR10 | 11.46 | 417 | 46.3 | 7.37 | 0.336504289 |
| NTMT1 | 26.96 | 223 | 25.4 | 5.52 | 0.336634173 |
| TSFM | 42.32 | 325 | 35.4 | 8.38 | 0.33666572 |
| DDAH1 | 2.47 | 182 | 20.2 | 5.85 | 0.337169134 |
| DHX38 | 4.58 | 1227 | 140.4 | 6.54 | 0.337574953 |
| MRPL46 | 6.03 | 279 | 31.7 | 7.05 | 0.337757895 |
| NOP53 | 9.35 | 478 | 54.4 | 10.32 | 0.337895306 |
| SRM | 128.56 | 302 | 33.8 | 5.49 | 0.338567133 |
| TFIP11 | 5.75 | 837 | 96.8 | 5.67 | 0.338804805 |
| CMTR1 | 2.91 | 835 | 95.3 | 7.05 | 0.339841282 |
| ACO2 | 154.11 | 780 | 85.4 | 7.61 | 0.33990435 |
| PLPBP | 2.02 | 275 | 30.3 | 7.5 | 0.339973366 |
| GON7 | 3.23 | 100 | 10.9 | 4.27 | 0.340071003 |
| ABCB7 | 6.1 | 712 | 78.1 | 9.13 | 0.341097751 |
| WDR75 | 36.11 | 830 | 94.4 | 5.96 | 0.343416818 |
| SCAMP2 | 11.92 | 329 | 36.6 | 6.1 | 0.343742851 |
| TXNRD2 | 47.81 | 498 | 53.9 | 6.62 | 0.344641143 |
| ITPK1 | 22.55 | 414 | 45.6 | 6.16 | 0.344911176 |
| CXADR | 10.18 | 365 | 40 | 7.56 | 0.345107795 |
| LSM12 | 6.58 | 195 | 21.7 | 7.74 | 0.345134049 |
| NAA50 | 17.85 | 169 | 19.4 | 8.81 | 0.345140019 |
| SRSF7 | 137.01 | 132 | 15.2 | 9.63 | 0.346264528 |
| NOCT | 4.22 | 431 | 48.2 | 7.23 | 0.346294703 |
| UFD1 | 61.03 | 307 | 34.5 | 6.7 | 0.346456749 |
| ATP6V1C1 | 23.63 | 382 | 43.9 | 7.46 | 0.346710402 |
| HSPA2 | 1407.18 | 639 | 70 | 5.74 | 0.347073175 |
| GPSM1 | 3.64 | 166 | 18 | 4.7 | 0.347163603 |
| COX15 | 8.87 | 388 | 43.8 | 9.64 | 0.347235883 |
| LRSAM1 | 2.85 | 696 | 80.4 | 6 | 0.348561898 |
| ELOC | 66.25 | 112 | 12.5 | 4.78 | 0.348996762 |
| MRPS9 | 40.2 | 396 | 45.8 | 9.51 | 0.348996994 |
| DYNLRB1 | 43.26 | 96 | 10.9 | 7.25 | 0.349064804 |
| CHD1L | 17.24 | 803 | 90.2 | 7.2 | 0.349801959 |
| PLS3 | 281.46 | 630 | 70.8 | 5.6 | 0.350665516 |
| GLO1 | 143.65 | 169 | 19 | 6.05 | 0.350834149 |
| COX6A1 | 5.05 | 109 | 12.1 | 9.32 | 0.351039844 |
| MAN2A1 | 26.46 | 1144 | 131.1 | 7.58 | 0.35124423 |
| RABIF | 2.61 | 123 | 13.8 | 5.52 | 0.351533949 |
| BROX | 2.62 | 411 | 46.4 | 7.65 | 0.351636783 |
| STOM | 43.31 | 288 | 31.7 | 7.88 | 0.352329752 |
| GTF2F1 | 29.7 | 517 | 58.2 | 7.49 | 0.35295727 |
| EIF2D | 12.49 | 584 | 64.7 | 7.65 | 0.353135505 |
| SGTA | 73.36 | 313 | 34 | 4.87 | 0.353938738 |
| NOP10 | 3.13 | 64 | 7.7 | 9.99 | 0.35455337 |
| SDSL | 0 | 329 | 34.7 | 6.89 | 0.35457983 |
| MCAT | 2.69 | 180 | 19.2 | 6.16 | 0.35468335 |
| PDCD6IP | 140.94 | 868 | 96 | 6.52 | 0.354797249 |
| CLTA | 87.88 | 218 | 23.6 | 4.53 | 0.354947811 |
| NSMCE2 | 2.02 | 247 | 27.9 | 7.74 | 0.356364072 |
| LAS1L | 21.08 | 717 | 81.2 | 4.69 | 0.356656301 |
| NSUN5 | 59.29 | 429 | 46.7 | 8.62 | 0.356904369 |
| ZC3H18 | 3.77 | 757 | 84 | 5.62 | 0.357403505 |
| TUBB4A | 3203.03 | 444 | 49.6 | 4.88 | 0.357804417 |
| MRPL9 | 8.28 | 267 | 30.2 | 10.08 | 0.358777365 |
| PPP1R7 | 17.01 | 360 | 41.5 | 4.91 | 0.358810035 |
| PPT1 | 53.93 | 306 | 34.2 | 6.52 | 0.358858381 |
| SPATA5L1 | 5.41 | 392 | 41.1 | 10.2 | 0.360057153 |
| MTMR6 | 6.39 | 548 | 63.7 | 7.83 | 0.361070251 |
| RWDD1 | 5.19 | 147 | 17.1 | 4.41 | 0.36125053 |
| SEC61A1 | 52.03 | 476 | 52.2 | 8.06 | 0.362336268 |
| CPSF2 | 19.57 | 782 | 88.4 | 5.11 | 0.362516918 |
| ELMSAN1 | 3 | 1045 | 114.9 | 9.19 | 0.363108437 |
| THOC3 | 6.38 | 327 | 36 | 6.83 | 0.363226409 |
| CIAPIN1 | 81.48 | 299 | 32.2 | 5.62 | 0.363300354 |
| IQGAP3 | 31.45 | 1631 | 184.6 | 7.65 | 0.363417191 |
| SKIV2L | 17.09 | 1246 | 137.7 | 6.06 | 0.363718845 |
| MOCS3 | 5.33 | 460 | 49.6 | 6.21 | 0.364147603 |
| OGFOD1 | 30.43 | 542 | 63.2 | 5.11 | 0.364519949 |
| DLAT | 108.93 | 647 | 69 | 7.84 | 0.365061231 |
| KRT73 | 25.65 | 540 | 58.9 | 7.23 | 0.365081069 |
| PABPC4 | 285.42 | 631 | 69.5 | 9.52 | 0.365140807 |
| DRAP1 | 3.35 | 205 | 22.3 | 5.17 | 0.365478017 |
| TIMM10 | 5.61 | 90 | 10.3 | 6.29 | 0.365642838 |
| KLC2 | 20.39 | 622 | 68.9 | 7.15 | 0.366782605 |
| REXO2 | 8.39 | 199 | 23.2 | 6.09 | 0.366936655 |
| ASCC2 | 2.49 | 370 | 41.8 | 4.93 | 0.367224254 |
| HP1BP3 | 25.3 | 553 | 61.2 | 9.67 | 0.367761384 |
| RRP1B | 11.61 | 740 | 82.1 | 9.8 | 0.367820324 |
| PPP2R2A | 19.17 | 447 | 51.7 | 6.2 | 0.367898722 |
| PURB | 10.75 | 312 | 33.2 | 5.43 | 0.368361641 |
| SNRNP40 | 23.82 | 357 | 39.3 | 8.1 | 0.368522917 |
| GPHN | 16.48 | 736 | 79.7 | 5.43 | 0.36853313 |
| ERLIN2 | 65.67 | 339 | 37.8 | 5.62 | 0.369070099 |
| PDE12 | 34.46 | 535 | 58.9 | 6.9 | 0.369109457 |
| YTHDF2 | 26.32 | 529 | 56.8 | 8.94 | 0.369161408 |
| IDI1 | 51.15 | 227 | 26.3 | 6.34 | 0.369191692 |
| RPIA | 20.88 | 311 | 33.2 | 8.54 | 0.369198551 |
| CTSA | 7.07 | 463 | 52.5 | 6.98 | 0.369711601 |
| RBBP4 | 118.15 | 410 | 46.1 | 5.07 | 0.36994852 |
| ISY1 | 2.24 | 285 | 33 | 5.17 | 0.370218646 |
| CPNE1 | 17.06 | 537 | 59 | 5.83 | 0.370500284 |
| METAP2 | 50.06 | 455 | 50.5 | 5.35 | 0.37082462 |
| GYS1 | 12.18 | 673 | 76.4 | 6.54 | 0.371000695 |
| TSPAN8 | 3.57 | 237 | 26 | 5.6 | 0.372473306 |
| ACADS | 12.02 | 412 | 44.3 | 7.99 | 0.372975123 |
| NFYC | 2.55 | 301 | 33.7 | 5.06 | 0.372979167 |
| CLIP2 | 13.27 | 1011 | 111.7 | 6.83 | 0.373559247 |
| EPHA5 | 9.36 | 1015 | 112.6 | 7.01 | 0.373752329 |
| BCCIP | 53.96 | 314 | 36 | 4.61 | 0.37399822 |
| DNMT1 | 48.86 | 1616 | 183 | 7.75 | 0.37466656 |
| IST1 | 13.31 | 335 | 36.6 | 5.44 | 0.374819969 |
| PPAT | 11.81 | 517 | 57.4 | 6.76 | 0.374855143 |
| CCNK | 6.39 | 357 | 41.3 | 8.15 | 0.375316832 |
| COL8A1 | 2.97 | 744 | 73.3 | 9.61 | 0.375423744 |
| TMEM43 | 42.4 | 400 | 44.8 | 8.13 | 0.376681068 |
| NUDT11 | 15.15 | 164 | 18.5 | 5.99 | 0.376689184 |
| PDHA1 | 155.09 | 390 | 43.3 | 8.06 | 0.377165987 |
| IMPA1 | 20.82 | 277 | 30.2 | 5.26 | 0.377188108 |
| SCYL1 | 2.17 | 626 | 69.2 | 8.68 | 0.377521491 |
| LRWD1 | 5.34 | 647 | 70.8 | 7.21 | 0.378373581 |
| FERMT1 | 7.07 | 495 | 56.5 | 6.86 | 0.378697412 |
| GLE1 | 2.61 | 659 | 75.4 | 7.03 | 0.378728385 |
| FTSJ3 | 65.24 | 847 | 96.5 | 8.4 | 0.379459122 |
| HLTF | 9.44 | 887 | 99.9 | 8.69 | 0.379945088 |
| SPOUT1 | 10.56 | 376 | 42 | 7.43 | 0.380046945 |
| YTHDF3 | 21.7 | 585 | 63.8 | 9.04 | 0.380544639 |
| SUPT6H | 34.58 | 1726 | 198.9 | 4.91 | 0.38102859 |
| MINDY3 | 0 | 445 | 49.7 | 4.77 | 0.381493197 |
| TBC1D10B | 0 | 233 | 26.6 | 9.95 | 0.382141937 |
| CNN2 | 41.73 | 309 | 33.7 | 7.33 | 0.382485903 |
| MRPL15 | 9.97 | 296 | 33.4 | 10.01 | 0.382785558 |
| GCSH | 11.25 | 173 | 18.9 | 4.88 | 0.38313145 |
| HNRNPUL1 | 55.44 | 756 | 84.7 | 8.78 | 0.3831645 |
| EIF3C | 202.08 | 903 | 104 | 5.63 | 0.383370985 |
| ANKRD27 | 4.67 | 1050 | 116.9 | 6.87 | 0.383459812 |
| ACE2 | 0 | 555 | 63.9 | 5.08 | 0.384792304 |
| SH3KBP1 | 65.75 | 427 | 46.6 | 8.82 | 0.384870446 |
| PYCR1 | 151.66 | 316 | 33.3 | 6.8 | 0.384992798 |
| RPL37 | 27.84 | 97 | 11.1 | 11.74 | 0.385129914 |
| CUL4B | 65.45 | 895 | 102.2 | 7.94 | 0.385258278 |
| AFDN | 5.82 | 1612 | 181.9 | 6.52 | 0.385999687 |
| MTHFD1L | 37.97 | 978 | 105.7 | 8.06 | 0.38685564 |
| NUDT16 | 4.64 | 159 | 17.7 | 6.2 | 0.388051442 |
| HNRNPLL | 35.43 | 508 | 56.4 | 7.64 | 0.388166972 |
| NDUFA10 | 11.3 | 355 | 40.7 | 8.48 | 0.388632228 |
| ACSL4 | 84.37 | 670 | 74.4 | 8.03 | 0.388881289 |
| SH3BGRL | 43.63 | 114 | 12.8 | 5.25 | 0.388935648 |
| NRAS | 14.7 | 189 | 21.2 | 5.17 | 0.389718358 |
| GRSF1 | 58.14 | 480 | 53.1 | 6.19 | 0.389911605 |
| PATL1 | 2.28 | 627 | 70.6 | 9.69 | 0.390851378 |
| CBX8 | 4.56 | 389 | 43.4 | 9.91 | 0.391306679 |
| UBR4 | 124.84 | 5159 | 571.5 | 6.04 | 0.391779355 |
| GDI1 | 279.99 | 447 | 50.6 | 5.14 | 0.392487367 |
| KRI1 | 3.17 | 703 | 82.5 | 5.14 | 0.393853015 |
| MRPL53 | 2.7 | 112 | 12.1 | 8.76 | 0.393991253 |
| PLOD3 | 82.83 | 738 | 84.7 | 6.05 | 0.394487268 |
| H1FX | 11.43 | 213 | 22.5 | 10.76 | 0.394707822 |
| ABCB10 | 24.82 | 738 | 79.1 | 9.85 | 0.395427627 |
| CORO1C | 150.36 | 474 | 53.2 | 7.08 | 0.395525628 |
| CTTNBP2NL | 4.94 | 639 | 70.1 | 8.06 | 0.395635283 |
| FKBP5 | 8.45 | 457 | 51.2 | 5.9 | 0.396542131 |
| CHMP4B | 37.94 | 224 | 24.9 | 4.82 | 0.397025783 |
| NFYB | 3.18 | 207 | 22.8 | 4.59 | 0.397267178 |
| KRR1 | 6.2 | 324 | 36.8 | 9.64 | 0.39813868 |
| MCM5 | 111.87 | 734 | 82.2 | 8.37 | 0.39814275 |
| CYP4F11 | 74.21 | 524 | 60.1 | 6.73 | 0.399281906 |
| ATXN2L | 48.48 | 1075 | 113.3 | 8.59 | 0.399289216 |
| PDXK | 15.76 | 284 | 31.8 | 5.95 | 0.399321136 |
| THADA | 5.05 | 1632 | 183.3 | 5.69 | 0.399370083 |
| THOP1 | 53.93 | 689 | 78.8 | 6.05 | 0.399463043 |
| OR1M1 | 11.3 | 313 | 34.8 | 8.91 | 0.400095775 |
| NOA1 | 6.28 | 698 | 78.4 | 8.66 | 0.400116008 |
| AAMP | 8.25 | 434 | 46.7 | 4.42 | 0.400687575 |
| IPO4 | 140.32 | 1081 | 118.6 | 4.96 | 0.401393529 |
| TSNAX | 32.54 | 290 | 33.1 | 6.55 | 0.401415594 |
| FAU | 106.68 | 59 | 6.6 | 12.15 | 0.401609699 |
| MLF2 | 2.67 | 248 | 28.1 | 6.9 | 0.40229411 |
| DCAF6 | 0 | 860 | 96.2 | 5.27 | 0.402354557 |
| CD2AP | 13.23 | 639 | 71.4 | 6.4 | 0.402882154 |
| CEBPZ | 53.98 | 1054 | 120.9 | 5.94 | 0.402999734 |
| RWDD4 | 1.91 | 188 | 21.2 | 5.31 | 0.403228706 |
| RPS18 | 383.87 | 152 | 17.7 | 10.99 | 0.4034334 |
| PPP2R5D | 36.84 | 496 | 58.4 | 6.77 | 0.403538562 |
| AP2A2 | 78.38 | 939 | 103.9 | 6.96 | 0.403716485 |
| PPP4C | 42.81 | 307 | 35.1 | 5.06 | 0.404831061 |
| DBNL | 58.15 | 431 | 48.3 | 5.05 | 0.405863427 |
| UBFD1 | 6.2 | 309 | 33.4 | 5.77 | 0.406843076 |
| RAB7A | 240 | 207 | 23.5 | 6.7 | 0.406854583 |
| BICD2 | 14.51 | 824 | 93.5 | 5.44 | 0.40686762 |
| SNAP29 | 16.69 | 258 | 29 | 5.81 | 0.408839143 |
| NDUFS6 | 9.54 | 124 | 13.7 | 8.28 | 0.408987972 |
| CFAP100 | 2.2 | 611 | 71.1 | 7.11 | 0.409402844 |
| VAT1 | 129.89 | 393 | 41.9 | 6.29 | 0.409564516 |
| GAR1 | 16.54 | 199 | 20.8 | 10.43 | 0.409692164 |
| SUMO1 | 12.48 | 101 | 11.5 | 5.52 | 0.411373621 |
| ACY1 | 7.16 | 336 | 37.6 | 5.91 | 0.411512972 |
| SNX9 | 15.85 | 595 | 66.5 | 5.58 | 0.412186576 |
| TAF10 | 11.32 | 218 | 21.7 | 6.57 | 0.412238988 |
| IARS | 433.4 | 1262 | 144.4 | 6.15 | 0.412292717 |
| BAZ1B | 49.93 | 1479 | 170.3 | 8.48 | 0.412324059 |
| NOP9 | 11.33 | 636 | 69.4 | 7.28 | 0.412654565 |
| HDAC2 | 52.68 | 458 | 52 | 5.74 | 0.412874969 |
| ELAC2 | 89.92 | 786 | 87.5 | 7.97 | 0.414029942 |
| COPB2 | 182.7 | 877 | 99 | 5.16 | 0.414107834 |
| BAZ1A | 10.4 | 1524 | 175.3 | 6.81 | 0.414248755 |
| ATL3 | 38.43 | 541 | 60.5 | 5.66 | 0.414270142 |
| RFT1 | 7.07 | 541 | 60.3 | 8.85 | 0.414724727 |
| AP3D1 | 82.95 | 1021 | 114.6 | 7.99 | 0.414819639 |
| MAGT1 | 28.2 | 335 | 38 | 9.63 | 0.415887648 |
| EIF4G2 | 119.62 | 869 | 98.1 | 6.99 | 0.416341684 |
| DCUN1D5 | 9.49 | 237 | 27.5 | 5.58 | 0.416950966 |
| PSIP1 | 22.11 | 530 | 60.1 | 9.13 | 0.41724342 |
| THOC6 | 19.52 | 296 | 32.9 | 7.24 | 0.417635461 |
| SF3A3 | 63.03 | 501 | 58.8 | 5.38 | 0.418055521 |
| NCAPD2 | 88.33 | 1401 | 157.1 | 6.61 | 0.418212514 |
| RHOF | 0 | 211 | 23.6 | 8.65 | 0.41891152 |
| EIF3K | 85.36 | 218 | 25 | 4.93 | 0.419196141 |
| FBXO22 | 29.32 | 403 | 44.5 | 7.03 | 0.419425049 |
| SCP2 | 20.54 | 466 | 50.3 | 7.02 | 0.420274731 |
| RFC4 | 38.25 | 303 | 33.7 | 9.57 | 0.420946072 |
| TRIP10 | 11.89 | 545 | 62.6 | 5.33 | 0.420947988 |
| SLC25A10 | 12.95 | 287 | 31.3 | 9.54 | 0.421242577 |
| GSPT1 | 224.42 | 636 | 68.6 | 5.33 | 0.421243981 |
| SYMPK | 22.03 | 1274 | 141.1 | 6.13 | 0.421281529 |
| PRRC2C | 37.88 | 2700 | 295.6 | 9.01 | 0.421552514 |
| ZC3H4 | 2.85 | 1303 | 140.2 | 6.27 | 0.423552771 |
| GLB1 | 10.08 | 546 | 60.5 | 6.99 | 0.424074123 |
| STX7 | 4.75 | 239 | 27.4 | 5.12 | 0.424095796 |
| UBE2G1 | 9.21 | 170 | 19.5 | 5.3 | 0.424484483 |
| STXBP1 | 24.1 | 594 | 67.5 | 6.96 | 0.42539006 |
| GLRX2 | 2.44 | 164 | 18 | 9.11 | 0.426342032 |
| MVP | 67.45 | 893 | 99.3 | 5.48 | 0.426427729 |
| CRK | 22.23 | 204 | 22.9 | 5.48 | 0.426843087 |
| C1orf167 | 2.73 | 1468 | 162.3 | 10.7 | 0.427729286 |
| DDX23 | 46.44 | 820 | 95.5 | 9.55 | 0.428383929 |
| HLA-A | 124.77 | 365 | 40.7 | 6.34 | 0.428428041 |
| MRPS28 | 10.63 | 187 | 20.8 | 9.1 | 0.428647994 |
| ACAT2 | 59.62 | 397 | 41.3 | 6.92 | 0.428777293 |
| AKAP13 | 2.92 | 2793 | 305.1 | 5.2 | 0.429429431 |
| NOS2 | 0 | 1114 | 126.7 | 7.96 | 0.429477876 |
| TSTA3 | 6.35 | 321 | 35.9 | 6.6 | 0.429824445 |
| G3BP2 | 59.38 | 449 | 50.8 | 5.38 | 0.430150924 |
| SPTLC1 | 19.5 | 473 | 52.7 | 6.01 | 0.432179119 |
| HELLS | 2.99 | 708 | 81.6 | 7.83 | 0.433163466 |
| SRRM2 | 126.78 | 2752 | 299.4 | 12.06 | 0.433284407 |
| RTTN | 2.37 | 2200 | 245.5 | 6.73 | 0.434114098 |
| YLPM1 | 9.65 | 2146 | 241.5 | 6.55 | 0.434131277 |
| DHX15 | 158.76 | 795 | 90.9 | 7.46 | 0.434199667 |
| GLS | 77.21 | 598 | 65.4 | 7.88 | 0.434892408 |
| TOR1AIP2 | 9.66 | 470 | 51.2 | 4.96 | 0.436072384 |
| DPM3 | 9.57 | 92 | 10.1 | 5.94 | 0.436520076 |
| MCM4 | 145.62 | 863 | 96.5 | 6.74 | 0.436844751 |
| AP2A1 | 112.38 | 955 | 105.3 | 7.66 | 0.436845402 |
| UGDH | 943.51 | 494 | 55 | 7.12 | 0.437158531 |
| TIPRL | 21.3 | 272 | 31.4 | 5.91 | 0.437669747 |
| NDUFA6 | 13.14 | 154 | 17.9 | 10.14 | 0.437925695 |
| RRM2B | 12.97 | 351 | 40.7 | 4.97 | 0.43827752 |
| PTCD2 | 0 | 388 | 43.9 | 9.23 | 0.438343244 |
| GTF3C1 | 11.05 | 2084 | 236.1 | 7.52 | 0.438895295 |
| PSME2 | 106.81 | 239 | 27.4 | 5.73 | 0.438995073 |
| PITPNA | 31.95 | 270 | 31.8 | 6.55 | 0.43904848 |
| AHR | 2.63 | 848 | 96.1 | 6.38 | 0.439062814 |
| CTSB | 10.67 | 339 | 37.8 | 6.3 | 0.439353822 |
| FAM227B | 0 | 304 | 36.3 | 8.59 | 0.439469526 |
| ACTN2 | 495.4 | 894 | 103.8 | 5.45 | 0.439803923 |
| DSG2 | 76.2 | 1118 | 122.2 | 5.24 | 0.440084029 |
| OSBPL11 | 0 | 747 | 83.6 | 7.06 | 0.440785536 |
| QSOX2 | 5.49 | 698 | 77.5 | 7.72 | 0.440988415 |
| TMED7 | 5 | 188 | 21.2 | 6.2 | 0.441006081 |
| MGST2 | 7.05 | 147 | 16.6 | 9.55 | 0.441155974 |
| GARS | 252.38 | 739 | 83.1 | 7.03 | 0.441510347 |
| RFC2 | 17.66 | 354 | 39.1 | 6.44 | 0.441594976 |
| LACTB2 | 8.45 | 288 | 32.8 | 6.8 | 0.441945745 |
| CNOT9 | 5.87 | 299 | 33.6 | 8.03 | 0.441957688 |
| UTP4 | 11.74 | 686 | 76.8 | 8.85 | 0.442158303 |
| CHP1 | 53.59 | 195 | 22.4 | 5.1 | 0.442210639 |
| PSMB2 | 174.39 | 201 | 22.8 | 7.02 | 0.44364165 |
| TRMT112 | 69.21 | 125 | 14.2 | 5.26 | 0.444123796 |
| DTD1 | 26.42 | 209 | 23.4 | 8.24 | 0.445409327 |
| CBR3 | 115.45 | 277 | 30.8 | 6.18 | 0.445858492 |
| MCCC2 | 47.61 | 525 | 57.5 | 7.71 | 0.446101406 |
| PCOLCE2 | 7.78 | 415 | 45.7 | 8.47 | 0.446269669 |
| PGK2 | 298.08 | 417 | 44.8 | 8.54 | 0.44636739 |
| GBA | 14.14 | 449 | 50.3 | 7.15 | 0.447448268 |
| UBE2D2 | 35.59 | 147 | 16.7 | 7.83 | 0.447765675 |
| DDX6 | 57.04 | 483 | 54.4 | 8.66 | 0.449183468 |
| RHOT2 | 14.74 | 618 | 68.1 | 5.86 | 0.449280801 |
| LSS | 11.09 | 652 | 74.2 | 6.46 | 0.449594897 |
| GIGYF2 | 30.21 | 1286 | 148.5 | 5.54 | 0.449855817 |
| FOSL1 | 12.71 | 271 | 29.4 | 8.02 | 0.450307989 |
| POLR2H | 25.18 | 150 | 17.1 | 4.68 | 0.450334907 |
| CNTD1 | 2.41 | 128 | 13.8 | 6.79 | 0.450390383 |
| NAT10 | 112.5 | 1025 | 115.7 | 8.27 | 0.450460217 |
| GYG1 | 28.14 | 333 | 37.5 | 5.4 | 0.450839528 |
| GALNT7 | 11.8 | 657 | 75.3 | 7.11 | 0.450978697 |
| NOL3 | 33.9 | 208 | 22.6 | 4.18 | 0.451279198 |
| PPP4R2 | 2.4 | 360 | 40.2 | 4.46 | 0.451747508 |
| COPS6 | 40.39 | 327 | 36.1 | 5.73 | 0.452905313 |
| FRMD8 | 2.4 | 408 | 45.1 | 6.8 | 0.453110354 |
| MRPS35 | 13.45 | 323 | 36.8 | 8.24 | 0.453586224 |
| CPNE2 | 25.13 | 446 | 49.9 | 6.25 | 0.453793886 |
| RAB34 | 28.53 | 237 | 26.7 | 6.99 | 0.455147134 |
| UBA6 | 34.6 | 1052 | 117.9 | 6.14 | 0.455276528 |
| WDR4 | 6.32 | 266 | 29.9 | 7.36 | 0.45542864 |
| DDX47 | 26.9 | 406 | 45.1 | 9.25 | 0.455472834 |
| EDEM3 | 2.63 | 905 | 101.9 | 4.94 | 0.455533345 |
| PREB | 21.65 | 417 | 45.4 | 7.88 | 0.456346582 |
| PPP3R1 | 18.12 | 170 | 19.3 | 4.81 | 0.456877504 |
| PPWD1 | 4.54 | 646 | 73.5 | 7.15 | 0.456941125 |
| SNX17 | 3.65 | 445 | 50 | 6.95 | 0.45728228 |
| MTHFS | 2.35 | 179 | 20.5 | 5.9 | 0.458020444 |
| MPI | 2.41 | 362 | 39.8 | 5.4 | 0.458083115 |
| MRPS23 | 23.74 | 190 | 21.8 | 8.9 | 0.458481534 |
| COPS4 | 59.9 | 406 | 46.2 | 5.83 | 0.458600843 |
| LSM3 | 24.45 | 102 | 11.8 | 4.7 | 0.458613234 |
| UMPS | 45.71 | 480 | 52.2 | 7.24 | 0.458943045 |
| STOML2 | 142.87 | 356 | 38.5 | 7.39 | 0.459408838 |
| FDX1 | 7.08 | 184 | 19.4 | 5.83 | 0.459591507 |
| KMT2D | 0 | 5537 | 593 | 5.58 | 0.460213325 |
| ATP2B1 | 87.51 | 1184 | 130.5 | 5.96 | 0.460273386 |
| LRRC57 | 2.45 | 239 | 26.7 | 8.43 | 0.460369421 |
| EMC10 | 4.22 | 254 | 26.8 | 6.29 | 0.460943008 |
| FAM57A | 2.15 | 225 | 25.9 | 9.52 | 0.461205601 |
| NPEPL1 | 0 | 393 | 42.1 | 9.2 | 0.461680902 |
| POGZ | 3.36 | 363 | 37.9 | 8.18 | 0.461759725 |
| DDX5 | 795.95 | 614 | 69.1 | 8.92 | 0.461929658 |
| DNAJA2 | 72.87 | 412 | 45.7 | 6.48 | 0.462229099 |
| BAG6 | 56.28 | 1077 | 113.4 | 5.69 | 0.46238638 |
| SCARA5 | 8.04 | 495 | 54 | 7.09 | 0.463010255 |
| OLA1 | 168.35 | 396 | 44.7 | 7.81 | 0.463322266 |
| HNRNPH1 | 428.41 | 449 | 49.2 | 6.3 | 0.463965966 |
| DHX57 | 5.43 | 1386 | 155.5 | 7.71 | 0.464837348 |
| ATP6V1A | 94.33 | 584 | 64.7 | 5.66 | 0.465016895 |
| MRPL49 | 18.43 | 166 | 19.2 | 9.45 | 0.465332412 |
| USP39 | 21.61 | 488 | 56.3 | 9.38 | 0.466280909 |
| SPC24 | 7.37 | 197 | 22.5 | 4.7 | 0.466452442 |
| IL1RL1 | 0 | 556 | 63.3 | 8.35 | 0.468288549 |
| DDX50 | 97.9 | 737 | 82.5 | 9.17 | 0.469156258 |
| TIMMDC1 | 6.03 | 285 | 32.2 | 8.5 | 0.469217122 |
| CMAS | 17.06 | 434 | 48.3 | 7.93 | 0.469654461 |
| P3H2 | 37.7 | 527 | 60.3 | 5.07 | 0.469760564 |
| ARPC5 | 41.51 | 151 | 16.3 | 5.67 | 0.470212773 |
| UQCRC1 | 299.42 | 480 | 52.6 | 6.37 | 0.470591455 |
| ITGB1 | 165.77 | 798 | 88.4 | 5.39 | 0.470646625 |
| ASAP2 | 38.11 | 961 | 106.9 | 6.49 | 0.470748385 |
| SEC11A | 32.34 | 164 | 19 | 8.4 | 0.471056571 |
| LAMC1 | 173.25 | 1609 | 177.5 | 5.12 | 0.471398463 |
| VRK1 | 12.03 | 396 | 45.4 | 8.91 | 0.471840857 |
| DIS3 | 21 | 958 | 108.9 | 7.14 | 0.472020511 |
| TBCA | 214.43 | 108 | 12.8 | 5.29 | 0.472268444 |
| PARD3 | 8.38 | 988 | 108.5 | 6.65 | 0.472555179 |
| GMPPB | 9.67 | 360 | 39.8 | 6.61 | 0.473042543 |
| GEMIN8 | 4.63 | 242 | 28.6 | 6.8 | 0.473442367 |
| UBE2A | 28.93 | 152 | 17.3 | 5.15 | 0.473622622 |
| DPP9 | 10.73 | 836 | 95 | 6.48 | 0.473676846 |
| CD97 | 19.73 | 742 | 81.7 | 7.05 | 0.474385607 |
| PARN | 5.99 | 578 | 66.5 | 6.38 | 0.474746788 |
| FAF2 | 68.48 | 445 | 52.6 | 5.62 | 0.476044774 |
| PFDN2 | 104.41 | 154 | 16.6 | 6.58 | 0.476046133 |
| NOSIP | 24.42 | 301 | 33.2 | 8.82 | 0.476203024 |
| C16orf58 | 3.65 | 468 | 51 | 6.93 | 0.477134851 |
| AKR1A1 | 115.66 | 325 | 36.5 | 6.79 | 0.478024862 |
| SEC22B | 114.49 | 215 | 24.6 | 6.92 | 0.478681825 |
| EXOSC2 | 16.44 | 293 | 32.8 | 7.5 | 0.479001368 |
| GVINP1 | 0 | 2422 | 278.9 | 6.55 | 0.479071562 |
| SCRIB | 21.21 | 1630 | 174.8 | 5.07 | 0.479295449 |
| SLC39A8 | 20.74 | 393 | 43.1 | 6.46 | 0.480569788 |
| CPOX | 9.7 | 454 | 50.1 | 8.25 | 0.48099848 |
| SERPINB9 | 145.16 | 376 | 42.4 | 5.86 | 0.481317729 |
| VAPB | 29.49 | 243 | 27.2 | 7.3 | 0.481772026 |
| BCKDHA | 2.34 | 445 | 50.4 | 8.27 | 0.481927554 |
| GAMT | 2.61 | 236 | 26.3 | 6.14 | 0.482132938 |
| AIFM1 | 222.15 | 609 | 66.3 | 8.94 | 0.482526422 |
| TAB1 | 2.72 | 504 | 54.6 | 5.52 | 0.482644389 |
| NT5C | 4.97 | 201 | 23.4 | 6.64 | 0.482800612 |
| CEP97 | 2.07 | 865 | 96.9 | 5.02 | 0.483534291 |
| LRPAP1 | 14.45 | 357 | 41.4 | 8.78 | 0.483750692 |
| ACAA1 | 15.95 | 424 | 44.3 | 8.44 | 0.483884297 |
| ABCF1 | 62.68 | 845 | 95.9 | 6.8 | 0.484314869 |
| CDK5 | 27.6 | 292 | 33.3 | 7.66 | 0.484369996 |
| TNFAIP3 | 8.31 | 790 | 89.6 | 8.22 | 0.485036637 |
| ACADM | 37.62 | 421 | 46.6 | 8.37 | 0.485261608 |
| NUP43 | 17.9 | 380 | 42.1 | 5.63 | 0.485285938 |
| KDELR1 | 19.63 | 212 | 24.5 | 8.62 | 0.486005909 |
| RBM7 | 5.95 | 266 | 30.5 | 9.57 | 0.486576765 |
| ELP1 | 32.65 | 1332 | 150.2 | 5.94 | 0.486716013 |
| FTL | 3.88 | 175 | 20 | 5.78 | 0.487178511 |
| POLDIP2 | 29.78 | 368 | 42 | 8.63 | 0.487402451 |
| IMMT | 278.4 | 747 | 82.6 | 6.57 | 0.487824722 |
| IRS2 | 8.33 | 1338 | 137.2 | 8.65 | 0.488475309 |
| RAB9A | 4.18 | 201 | 22.8 | 5.47 | 0.488531509 |
| UQCR11 | 14.84 | 56 | 6.6 | 9.88 | 0.488587471 |
| NT5DC3 | 2.15 | 464 | 54.6 | 7.11 | 0.488604777 |
| PPP1R12A | 24.34 | 971 | 109 | 5.41 | 0.488660005 |
| MMTAG2 | 2.29 | 141 | 15.5 | 9.14 | 0.488687982 |
| NOTCH2 | 2.07 | 2471 | 265.2 | 5.14 | 0.488793186 |
| ABCC2 | 2.69 | 1545 | 174.1 | 8.32 | 0.488816128 |
| NDUFA4 | 39.76 | 81 | 9.4 | 9.38 | 0.489079252 |
| PDHX | 7.2 | 486 | 51.4 | 6.34 | 0.489308739 |
| CCDC96 | 1.74 | 555 | 62.7 | 4.94 | 0.490277638 |
| TBCB | 131.07 | 244 | 27.3 | 5.15 | 0.490362652 |
| MOGS | 40.32 | 731 | 80.7 | 7.11 | 0.49049066 |
| CNOT2 | 0 | 365 | 40.8 | 6.25 | 0.490853303 |
| SDHA | 145.2 | 664 | 72.6 | 7.39 | 0.492217239 |
| GABPB1 | 2.51 | 348 | 36.9 | 4.87 | 0.492227533 |
| ITPA | 23.03 | 177 | 19.6 | 5.19 | 0.492886498 |
| SLC25A15 | 2.05 | 301 | 32.7 | 9.13 | 0.493121033 |
| EFL1 | 6.25 | 1120 | 125.3 | 5.91 | 0.493491985 |
| PYCR2 | 119.45 | 320 | 33.6 | 7.77 | 0.493742986 |
| AAAS | 9.93 | 513 | 55.8 | 7.33 | 0.493809757 |
| BIRC6 | 11.18 | 4857 | 529.9 | 6.05 | 0.494246864 |
| TXLNG | 14.49 | 528 | 60.5 | 7.52 | 0.49442766 |
| SARS | 52.38 | 514 | 58.7 | 6.43 | 0.494564671 |
| AKT1 | 13.84 | 418 | 48.3 | 5.76 | 0.495190089 |
| PPIL4 | 2.71 | 492 | 57.2 | 5.92 | 0.495211425 |
| UBAP2L | 191.4 | 976 | 103.1 | 6.87 | 0.495466809 |
| HSPBP1 | 35.63 | 362 | 39.4 | 5.21 | 0.495746443 |
| GPAA1 | 3.36 | 561 | 61.1 | 7.65 | 0.495900472 |
| RNMT | 4.92 | 476 | 54.8 | 6.61 | 0.495907637 |
| LUC7L2 | 111.54 | 392 | 46.5 | 10.01 | 0.496981695 |
| TUBA1C | 2753.94 | 449 | 49.9 | 5.1 | 0.497011025 |
| MPRIP | 25.12 | 1000 | 114 | 6.35 | 0.497392408 |
| SEC11C | 2.58 | 192 | 21.5 | 9.2 | 0.497517073 |
| CD320 | 6.89 | 240 | 24.4 | 4.81 | 0.497799592 |
| TMTC3 | 10.36 | 914 | 103.8 | 8.84 | 0.49795721 |
| UQCRC2 | 273.87 | 453 | 48.4 | 8.63 | 0.498327183 |
| UBAP2 | 22.76 | 1119 | 117 | 7.34 | 0.499316534 |
| SON | 26.98 | 2386 | 259.4 | 5.73 | 0.499502253 |
| WDR60 | 2.19 | 1066 | 122.5 | 7.31 | 0.499587596 |
| EMC7 | 10.02 | 242 | 26.5 | 9.25 | 0.499772179 |
| AP4S1 | 3.13 | 144 | 17 | 5.19 | 0.501963217 |
| AMPD2 | 16.01 | 798 | 92 | 6.28 | 0.502102997 |
| TFCP2 | 7.16 | 450 | 51.3 | 5.44 | 0.502135969 |
| GEMIN4 | 16.54 | 1058 | 120 | 6.04 | 0.503324553 |
| PRMT3 | 13.1 | 469 | 52.8 | 6.19 | 0.503765411 |
| PITRM1 | 32.21 | 1037 | 117.3 | 6.92 | 0.503934544 |
| PSPC1 | 38.26 | 393 | 45.5 | 6.58 | 0.50398943 |
| SLTM | 0 | 1034 | 117.1 | 7.87 | 0.504077248 |
| TIMM44 | 55.23 | 452 | 51.3 | 8.32 | 0.504292303 |
| COPE | 22.54 | 257 | 28.8 | 5.29 | 0.504690901 |
| LASP1 | 228.93 | 261 | 29.7 | 7.05 | 0.505202291 |
| CAB39 | 11.2 | 341 | 39.8 | 6.89 | 0.505467196 |
| KIF2A | 16.98 | 660 | 75 | 6.4 | 0.505496663 |
| DNAAF5 | 92.47 | 855 | 93.5 | 6.42 | 0.506151296 |
| BYSL | 64.31 | 437 | 49.6 | 8.12 | 0.50642279 |
| PURA | 11.16 | 322 | 34.9 | 6.44 | 0.506890895 |
| CCDC86 | 4.46 | 360 | 40.2 | 10.33 | 0.50761037 |
| PRPS1 | 204.95 | 318 | 34.8 | 6.98 | 0.507745689 |
| SDF2L1 | 27.94 | 221 | 23.6 | 7.03 | 0.50777098 |
| TGOLN2 | 4.85 | 379 | 39.5 | 5.59 | 0.508026905 |
| EXOC5 | 8.34 | 708 | 81.8 | 6.71 | 0.508129074 |
| HMGCS1 | 36.72 | 520 | 57.3 | 5.41 | 0.508198913 |
| PGM1 | 53.77 | 562 | 61.4 | 6.76 | 0.508236904 |
| GNB1 | 126.46 | 340 | 37.4 | 6 | 0.508249694 |
| CRYZ | 10.92 | 295 | 31.5 | 8.47 | 0.508564963 |
| ASL | 2.22 | 438 | 48.7 | 6.13 | 0.508958596 |
| GRN | 10.74 | 593 | 63.5 | 6.83 | 0.509085034 |
| ZRANB2 | 13.24 | 320 | 36.3 | 9.76 | 0.509251835 |
| NAA30 | 3.19 | 325 | 35.6 | 5.77 | 0.509794988 |
| SQLE | 2.46 | 574 | 63.9 | 8.63 | 0.51008111 |
| ICOSLG | 0 | 185 | 20.1 | 5.83 | 0.510101918 |
| JTB | 2.91 | 117 | 13.3 | 8.31 | 0.510350478 |
| CSDE1 | 227.19 | 798 | 88.8 | 6.25 | 0.51040435 |
| PCM1 | 0 | 530 | 59.2 | 5.85 | 0.510471163 |
| PSMA6 | 223.55 | 246 | 27.4 | 6.76 | 0.510531316 |
| EHD4 | 85.23 | 541 | 61.1 | 6.76 | 0.510709877 |
| SNU13 | 77.89 | 128 | 14.2 | 8.46 | 0.5111865 |
| USP15 | 45.78 | 952 | 109.2 | 5.15 | 0.511217755 |
| CAPG | 53.3 | 333 | 36.8 | 7.2 | 0.512992512 |
| CLPB | 23.74 | 677 | 75.4 | 9.01 | 0.513047446 |
| VPS4A | 12.39 | 437 | 48.9 | 7.8 | 0.513131837 |
| RAB3D | 131.64 | 219 | 24.3 | 4.93 | 0.513471722 |
| ARL8B | 29.94 | 186 | 21.5 | 8.43 | 0.514126653 |
| BUB1 | 0 | 1028 | 115.8 | 6.55 | 0.51487802 |
| TMPO | 248.95 | 454 | 50.6 | 9.38 | 0.515339632 |
| DDX39B | 371.53 | 428 | 49 | 5.67 | 0.515505966 |
| PSMD2 | 279.23 | 908 | 100.1 | 5.2 | 0.516430239 |
| PES1 | 78.13 | 583 | 67.4 | 7.53 | 0.517170413 |
| THEM6 | 6.03 | 208 | 23.8 | 9.55 | 0.518122918 |
| SPECC1L | 9.35 | 1078 | 120.1 | 5.76 | 0.518321773 |
| INTS3 | 15.95 | 1042 | 117.9 | 5.8 | 0.518408142 |
| EIF2B2 | 16.25 | 351 | 39 | 6.16 | 0.518453947 |
| ESS2 | 3.7 | 476 | 52.5 | 7.56 | 0.519562047 |
| SPATS2 | 12.61 | 545 | 59.5 | 8.9 | 0.521946413 |
| ERAP1 | 6.78 | 941 | 107.2 | 6.46 | 0.521953205 |
| RPL21 | 132.57 | 160 | 18.6 | 10.49 | 0.522134545 |
| ARL3 | 50.71 | 182 | 20.4 | 7.24 | 0.522261736 |
| NPC1 | 8.49 | 1278 | 142.1 | 5.36 | 0.522297283 |
| CFAP36 | 3.3 | 342 | 39.4 | 4.97 | 0.522699393 |
| TSSC4 | 3.4 | 265 | 27.9 | 7.11 | 0.522833854 |
| UBXN7 | 2.35 | 489 | 54.8 | 5.16 | 0.523179002 |
| MPG | 21.66 | 281 | 30.8 | 9.16 | 0.524152986 |
| RPS23 | 229.9 | 143 | 15.8 | 10.49 | 0.524615709 |
| SPAG9 | 95.44 | 1307 | 144.6 | 5.17 | 0.524991879 |
| ELP2 | 6.57 | 705 | 78.5 | 5.68 | 0.525533257 |
| SENP1 | 2.99 | 643 | 73.3 | 8.56 | 0.526574426 |
| HAT1 | 22.07 | 334 | 39.8 | 5.92 | 0.527357618 |
| PSMB4 | 119.01 | 264 | 29.2 | 5.97 | 0.52838215 |
| POR | 97.17 | 677 | 76.6 | 5.58 | 0.529208327 |
| CD151 | 44.16 | 253 | 28.3 | 7.47 | 0.5293277 |
| ZCRB1 | 8.36 | 217 | 24.6 | 8.53 | 0.530017203 |
| EPB41L3 | 433.89 | 883 | 98.4 | 5.34 | 0.530261081 |
| CREG1 | 8.44 | 220 | 24.1 | 7.59 | 0.530656926 |
| CAPNS1 | 109.58 | 268 | 28.3 | 5.2 | 0.530665326 |
| WASH3P | 7.27 | 463 | 50 | 5.49 | 0.530956417 |
| S100A16 | 3 | 103 | 11.8 | 6.79 | 0.531228017 |
| TOMM40 | 58 | 361 | 37.9 | 7.25 | 0.532053423 |
| PLIN3 | 171.7 | 422 | 45.8 | 5.43 | 0.532274301 |
| ELP3 | 15.36 | 533 | 60.6 | 8.73 | 0.533290594 |
| ETF1 | 195.5 | 437 | 49 | 5.71 | 0.53407584 |
| ANAPC7 | 22.29 | 537 | 60 | 8.41 | 0.534202013 |
| ISCA2 | 3.19 | 154 | 16.5 | 5.25 | 0.534628572 |
| CACNA1A | 0 | 2240 | 254.2 | 8.35 | 0.534852887 |
| ANAPC2 | 3.98 | 819 | 93.4 | 5.25 | 0.534935648 |
| DHX9 | 1067.42 | 1270 | 140.9 | 6.84 | 0.535034678 |
| INPP5K | 3.12 | 372 | 42.8 | 6.47 | 0.537573358 |
| TRMT11 | 0 | 257 | 29.2 | 6.84 | 0.537685667 |
| ARMC1 | 9.05 | 282 | 31.3 | 5.74 | 0.537911254 |
| TOMM6 | 18.46 | 74 | 8 | 4.89 | 0.538063886 |
| RPA2 | 22.76 | 270 | 29.2 | 6.15 | 0.538358302 |
| ZNF326 | 4.59 | 582 | 65.6 | 5.15 | 0.538662316 |
| MRPL45 | 16.06 | 306 | 35.3 | 9.03 | 0.538898067 |
| CARHSP1 | 12.22 | 147 | 15.9 | 8.21 | 0.539201121 |
| ASCC3 | 32.9 | 2202 | 251.3 | 7.09 | 0.539309855 |
| RPS4X | 607.29 | 263 | 29.6 | 10.15 | 0.539560497 |
| ADAR | 194.18 | 931 | 103.6 | 8.66 | 0.539681512 |
| HIST3H2BB | 1207.24 | 126 | 13.9 | 10.32 | 0.540939013 |
| GOLGA1 | 3.67 | 767 | 88.1 | 5.27 | 0.54111039 |
| GGCT | 32.86 | 188 | 21 | 5.14 | 0.541567072 |
| TOP2B | 150.86 | 1621 | 182.5 | 8.06 | 0.54282362 |
| TPM1 | 428.35 | 245 | 28.4 | 4.77 | 0.544368284 |
| FASTKD5 | 13.03 | 764 | 86.5 | 8.13 | 0.54475972 |
| CHID1 | 5.72 | 362 | 41.7 | 8.63 | 0.545068132 |
| BCS1L | 16.12 | 419 | 47.5 | 8.5 | 0.545174426 |
| ACP1 | 122.78 | 158 | 18 | 6.74 | 0.545189758 |
| VAMP2 | 31.68 | 116 | 12.7 | 8.13 | 0.545605292 |
| ANXA4 | 165.89 | 319 | 35.9 | 6.13 | 0.546055793 |
| PIGS | 15.38 | 547 | 61 | 6.43 | 0.546751397 |
| LZIC | 11.97 | 190 | 21.5 | 4.94 | 0.546990942 |
| MAK16 | 15.65 | 300 | 35.3 | 5.38 | 0.547045472 |
| TOR3A | 2.99 | 336 | 38.8 | 6.28 | 0.547081419 |
| AKR1C1 | 1062.04 | 323 | 36.8 | 7.88 | 0.547281088 |
| SLITRK1 | 0 | 696 | 77.7 | 6.44 | 0.547985054 |
| M1AP | 5.09 | 365 | 41 | 6.23 | 0.549126311 |
| GLYR1 | 10.08 | 484 | 52.5 | 8.82 | 0.54964219 |
| TUBB3 | 2068.45 | 450 | 50.4 | 4.93 | 0.549802753 |
| DBN1 | 102.71 | 649 | 71.4 | 4.45 | 0.550084378 |
| SERPINE2 | 37.15 | 397 | 44 | 9.38 | 0.551123467 |
| LYPLA2 | 10.69 | 231 | 24.7 | 7.23 | 0.551303452 |
| GBE1 | 68.51 | 702 | 80.4 | 6.32 | 0.551573307 |
| NUDT2 | 7.11 | 147 | 16.8 | 5.35 | 0.552060631 |
| LCP1 | 101.85 | 627 | 70.2 | 5.43 | 0.552095803 |
| PSME4 | 19.23 | 1843 | 211.2 | 6.9 | 0.552387769 |
| FAM3C | 10.87 | 227 | 24.7 | 8.29 | 0.55249274 |
| ELMO2 | 10.61 | 720 | 82.6 | 5.9 | 0.553010948 |
| TRIM25 | 38.66 | 630 | 70.9 | 8.09 | 0.553131688 |
| ABCE1 | 152.17 | 599 | 67.3 | 8.34 | 0.553692149 |
| VPS29 | 65.68 | 182 | 20.5 | 6.79 | 0.553799908 |
| APIP | 3.58 | 204 | 22.8 | 6.68 | 0.554151255 |
| XAB2 | 21.82 | 855 | 99.9 | 6.23 | 0.555476359 |
| SQOR | 4.9 | 450 | 49.9 | 9.11 | 0.555797004 |
| AGK | 64.34 | 422 | 47.1 | 8.09 | 0.555819163 |
| ZC3H11A | 25.23 | 810 | 89.1 | 8.37 | 0.556049998 |
| LAGE3 | 15.24 | 143 | 14.8 | 8.63 | 0.556475008 |
| SMC1A | 44.01 | 1233 | 143.1 | 7.64 | 0.556752209 |
| TMA16 | 4.84 | 203 | 23.8 | 9.26 | 0.556996226 |
| GLA | 20.04 | 429 | 48.7 | 5.6 | 0.557371181 |
| PDLIM7 | 14.13 | 153 | 16 | 9.79 | 0.557515202 |
| ENDOG | 0 | 297 | 32.6 | 9.5 | 0.55752903 |
| ANXA6 | 367.78 | 641 | 72.4 | 5.6 | 0.557582548 |
| HSPA4L | 327.01 | 839 | 94.5 | 5.88 | 0.557890425 |
| QKI | 5.2 | 319 | 35.2 | 7.43 | 0.55806855 |
| EFEMP1 | 32.13 | 355 | 39.2 | 6.49 | 0.559026809 |
| PHF5A | 32.02 | 110 | 12.4 | 8.41 | 0.559226678 |
| SCAF11 | 2.78 | 1148 | 128.8 | 8.29 | 0.55989366 |
| NUP93 | 204.11 | 819 | 93.4 | 5.72 | 0.56069568 |
| DSC2 | 2.3 | 847 | 93.7 | 5.47 | 0.561543762 |
| PCMT1 | 62.6 | 227 | 24.6 | 7.21 | 0.561779072 |
| HOOK3 | 8.28 | 718 | 83.1 | 5.17 | 0.561854088 |
| RIT1 | 3.82 | 183 | 21.6 | 9.09 | 0.562337644 |
| METTL14 | 3.76 | 456 | 52.1 | 6.21 | 0.562712133 |
| LSM7 | 11.72 | 103 | 11.6 | 5.27 | 0.562831335 |
| SLC2A1 | 63.13 | 492 | 54 | 8.72 | 0.563686401 |
| MINOS1 | 2.19 | 24 | 2.8 | 5.02 | 0.563805359 |
| HSD17B4 | 147.63 | 736 | 79.6 | 8.84 | 0.564817469 |
| TTC27 | 0 | 843 | 96.6 | 5.59 | 0.565365761 |
| UPF3B | 2.14 | 470 | 56.2 | 9.42 | 0.565511857 |
| MAP4K4 | 2.67 | 1165 | 133.3 | 7.65 | 0.565591107 |
| ITPRIP | 8.51 | 547 | 62 | 5.88 | 0.566111687 |
| NIT2 | 56.83 | 276 | 30.6 | 7.21 | 0.566242928 |
| GTF3C3 | 22.5 | 886 | 101.2 | 5.07 | 0.567086145 |
| CISD3 | 0 | 127 | 14.2 | 10.55 | 0.567538509 |
| SRPRA | 35.81 | 610 | 66.5 | 9.04 | 0.568218611 |
| HLA-C | 128.5 | 366 | 40.9 | 6.3 | 0.568403035 |
| DNAJC7 | 99.42 | 494 | 56.4 | 6.96 | 0.56858284 |
| ENO2 | 735.97 | 391 | 42.7 | 5.12 | 0.568665445 |
| NPTN | 21.88 | 278 | 30.8 | 7.84 | 0.569273358 |
| SH3GL1 | 5.88 | 368 | 41.5 | 5.43 | 0.569482855 |
| DDX41 | 11.73 | 622 | 69.8 | 6.84 | 0.569713378 |
| SEC23B | 49.94 | 767 | 86.4 | 6.89 | 0.569761641 |
| RMDN3 | 18.25 | 470 | 52.1 | 5.1 | 0.570081178 |
| WDR33 | 3.34 | 1336 | 145.8 | 9.17 | 0.570284069 |
| ARFGEF3 | 0 | 2177 | 240.5 | 5.82 | 0.570314967 |
| CIRBP | 26.15 | 172 | 18.6 | 9.51 | 0.571049145 |
| NSF | 55.25 | 744 | 82.5 | 6.95 | 0.571150527 |
| NFS1 | 5.62 | 397 | 44 | 7.11 | 0.571305854 |
| SMC4 | 47.97 | 1288 | 147.1 | 6.79 | 0.571330943 |
| PSMD1 | 192.18 | 922 | 102.2 | 5.27 | 0.571597619 |
| UAP1 | 12.59 | 505 | 57 | 6.38 | 0.571922869 |
| PDK1 | 3.31 | 436 | 49.2 | 8.81 | 0.572595456 |
| COA3 | 5.64 | 106 | 11.7 | 9.6 | 0.572732715 |
| SEPT7 | 194.59 | 436 | 50.5 | 8.63 | 0.572920376 |
| EWSR1 | 74.66 | 583 | 61.2 | 9.07 | 0.57400443 |
| ODR4 | 3.98 | 335 | 37.7 | 8.21 | 0.574244707 |
| NOC4L | 12.7 | 516 | 58.4 | 7.49 | 0.575400532 |
| POLR1A | 38.77 | 1720 | 194.7 | 7.03 | 0.575680954 |
| PRKDC | 648.24 | 4128 | 468.8 | 7.12 | 0.576834938 |
| GART | 292.59 | 1010 | 107.7 | 6.7 | 0.576962772 |
| RPL32 | 132.36 | 135 | 15.8 | 11.33 | 0.577386597 |
| CASC3 | 2.43 | 703 | 76.2 | 6.48 | 0.578057765 |
| NPEPPS | 268.93 | 919 | 103.2 | 5.72 | 0.578118439 |
| CAP2 | 11.71 | 477 | 52.8 | 6.37 | 0.578716003 |
| DDX3Y | 520.34 | 660 | 73.1 | 7.55 | 0.579692592 |
| DBR1 | 5.93 | 544 | 61.5 | 5.47 | 0.579826457 |
| PGP | 56.86 | 321 | 34 | 6.14 | 0.580586845 |
| TMOD3 | 59.87 | 352 | 39.6 | 5.19 | 0.581090008 |
| TOR1AIP1 | 61.65 | 584 | 66.3 | 8.18 | 0.58206217 |
| UPF1 | 72.49 | 1118 | 123 | 6.68 | 0.582194206 |
| MCM6 | 135.91 | 821 | 92.8 | 5.41 | 0.582819595 |
| MRPL44 | 50.14 | 332 | 37.5 | 8.4 | 0.582967207 |
| CHCHD2 | 2.99 | 151 | 15.5 | 9.22 | 0.583199043 |
| ACTR1A | 47.17 | 376 | 42.6 | 6.64 | 0.583432322 |
| EXOSC6 | 19.44 | 272 | 28.2 | 6.28 | 0.584343807 |
| BABAM2 | 12.28 | 376 | 42.7 | 5.44 | 0.584397995 |
| POLR2J | 10.59 | 117 | 13.3 | 5.86 | 0.584404024 |
| LIMD1 | 0 | 676 | 72.1 | 6.65 | 0.584627812 |
| ANAPC1 | 2.51 | 1944 | 216.4 | 6.3 | 0.584878875 |
| NDRG3 | 26.31 | 363 | 40 | 5.39 | 0.585184 |
| MB21D2 | 0 | 491 | 55.8 | 7.03 | 0.585221171 |
| SCYL2 | 5.4 | 929 | 103.6 | 8.22 | 0.586236059 |
| NCSTN | 32.53 | 689 | 76.7 | 5.68 | 0.587197741 |
| IRF3 | 2.18 | 154 | 16.7 | 4.91 | 0.587884266 |
| SNX3 | 22.16 | 162 | 18.8 | 8.66 | 0.588001843 |
| HK1 | 227.31 | 905 | 101 | 6.9 | 0.588168566 |
| KIF3B | 12.22 | 747 | 85.1 | 7.69 | 0.588282258 |
| RPSA | 513.65 | 295 | 32.8 | 4.87 | 0.588530011 |
| VPS16 | 2.56 | 695 | 78.3 | 7.87 | 0.588568512 |
| RBM27 | 24.41 | 1060 | 118.6 | 9.19 | 0.589836594 |
| PRKAR2A | 24.15 | 382 | 43 | 5.08 | 0.589899842 |
| SQSTM1 | 105.44 | 440 | 47.7 | 5.22 | 0.589993035 |
| PDCD11 | 79.22 | 1871 | 208.6 | 8.87 | 0.590530716 |
| PAICS | 268.92 | 425 | 47 | 7.23 | 0.591538734 |
| USO1 | 77.92 | 962 | 107.8 | 4.91 | 0.591670666 |
| FH | 305.81 | 467 | 50.2 | 7.4 | 0.591791893 |
| CHUK | 5.59 | 745 | 84.6 | 6.73 | 0.592524346 |
| MRPL27 | 6.84 | 148 | 16.1 | 10.42 | 0.59274061 |
| VASP | 24.85 | 380 | 39.8 | 8.94 | 0.59366711 |
| PIP4K2C | 4.02 | 403 | 45 | 6.48 | 0.593845556 |
| TRMT6 | 15.48 | 497 | 55.8 | 7.55 | 0.59438489 |
| SORBS2 | 4.84 | 644 | 71.6 | 9.48 | 0.594845855 |
| SLC7A2 | 6.81 | 658 | 71.6 | 7.28 | 0.595102475 |
| OXA1L | 16.41 | 419 | 46.7 | 9.25 | 0.595539158 |
| EPRS | 610.73 | 1512 | 170.5 | 7.33 | 0.59558096 |
| SREK1 | 3.84 | 508 | 59.3 | 10.39 | 0.595597447 |
| PTPN23 | 10.62 | 1636 | 178.9 | 6.92 | 0.595621628 |
| CTSC | 116.27 | 463 | 51.8 | 6.99 | 0.595729678 |
| IER3IP1 | 9.57 | 82 | 9 | 8.22 | 0.596484886 |
| GCLC | 19.64 | 637 | 72.7 | 6.09 | 0.597126568 |
| RAB11B | 178.66 | 218 | 24.5 | 5.94 | 0.597387795 |
| GLTP | 5.67 | 209 | 23.8 | 7.39 | 0.597499301 |
| LIMCH1 | 42.22 | 1056 | 118.9 | 6.3 | 0.598477249 |
| COX17 | 13.06 | 63 | 6.9 | 7.24 | 0.598604701 |
| TBPL1 | 3.26 | 186 | 20.9 | 9.54 | 0.599064979 |
| PGLS | 44.59 | 258 | 27.5 | 6.05 | 0.599147549 |
| ITIH2 | 4.97 | 946 | 106.4 | 6.86 | 0.599791528 |
| UBQLN2 | 108.57 | 624 | 65.7 | 5.22 | 0.60031333 |
| MTIF2 | 10.74 | 727 | 81.3 | 7.15 | 0.600314588 |
| EED | 0 | 400 | 45.5 | 6.71 | 0.600653662 |
| CASK | 7.86 | 897 | 102 | 6.35 | 0.600804651 |
| SMARCC2 | 33.07 | 1130 | 124.8 | 5.55 | 0.601285584 |
| MZT2A | 17.75 | 158 | 16.2 | 9.64 | 0.601959145 |
| NRDC | 66.9 | 1150 | 131.5 | 5 | 0.603379464 |
| NAPG | 12.19 | 312 | 34.7 | 5.41 | 0.603406239 |
| TMTC4 | 2.26 | 612 | 68.3 | 8.78 | 0.60393481 |
| NUP98 | 31.09 | 1714 | 186.2 | 6.29 | 0.604458126 |
| HPS5 | 3.93 | 1015 | 114.8 | 5.39 | 0.604473994 |
| MAD2L1 | 13.14 | 205 | 23.5 | 5.08 | 0.605246447 |
| PFKL | 176.35 | 780 | 85 | 7.5 | 0.605378602 |
| FASTKD2 | 13.05 | 648 | 74.5 | 7.99 | 0.60545279 |
| FKBP10 | 267.94 | 582 | 64.2 | 5.62 | 0.605634881 |
| MTA2 | 27.79 | 668 | 75 | 9.66 | 0.606618452 |
| WDR36 | 48.67 | 951 | 105.3 | 7.53 | 0.606792138 |
| TNPO1 | 354.07 | 890 | 101.2 | 4.96 | 0.607269204 |
| AKR7A2 | 10.68 | 359 | 39.6 | 7.17 | 0.607668464 |
| AAGAB | 0 | 315 | 34.6 | 4.64 | 0.607688591 |
| SBNO1 | 12.42 | 1391 | 154 | 7.77 | 0.608150689 |
| DAD1 | 28.23 | 113 | 12.5 | 7.08 | 0.608309653 |
| NDUFA7 | 7.8 | 113 | 12.5 | 10.18 | 0.608634612 |
| TRNT1 | 4.97 | 414 | 47.8 | 8.48 | 0.608728284 |
| HPF1 | 4.25 | 346 | 39.4 | 6.8 | 0.609151211 |
| CD276 | 14.13 | 493 | 52.7 | 5.52 | 0.610704823 |
| ETHE1 | 14.59 | 254 | 27.9 | 6.83 | 0.611183514 |
| COL5A2 | 37.66 | 1499 | 144.8 | 6.46 | 0.611591647 |
| CRYZL1 | 2.51 | 198 | 21.9 | 5.78 | 0.612030064 |
| SLC29A1 | 15.12 | 456 | 50.2 | 8.29 | 0.612271334 |
| PHKB | 13.49 | 1087 | 124 | 6.67 | 0.613231823 |
| NUP153 | 10.36 | 1475 | 153.8 | 8.73 | 0.613232559 |
| SUPV3L1 | 4.83 | 786 | 87.9 | 7.99 | 0.613345009 |
| EDC4 | 124.86 | 1401 | 151.6 | 5.86 | 0.613640772 |
| PRPF19 | 65.58 | 504 | 55.1 | 6.61 | 0.614131683 |
| HNRNPH3 | 160.58 | 331 | 35.2 | 6.87 | 0.614159619 |
| DDB2 | 0 | 238 | 26.7 | 9.61 | 0.614665885 |
| OAS1 | 46 | 364 | 41.7 | 8.91 | 0.615357731 |
| CALD1 | 25.15 | 532 | 61.2 | 6.71 | 0.615418443 |
| TLR10 | 0 | 811 | 94.5 | 6.79 | 0.615905989 |
| SPECC1 | 3.14 | 703 | 79 | 5.34 | 0.61627252 |
| APOO | 14.04 | 180 | 20.2 | 9.32 | 0.616321025 |
| HTATSF1 | 40.9 | 755 | 85.8 | 4.4 | 0.616451552 |
| VCAN | 2.62 | 1642 | 181.9 | 4.89 | 0.617110074 |
| TBC1D15 | 10.7 | 674 | 77.3 | 5.39 | 0.61740321 |
| KIFC3 | 40.8 | 687 | 77 | 6.65 | 0.617425202 |
| CTCFL | 0 | 299 | 35.4 | 9.79 | 0.617425625 |
| COA6 | 25.14 | 79 | 9.4 | 7.74 | 0.617468776 |
| CAND1 | 331.89 | 1230 | 136.3 | 5.78 | 0.617565254 |
| NES | 27.29 | 1621 | 177.3 | 4.36 | 0.618691234 |
| COASY | 26.9 | 564 | 62.3 | 6.99 | 0.618868017 |
| SPCS2 | 26.13 | 226 | 25 | 8.47 | 0.620031437 |
| PRDX5 | 183.53 | 162 | 17 | 7.24 | 0.620226245 |
| XPC | 5.33 | 903 | 101.8 | 9.1 | 0.620267918 |
| AACS | 13.59 | 672 | 75.1 | 6.24 | 0.620877202 |
| STAT6 | 14.14 | 737 | 81.7 | 6.24 | 0.620946288 |
| ALDH4A1 | 2.77 | 503 | 55.1 | 6.8 | 0.62181438 |
| ENG | 5.32 | 625 | 67.5 | 6.57 | 0.621981711 |
| COLGALT1 | 74.63 | 622 | 71.6 | 7.31 | 0.622538904 |
| TXNDC9 | 7.41 | 188 | 22.2 | 5.83 | 0.622625904 |
| PDCD5 | 82.33 | 125 | 14.3 | 6.04 | 0.622921013 |
| CD2BP2 | 13.48 | 341 | 37.6 | 4.61 | 0.623718006 |
| POLDIP3 | 12.21 | 392 | 42.9 | 9.91 | 0.623981861 |
| GTF2F2 | 11.2 | 249 | 28.4 | 9.23 | 0.624258934 |
| MRPL23 | 15.94 | 153 | 17.8 | 9.69 | 0.62502177 |
| CDV3 | 29.54 | 258 | 27.3 | 6.4 | 0.625872057 |
| NFIC | 2.63 | 406 | 45.4 | 8.82 | 0.625946218 |
| TPR | 161.33 | 2363 | 267.1 | 5.02 | 0.626633156 |
| KRAS | 16.5 | 188 | 21.4 | 8.12 | 0.626754622 |
| MTAP | 83.85 | 283 | 31.2 | 7.18 | 0.626883677 |
| TIMM50 | 44.72 | 353 | 39.6 | 8.37 | 0.626991525 |
| SUMO2 | 79.45 | 71 | 8.1 | 5.41 | 0.627806377 |
| RBFOX2 | 7.32 | 367 | 39.5 | 6.65 | 0.628071259 |
| SLC26A2 | 3.65 | 739 | 81.6 | 8.38 | 0.628158876 |
| PMM2 | 22.96 | 246 | 28.1 | 6.77 | 0.628328815 |
| PDE3A | 7.1 | 1141 | 124.9 | 6 | 0.628487027 |
| MPST | 19.74 | 297 | 33.2 | 6.6 | 0.629042848 |
| RENBP | 2.33 | 427 | 48.8 | 6.37 | 0.629258898 |
| DYNC1LI2 | 41.57 | 415 | 45 | 6.14 | 0.629951268 |
| TANC1 | 4.88 | 1755 | 191.3 | 8.62 | 0.630359985 |
| NVL | 8.59 | 765 | 85 | 6.2 | 0.630493447 |
| TMEM167A | 17.65 | 72 | 8.1 | 8.95 | 0.63080006 |
| WASHC4 | 4.58 | 851 | 98.7 | 7.58 | 0.63087163 |
| PSMB8 | 7.08 | 272 | 29.8 | 5.82 | 0.631293756 |
| BAG3 | 77.11 | 575 | 61.6 | 6.95 | 0.631643481 |
| TCAF2 | 12.85 | 815 | 90.6 | 7.69 | 0.631911528 |
| TOMM20 | 13.8 | 145 | 16.3 | 8.6 | 0.632954538 |
| SLC2A13 | 7.53 | 648 | 70.3 | 5.96 | 0.633111586 |
| SRP9 | 72.48 | 86 | 10.1 | 7.97 | 0.633388605 |
| WDR61 | 25.66 | 305 | 33.6 | 5.47 | 0.634120759 |
| MSH6 | 77.38 | 1360 | 152.7 | 6.9 | 0.636554652 |
| RPP30 | 28.61 | 268 | 29.3 | 8.91 | 0.636559562 |
| NAA35 | 5.99 | 294 | 33.1 | 5.08 | 0.637007096 |
| FAM213A | 7.93 | 218 | 24.4 | 9.01 | 0.637138378 |
| RRP12 | 116.11 | 1197 | 132.6 | 8.68 | 0.63720617 |
| TSR1 | 71.25 | 804 | 91.8 | 7.42 | 0.637467035 |
| NIP7 | 14.45 | 133 | 15.2 | 8.27 | 0.63773564 |
| PPP1CB | 235.76 | 327 | 37.2 | 6.19 | 0.637759591 |
| C17orf62 | 7.17 | 173 | 19.4 | 6.8 | 0.638828076 |
| USP34 | 0 | 3312 | 377.4 | 5.94 | 0.639114087 |
| UBE3C | 28.77 | 1083 | 123.8 | 6.71 | 0.640330142 |
| TJP2 | 21.35 | 1167 | 131.3 | 7.06 | 0.640330846 |
| RRAS2 | 11.35 | 204 | 23.4 | 6.01 | 0.640331834 |
| SLC25A24 | 22.79 | 477 | 53.3 | 6.33 | 0.641642185 |
| TTC19 | 8.86 | 380 | 42.4 | 5.77 | 0.641852252 |
| HPGDS | 9.98 | 199 | 23.3 | 5.85 | 0.6425102 |
| SMARCA5 | 53.16 | 1052 | 121.8 | 8.09 | 0.643688777 |
| HNRNPH2 | 285.38 | 449 | 49.2 | 6.3 | 0.644360101 |
| DOCK7 | 8.64 | 2098 | 238.1 | 6.83 | 0.644716845 |
| NPLOC4 | 41.82 | 608 | 68.1 | 6.38 | 0.644784205 |
| SEL1L | 8.6 | 794 | 88.7 | 5.39 | 0.645547266 |
| ACACA | 42.01 | 2268 | 257.1 | 6.61 | 0.645596625 |
| RPL22L1 | 31.8 | 122 | 14.6 | 9.38 | 0.647420536 |
| WDR46 | 7.22 | 610 | 68 | 9.67 | 0.647468044 |
| C12orf10 | 82.88 | 376 | 42.4 | 6.67 | 0.647494875 |
| FAM198B | 2.3 | 331 | 36.1 | 10.45 | 0.64811795 |
| FITM2 | 3.04 | 262 | 29.8 | 8.65 | 0.65034779 |
| KCNAB2 | 4.04 | 300 | 33.6 | 8.35 | 0.651061898 |
| CERS6 | 6.76 | 384 | 44.9 | 7.65 | 0.651206901 |
| SMARCAD1 | 3.12 | 596 | 68.7 | 7.55 | 0.651850784 |
| TRMT1L | 2.93 | 733 | 81.7 | 7.88 | 0.652243266 |
| METTL7B | 0 | 244 | 27.8 | 8.38 | 0.652808225 |
| ISOC2 | 7.19 | 135 | 14.8 | 9.01 | 0.653048805 |
| ARPC1B | 36.18 | 372 | 40.9 | 8.35 | 0.653216159 |
| CMTM4 | 2.51 | 179 | 19.7 | 4.91 | 0.653773945 |
| ASPSCR1 | 7.69 | 553 | 60.1 | 6.64 | 0.654065266 |
| VTN | 9.43 | 478 | 54.3 | 5.8 | 0.65416299 |
| TACO1 | 10.8 | 297 | 32.5 | 8.13 | 0.654694264 |
| RNF123 | 0 | 1314 | 148.4 | 6.74 | 0.654821332 |
| PEX14 | 0 | 334 | 36.7 | 5.14 | 0.655765626 |
| PPP1CA | 265.97 | 330 | 37.5 | 6.33 | 0.655933926 |
| NIF3L1 | 2.35 | 350 | 39 | 6.4 | 0.656617583 |
| ANKFY1 | 28.18 | 1169 | 128.3 | 6.1 | 0.656868882 |
| MLST8 | 2.2 | 260 | 28.7 | 5.4 | 0.656942618 |
| GPRC5A | 31.17 | 357 | 40.2 | 8.15 | 0.658272988 |
| PRAF2 | 15.18 | 178 | 19.2 | 9.19 | 0.658712177 |
| RAB27B | 12.25 | 218 | 24.6 | 5.52 | 0.658841045 |
| MGST1 | 64.28 | 155 | 17.6 | 9.39 | 0.659158926 |
| FXR2 | 61.74 | 673 | 74.2 | 6.23 | 0.659996118 |
| SLC7A11 | 32.39 | 501 | 55.4 | 9.19 | 0.660111967 |
| NOMO1 | 61.96 | 1222 | 134.2 | 5.81 | 0.660935253 |
| PPIA | 834.46 | 105 | 11.4 | 7.9 | 0.660968531 |
| GOLGA2 | 8.1 | 1002 | 113 | 5.02 | 0.661601819 |
| MT2A | 47.87 | 61 | 6 | 7.83 | 0.661848016 |
| NDUFB4 | 14.65 | 129 | 15.2 | 9.85 | 0.662309751 |
| MRPL28 | 11.55 | 256 | 30.1 | 8.29 | 0.662444916 |
| RFC5 | 26.43 | 319 | 36.1 | 6.95 | 0.662504448 |
| ITGA6 | 100.4 | 1068 | 119 | 7.27 | 0.663606417 |
| RANBP2 | 130.31 | 3224 | 358 | 6.2 | 0.663934195 |
| EMC1 | 93.66 | 992 | 111.6 | 7.66 | 0.664424684 |
| KDM3B | 5.52 | 1761 | 191.5 | 7.18 | 0.664827712 |
| CSNK2A1 | 30.01 | 391 | 45.1 | 7.74 | 0.665170405 |
| SDAD1 | 8.07 | 590 | 68.4 | 9.39 | 0.665563198 |
| ISG20 | 20.43 | 181 | 20.4 | 8.92 | 0.666343048 |
| CLPTM1 | 12.71 | 655 | 75.1 | 6.64 | 0.666532015 |
| CTU1 | 3.65 | 348 | 36.4 | 9.2 | 0.666536022 |
| IMPDH1 | 93.36 | 489 | 52.6 | 6.64 | 0.667174625 |
| TMED9 | 127.25 | 235 | 27.3 | 8.02 | 0.667349141 |
| PRSS3 | 2.4 | 240 | 25.9 | 5.83 | 0.667441556 |
| PSMD13 | 175.11 | 376 | 42.9 | 5.81 | 0.668648333 |
| YIPF5 | 7.91 | 257 | 28 | 4.36 | 0.669910244 |
| POLR1B | 11.93 | 952 | 107.8 | 7.56 | 0.670000483 |
| GPC1 | 31.33 | 558 | 61.6 | 7.3 | 0.671075259 |
| TRMT10C | 46.72 | 403 | 47.3 | 9.36 | 0.671153875 |
| ESYT2 | 79.5 | 893 | 98.8 | 8.68 | 0.671394202 |
| MCM3 | 120.21 | 808 | 90.9 | 5.77 | 0.672735971 |
| NUP210 | 39.66 | 1887 | 205 | 6.81 | 0.674212146 |
| PRKCH | 2.16 | 522 | 59.5 | 7.46 | 0.675225975 |
| HIBCH | 12.72 | 338 | 38 | 8.54 | 0.675261297 |
| ATP5F1 | 63.78 | 256 | 28.9 | 9.36 | 0.675626328 |
| CORO7 | 14.68 | 1048 | 114.1 | 6.33 | 0.676260978 |
| NSA2 | 20.57 | 260 | 30 | 10.27 | 0.676386601 |
| RFLNB | 0 | 214 | 22.9 | 5.44 | 0.677374212 |
| ACADVL | 221.6 | 633 | 68 | 8.56 | 0.677483071 |
| PLCB4 | 2.21 | 1175 | 134.4 | 6.9 | 0.677717035 |
| NEMP1 | 0 | 371 | 42 | 6.16 | 0.678698186 |
| CYB5A | 2.76 | 98 | 11.3 | 5.14 | 0.678923903 |
| GATAD2A | 14.25 | 608 | 65.2 | 9.8 | 0.679086528 |
| ABHD16A | 11.13 | 525 | 59.3 | 7.84 | 0.679354761 |
| LMAN1 | 85.29 | 510 | 57.5 | 6.77 | 0.679829628 |
| BECN1 | 0 | 450 | 51.9 | 4.89 | 0.680185751 |
| CKMT1A | 10.19 | 417 | 47 | 8.34 | 0.680217494 |
| ACO1 | 42.01 | 889 | 98.3 | 6.68 | 0.680509584 |
| NOP58 | 48.35 | 529 | 59.5 | 8.92 | 0.680695395 |
| ALG1 | 24.46 | 464 | 52.5 | 7.23 | 0.681645467 |
| TRIAP1 | 3.35 | 76 | 8.8 | 5.48 | 0.682382245 |
| SLC9A3R2 | 9.09 | 326 | 36.1 | 8.21 | 0.682848343 |
| CAV2 | 8.64 | 149 | 16.8 | 5.66 | 0.683043011 |
| RNF20 | 28.3 | 975 | 113.6 | 5.94 | 0.683186275 |
| CPSF6 | 62.85 | 478 | 52.3 | 6.43 | 0.683196616 |
| DDX1 | 174.95 | 740 | 82.4 | 7.23 | 0.683449569 |
| GNL3 | 80.07 | 537 | 60.5 | 8.79 | 0.684622199 |
| CNOT1 | 35.86 | 2371 | 266.2 | 7.05 | 0.684640639 |
| DNAJB1 | 89.68 | 340 | 38 | 8.63 | 0.684645379 |
| TFAM | 29.46 | 246 | 29.1 | 9.72 | 0.685475567 |
| ZC3HAV1 | 112.77 | 902 | 101.4 | 8.4 | 0.685542103 |
| TPP2 | 18.36 | 1249 | 138.3 | 6.32 | 0.68554953 |
| MINPP1 | 8.45 | 286 | 33.1 | 6.64 | 0.68560382 |
| TMEM97 | 2.06 | 176 | 20.8 | 9.38 | 0.68575404 |
| DNPEP | 29.57 | 475 | 52.4 | 7.42 | 0.686593215 |
| PEX16 | 2.33 | 336 | 38.6 | 9.85 | 0.686749195 |
| SLCO4A1 | 2.82 | 583 | 62.3 | 8.1 | 0.687268722 |
| GLCE | 19.55 | 617 | 70.1 | 8.97 | 0.687598646 |
| LPP | 2.31 | 612 | 65.7 | 7.37 | 0.687825998 |
| ARPC4 | 153.29 | 168 | 19.7 | 8.43 | 0.687978156 |
| UCHL5 | 132.57 | 316 | 36.1 | 5.22 | 0.688038891 |
| SEC13 | 66.86 | 308 | 34 | 5.72 | 0.688120887 |
| WDR82 | 7.95 | 313 | 35.1 | 7.69 | 0.688607748 |
| GLG1 | 9.46 | 1179 | 134.5 | 6.9 | 0.688931225 |
| HMGXB4 | 3.2 | 601 | 65.7 | 9.32 | 0.68914363 |
| CRKL | 17.06 | 303 | 33.8 | 6.74 | 0.690781548 |
| SLC6A6 | 2.94 | 620 | 69.8 | 7.39 | 0.690986532 |
| SMU1 | 99.55 | 513 | 57.5 | 7.18 | 0.691067772 |
| DOCK10 | 16.65 | 2180 | 249.3 | 7.17 | 0.691240623 |
| PPP1R35 | 5.1 | 253 | 27.9 | 8.68 | 0.691389586 |
| FUCA1 | 2.88 | 466 | 53.7 | 6.84 | 0.691402669 |
| PNN | 49.79 | 717 | 81.6 | 7.14 | 0.691432749 |
| STK26 | 42.6 | 339 | 37.7 | 6.09 | 0.69253149 |
| PACSIN3 | 8.72 | 424 | 48.5 | 6.18 | 0.693071005 |
| PSMD9 | 41.96 | 223 | 24.7 | 6.95 | 0.693199985 |
| FKBP9 | 84.55 | 570 | 63 | 5.08 | 0.69364599 |
| POLA1 | 10.99 | 1462 | 165.8 | 5.85 | 0.693929286 |
| DCXR | 45.8 | 244 | 25.9 | 8.1 | 0.694119175 |
| KIF14 | 9.77 | 1648 | 186.4 | 7.91 | 0.694154632 |
| ALKBH2 | 3.52 | 261 | 29.3 | 9.66 | 0.694267953 |
| MRPL40 | 10.69 | 206 | 24.5 | 9.63 | 0.694526654 |
| DKC1 | 28.99 | 514 | 57.6 | 9.42 | 0.694599069 |
| ELOVL1 | 10.85 | 279 | 32.6 | 9.6 | 0.694882572 |
| SEC16A | 10.48 | 2134 | 228.7 | 5.62 | 0.695184132 |
| MTA1 | 31.42 | 430 | 49 | 5.5 | 0.695305776 |
| AHCYL1 | 54.54 | 530 | 58.9 | 6.89 | 0.695665814 |
| SAMHD1 | 3.53 | 591 | 68.1 | 6.9 | 0.695899828 |
| HNRNPD | 439.37 | 287 | 30.7 | 8.41 | 0.695925635 |
| CASP7 | 40.93 | 303 | 34.3 | 6.07 | 0.696454972 |
| XPNPEP3 | 112.54 | 428 | 48.1 | 5.62 | 0.696891038 |
| PTPN2 | 2.32 | 353 | 41 | 6.4 | 0.697016015 |
| SLC30A1 | 20.08 | 507 | 55.3 | 6.48 | 0.697699693 |
| SPAG5 | 19.93 | 1193 | 134.3 | 5 | 0.697921433 |
| SH3GLB2 | 11.62 | 395 | 43.9 | 5.99 | 0.697942387 |
| PREP | 49.15 | 710 | 80.6 | 5.86 | 0.698525252 |
| DYNC1H1 | 1644.14 | 4646 | 532.1 | 6.4 | 0.698525933 |
| NUP50 | 10.33 | 440 | 46.8 | 8.27 | 0.698735837 |
| CDC73 | 35.34 | 531 | 60.5 | 9.61 | 0.699147216 |
| STT3A | 77.89 | 705 | 80.5 | 8.07 | 0.699963613 |
| HYOU1 | 342.89 | 999 | 111.3 | 5.22 | 0.70060729 |
| SLC16A6 | 9.75 | 523 | 57.4 | 7.81 | 0.700704717 |
| TIMELESS | 12.4 | 1207 | 138.4 | 5.38 | 0.701938164 |
| DHX36 | 9.33 | 979 | 111.4 | 7.56 | 0.702390595 |
| DUSP3 | 26.46 | 144 | 16.2 | 9.26 | 0.703418917 |
| CD59 | 34.04 | 128 | 14.2 | 6.48 | 0.703665556 |
| PTPA | 17.52 | 294 | 33.4 | 6.29 | 0.704074994 |
| MRPL12 | 88.53 | 198 | 21.3 | 8.87 | 0.704244145 |
| RPL3 | 363.58 | 403 | 46.1 | 10.18 | 0.704301759 |
| MFF | 90.64 | 291 | 33 | 7.44 | 0.704601138 |
| APOOL | 2.63 | 268 | 29.1 | 9.52 | 0.704977436 |
| NR0B1 | 10.65 | 400 | 43.6 | 8.25 | 0.705108868 |
| CBWD1 | 0 | 113 | 12.2 | 4.26 | 0.705609093 |
| MTMR1 | 5.39 | 568 | 63.3 | 7.46 | 0.706185499 |
| ITGA5 | 4.09 | 1049 | 114.5 | 5.77 | 0.708299362 |
| TFPI2 | 2.58 | 224 | 25.8 | 8.65 | 0.708310435 |
| SEC31A | 71.02 | 1067 | 117.6 | 7.39 | 0.708359081 |
| TRMU | 4.59 | 376 | 42.9 | 7.71 | 0.708446534 |
| SH2D4A | 5.86 | 409 | 46.9 | 7.53 | 0.708586084 |
| CEP350 | 0 | 3117 | 350.7 | 6.33 | 0.708591072 |
| CCNH | 8.23 | 323 | 37.6 | 7.15 | 0.709022171 |
| DPH2 | 2.13 | 261 | 27.5 | 4.7 | 0.709385935 |
| ATAD3A | 154.75 | 586 | 66.2 | 9.25 | 0.709965248 |
| TAPBP | 5.74 | 412 | 43.9 | 6.99 | 0.710283153 |
| NIPSNAP1 | 5.18 | 284 | 33.3 | 9.31 | 0.710694322 |
| GMPS | 156.86 | 693 | 76.7 | 6.87 | 0.710914127 |
| QTRT2 | 6.35 | 292 | 33.4 | 5.88 | 0.710977512 |
| TMCO1 | 39.85 | 169 | 18.8 | 9.61 | 0.711657401 |
| SNX2 | 30.3 | 519 | 58.4 | 5.12 | 0.712624405 |
| UBE3A | 21.56 | 852 | 97.9 | 5.22 | 0.712896499 |
| RALB | 11.41 | 206 | 23.4 | 6.62 | 0.712917616 |
| TBCD | 60.38 | 1192 | 132.5 | 6.19 | 0.713196931 |
| ARPC5L | 36.05 | 153 | 16.9 | 6.6 | 0.713436248 |
| VKORC1L1 | 22.8 | 176 | 19.8 | 9.13 | 0.713936376 |
| RBM26 | 16.49 | 980 | 110.6 | 9.07 | 0.714287501 |
| CCS | 6.8 | 274 | 29 | 5.58 | 0.714530655 |
| PHB | 413.11 | 272 | 29.8 | 5.76 | 0.714793953 |
| PLAA | 14.3 | 795 | 87.1 | 6.37 | 0.714881799 |
| PDE1C | 7 | 634 | 72.2 | 6.64 | 0.715179508 |
| TOM1 | 3.21 | 447 | 48.6 | 4.67 | 0.715803284 |
| STAMBP | 14.51 | 424 | 48 | 6.29 | 0.715839083 |
| SF3B2 | 109.71 | 895 | 100.2 | 5.67 | 0.716826903 |
| SLFN5 | 18.61 | 891 | 101 | 8.22 | 0.716915086 |
| GRIN1 | 0 | 901 | 101.1 | 8.35 | 0.717014878 |
| RPS15A | 314.57 | 130 | 14.8 | 10.13 | 0.71792511 |
| SNRPC | 26.91 | 159 | 17.4 | 9.67 | 0.718299646 |
| SLC35F6 | 6.69 | 371 | 40.2 | 6.93 | 0.718742784 |
| PPIE | 19.22 | 301 | 33.4 | 5.6 | 0.718960127 |
| PAK1IP1 | 6.15 | 392 | 43.9 | 8.91 | 0.719164271 |
| NDUFS2 | 61.46 | 457 | 51.8 | 8.15 | 0.719185467 |
| MEN1 | 3.84 | 575 | 63.7 | 6.62 | 0.719404262 |
| FARSA | 97.56 | 508 | 57.5 | 7.8 | 0.720056558 |
| CPVL | 0 | 476 | 54.1 | 5.62 | 0.72051888 |
| M6PR | 36.64 | 277 | 31 | 5.83 | 0.721233677 |
| PANK3 | 2.79 | 370 | 41.1 | 6.55 | 0.721511089 |
| TUBGCP5 | 0 | 1024 | 118.2 | 5.9 | 0.721818573 |
| PLXNB2 | 14.37 | 1838 | 205 | 6.24 | 0.722390069 |
| AURKA | 6.57 | 403 | 45.8 | 9.39 | 0.722886172 |
| NDUFAF7 | 2.44 | 343 | 38 | 8.6 | 0.723412389 |
| RBM25 | 47.9 | 843 | 100.1 | 6.32 | 0.723607589 |
| QDPR | 5.26 | 213 | 22.4 | 9.11 | 0.72514754 |
| MIA3 | 8.53 | 1848 | 206.8 | 4.82 | 0.725727379 |
| HSD17B11 | 25.86 | 300 | 32.9 | 9.07 | 0.726239175 |
| PHF10 | 4.34 | 451 | 51.3 | 6.77 | 0.726659432 |
| CYC1 | 61.52 | 325 | 35.4 | 9 | 0.726780362 |
| TDP2 | 7.39 | 362 | 40.9 | 5.1 | 0.727014656 |
| UBXN1 | 10.16 | 297 | 33.3 | 5.25 | 0.72716433 |
| NT5C2 | 11.01 | 532 | 61.4 | 5.74 | 0.727168773 |
| CCDC6 | 27.47 | 474 | 53.3 | 7.34 | 0.727255914 |
| NCBP2 | 0 | 103 | 11.9 | 8.92 | 0.727836049 |
| CFAP20 | 29.44 | 193 | 22.8 | 9.76 | 0.727963387 |
| LLPH | 19.46 | 129 | 15.2 | 10.37 | 0.72836311 |
| FNDC3B | 7.75 | 629 | 69.5 | 7.25 | 0.728499161 |
| RBM12B | 15.94 | 1001 | 118 | 6.81 | 0.728918612 |
| EXOC4 | 25.32 | 974 | 110.4 | 6.49 | 0.728938401 |
| AP1G1 | 17.98 | 822 | 91.3 | 6.8 | 0.729448745 |
| SNRPB2 | 86.54 | 225 | 25.5 | 9.72 | 0.729794306 |
| MOB4 | 21.5 | 193 | 22.3 | 6.14 | 0.729821073 |
| PWP2 | 31.96 | 919 | 102.4 | 6.15 | 0.730236235 |
| ZZEF1 | 3.44 | 1085 | 119.9 | 5.67 | 0.730384189 |
| PM20D2 | 5.56 | 436 | 47.7 | 5.85 | 0.73143056 |
| TBCE | 55.3 | 527 | 59.3 | 6.76 | 0.731569118 |
| PPM1F | 7.59 | 350 | 38.5 | 6.76 | 0.732541656 |
| TDP1 | 6.28 | 608 | 68.4 | 7.65 | 0.732985183 |
| SEC24D | 15.52 | 1032 | 112.9 | 7.25 | 0.733590093 |
| KCT2 | 0 | 265 | 29.2 | 5.08 | 0.733776116 |
| RABL3 | 2.97 | 236 | 26.4 | 7.11 | 0.734546796 |
| SERPINB1 | 112.01 | 379 | 42.7 | 6.28 | 0.734553907 |
| ATP7B | 2.27 | 1258 | 133.5 | 6.43 | 0.734884428 |
| RTRAF | 136.89 | 244 | 28.1 | 6.65 | 0.734974178 |
| GPN3 | 3.54 | 284 | 32.7 | 4.5 | 0.735768189 |
| MRPL22 | 14.74 | 206 | 23.6 | 9.94 | 0.735956096 |
| RBM10 | 47.52 | 929 | 103.4 | 5.97 | 0.736335251 |
| GAPVD1 | 35.96 | 1433 | 159.7 | 5.17 | 0.73660175 |
| RAB3GAP2 | 41.94 | 1393 | 155.9 | 5.62 | 0.738290842 |
| F11R | 1.91 | 299 | 32.6 | 7.9 | 0.738555411 |
| ANKRD40 | 5.02 | 368 | 41.1 | 4.97 | 0.738972338 |
| NCAPH2 | 2.43 | 583 | 65.7 | 4.68 | 0.739251865 |
| PITHD1 | 9.52 | 210 | 24.1 | 5.74 | 0.739414163 |
| FECH | 6.16 | 423 | 47.8 | 8.73 | 0.739430218 |
| EIF3H | 116.79 | 352 | 39.9 | 6.54 | 0.739613624 |
| MDN1 | 10.3 | 5596 | 632.4 | 5.68 | 0.74051034 |
| CWC27 | 5.3 | 390 | 44 | 5.74 | 0.74090668 |
| SSU72 | 12.58 | 194 | 22.6 | 5.33 | 0.74173472 |
| LYPLAL1 | 14.45 | 237 | 26.3 | 7.84 | 0.742982653 |
| EDC3 | 3.95 | 508 | 56 | 7.11 | 0.743296321 |
| RELA | 5.93 | 537 | 58.8 | 5.91 | 0.743364925 |
| PLK1 | 3.31 | 603 | 68.2 | 8.91 | 0.743604954 |
| RIF1 | 10.02 | 2446 | 271.5 | 5.41 | 0.743749658 |
| LSM6 | 8.69 | 80 | 9.1 | 9.58 | 0.744048725 |
| RABL6 | 28.16 | 729 | 79.5 | 5.22 | 0.74441564 |
| KPNA2 | 326.59 | 529 | 57.8 | 5.4 | 0.744584075 |
| GTF3C4 | 22.34 | 822 | 91.9 | 6.65 | 0.74543116 |
| TM9SF3 | 9.18 | 589 | 67.8 | 7.21 | 0.7463419 |
| PSMB9 | 19.51 | 209 | 22.3 | 5.01 | 0.747293029 |
| GSTZ1 | 6.05 | 161 | 17.9 | 5.96 | 0.747330205 |
| NUDT5 | 99.26 | 219 | 24.3 | 4.94 | 0.747336119 |
| QRSL1 | 2.01 | 303 | 32.4 | 6.68 | 0.748066184 |
| PIGK | 7.06 | 395 | 45.2 | 6.16 | 0.748720415 |
| PTPN14 | 6.55 | 1187 | 135.2 | 8.31 | 0.749118354 |
| SLC25A1 | 35.82 | 311 | 34 | 9.89 | 0.751895464 |
| PPP2CA | 191.69 | 309 | 35.6 | 5.54 | 0.752136693 |
| GPATCH4 | 42.39 | 446 | 50.4 | 9.63 | 0.752378075 |
| NDUFS7 | 20.92 | 206 | 22.2 | 9.88 | 0.752698805 |
| ATP6V1F | 3.29 | 119 | 13.4 | 5.52 | 0.753220246 |
| ABT1 | 6.56 | 272 | 31.1 | 9.88 | 0.753267008 |
| TBC1D13 | 6.24 | 400 | 46.5 | 5.24 | 0.753304324 |
| CHCHD3 | 48.47 | 227 | 26.1 | 8.28 | 0.753529745 |
| CMBL | 52.63 | 245 | 28 | 7.18 | 0.754930293 |
| TMEM214 | 33.43 | 644 | 72 | 9.26 | 0.755248983 |
| WDR55 | 15.41 | 383 | 42 | 4.92 | 0.755307696 |
| CCDC47 | 36.32 | 483 | 55.8 | 4.87 | 0.756051923 |
| LSM14A | 8.39 | 463 | 50.5 | 9.52 | 0.758056266 |
| SLC27A4 | 22.41 | 643 | 72 | 8.47 | 0.758108668 |
| SART1 | 41.15 | 800 | 90.2 | 6.13 | 0.758254103 |
| RTFDC1 | 11.91 | 306 | 33.9 | 8.59 | 0.758473823 |
| FMNL2 | 4.31 | 1086 | 123.2 | 7.4 | 0.759901537 |
| NDUFA5 | 26.73 | 116 | 13.5 | 5.99 | 0.760173204 |
| CTNNAL1 | 3.95 | 650 | 72.6 | 7.06 | 0.760681518 |
| ALG13 | 4.82 | 165 | 18.2 | 6.52 | 0.76113962 |
| HSPA13 | 5.49 | 471 | 51.9 | 5.76 | 0.761148017 |
| ATAD2 | 0 | 1390 | 158.5 | 6.32 | 0.761249556 |
| CHD4 | 70.2 | 1912 | 217.9 | 5.86 | 0.761425148 |
| EHD1 | 54.13 | 534 | 60.6 | 6.83 | 0.761840798 |
| PPP1R8 | 3.02 | 351 | 38.5 | 7.37 | 0.761908365 |
| IDH3B | 49.83 | 385 | 42.2 | 8.46 | 0.762069681 |
| FKBP15 | 15.61 | 1209 | 132.4 | 5.17 | 0.762091068 |
| EIF4E | 45.05 | 217 | 25.1 | 6.15 | 0.762656649 |
| NUP214 | 34.03 | 2079 | 212.4 | 7.37 | 0.763197648 |
| MTOR | 3.12 | 2549 | 288.7 | 7.17 | 0.763247454 |
| TRAM1 | 6.2 | 343 | 39.7 | 9.63 | 0.763719789 |
| NSD2 | 2.59 | 484 | 53.5 | 9.19 | 0.763736101 |
| DNAJC9 | 26.02 | 260 | 29.9 | 5.73 | 0.764012156 |
| ERLIN1 | 91.83 | 346 | 38.9 | 7.87 | 0.764294702 |
| OAT | 165.5 | 439 | 48.5 | 7.03 | 0.76443398 |
| UTP15 | 18.01 | 499 | 56.3 | 9.13 | 0.764455998 |
| MYADM | 6.76 | 322 | 35.3 | 8.15 | 0.764674822 |
| POFUT1 | 20.7 | 388 | 43.9 | 8.53 | 0.764717951 |
| PDCL3 | 14.24 | 239 | 27.6 | 4.84 | 0.764858315 |
| PEX19 | 2.87 | 261 | 29.2 | 4.55 | 0.764970541 |
| DNASE1L1 | 3.73 | 302 | 33.9 | 5.74 | 0.765469859 |
| UBA3 | 24.83 | 463 | 51.8 | 5.45 | 0.765556615 |
| TRMT1 | 22.37 | 630 | 69.3 | 7.23 | 0.766568291 |
| WDR5 | 17.04 | 334 | 36.6 | 8.27 | 0.766860982 |
| SORD | 234.08 | 357 | 38.3 | 7.97 | 0.766947859 |
| GRIA1 | 11.13 | 826 | 92.1 | 7.74 | 0.766980747 |
| SCRN2 | 29.54 | 378 | 41.3 | 6.19 | 0.76703131 |
| CEP170 | 5.54 | 1460 | 161.3 | 7.01 | 0.767296874 |
| COPB1 | 225.83 | 953 | 107.1 | 6.05 | 0.767411708 |
| SLC2A3 | 19.87 | 496 | 53.9 | 7.2 | 0.767527841 |
| NUP160 | 21.57 | 1436 | 162 | 5.5 | 0.767693785 |
| EXOSC10 | 51.53 | 860 | 98 | 8.4 | 0.767906778 |
| ZNF551 | 4.88 | 597 | 69.3 | 8.57 | 0.768369032 |
| PLRG1 | 2.55 | 505 | 56.3 | 9.17 | 0.768559853 |
| PCID2 | 5.55 | 376 | 43.3 | 8.63 | 0.768586977 |
| RPP25L | 2.47 | 163 | 17.6 | 10.3 | 0.76897867 |
| ATRX | 5.4 | 2288 | 259.7 | 6.79 | 0.769049826 |
| LONP1 | 65.53 | 763 | 85.6 | 6.04 | 0.769669037 |
| UBE2E1 | 2.98 | 160 | 18 | 8.78 | 0.770253379 |
| TP53I3 | 4.27 | 332 | 35.5 | 7.17 | 0.770271873 |
| TM9SF2 | 37.98 | 663 | 75.7 | 7.44 | 0.770294293 |
| GRPEL1 | 28.86 | 217 | 24.3 | 8.12 | 0.770477701 |
| DNAJC13 | 13.39 | 2243 | 254.3 | 6.74 | 0.771979324 |
| USP10 | 34.31 | 798 | 87.1 | 5.31 | 0.772415973 |
| U2SURP | 16.38 | 1028 | 118.2 | 8.47 | 0.773789636 |
| PLCB1 | 18.94 | 1173 | 133.6 | 6.42 | 0.77392832 |
| RBM3 | 19.33 | 157 | 17.2 | 8.91 | 0.77411188 |
| NT5E | 7.77 | 524 | 57.9 | 6.98 | 0.774613002 |
| PUM1 | 9.63 | 1162 | 124.3 | 6.79 | 0.774978653 |
| HTRA2 | 12.07 | 361 | 38.5 | 10.71 | 0.775576251 |
| KDELC1 | 3.69 | 502 | 58 | 7.71 | 0.776694985 |
| CUL4A | 54.94 | 759 | 87.6 | 8.13 | 0.776712757 |
| FHOD1 | 49.38 | 1164 | 126.5 | 6.39 | 0.777128515 |
| RTCB | 108.18 | 505 | 55.2 | 7.23 | 0.777441645 |
| CHD3 | 45.21 | 2000 | 226.4 | 7.3 | 0.778191708 |
| RNASEH2C | 10.61 | 163 | 17.7 | 4.94 | 0.778597033 |
| PRPF3 | 15.45 | 683 | 77.5 | 9.5 | 0.778603915 |
| TRIP11 | 4.78 | 1979 | 227.4 | 5.26 | 0.778604647 |
| RNF185 | 2.05 | 192 | 20.4 | 6.52 | 0.779002576 |
| ATP1B3 | 67 | 279 | 31.5 | 8.35 | 0.779067825 |
| TYMS | 32.64 | 313 | 35.7 | 7.01 | 0.779348157 |
| LTF | 19 | 666 | 73.1 | 7.85 | 0.779578199 |
| VPS18 | 2.59 | 973 | 110.1 | 6.07 | 0.779815331 |
| LAMB1 | 42.53 | 1786 | 197.9 | 4.94 | 0.780089505 |
| TUBGCP2 | 19.87 | 902 | 102.5 | 6.84 | 0.78116834 |
| TWF1 | 47.63 | 350 | 40.3 | 6.96 | 0.781256246 |
| TMEM245 | 9.64 | 503 | 55.4 | 9.33 | 0.781819299 |
| ATP13A1 | 17.72 | 1086 | 121 | 7.75 | 0.781900785 |
| NUP54 | 6.39 | 291 | 33.2 | 6.37 | 0.78229427 |
| NKRF | 21.53 | 690 | 77.6 | 8.79 | 0.782554295 |
| HINT2 | 32.79 | 163 | 17.2 | 9.16 | 0.783294174 |
| ALDOC | 517.59 | 364 | 39.4 | 6.87 | 0.783325308 |
| HEBP2 | 24.26 | 205 | 22.9 | 4.63 | 0.783428518 |
| SEC23A | 139.07 | 765 | 86.1 | 7.08 | 0.785060535 |
| GPKOW | 22.51 | 476 | 52.2 | 6.15 | 0.785343847 |
| GNPNAT1 | 26.49 | 184 | 20.7 | 7.99 | 0.785368074 |
| MPZL1 | 5.93 | 145 | 15.9 | 9.03 | 0.785694672 |
| ADD1 | 22.44 | 631 | 69.9 | 6.46 | 0.785816834 |
| SLC25A19 | 0 | 263 | 29.2 | 9.77 | 0.78593307 |
| BTF3L4 | 14.08 | 158 | 17.3 | 6.35 | 0.785996227 |
| SRSF3 | 229.56 | 124 | 14.2 | 10.08 | 0.786379465 |
| MPV17 | 2.1 | 176 | 19.7 | 9.47 | 0.786876938 |
| SLC9A6 | 4.33 | 649 | 72.2 | 5.74 | 0.786882242 |
| TMEM109 | 46.44 | 243 | 26.2 | 10.48 | 0.787029017 |
| CHMP5 | 19.1 | 219 | 24.6 | 4.83 | 0.787339948 |
| CCAR2 | 46.68 | 923 | 102.8 | 5.22 | 0.788509586 |
| CDC42BPB | 8.47 | 1711 | 194.2 | 6.37 | 0.788767417 |
| AK2 | 189.5 | 232 | 25.6 | 7.88 | 0.788838399 |
| NNT | 36.33 | 1086 | 113.8 | 8.09 | 0.789315701 |
| NUDT19 | 13.69 | 375 | 42.2 | 7.64 | 0.7893233 |
| LIN7C | 9.35 | 197 | 21.8 | 8.43 | 0.789640201 |
| KDM1A | 23.27 | 852 | 92.8 | 6.52 | 0.789704143 |
| GLMN | 2.04 | 417 | 48.1 | 5.45 | 0.790860443 |
| HM13 | 54.09 | 335 | 36.8 | 6.68 | 0.791105505 |
| RRP1 | 14.25 | 461 | 52.8 | 9.33 | 0.791638581 |
| ATG16L1 | 2.35 | 444 | 49.5 | 7.9 | 0.791728271 |
| PPID | 95.45 | 370 | 40.7 | 7.21 | 0.792125055 |
| JMJD6 | 31.63 | 372 | 43.1 | 8.88 | 0.792203102 |
| DDX39A | 333.86 | 427 | 49.1 | 5.68 | 0.792344942 |
| ACAT1 | 170.42 | 427 | 45.2 | 8.85 | 0.792456601 |
| NELFA | 7.36 | 528 | 57.2 | 9.03 | 0.793065371 |
| T | 22.44 | 377 | 41.1 | 6.58 | 0.793174433 |
| POLA2 | 2.18 | 361 | 40.6 | 4.89 | 0.793404402 |
| ARPC3 | 46.76 | 178 | 20.5 | 8.59 | 0.794122119 |
| NHLRC2 | 15.64 | 726 | 79.4 | 5.55 | 0.794244787 |
| MRPL37 | 26.85 | 423 | 48.1 | 8.59 | 0.795113155 |
| C18orf25 | 0 | 342 | 37 | 5.12 | 0.795538436 |
| AKT1S1 | 9.39 | 256 | 27.4 | 4.75 | 0.795677225 |
| PAFAH1B3 | 44.71 | 231 | 25.7 | 6.84 | 0.795893337 |
| CD99 | 53.74 | 169 | 17.1 | 4.56 | 0.795968217 |
| NECAP1 | 3.04 | 102 | 11.4 | 6.7 | 0.795977022 |
| THUMPD1 | 22.77 | 353 | 39.3 | 7.88 | 0.796055264 |
| LARP4 | 41.88 | 723 | 80.4 | 6.61 | 0.796057594 |
| HMOX1 | 18.93 | 288 | 32.8 | 8.25 | 0.796771516 |
| PAK2 | 47.77 | 524 | 58 | 5.96 | 0.797005761 |
| DDX42 | 58.56 | 819 | 90 | 7.75 | 0.797165335 |
| DNAJC11 | 24.2 | 507 | 57.2 | 7.53 | 0.797463142 |
| RBM22 | 13.51 | 371 | 41.1 | 8.19 | 0.797600474 |
| U2AF2 | 125.4 | 471 | 53.1 | 9.09 | 0.797812977 |
| PTGFRN | 7.34 | 879 | 98.5 | 6.61 | 0.797835635 |
| MESD | 40.83 | 234 | 26.1 | 7.78 | 0.798571068 |
| NUDT9 | 3.38 | 300 | 33.8 | 6.76 | 0.799257461 |
| ATL2 | 20.77 | 565 | 64.5 | 5.68 | 0.799626514 |
| RABGEF1 | 2.17 | 491 | 56.9 | 7.02 | 0.799689245 |
| TXNL1 | 130.19 | 289 | 32.2 | 4.96 | 0.800083552 |
| SRRM1 | 13.29 | 902 | 102.1 | 11.81 | 0.800584712 |
| PFDN6 | 65.19 | 129 | 14.6 | 8.88 | 0.801117947 |
| AK1 | 75.5 | 194 | 21.6 | 8.63 | 0.801210236 |
| XPO1 | 370.21 | 1071 | 123.3 | 6.06 | 0.801671156 |
| DHCR24 | 15.35 | 475 | 54.7 | 6.92 | 0.80173613 |
| BCAS2 | 3.81 | 225 | 26.1 | 5.66 | 0.801872771 |
| MGME1 | 0 | 344 | 39.4 | 7.68 | 0.801979598 |
| MAGOH | 48.97 | 146 | 17.2 | 6.11 | 0.80251204 |
| ADRM1 | 38.51 | 407 | 42.1 | 5.07 | 0.802631166 |
| ALAS1 | 3.04 | 640 | 70.5 | 8.46 | 0.802640238 |
| RAB8B | 238.25 | 207 | 23.6 | 9.07 | 0.802742887 |
| DPYSL3 | 86.32 | 684 | 73.9 | 6.35 | 0.80359885 |
| MT-CO3 | 0 | 261 | 29.9 | 7.31 | 0.803991253 |
| RBM14 | 159.58 | 669 | 69.4 | 9.67 | 0.804813965 |
| ABRAXAS2 | 2.91 | 415 | 46.9 | 6.21 | 0.805230775 |
| TMED5 | 8.65 | 229 | 26 | 4.84 | 0.805565144 |
| CTPS2 | 26.05 | 586 | 65.6 | 6.9 | 0.805852866 |
| APOL2 | 2.07 | 337 | 37.1 | 6.74 | 0.806220065 |
| ELAVL1 | 153.43 | 326 | 36.1 | 9.17 | 0.806254465 |
| MIEN1 | 8.11 | 115 | 12.4 | 4.37 | 0.806283593 |
| SLC25A12 | 69.98 | 678 | 74.7 | 8.38 | 0.806487449 |
| PROCR | 91.07 | 238 | 26.7 | 7.18 | 0.806706193 |
| CSNK2A2 | 34.34 | 350 | 41.2 | 8.56 | 0.806845065 |
| MTMR9 | 2.68 | 464 | 53.8 | 6.34 | 0.807231388 |
| CSTF2 | 78.27 | 560 | 59.2 | 6.76 | 0.807433056 |
| SEPT10 | 44.21 | 431 | 50 | 7.02 | 0.807847998 |
| PABPC4L | 3.05 | 370 | 41.8 | 9.5 | 0.807919438 |
| PCBP2 | 564.04 | 318 | 33.5 | 8.24 | 0.808217489 |
| MT-ND3 | 3.55 | 115 | 13.2 | 4.44 | 0.808874245 |
| ARGLU1 | 0 | 198 | 23.8 | 9.2 | 0.809552592 |
| NUP205 | 59.61 | 2012 | 227.8 | 6.19 | 0.809666332 |
| RBM4 | 44.79 | 364 | 40.3 | 7.08 | 0.809731059 |
| HSPB6 | 45.02 | 160 | 17.1 | 6.4 | 0.809946698 |
| MAP2K2 | 57.14 | 400 | 44.4 | 6.55 | 0.810715637 |
| IVD | 20.33 | 423 | 46.3 | 8.19 | 0.811231013 |
| RRP9 | 20.61 | 475 | 51.8 | 7.85 | 0.811295298 |
| UCHL3 | 3.54 | 230 | 26.2 | 4.92 | 0.811328734 |
| AKR1C2 | 737.53 | 323 | 36.7 | 7.49 | 0.811549912 |
| GPRIN3 | 0 | 776 | 82.4 | 7.56 | 0.811591094 |
| DENR | 37.89 | 198 | 22.1 | 5.3 | 0.811874659 |
| TPM1 | 422.23 | 284 | 32.8 | 4.77 | 0.81243855 |
| ASNS | 52.45 | 478 | 54.8 | 6.49 | 0.81281376 |
| CTSL | 199.18 | 333 | 37.5 | 5.45 | 0.812825076 |
| MFAP1 | 5.21 | 439 | 51.9 | 4.98 | 0.812936459 |
| PLEC | 1791.35 | 4547 | 515.9 | 5.8 | 0.812977568 |
| CCAR1 | 24.45 | 1135 | 131 | 5.76 | 0.813151427 |
| TIMM9 | 15.16 | 89 | 10.4 | 7.21 | 0.813176352 |
| PTCD3 | 12.27 | 689 | 78.5 | 6.42 | 0.813651078 |
| SEC24A | 37.68 | 1093 | 119.7 | 7.66 | 0.813653148 |
| WASHC3 | 6.9 | 194 | 21.2 | 4.46 | 0.813785567 |
| TGFBI | 30.23 | 683 | 74.6 | 7.71 | 0.81429633 |
| COMT | 38.2 | 221 | 24.4 | 5.33 | 0.814372286 |
| RIOK2 | 2.92 | 474 | 54.5 | 5.41 | 0.814635586 |
| CRELD2 | 5.93 | 325 | 35.3 | 4.65 | 0.814647923 |
| GOSR2 | 4.96 | 195 | 22.7 | 6.95 | 0.815092245 |
| TJP1 | 60.02 | 1668 | 186.9 | 6.79 | 0.815228644 |
| NHP2 | 36.74 | 153 | 17.2 | 8.22 | 0.81541464 |
| PPFIBP1 | 9.3 | 980 | 110.2 | 5.41 | 0.815728346 |
| SYNGR2 | 13.33 | 224 | 24.8 | 4.94 | 0.816474419 |
| DIDO1 | 10.89 | 2240 | 243.7 | 7.88 | 0.817031725 |
| XPOT | 92.27 | 962 | 109.9 | 5.39 | 0.817118172 |
| AIMP2 | 87.84 | 320 | 35.3 | 8.22 | 0.81719981 |
| ARL6IP1 | 19.48 | 203 | 23.3 | 9.32 | 0.81769979 |
| SRSF10 | 65.84 | 165 | 20.1 | 10.05 | 0.817782911 |
| FKBP8 | 14.28 | 412 | 44.5 | 4.84 | 0.818219929 |
| DHX29 | 10.14 | 1369 | 155.1 | 8.09 | 0.818406774 |
| WASF2 | 13.01 | 498 | 54.3 | 5.53 | 0.819410255 |
| PAXX | 17.37 | 204 | 21.6 | 5.48 | 0.81989516 |
| HS1BP3 | 19.28 | 392 | 42.8 | 5.01 | 0.819943256 |
| PGM3 | 30.84 | 542 | 59.8 | 6.25 | 0.820609249 |
| MAGED2 | 36.83 | 606 | 64.9 | 9.32 | 0.820823381 |
| MTX1 | 19.96 | 317 | 35.8 | 6.29 | 0.822136053 |
| BOP1 | 63.01 | 746 | 83.6 | 6.19 | 0.822331669 |
| CHML | 12.27 | 656 | 74 | 4.93 | 0.823289514 |
| ATP5F1E | 15.27 | 51 | 5.8 | 9.92 | 0.823365701 |
| MEPCE | 16.26 | 689 | 74.3 | 9.57 | 0.823742161 |
| LARS | 267.86 | 1176 | 134.4 | 7.3 | 0.823891094 |
| BAG4 | 10.06 | 421 | 45.4 | 5.05 | 0.824064051 |
| YES1 | 50.59 | 543 | 60.8 | 6.74 | 0.824311419 |
| PFAS | 299.94 | 1338 | 144.6 | 5.76 | 0.825113883 |
| SF3B5 | 10.95 | 86 | 10.1 | 6.35 | 0.825663917 |
| JUP | 227.89 | 745 | 81.7 | 6.14 | 0.825834735 |
| NUP62 | 14.32 | 522 | 53.2 | 5.31 | 0.825928758 |
| RBMS2 | 5.63 | 407 | 43.9 | 9.07 | 0.825945023 |
| MRPL20 | 7.16 | 149 | 17.4 | 10.86 | 0.827147441 |
| BPIFA3 | 0 | 218 | 24.3 | 7.31 | 0.827429923 |
| NUP88 | 17.6 | 741 | 83.5 | 5.69 | 0.828332143 |
| PLCB3 | 44.22 | 1167 | 131.1 | 5.88 | 0.828409621 |
| RALY | 87.49 | 306 | 32.4 | 9.17 | 0.829370463 |
| NSMCE1 | 5.54 | 266 | 30.8 | 7.47 | 0.829472901 |
| CTBP2 | 23.28 | 445 | 48.9 | 6.95 | 0.829691834 |
| INTS2 | 2.15 | 1204 | 134.2 | 6.05 | 0.830042506 |
| SEC61B | 12.47 | 96 | 10 | 11.56 | 0.830341665 |
| MRPS2 | 5.82 | 296 | 33.2 | 9.26 | 0.83046626 |
| GALE | 13.37 | 348 | 38.3 | 6.73 | 0.830817511 |
| PFDN5 | 32.31 | 154 | 17.3 | 6.33 | 0.830890551 |
| EGLN1 | 15.36 | 325 | 36.5 | 8.37 | 0.831322164 |
| UFL1 | 34.86 | 794 | 89.5 | 6.79 | 0.832002469 |
| SFXN3 | 36.18 | 325 | 36 | 9.09 | 0.832014845 |
| UBE2Z | 5.5 | 246 | 28.1 | 6.1 | 0.832263548 |
| GMPPA | 9.11 | 420 | 46.3 | 7.21 | 0.832293475 |
| PIN1 | 18.82 | 163 | 18.2 | 8.82 | 0.832457029 |
| HIP1R | 13.98 | 1068 | 119.3 | 6.67 | 0.832542452 |
| TMEM205 | 28.24 | 189 | 21.2 | 8.62 | 0.833160882 |
| NUBP2 | 30.31 | 271 | 28.8 | 5.83 | 0.833608645 |
| CKAP5 | 120.63 | 1972 | 218.4 | 8.06 | 0.833872918 |
| RPL36 | 193.18 | 105 | 12.2 | 11.59 | 0.834288657 |
| BOLA3 | 4.35 | 107 | 12.1 | 9.64 | 0.835264663 |
| EXOSC8 | 5.24 | 276 | 30 | 5.3 | 0.835266815 |
| TMOD2 | 7.34 | 351 | 39.6 | 5.27 | 0.835552579 |
| MAGEB2 | 92.81 | 319 | 35.3 | 8.76 | 0.835597549 |
| COPA | 443.02 | 1224 | 138.3 | 7.66 | 0.835637486 |
| VPS35 | 150.15 | 796 | 91.6 | 5.49 | 0.835669543 |
| TPRKB | 14.99 | 175 | 19.6 | 6.79 | 0.836083771 |
| CDK6 | 31.57 | 326 | 36.9 | 6.46 | 0.836987809 |
| TIAL1 | 57.78 | 375 | 41.6 | 7.74 | 0.836998459 |
| PMVK | 53.8 | 192 | 22 | 5.73 | 0.837689827 |
| AGFG1 | 60.16 | 522 | 54.1 | 8.92 | 0.837957158 |
| POLR1E | 0 | 419 | 47.2 | 8.94 | 0.838321451 |
| EIF2AK2 | 59.29 | 551 | 62.1 | 8.4 | 0.838957673 |
| TRAPPC1 | 6.57 | 145 | 16.8 | 9.16 | 0.839294949 |
| URB2 | 3.88 | 1524 | 170.4 | 7.31 | 0.839318944 |
| CASP8 | 30.21 | 464 | 53.7 | 5.2 | 0.839424032 |
| COG1 | 3.1 | 980 | 108.9 | 7.31 | 0.839733764 |
| ZNF787 | 3.34 | 383 | 40.5 | 7.84 | 0.839767266 |
| NELFCD | 7.9 | 581 | 65.4 | 5.1 | 0.841604603 |
| SPR | 49.64 | 261 | 28 | 8.05 | 0.841637276 |
| TEX10 | 24.99 | 913 | 103.8 | 9.36 | 0.84187567 |
| DNTTIP2 | 24.97 | 756 | 84.4 | 6.16 | 0.841918791 |
| LRRC40 | 8.94 | 602 | 68.2 | 6.43 | 0.842306985 |
| TCEA1 | 43.15 | 301 | 33.9 | 8.38 | 0.842489275 |
| PHF14 | 4.22 | 663 | 75.5 | 7.71 | 0.842540228 |
| ATPAF1 | 9.24 | 328 | 36.4 | 7.96 | 0.842702988 |
| CHD1 | 14.89 | 1709 | 196.5 | 7.14 | 0.842855861 |
| AP1G2 | 7.18 | 785 | 87.1 | 6.55 | 0.843076477 |
| DPP3 | 44.58 | 707 | 79.3 | 5.03 | 0.84410442 |
| HEXIM1 | 54.3 | 359 | 40.6 | 4.89 | 0.844150671 |
| TNPO3 | 41.51 | 923 | 104.1 | 5.57 | 0.84478825 |
| DCPS | 45.69 | 337 | 38.6 | 6.38 | 0.844946903 |
| CCDC94 | 2.67 | 323 | 37.1 | 5.92 | 0.845502582 |
| TRA2B | 153.65 | 188 | 21.9 | 10.15 | 0.845528481 |
| SURF4 | 12.6 | 159 | 18 | 6.05 | 0.845698285 |
| HTATIP2 | 52.65 | 242 | 27 | 8.38 | 0.845742853 |
| ORC6 | 2.77 | 252 | 28.1 | 8.66 | 0.845808944 |
| ADK | 46.03 | 345 | 38.7 | 6.68 | 0.845912922 |
| EIF1AX | 115.38 | 144 | 16.5 | 5.24 | 0.846111662 |
| RFK | 11.76 | 155 | 17.6 | 8.13 | 0.84620154 |
| ME1 | 146.67 | 572 | 64.1 | 6.13 | 0.846561301 |
| EEFSEC | 3.99 | 596 | 65.3 | 8.35 | 0.846893479 |
| EIF4EBP1 | 2.01 | 118 | 12.6 | 5.48 | 0.847584841 |
| XPO4 | 4.18 | 1151 | 130.1 | 5.05 | 0.847990583 |
| NEDD1 | 17.96 | 571 | 62.3 | 8.28 | 0.84825421 |
| SYNM | 57.32 | 1565 | 172.7 | 5.16 | 0.849359937 |
| PRPSAP1 | 23.63 | 356 | 39.4 | 7.2 | 0.849522762 |
| UFC1 | 28.79 | 167 | 19.4 | 7.4 | 0.849686534 |
| GALNS | 2.26 | 522 | 58 | 6.74 | 0.850549656 |
| ALDH1A2 | 17.46 | 422 | 46.1 | 6.29 | 0.85077059 |
| RAB39B | 114.19 | 213 | 24.6 | 7.83 | 0.852710162 |
| RTN3 | 11.96 | 236 | 25.6 | 8.51 | 0.85310014 |
| CHURC1 | 0 | 139 | 16.1 | 5.54 | 0.853384233 |
| AIMP1 | 56.64 | 312 | 34.3 | 8.43 | 0.85353323 |
| HSPA14 | 27.99 | 509 | 54.8 | 5.59 | 0.853654728 |
| EIF2B5 | 6.62 | 721 | 80.3 | 5.08 | 0.853829303 |
| CARNMT1 | 10.88 | 409 | 47.2 | 6.32 | 0.854054007 |
| NME3 | 6.85 | 169 | 19 | 7.84 | 0.85409116 |
| NOL11 | 12.49 | 719 | 81.1 | 6.07 | 0.85414984 |
| DLG1 | 8.09 | 853 | 95.1 | 5.97 | 0.854315649 |
| CHERP | 10.98 | 916 | 103.6 | 9.04 | 0.854595325 |
| CORO1B | 21.46 | 489 | 54.2 | 5.88 | 0.855045851 |
| SLC44A1 | 30.33 | 657 | 73.3 | 8.6 | 0.85527276 |
| PACSIN2 | 11.65 | 445 | 51.3 | 5.39 | 0.856014612 |
| SEC63 | 15.45 | 760 | 87.9 | 5.31 | 0.856069883 |
| SEPT8 | 94.61 | 369 | 43.5 | 6.34 | 0.856472836 |
| JPT2 | 74.6 | 174 | 18.4 | 9.26 | 0.857189255 |
| GHITM | 16.37 | 345 | 37.2 | 9.94 | 0.85727045 |
| DHX16 | 17.07 | 1041 | 119.2 | 6.8 | 0.857937555 |
| BABAM1 | 3.24 | 171 | 18.6 | 4.44 | 0.858498769 |
| MRPL55 | 6.03 | 128 | 15.1 | 11.15 | 0.859327287 |
| NEDD8 | 38.95 | 81 | 9.1 | 8.43 | 0.859339924 |
| POLE3 | 20.98 | 147 | 16.8 | 4.74 | 0.859501729 |
| PFKP | 667.85 | 784 | 85.5 | 7.55 | 0.8595433 |
| POLR2M | 9.33 | 86 | 8.8 | 8.25 | 0.859877169 |
| PGM2 | 56.45 | 612 | 68.2 | 6.73 | 0.859930242 |
| GTF2H4 | 10.34 | 462 | 52.2 | 9.04 | 0.860215649 |
| CPT1A | 60.46 | 756 | 86.2 | 8.44 | 0.861106396 |
| TMEM106B | 11.14 | 274 | 31.1 | 6.99 | 0.861171295 |
| KLF16 | 2.89 | 252 | 25.4 | 9.88 | 0.861310147 |
| NAA25 | 10.47 | 859 | 99.2 | 6.27 | 0.861659156 |
| CAD | 351 | 2225 | 242.8 | 6.46 | 0.861831011 |
| AK4 | 6.38 | 223 | 25.3 | 8.4 | 0.861923032 |
| NSMCE3 | 2.29 | 304 | 34.3 | 9.28 | 0.861968305 |
| DPM1 | 55.38 | 260 | 29.6 | 9.57 | 0.862629875 |
| CDKN2AIPNL | 2.9 | 116 | 13.2 | 5 | 0.863822989 |
| UPP1 | 10.27 | 310 | 33.9 | 7.88 | 0.86396637 |
| ASMTL | 16.57 | 605 | 67.2 | 6.14 | 0.864010208 |
| LEMD2 | 2.26 | 503 | 56.9 | 9 | 0.864923764 |
| ZNF638 | 9.14 | 1978 | 220.5 | 6.38 | 0.865204775 |
| XPNPEP1 | 56.27 | 642 | 72.1 | 6.02 | 0.865280602 |
| TMUB1 | 0 | 246 | 26.2 | 5.72 | 0.865522188 |
| GGH | 5.67 | 318 | 35.9 | 7.11 | 0.865791502 |
| SF3B4 | 27.7 | 424 | 44.4 | 8.56 | 0.866004661 |
| ADAM17 | 7.98 | 694 | 78.5 | 5.88 | 0.86602367 |
| TIMM23 | 11.32 | 209 | 21.9 | 8.6 | 0.866185504 |
| USP7 | 54.84 | 1086 | 126.2 | 5.71 | 0.866743788 |
| GPX1 | 4.7 | 203 | 22.1 | 6.55 | 0.866876103 |
| EBNA1BP2 | 72.69 | 306 | 34.8 | 10.1 | 0.866935102 |
| MGEA5 | 41.62 | 863 | 96.9 | 5.02 | 0.867032694 |
| POLR2A | 16.08 | 1970 | 217 | 7.37 | 0.867034623 |
| TTC1 | 8.77 | 292 | 33.5 | 4.84 | 0.867303762 |
| HSD17B10 | 418.1 | 261 | 26.9 | 7.78 | 0.867674677 |
| APP | 4.45 | 733 | 82.9 | 4.78 | 0.867902977 |
| TMEM263 | 2.49 | 116 | 11.7 | 9.32 | 0.867903088 |
| MRTO4 | 81.41 | 239 | 27.5 | 8.29 | 0.868507694 |
| PMPCA | 46.46 | 525 | 58.2 | 6.92 | 0.868797015 |
| SH3PXD2B | 12.21 | 911 | 101.5 | 8.69 | 0.868985729 |
| NXN | 28.21 | 435 | 48.4 | 4.97 | 0.869556197 |
| KCTD3 | 2.46 | 169 | 18.5 | 9.16 | 0.869882904 |
| CAVIN1 | 183.79 | 390 | 43.4 | 5.6 | 0.870089949 |
| CBFB | 41.15 | 187 | 22 | 5.58 | 0.87016761 |
| STRN | 6.89 | 731 | 80.7 | 5.17 | 0.870525076 |
| PPIL3 | 19.2 | 161 | 18.1 | 6.79 | 0.871007815 |
| PICALM | 13.89 | 610 | 66.4 | 8.81 | 0.872350878 |
| TMED3 | 7.54 | 217 | 24.8 | 5.6 | 0.872374382 |
| HNRNPD | 434.09 | 306 | 32.8 | 8.16 | 0.872375713 |
| SNF8 | 4.58 | 257 | 28.7 | 6.44 | 0.872588514 |
| HSP90AA4P | 410.05 | 418 | 47.7 | 5.19 | 0.872652888 |
| SNRPGP15 | 76.65 | 76 | 8.5 | 8.84 | 0.872820385 |
| STYX | 4.4 | 223 | 25.5 | 6.29 | 0.874243874 |
| TBRG4 | 36.18 | 631 | 70.7 | 7.42 | 0.874399203 |
| AHNAK2 | 45.36 | 5695 | 605.3 | 5.36 | 0.874408981 |
| FN1 | 82.59 | 2176 | 239.5 | 5.88 | 0.874473201 |
| AP1M1 | 26.64 | 423 | 48.6 | 7.3 | 0.874825904 |
| LYPLA1 | 16.52 | 214 | 22.9 | 6.51 | 0.874940662 |
| TGM3 | 0 | 693 | 76.6 | 5.86 | 0.874993315 |
| PIGU | 5.1 | 415 | 47.6 | 8.18 | 0.875020965 |
| UTP6 | 20.87 | 597 | 70.1 | 7.28 | 0.875449583 |
| SLC5A6 | 6.12 | 635 | 68.6 | 8.27 | 0.875614638 |
| KPNA4 | 21.51 | 521 | 57.9 | 4.96 | 0.875646183 |
| AAK1 | 4.76 | 863 | 93.5 | 8.53 | 0.876198434 |
| ENAH | 34.82 | 533 | 60.3 | 7.55 | 0.876311965 |
| PTGES | 3.21 | 152 | 17.1 | 9.5 | 0.876551975 |
| SERPINH1 | 78.92 | 418 | 46.4 | 8.69 | 0.876553527 |
| TBL1XR1 | 20.03 | 514 | 55.6 | 5.55 | 0.877016413 |
| KPNA3 | 31.63 | 521 | 57.8 | 4.94 | 0.877237086 |
| SNX12 | 34.35 | 162 | 18.9 | 8.44 | 0.877318977 |
| RAB8A | 257.32 | 207 | 23.7 | 9.07 | 0.877796371 |
| STX8 | 5.39 | 236 | 26.9 | 4.98 | 0.877841616 |
| HGS | 9.41 | 690 | 76.3 | 5.86 | 0.878013845 |
| SHMT1 | 51.8 | 403 | 44.5 | 7.5 | 0.878044287 |
| RGP1 | 2.55 | 391 | 42.4 | 5.96 | 0.878082268 |
| SURF6 | 7.96 | 361 | 41.4 | 10.64 | 0.878439731 |
| OVCA2 | 4.19 | 227 | 24.4 | 6.89 | 0.878788743 |
| KIAA2013 | 8.6 | 634 | 69.1 | 8.19 | 0.879473879 |
| PPP1CC | 265.43 | 323 | 37 | 6.54 | 0.879484722 |
| TBK1 | 2.38 | 729 | 83.6 | 6.79 | 0.879548359 |
| ZFC3H1 | 0 | 346 | 37.8 | 9.77 | 0.879583252 |
| UBE2V1 | 113.69 | 147 | 16.5 | 7.93 | 0.879643257 |
| STAU1 | 34.03 | 496 | 54.9 | 9.51 | 0.879989219 |
| ABCD3 | 22.95 | 549 | 62.7 | 9.23 | 0.880124887 |
| TRIM16 | 34.42 | 564 | 63.9 | 5.49 | 0.88015216 |
| SULT1A1 | 4.35 | 217 | 25.4 | 6.21 | 0.880730935 |
| TRA2A | 65.65 | 181 | 20.7 | 10.24 | 0.882045249 |
| DLGAP4 | 3.45 | 453 | 49.4 | 6.01 | 0.882356176 |
| ASS1 | 73.72 | 412 | 46.5 | 8.02 | 0.882824108 |
| TFG | 75.87 | 396 | 43 | 5.17 | 0.883053432 |
| OARD1 | 2.54 | 152 | 17 | 8.31 | 0.883109772 |
| SHCBP1 | 17.43 | 672 | 75.6 | 4.75 | 0.883204399 |
| LXN | 19.24 | 222 | 25.7 | 5.78 | 0.883270606 |
| CSTF3 | 24.24 | 717 | 82.9 | 8.12 | 0.883330451 |
| DRG1 | 100.32 | 367 | 40.5 | 8.9 | 0.883403554 |
| NDUFV1 | 39.76 | 455 | 49.8 | 8.21 | 0.883781279 |
| ARL6IP4 | 3.31 | 421 | 44.9 | 10.93 | 0.883962921 |
| EMC3 | 18.68 | 261 | 29.9 | 6.81 | 0.884039312 |
| IPO7 | 157.37 | 1038 | 119.4 | 4.82 | 0.884271697 |
| DSCAML1 | 0 | 1842 | 200.9 | 8.35 | 0.88457306 |
| GTF2E1 | 14.47 | 439 | 49.4 | 4.82 | 0.885686736 |
| DENND3 | 0 | 1146 | 129.7 | 6.87 | 0.885965028 |
| DPY19L1 | 20.93 | 675 | 77.3 | 8.95 | 0.886080164 |
| DHFR | 5.23 | 135 | 15.7 | 6.34 | 0.886437105 |
| ECPAS | 116.31 | 1845 | 204.2 | 7.12 | 0.886437466 |
| ALDH16A1 | 19.98 | 751 | 79.9 | 7.58 | 0.886654464 |
| NOP16 | 30.84 | 178 | 21.2 | 9.94 | 0.886665482 |
| TMED1 | 2.98 | 227 | 25.2 | 4.48 | 0.887039166 |
| HAX1 | 5.89 | 124 | 14.2 | 4.87 | 0.887113828 |
| EIF3L | 76.83 | 564 | 66.7 | 6.34 | 0.887347988 |
| KIF23 | 26.92 | 856 | 98 | 8.47 | 0.887428982 |
| TIMP1 | 6.37 | 207 | 23.2 | 8.1 | 0.887453754 |
| SAP18 | 20.69 | 153 | 17.5 | 9.35 | 0.887559829 |
| ZSWIM8 | 0 | 1841 | 197.5 | 6.83 | 0.887572066 |
| PKD1 | 3.35 | 4292 | 461.1 | 6.77 | 0.887734449 |
| MYH14 | 223.08 | 1995 | 227.7 | 5.6 | 0.888155873 |
| PCK2 | 5.4 | 441 | 47.5 | 8.13 | 0.888239873 |
| MPHOSPH10 | 15.68 | 681 | 78.8 | 4.86 | 0.888296829 |
| TMPO | 363.06 | 694 | 75.4 | 7.66 | 0.888353725 |
| SCARB2 | 23.1 | 478 | 54.3 | 5.14 | 0.888897954 |
| DDX46 | 96.97 | 1031 | 117.3 | 9.29 | 0.889305555 |
| SETD3 | 18.7 | 594 | 67.2 | 5.96 | 0.889368234 |
| LPCAT1 | 23.49 | 534 | 59.1 | 6.02 | 0.889431178 |
| NUDCD3 | 14.4 | 361 | 40.8 | 5.25 | 0.889647368 |
| RETSAT | 6.79 | 610 | 66.8 | 8.28 | 0.889786701 |
| PUF60 | 92.07 | 499 | 54 | 5.33 | 0.889884158 |
| MRPL32 | 2.42 | 188 | 21.4 | 9.73 | 0.889912015 |
| SUZ12 | 2.36 | 739 | 83 | 8.81 | 0.890288625 |
| TBL3 | 66.52 | 808 | 89 | 6.9 | 0.890338848 |
| TLN1 | 457.35 | 2541 | 269.6 | 6.07 | 0.890823319 |
| TMX1 | 63.47 | 280 | 31.8 | 4.98 | 0.891011915 |
| DAP3 | 44.34 | 357 | 41 | 8.98 | 0.891161039 |
| HEATR1 | 111.19 | 2144 | 242.2 | 6.54 | 0.89136379 |
| TPD52 | 63.89 | 184 | 19.9 | 4.96 | 0.891809337 |
| COMMD9 | 2.25 | 156 | 17.3 | 6.93 | 0.891897313 |
| USP47 | 4.35 | 157 | 18.1 | 5.19 | 0.891919051 |
| SLC1A4 | 18.12 | 532 | 55.7 | 6.25 | 0.892089012 |
| HCCS | 10.99 | 268 | 30.6 | 6.68 | 0.893255419 |
| COPS3 | 32.61 | 403 | 45.7 | 6.65 | 0.893735885 |
| BAIAP2 | 6.9 | 512 | 56.6 | 8.97 | 0.893750001 |
| ANLN | 29.07 | 1087 | 119.9 | 8.16 | 0.894799296 |
| OXCT1 | 50.88 | 520 | 56.1 | 7.46 | 0.894824313 |
| DDX56 | 18.75 | 507 | 57.2 | 9.22 | 0.895572034 |
| IPO9 | 75.33 | 1041 | 115.9 | 4.81 | 0.895932653 |
| PSMD10 | 46.35 | 226 | 24.4 | 6.1 | 0.896322474 |
| RNASEH2B | 0 | 257 | 29 | 8.82 | 0.896323062 |
| MRPL11 | 22.62 | 192 | 20.7 | 9.91 | 0.897112917 |
| SAFB2 | 42.66 | 953 | 107.4 | 6.16 | 0.89722455 |
| MRPS17 | 9.29 | 130 | 14.5 | 9.85 | 0.897604083 |
| CCDC93 | 5.47 | 631 | 73.2 | 8.15 | 0.897779833 |
| KIF22 | 9.49 | 597 | 66.3 | 8.91 | 0.897831533 |
| SUCLG2 | 95.97 | 432 | 46.5 | 6.39 | 0.897881516 |
| DDX18 | 142.08 | 670 | 75.4 | 9.5 | 0.898059347 |
| SNRNP200 | 388.12 | 2136 | 244.4 | 6.06 | 0.89840339 |
| PHF8 | 2.91 | 948 | 105.8 | 7.55 | 0.89848107 |
| HEATR3 | 25.98 | 594 | 65.8 | 5.11 | 0.900420194 |
| CTCF | 5.23 | 399 | 46 | 8.43 | 0.900710132 |
| CAPZB | 115.13 | 272 | 30.6 | 6 | 0.900740631 |
| BRD4 | 4.24 | 1362 | 152.1 | 9.19 | 0.901017683 |
| HCLS1 | 7.28 | 486 | 54 | 4.81 | 0.901134209 |
| NQO2 | 39.1 | 231 | 25.9 | 6.29 | 0.901191361 |
| SRP19 | 6.97 | 144 | 16.1 | 9.85 | 0.90119665 |
| GMPR2 | 15.25 | 320 | 34.6 | 7.68 | 0.901733824 |
| MT-CO1 | 3.8 | 513 | 57 | 6.7 | 0.901776674 |
| NUP155 | 83.73 | 1332 | 148.9 | 6.34 | 0.901800601 |
| RUFY1 | 13.58 | 412 | 44.5 | 6.11 | 0.902102337 |
| AIDA | 9.02 | 306 | 35 | 6.55 | 0.902395202 |
| SHPK | 6.76 | 478 | 51.5 | 6.83 | 0.902805132 |
| PTRH2 | 15.56 | 179 | 19.2 | 8.73 | 0.903495822 |
| PPM1A | 5.75 | 324 | 35.9 | 5.78 | 0.903838137 |
| MCC | 2.69 | 829 | 93 | 5.52 | 0.904335248 |
| EEA1 | 35.2 | 1411 | 162.4 | 5.68 | 0.904371193 |
| GPN1 | 3.8 | 295 | 33.4 | 4.68 | 0.904510318 |
| SAP30 | 4.75 | 220 | 23.3 | 9.17 | 0.904840748 |
| RBM47 | 9 | 524 | 56.9 | 6.33 | 0.904903624 |
| ARL1 | 52.32 | 164 | 18.6 | 5.71 | 0.905028419 |
| UBA2 | 64.64 | 640 | 71.2 | 5.29 | 0.905274044 |
| COMMD3 | 7.73 | 195 | 22.1 | 5.99 | 0.905274495 |
| VPS51 | 2.69 | 658 | 72.6 | 7.14 | 0.905394211 |
| IK | 5.43 | 557 | 65.6 | 6.64 | 0.906404519 |
| MRPS27 | 36.77 | 414 | 47.6 | 6.18 | 0.906438877 |
| POLR1C | 32.92 | 342 | 38.6 | 6.01 | 0.907614358 |
| AAR2 | 10.66 | 384 | 43.4 | 5.96 | 0.907622176 |
| PSMB1 | 197.63 | 241 | 26.5 | 8.13 | 0.907688082 |
| NDUFV3 | 17.63 | 473 | 51 | 9.17 | 0.907776047 |
| METTL3 | 13.78 | 580 | 64.4 | 6.42 | 0.907931317 |
| DCTPP1 | 56.96 | 170 | 18.7 | 5.03 | 0.908369595 |
| ZFR | 57.82 | 1074 | 116.9 | 9.04 | 0.908767822 |
| IGF2R | 212.29 | 2491 | 274.2 | 5.94 | 0.908971421 |
| MAT2A | 165 | 395 | 43.6 | 6.48 | 0.910321852 |
| UBE2T | 28.07 | 197 | 22.5 | 7.99 | 0.910369214 |
| MOV10 | 6.57 | 900 | 101.5 | 9.2 | 0.910450898 |
| CENPH | 9.84 | 247 | 28.5 | 5.29 | 0.910495422 |
| PTK2B | 6.9 | 967 | 111.1 | 5.91 | 0.910519002 |
| THOC5 | 2.29 | 683 | 78.5 | 6.87 | 0.910986781 |
| NOL10 | 16.27 | 638 | 74.5 | 8.38 | 0.911348758 |
| CCDC43 | 14.2 | 224 | 25.2 | 4.92 | 0.91146848 |
| NECAP2 | 2.62 | 172 | 19.4 | 6.57 | 0.911912353 |
| POLR3D | 2.45 | 398 | 44.4 | 6.98 | 0.912104558 |
| GGT1 | 23.5 | 569 | 61.4 | 7.12 | 0.912110613 |
| TOMM70 | 57.69 | 608 | 67.4 | 7.12 | 0.912262526 |
| AUP1 | 36.93 | 410 | 45.8 | 8.65 | 0.912895326 |
| VPS36 | 8.35 | 328 | 36.9 | 7.49 | 0.913475936 |
| RAB20 | 5.9 | 234 | 26.3 | 6.55 | 0.913601206 |
| PKLR | 137.88 | 543 | 58.5 | 7.05 | 0.914156084 |
| KIF21A | 27.76 | 1621 | 180.6 | 6.33 | 0.914489935 |
| PLS1 | 77.5 | 629 | 70.2 | 5.41 | 0.914726833 |
| LARP1 | 81.86 | 1096 | 123.4 | 8.82 | 0.914827412 |
| MAP1S | 7.14 | 1033 | 109.7 | 7.65 | 0.915126228 |
| TUBB2A | 3361.07 | 445 | 49.9 | 4.89 | 0.915166431 |
| NFU1 | 12.18 | 230 | 25.9 | 4.69 | 0.915586977 |
| EXOSC9 | 27.93 | 439 | 48.9 | 5.29 | 0.915958044 |
| FERMT2 | 17.17 | 680 | 77.8 | 6.7 | 0.916914203 |
| CELF1 | 38.19 | 468 | 50.1 | 8.85 | 0.91710877 |
| RPS27 | 74.53 | 84 | 9.5 | 9.45 | 0.917278068 |
| UBE2C | 5.37 | 140 | 15.8 | 5.83 | 0.917469265 |
| MRPS22 | 24.11 | 360 | 41.3 | 7.9 | 0.917963803 |
| SRSF11 | 45.3 | 483 | 53.4 | 10.52 | 0.918323946 |
| C12orf57 | 2.49 | 126 | 13.2 | 5.14 | 0.918518 |
| POTEJ | 793.22 | 1038 | 117.3 | 5.97 | 0.918656442 |
| HIST1H1D | 259.22 | 221 | 22.3 | 11.02 | 0.919408946 |
| REXO4 | 3.52 | 422 | 46.6 | 9.77 | 0.919948188 |
| HPCAL1 | 139.92 | 193 | 22.3 | 5.35 | 0.920241307 |
| PSAT1 | 290.87 | 370 | 40.4 | 7.66 | 0.920303226 |
| BMS1 | 16.65 | 1282 | 145.7 | 6.44 | 0.920362494 |
| MRGBP | 9.91 | 204 | 22.4 | 5.83 | 0.921215166 |
| CNIH4 | 18.83 | 139 | 16.1 | 6.65 | 0.921245222 |
| LIG3 | 20.54 | 1009 | 112.8 | 9.01 | 0.921253101 |
| SMG8 | 0 | 588 | 65 | 7.66 | 0.921413478 |
| LRRC41 | 2.36 | 729 | 79.2 | 8.81 | 0.921431412 |
| PCCB | 4.44 | 539 | 58.2 | 7.64 | 0.921544709 |
| CDK11A | 2.65 | 397 | 45.2 | 9.25 | 0.921560976 |
| RRS1 | 52.5 | 365 | 41.2 | 10.7 | 0.921664945 |
| MCAM | 3.28 | 527 | 57.6 | 5.52 | 0.922278484 |
| FKBP2 | 16.53 | 142 | 15.6 | 9.13 | 0.922463006 |
| NCAPH | 26.87 | 741 | 82.5 | 5.06 | 0.923385548 |
| HDDC2 | 17 | 170 | 19.5 | 5.6 | 0.923759759 |
| GNAI1 | 70.72 | 354 | 40.3 | 5.97 | 0.923799733 |
| MTREX | 109.03 | 1042 | 117.7 | 6.52 | 0.924573334 |
| RAE1 | 29.7 | 368 | 40.9 | 7.83 | 0.925024069 |
| STT3B | 49.7 | 826 | 93.6 | 8.91 | 0.925040807 |
| PCYOX1 | 46.37 | 505 | 56.6 | 6.18 | 0.925646719 |
| TAP1 | 4.25 | 808 | 87.2 | 8.02 | 0.925999435 |
| ACSL3 | 130.84 | 720 | 80.4 | 8.38 | 0.926228017 |
| RPRD1A | 7.73 | 276 | 31.6 | 7.08 | 0.927162303 |
| PPIL1 | 24.26 | 166 | 18.2 | 7.99 | 0.927400758 |
| NUP58 | 2.91 | 485 | 50.2 | 8.72 | 0.927623785 |
| BAX | 21.82 | 164 | 18.1 | 8.16 | 0.927672232 |
| CLPP | 11.27 | 277 | 30.2 | 8.09 | 0.927785061 |
| PSMB6 | 73.89 | 239 | 25.3 | 4.92 | 0.928068249 |
| RALGAPB | 7.18 | 1273 | 141.8 | 6.81 | 0.928280212 |
| RPRD2 | 2.72 | 1424 | 152.5 | 7.33 | 0.928514386 |
| GTF2I | 5.17 | 957 | 107.9 | 7.94 | 0.928799157 |
| FMR1 | 37.05 | 586 | 66.2 | 8.47 | 0.928886091 |
| EXO5 | 0 | 373 | 41.8 | 5.43 | 0.929138691 |
| SUMO4 | 79.21 | 95 | 10.7 | 7.18 | 0.929168016 |
| CBR1 | 161.21 | 277 | 30.4 | 8.32 | 0.929309187 |
| HADH | 61.8 | 314 | 34.3 | 8.85 | 0.929888 |
| P3H4 | 5.08 | 437 | 50.3 | 4.77 | 0.930065378 |
| PRMT7 | 6.45 | 567 | 63.8 | 5.81 | 0.930304157 |
| FNTA | 2.22 | 312 | 36.5 | 5.2 | 0.930366168 |
| FAM96A | 10.16 | 160 | 18.3 | 4.88 | 0.930797561 |
| SNX27 | 7.23 | 448 | 52.3 | 6.37 | 0.931864695 |
| PPP3CA | 20.52 | 454 | 51.2 | 5.74 | 0.931940417 |
| SAR1B | 89.11 | 198 | 22.4 | 6.11 | 0.932000805 |
| KDELC2 | 6.11 | 507 | 58.5 | 8.24 | 0.932023881 |
| CMC1 | 3.28 | 106 | 12.5 | 8.63 | 0.932801182 |
| TSC22D1 | 4.64 | 144 | 15.7 | 5.2 | 0.93283342 |
| LCMT1 | 3.07 | 279 | 32.2 | 6.32 | 0.932933646 |
| PFN2 | 144.86 | 140 | 15 | 6.99 | 0.933016221 |
| DHRS4 | 4.21 | 158 | 16.9 | 7.83 | 0.933091535 |
| MSH2 | 70.98 | 934 | 104.7 | 5.77 | 0.933340201 |
| UTP14A | 42.93 | 719 | 82 | 7.85 | 0.934401191 |
| ATG3 | 25.2 | 311 | 35.4 | 4.79 | 0.935071615 |
| CDH11 | 9.59 | 796 | 87.9 | 4.91 | 0.935307956 |
| SEC23IP | 64 | 1000 | 111 | 5.54 | 0.935313605 |
| RAPH1 | 16.03 | 619 | 70 | 6.07 | 0.936112487 |
| SLC4A7 | 18.08 | 1000 | 112.4 | 7.11 | 0.936154853 |
| DNAJA3 | 82.26 | 453 | 49.6 | 9.39 | 0.93618114 |
| PDCD10 | 21.64 | 212 | 24.7 | 8.19 | 0.936231184 |
| PPAN | 11.55 | 460 | 51.9 | 10.01 | 0.936271557 |
| LAMA5 | 2.53 | 3695 | 399.5 | 7.02 | 0.936899047 |
| YARS | 149.36 | 528 | 59.1 | 7.05 | 0.936928106 |
| DCAF7 | 9.67 | 342 | 38.9 | 5.52 | 0.937353247 |
| SLC25A4 | 676.75 | 298 | 33 | 9.76 | 0.93756128 |
| RAD50 | 8.85 | 1312 | 153.8 | 6.89 | 0.937904955 |
| SNX1 | 30.57 | 457 | 51.8 | 5.64 | 0.937989817 |
| NDUFS3 | 85.73 | 264 | 30.2 | 7.5 | 0.938048937 |
| KPNA1 | 45.38 | 538 | 60.2 | 5.01 | 0.938130719 |
| HK2 | 86.97 | 917 | 102.3 | 6.05 | 0.93814759 |
| PPHLN1 | 5.55 | 319 | 36.9 | 7.02 | 0.938249599 |
| CEP290 | 6.78 | 1539 | 180 | 6.84 | 0.938255739 |
| PBDC1 | 42.94 | 233 | 26 | 4.79 | 0.938311032 |
| GALK1 | 23.77 | 392 | 42.2 | 6.46 | 0.938404778 |
| HSPA6 | 1177.85 | 643 | 71 | 6.14 | 0.938909351 |
| PHGDH | 215.04 | 533 | 56.6 | 6.71 | 0.938968505 |
| VANGL1 | 0 | 522 | 59.7 | 8.81 | 0.939017158 |
| NCS1 | 8.67 | 190 | 21.9 | 4.83 | 0.939616667 |
| CUTA | 55.62 | 156 | 16.8 | 5.21 | 0.939982694 |
| EPS8 | 5.92 | 822 | 91.8 | 7.5 | 0.940379951 |
| HCFC1 | 29.37 | 1966 | 201.7 | 7.46 | 0.940421592 |
| PLEC | 1808.32 | 4684 | 531.5 | 5.96 | 0.940512453 |
| PON2 | 7.04 | 342 | 38 | 5.72 | 0.941490787 |
| CENPF | 11.43 | 3210 | 367.5 | 5.07 | 0.941502636 |
| MYCN | 2.32 | 464 | 49.5 | 5.66 | 0.941629843 |
| DYNC1LI1 | 10.93 | 523 | 56.5 | 6.42 | 0.941903998 |
| TSPAN4 | 0 | 238 | 26.1 | 6.47 | 0.941959301 |
| GOLIM4 | 13.18 | 696 | 81.8 | 4.77 | 0.942598182 |
| LAP3 | 220.59 | 488 | 52.7 | 6.74 | 0.942692753 |
| GMFB | 28.97 | 142 | 16.7 | 5.29 | 0.943041122 |
| NOC3L | 19.41 | 800 | 92.5 | 9.17 | 0.943219382 |
| SEPT9 | 324.73 | 586 | 65.4 | 8.97 | 0.943521662 |
| METAP1 | 29.53 | 386 | 43.2 | 7.17 | 0.943659641 |
| U2AF1 | 67.29 | 240 | 27.9 | 8.81 | 0.944568535 |
| PIN4 | 18.26 | 131 | 13.8 | 9.77 | 0.944582898 |
| GNB4 | 73.81 | 340 | 37.5 | 6 | 0.944620603 |
| RPP40 | 8.76 | 340 | 39.3 | 7.01 | 0.944737567 |
| KNTC1 | 5.89 | 2209 | 250.6 | 5.97 | 0.945221652 |
| PPP1R18 | 6.83 | 613 | 67.9 | 5.4 | 0.945648136 |
| NELFE | 10.27 | 380 | 43.2 | 9.33 | 0.946164822 |
| CTTN | 133.13 | 550 | 61.5 | 5.4 | 0.946215549 |
| NCKAP1 | 14.14 | 1128 | 128.7 | 6.62 | 0.946349919 |
| HECTD1 | 27.66 | 2610 | 289.2 | 5.35 | 0.946411622 |
| YKT6 | 17.85 | 164 | 18.6 | 7.33 | 0.946428261 |
| SMAD2 | 22.8 | 437 | 48.9 | 7.09 | 0.947286596 |
| WDR70 | 5.05 | 654 | 73.2 | 6.33 | 0.947887188 |
| MTPAP | 2.94 | 582 | 66.1 | 9.04 | 0.948076209 |
| PRPSAP2 | 41.13 | 320 | 35.5 | 8.78 | 0.948134676 |
| TMX3 | 23.31 | 454 | 51.8 | 4.91 | 0.948311083 |
| VPS13C | 10.24 | 3585 | 402.8 | 6.38 | 0.949254432 |
| FAM50A | 14.04 | 339 | 40.2 | 6.83 | 0.949494626 |
| GOLGA3 | 25.92 | 1390 | 155.6 | 5.4 | 0.949642652 |
| RAB21 | 32.9 | 225 | 24.3 | 7.94 | 0.94976182 |
| ABCD1 | 2.17 | 745 | 82.9 | 8.95 | 0.950723147 |
| ACOT1 | 62.38 | 421 | 46.2 | 7.34 | 0.951377074 |
| HUWE1 | 29.69 | 4358 | 479.9 | 5.24 | 0.95197145 |
| UBA5 | 4.63 | 348 | 38.5 | 4.78 | 0.952625391 |
| MTCH1 | 25.5 | 372 | 39.9 | 9.48 | 0.952799485 |
| RAB5A | 91.73 | 201 | 22.2 | 8.82 | 0.952906211 |
| PAPOLA | 13.22 | 745 | 82.8 | 7.37 | 0.952957025 |
| KIF3A | 7.18 | 699 | 80 | 6.54 | 0.952968578 |
| AP1S3 | 1.84 | 104 | 12.6 | 9.58 | 0.953197455 |
| WDHD1 | 6.57 | 1006 | 112.6 | 5.73 | 0.95330343 |
| RAB1B | 345.53 | 201 | 22.2 | 5.73 | 0.953434058 |
| SLC7A1 | 0 | 629 | 67.6 | 5.43 | 0.953548467 |
| CLIP1 | 72.57 | 1392 | 156.7 | 5.36 | 0.953823114 |
| FAM207A | 12.29 | 215 | 23.9 | 10.86 | 0.953955888 |
| REPIN1 | 0 | 567 | 63.5 | 9.98 | 0.953991272 |
| SIN3A | 5.26 | 1273 | 145.1 | 7.25 | 0.954433675 |
| VKORC1 | 10.29 | 92 | 9.9 | 8.4 | 0.954543661 |
| CENPV | 18.67 | 272 | 29.7 | 9.73 | 0.954874428 |
| DNAJB4 | 19.86 | 337 | 37.8 | 8.5 | 0.955165107 |
| RALGAPA1 | 3.24 | 2036 | 229.7 | 6.19 | 0.955290012 |
| VAC14 | 3.4 | 214 | 24.8 | 6.93 | 0.955775184 |
| RRM2 | 79.63 | 389 | 44.8 | 5.38 | 0.955904947 |
| PNP | 373.17 | 289 | 32.1 | 6.95 | 0.956467373 |
| LSM1 | 7.03 | 133 | 15.2 | 5.22 | 0.956824086 |
| CTAGE5 | 3.69 | 672 | 76.8 | 5.49 | 0.95702683 |
| DDX49 | 11.19 | 483 | 54.2 | 9.06 | 0.957371024 |
| MFN2 | 0 | 757 | 86.3 | 6.98 | 0.958043797 |
| CHMP1A | 4.76 | 196 | 21.7 | 8.06 | 0.958656213 |
| ATP5I | 36.88 | 69 | 7.9 | 9.35 | 0.958680447 |
| CTR9 | 6.01 | 1173 | 133.4 | 6.77 | 0.959072259 |
| VARS | 330.05 | 1264 | 140.4 | 7.59 | 0.959087031 |
| FERMT3 | 97.24 | 663 | 75.4 | 6.77 | 0.959254924 |
| DHPS | 10.08 | 369 | 40.9 | 5.36 | 0.959618414 |
| GOPC | 14.15 | 454 | 49.7 | 6.07 | 0.95982688 |
| MEMO1 | 19.09 | 274 | 31.3 | 7.09 | 0.959993666 |
| BUB1B | 5.92 | 933 | 105.8 | 5.36 | 0.960683475 |
| SMNDC1 | 3.47 | 238 | 26.7 | 7.24 | 0.960925596 |
| UBLCP1 | 7.59 | 318 | 36.8 | 6.46 | 0.960976119 |
| CNBP | 118.9 | 170 | 18.7 | 7.71 | 0.961256107 |
| PIGT | 2.62 | 367 | 41.9 | 8.56 | 0.962051241 |
| RPAP3 | 44.28 | 631 | 71.8 | 7.64 | 0.962091921 |
| P3H3 | 16.87 | 736 | 81.8 | 6.32 | 0.962708117 |
| VPS33B | 0 | 526 | 60 | 6.42 | 0.962718333 |
| SRA1 | 7.99 | 236 | 25.7 | 7.03 | 0.962732758 |
| UTP3 | 5.19 | 479 | 54.5 | 5.62 | 0.962779313 |
| TUBGCP3 | 6.87 | 824 | 93.8 | 7.8 | 0.9628195 |
| SPTAN1 | 1781.16 | 2472 | 284.4 | 5.35 | 0.962949151 |
| ODF2L | 0 | 513 | 59.8 | 7.01 | 0.963069629 |
| CCDC58 | 10.32 | 144 | 16.6 | 7.81 | 0.963219858 |
| DLST | 184.34 | 453 | 48.7 | 8.95 | 0.963296419 |
| HSDL1 | 4.89 | 330 | 37 | 8.72 | 0.963657204 |
| DDRGK1 | 17.42 | 314 | 35.6 | 5.12 | 0.96400553 |
| USP9X | 51.48 | 2554 | 290.3 | 5.8 | 0.964429952 |
| LSM14B | 8.88 | 385 | 42 | 9.69 | 0.964491243 |
| SRC | 61.84 | 536 | 59.8 | 7.42 | 0.964744685 |
| WAPL | 2.96 | 1190 | 132.9 | 5.44 | 0.965210124 |
| KANSL1L | 2.47 | 945 | 107.4 | 8.16 | 0.965285706 |
| PSMD6 | 113.71 | 389 | 45.5 | 5.62 | 0.965358394 |
| LIG1 | 20.83 | 801 | 88.5 | 5.83 | 0.965502 |
| MCTS1 | 48.71 | 181 | 20.5 | 8.82 | 0.965618319 |
| ADSL | 36.58 | 425 | 48.3 | 7.78 | 0.965880344 |
| MPP6 | 8.86 | 540 | 61.1 | 6.18 | 0.965969351 |
| EXOC7 | 9.36 | 653 | 74.7 | 6.42 | 0.966458304 |
| SEC24C | 71.41 | 1094 | 118.2 | 7.06 | 0.966510505 |
| RBPJ | 4.88 | 411 | 45.6 | 6.64 | 0.966543196 |
| CACNG6 | 3.39 | 260 | 28.1 | 9.17 | 0.966862307 |
| PLA2G4A | 16.34 | 749 | 85.2 | 5.38 | 0.967329584 |
| ASB9 | 4.11 | 252 | 26.8 | 6.58 | 0.967331934 |
| AP3M1 | 21.25 | 418 | 46.9 | 6.93 | 0.967542315 |
| PLPP1 | 2.45 | 284 | 32.1 | 7.97 | 0.968016945 |
| VPS11 | 5.07 | 941 | 107.8 | 7.05 | 0.968710283 |
| YARS2 | 50.16 | 477 | 53.2 | 8.98 | 0.969165923 |
| WDR74 | 7.47 | 366 | 40.2 | 8.38 | 0.969430128 |
| MRPS11 | 3.5 | 193 | 20.4 | 10.65 | 0.969483485 |
| NDUFS5 | 16.81 | 106 | 12.5 | 9.14 | 0.969918662 |
| TP53BP1 | 29.26 | 1972 | 213.4 | 4.7 | 0.969970832 |
| FAR1 | 2.21 | 515 | 59.3 | 9.17 | 0.970185759 |
| MRPL54 | 2.39 | 138 | 15.8 | 9.6 | 0.970197298 |
| CYP51A1 | 36.84 | 404 | 46.3 | 8.5 | 0.97034687 |
| ARFGEF2 | 24.17 | 1785 | 201.9 | 6.33 | 0.970427911 |
| SNTB2 | 12.38 | 267 | 27.7 | 9.86 | 0.971167862 |
| MRPL13 | 8.11 | 178 | 20.7 | 9.16 | 0.971270065 |
| BRK1 | 3.5 | 75 | 8.7 | 5.45 | 0.971375169 |
| CDC5L | 12.4 | 802 | 92.2 | 8.18 | 0.971600373 |
| VPS45 | 6.74 | 570 | 65 | 8.24 | 0.971792234 |
| CISD2 | 33.7 | 135 | 15.3 | 9.61 | 0.972383507 |
| AARS | 243.09 | 968 | 106.7 | 5.53 | 0.972391011 |
| PCNP | 9.47 | 178 | 18.9 | 7.49 | 0.972480564 |
| PELP1 | 23.63 | 1130 | 119.6 | 4.34 | 0.972719746 |
| DFFA | 56.72 | 331 | 36.5 | 4.79 | 0.972824441 |
| TIMM29 | 4.89 | 260 | 29.2 | 8.09 | 0.973835818 |
| CHMP4A | 23.88 | 222 | 25.1 | 4.7 | 0.974068611 |
| NDUFAF3 | 10.83 | 184 | 20.3 | 8.22 | 0.9741649 |
| RPRD1B | 13.72 | 326 | 36.9 | 5.97 | 0.974206016 |
| SDHC | 5.61 | 116 | 12.5 | 8.98 | 0.974321488 |
| KLC1 | 59.84 | 551 | 62.8 | 5.94 | 0.974927746 |
| STAT1 | 138.25 | 750 | 87.3 | 6.05 | 0.975084882 |
| PLAUR | 3.03 | 281 | 31.2 | 6.43 | 0.975210662 |
| IMP3 | 16.73 | 184 | 21.8 | 9.5 | 0.976028543 |
| SUN1 | 23.09 | 682 | 76.4 | 6.64 | 0.976166515 |
| SRPK1 | 42.11 | 655 | 74.3 | 6.16 | 0.976712457 |
| NACA | 269.01 | 215 | 23.4 | 4.56 | 0.977274778 |
| NAE1 | 20.3 | 445 | 50.6 | 5.27 | 0.97760558 |
| NAP1L4 | 69.04 | 375 | 42.8 | 4.69 | 0.977606798 |
| SNRNP70 | 130.94 | 428 | 50.6 | 9.89 | 0.977672279 |
| KLHDC3 | 0 | 382 | 43.1 | 8.07 | 0.97784916 |
| OCIAD1 | 39.27 | 245 | 27.6 | 7.49 | 0.978446807 |
| ACBD5 | 3.55 | 416 | 46.2 | 5.06 | 0.978588669 |
| MAVS | 12.42 | 540 | 56.5 | 5.52 | 0.979137775 |
| UBQLN4 | 98.34 | 601 | 63.8 | 5.22 | 0.979988684 |
| CSNK2B | 49.03 | 215 | 24.9 | 5.55 | 0.980235813 |
| CD46 | 6.8 | 336 | 36.8 | 7.65 | 0.980602113 |
| GTPBP4 | 102.74 | 634 | 73.9 | 9.5 | 0.980609391 |
| PVR | 8.27 | 364 | 39.3 | 6.21 | 0.980809365 |
| DIAPH2 | 8.03 | 1096 | 124.7 | 6.43 | 0.980832076 |
| NDUFB7 | 5.87 | 137 | 16.4 | 8.92 | 0.981222097 |
| SIRT3 | 3.06 | 399 | 43.5 | 8.7 | 0.981287188 |
| SMPD4 | 6.95 | 502 | 56.8 | 9.14 | 0.981531989 |
| LARP7 | 7.5 | 582 | 66.9 | 9.55 | 0.981722115 |
| VMA21 | 38.5 | 101 | 11.3 | 7.24 | 0.981820603 |
| SSSCA1 | 9.52 | 199 | 21.5 | 5.24 | 0.982020897 |
| XPR1 | 0 | 631 | 73.9 | 7.52 | 0.982421592 |
| MALT1 | 6.8 | 813 | 91 | 5.91 | 0.982455075 |
| CBX1 | 91.85 | 185 | 21.4 | 4.93 | 0.982572706 |
| NUFIP2 | 34.1 | 695 | 76.1 | 8.7 | 0.982581688 |
| CDC123 | 15.79 | 336 | 39.1 | 4.81 | 0.98320827 |
| TOMM5 | 19.24 | 51 | 6 | 9.7 | 0.983519501 |
| UQCC1 | 6.5 | 272 | 31.3 | 8.92 | 0.98364149 |
| SMC3 | 64.84 | 1217 | 141.5 | 7.18 | 0.983867254 |
| MICU2 | 3.53 | 434 | 49.6 | 9.09 | 0.984154336 |
| TMEM2 | 3.24 | 1320 | 147.3 | 8.38 | 0.984226527 |
| GRIPAP1 | 4.82 | 625 | 71.8 | 5.38 | 0.98437902 |
| DDX24 | 76.57 | 859 | 96.3 | 9.06 | 0.984666531 |
| MEAF6 | 4.32 | 191 | 21.6 | 9.32 | 0.985016268 |
| MCMBP | 25.18 | 640 | 72.7 | 5.78 | 0.985284293 |
| ACOT9 | 70.34 | 406 | 46.3 | 7.97 | 0.985415855 |
| UBXN4 | 32.53 | 508 | 56.7 | 6.38 | 0.985568865 |
| BCHE | 0 | 602 | 68.4 | 7.42 | 0.985743143 |
| B4GALT1 | 5.36 | 385 | 42.5 | 8.5 | 0.986002483 |
| ECI2 | 17.53 | 359 | 39.6 | 8.65 | 0.986131743 |
| KNSTRN | 3 | 241 | 26.6 | 8.98 | 0.986259539 |
| NUCB1 | 30.64 | 461 | 53.8 | 5.25 | 0.986664243 |
| NR2F2 | 2.05 | 261 | 29.1 | 6.4 | 0.986766026 |
| KIF1C | 6.94 | 1103 | 122.9 | 6.9 | 0.987012106 |
| UBE2K | 60.97 | 200 | 22.4 | 5.44 | 0.987132989 |
| MLPH | 17.41 | 572 | 62.8 | 6.09 | 0.987405788 |
| FAM162A | 12.47 | 154 | 17.3 | 9.77 | 0.987409649 |
| CDK16 | 24.88 | 496 | 55.7 | 7.62 | 0.987449065 |
| SYNCRIP | 424.96 | 562 | 62.6 | 7.56 | 0.987555853 |
| MRPL21 | 25.32 | 205 | 22.8 | 9.89 | 0.987607261 |
| TRIP13 | 16.63 | 432 | 48.5 | 6.09 | 0.987615596 |
| LGALS3BP | 92.07 | 585 | 65.3 | 5.27 | 0.987839332 |
| TES | 15.78 | 412 | 46.9 | 7.59 | 0.987885076 |
| RFC1 | 16.24 | 1147 | 128.1 | 9.36 | 0.987970028 |
| ACTL6A | 15.59 | 429 | 47.4 | 5.6 | 0.987990408 |
| PDRG1 | 0 | 133 | 15.5 | 6.06 | 0.988020918 |
| LAMP2 | 54.98 | 410 | 44.9 | 5.63 | 0.988473996 |
| SF3A1 | 98.83 | 793 | 88.8 | 5.22 | 0.988588204 |
| EIF2B3 | 3.23 | 401 | 44.8 | 6.65 | 0.988774754 |
| GNPDA1 | 52.23 | 289 | 32.6 | 6.92 | 0.989177664 |
| GTF2A1 | 7.14 | 376 | 41.5 | 4.55 | 0.989232651 |
| LGALSL | 4.3 | 172 | 19 | 5.35 | 0.989288943 |
| ILK | 17.31 | 318 | 36.4 | 9.41 | 0.989350681 |
| HIBADH | 36.15 | 336 | 35.3 | 8.13 | 0.989351563 |
| PDXDC1 | 37.83 | 697 | 76.5 | 5.48 | 0.989353418 |
| MGST3 | 10.05 | 152 | 16.5 | 9.38 | 0.989375656 |
| ARFGAP1 | 3.28 | 293 | 31.5 | 5.41 | 0.989941506 |
| DCUN1D1 | 5.23 | 259 | 30.1 | 5.34 | 0.990316301 |
| FBF1 | 0 | 1133 | 125.4 | 7.08 | 0.99033445 |
| EXOSC7 | 9.84 | 291 | 31.8 | 5.19 | 0.990366035 |
| CPNE3 | 72.99 | 537 | 60.1 | 5.85 | 0.990913914 |
| URB1 | 16.66 | 2271 | 254.2 | 6.47 | 0.990950939 |
| DIAPH1 | 54.06 | 1248 | 138.8 | 5.39 | 0.991234395 |
| EXOC1 | 7.06 | 879 | 100.2 | 6.6 | 0.991367966 |
| FLOT2 | 26.48 | 428 | 47 | 5.25 | 0.991385015 |
| SDCBP | 17.49 | 292 | 31.7 | 7.53 | 0.991926524 |
| DMD | 5.76 | 604 | 68.9 | 6.6 | 0.992178915 |
| DCTN3 | 10.8 | 158 | 18 | 6.05 | 0.992351405 |
| DCP1B | 2.52 | 617 | 67.7 | 8.56 | 0.992470778 |
| UTP18 | 17.88 | 556 | 62 | 8.76 | 0.992506486 |
| SPATA5 | 35.78 | 696 | 75.7 | 5.95 | 0.993056137 |
| DYNLL2 | 32.44 | 89 | 10.3 | 7.37 | 0.993134092 |
| PABPC1L | 141.63 | 614 | 68.3 | 8.87 | 0.993143859 |
| RBM42 | 19.27 | 446 | 47 | 9.5 | 0.993220696 |
| SH3BGRL3 | 94.21 | 93 | 10.4 | 4.93 | 0.993263773 |
| NSFL1C | 83.8 | 370 | 40.5 | 5.1 | 0.993288289 |
| OSBPL8 | 14.82 | 847 | 96.9 | 7.55 | 0.993344059 |
| BUD31 | 17.24 | 144 | 17 | 8.82 | 0.99379751 |
| CD9 | 2.33 | 228 | 25.4 | 7.15 | 0.993880526 |
| MAPKAPK2 | 1.97 | 400 | 45.5 | 8.68 | 0.994558821 |
| APOBEC3C | 5.03 | 190 | 22.8 | 7.59 | 0.994980539 |
| FUBP3 | 137.08 | 572 | 61.6 | 8.38 | 0.995049817 |
| C21orf33 | 18.89 | 268 | 28.2 | 8.27 | 0.995196009 |
| DLGAP5 | 12.7 | 765 | 85.6 | 8.7 | 0.995585726 |
| MLKL | 5.4 | 471 | 54.4 | 8.82 | 0.995678251 |
| PMPCB | 55.92 | 489 | 54.3 | 6.83 | 0.995965456 |
| MACF1 | 7.39 | 7388 | 837.8 | 5.39 | 0.997129139 |
| ATP6V1H | 5.66 | 465 | 54.1 | 6.48 | 0.997496229 |
| IFI16 | 44.09 | 785 | 88.2 | 9.28 | 0.997646194 |
| SNW1 | 37.75 | 536 | 61.5 | 9.52 | 0.998379035 |
| ATAD3B | 116.72 | 648 | 72.5 | 9.2 | 0.998532789 |
| AASDHPPT | 39.83 | 309 | 35.8 | 6.8 | 0.998701258 |
| TM9SF1 | 4.39 | 489 | 55.2 | 7.06 | 0.998962322 |
| TBC1D24 | 2.59 | 553 | 62.3 | 7.21 | 0.99907458 |
| SEPHS1 | 23.57 | 392 | 42.9 | 5.97 | 0.999404839 |
| RRN3P1 | 2.23 | 152 | 17.2 | 8.79 | 0.999492872 |
| ATP6V0A1 | 11.74 | 831 | 95.7 | 6.65 | 0.999745077 |

| **Supplementary** **Table 3.** CLPTM1L target genes after screening | | | | |  |
| --- | --- | --- | --- | --- | --- |
| Gene name | Score | # AAs | MW [kDa] | calc. pI | p_value |
| HIST1H4A | 1096.62 | 103 | 11.4 | 11.36 | 3.87E-103 |
| RPL15 | 144.17 | 204 | 24.1 | 11.62 | 3.75E-75 |
| HIST1H3A | 362.05 | 136 | 15.4 | 11.12 | 5.98E-45 |
| RPL6 | 173.39 | 288 | 32.7 | 10.58 | 1.22E-44 |
| S100A10 | 80.69 | 97 | 11.2 | 7.37 | 2.64E-25 |
| RPL18 | 134.03 | 188 | 21.6 | 11.72 | 4.84E-18 |
| KRT10 | 102.13 | 584 | 58.8 | 5.21 | 1.01E-15 |
| KRT9 | 70.28 | 623 | 62 | 5.24 | 6.11E-15 |
| RPL14 | 116.52 | 215 | 23.4 | 10.93 | 1.07E-14 |
| KRT1 | 236.11 | 644 | 66 | 8.12 | 1.3E-14 |
| RPL27 | 203.94 | 136 | 15.8 | 10.56 | 3E-11 |
| MIF | 106.54 | 115 | 12.5 | 7.88 | 8.58E-10 |
| INTS7 | 4.12 | 913 | 101.1 | 7.8 | 9.73E-09 |
| CLCC1 | 3.75 | 366 | 39.8 | 5.74 | 4.95E-08 |
| MICAL3 | 2.08 | 2002 | 224.2 | 5.55 | 9.28E-08 |
| TUBA1A | 2989.93 | 416 | 46.3 | 5.08 | 9.51E-08 |
| ZBED1 | 0 | 694 | 78.1 | 6.1 | 0.000000121 |
| NDUFA8 | 38.87 | 172 | 20.1 | 7.65 | 0.000000257 |
| BIN1 | 4.6 | 409 | 45.5 | 5.49 | 0.000000391 |
| AGTRAP | 5.23 | 152 | 16.7 | 6.28 | 0.000000545 |
| NUDT1 | 3.94 | 156 | 17.9 | 5.08 | 0.000000827 |
| BTAF1 | 2.71 | 1849 | 206.8 | 6.52 | 0.00000112 |
| RPF2 | 8.47 | 306 | 35.6 | 9.99 | 0.0000016 |
| PLEKHG3 | 0 | 1163 | 128.2 | 6.76 | 0.00000197 |
| PARP4 | 3.05 | 1724 | 192.5 | 5.66 | 0.000002 |
| SUGP2 | 2.72 | 1030 | 115.1 | 8.63 | 0.00000245 |
| NME2 | 871.97 | 267 | 30.1 | 8.92 | 0.00000304 |
| YTHDC2 | 5.2 | 1430 | 160.1 | 8.4 | 0.0000031 |
| OSBPL9 | 2.44 | 719 | 81.1 | 5.82 | 0.00000402 |
| DDX28 | 2.27 | 540 | 59.5 | 10.42 | 0.00000604 |
| MRPL10 | 4.27 | 261 | 29.3 | 9.58 | 0.00000708 |
| MAPK3 | 13.7 | 335 | 38.3 | 6.2 | 0.00000817 |
| BUD23 | 13.64 | 281 | 31.9 | 8.73 | 0.00000922 |
| PRIM2 | 3.13 | 509 | 58.8 | 7.91 | 0.0000101 |
| DDT | 38.08 | 118 | 12.7 | 7.3 | 0.000012 |
| ABHD14B | 41.61 | 210 | 22.3 | 6.4 | 0.0000122 |
| GINS3 | 2.38 | 138 | 15.6 | 4.93 | 0.0000124 |
| ACOX1 | 8.44 | 622 | 70.1 | 7.61 | 0.0000148 |
| DOHH | 4.09 | 302 | 32.9 | 4.83 | 0.0000162 |
| POP1 | 2.9 | 1024 | 114.6 | 9.22 | 0.0000185 |
| NDUFB1 | 3.2 | 58 | 7 | 8.92 | 0.0000186 |
| UEVLD | 0 | 215 | 24.2 | 7.88 | 0.0000195 |
| COX7A2 | 25.37 | 83 | 9.4 | 9.76 | 0.0000211 |
| GOLT1B | 36.48 | 138 | 15.4 | 10.36 | 0.0000233 |
| TEAD1 | 3.56 | 357 | 40 | 8.32 | 0.0000256 |
| MRPL57 | 0 | 102 | 12.3 | 11.44 | 0.0000397 |
| UBR5 | 18.45 | 2798 | 309 | 5.85 | 0.0000411 |
| GANAB | 434.95 | 944 | 106.8 | 6.14 | 0.0000424 |
| TMEM33 | 123.84 | 247 | 28 | 9.7 | 0.0000573 |
| EIF1AY | 97.73 | 144 | 16.4 | 5.24 | 0.0000645 |
| BAZ2A | 3.48 | 1878 | 208.3 | 6.6 | 0.0000845 |
| PEX3 | 6.49 | 373 | 42.1 | 8.15 | 0.0000891 |
| PAK4 | 2.98 | 426 | 47.9 | 9.47 | 0.000094 |
| POTEF | 1486.85 | 1075 | 121.4 | 6.2 | 0.0000982 |
| RBBP5 | 6.83 | 500 | 55 | 4.96 | 0.000109045 |
| DCTN4 | 14.79 | 460 | 52.3 | 7.34 | 0.000122235 |
| DVL2 | 2.04 | 736 | 78.9 | 6.02 | 0.000153285 |
| CHRAC1 | 2.06 | 131 | 14.7 | 5.1 | 0.000162158 |
| PDE3B | 0 | 1061 | 118.7 | 5.8 | 0.000219284 |
| C11orf54 | 3.08 | 204 | 23.2 | 6.79 | 0.00024384 |
| COA7 | 2.67 | 231 | 25.7 | 6.02 | 0.000245469 |
| RPL7L1 | 3.56 | 153 | 18 | 11.06 | 0.000246733 |
| TMEM56 | 0 | 263 | 30 | 9.33 | 0.000253755 |
| THOC2 | 37.28 | 1593 | 182.7 | 8.44 | 0.000285695 |
| PTCD1 | 2.3 | 700 | 78.8 | 8.59 | 0.000303539 |
| FAM210B | 6.13 | 192 | 20.4 | 10.43 | 0.000306085 |
| CENPX | 2.5 | 81 | 9 | 5.9 | 0.000321457 |
| METTL13 | 3.22 | 396 | 44.5 | 5.63 | 0.000339513 |
| ISOC1 | 11.59 | 298 | 32.2 | 7.39 | 0.000345032 |
| FAM83E | 0 | 478 | 51.7 | 9.35 | 0.000377633 |
| USP6 | 2.46 | 1089 | 121.9 | 7.46 | 0.000450823 |
| CAMK1 | 5.33 | 370 | 41.3 | 5.29 | 0.000517612 |
| SAAL1 | 0 | 474 | 53.5 | 4.5 | 0.000526474 |
| PISD | 0 | 375 | 43 | 9.7 | 0.000538991 |
| TSEN15 | 3.27 | 171 | 18.6 | 4.58 | 0.000576804 |
| MAFF | 2.14 | 135 | 14.6 | 10.15 | 0.000646637 |
| PPP4R3A | 21.06 | 820 | 93.8 | 4.89 | 0.000651468 |
| GXYLT1 | 5.58 | 409 | 46.8 | 8.95 | 0.000840987 |
| UBP1 | 7 | 504 | 56.4 | 6.29 | 0.000916324 |
| RMDN1 | 5.09 | 284 | 32.3 | 8.94 | 0.000945582 |
| CD14 | 0 | 375 | 40.1 | 6.23 | 0.000983474 |
| UBAC2 | 4.28 | 157 | 18 | 8.03 | 0.000993352 |
| MANBAL | 2.74 | 85 | 9.5 | 9.16 | 0.001028414 |
| KIF3C | 10.78 | 793 | 89.4 | 8.22 | 0.001039488 |
| GDAP2 | 2.03 | 496 | 56 | 6.16 | 0.001047695 |
| TRIO | 5.1 | 2563 | 287.2 | 6.34 | 0.001160912 |
| RAB34 | 6.34 | 198 | 21.1 | 12.16 | 0.001251442 |
| MRPS33 | 2.98 | 106 | 12.6 | 10.11 | 0.001276607 |
| SRGAP3 | 0 | 1075 | 121.7 | 6.58 | 0.001363638 |
| DR1 | 4 | 176 | 19.4 | 4.75 | 0.001416533 |
| NOLC1 | 301.09 | 700 | 73.7 | 9.47 | 0.001423515 |
| MTFR1L | 3.02 | 280 | 30.8 | 6.32 | 0.001465963 |
| PGR | 5.74 | 933 | 99 | 6.09 | 0.00146987 |
| TXN2 | 21.04 | 166 | 18.4 | 8.29 | 0.001470948 |
| UNC93B1 | 2.48 | 597 | 66.6 | 6.96 | 0.00148327 |
| NBAS | 2.12 | 2251 | 254.7 | 5.94 | 0.001492503 |
| KIRREL1 | 4.83 | 757 | 83.5 | 5.73 | 0.001678126 |
| ZC3HC1 | 8.93 | 431 | 47.7 | 5.19 | 0.001759254 |
| AHCYL2 | 46.31 | 508 | 56.7 | 8.09 | 0.001763465 |
| KCMF1 | 5.62 | 381 | 41.9 | 5.66 | 0.00208832 |
| TACC2 | 4.93 | 571 | 64.1 | 5.33 | 0.00223444 |
| KRT2 | 57.84 | 639 | 65.4 | 8 | 0.002240147 |
| CDK5RAP1 | 2.52 | 426 | 48 | 8.18 | 0.00226263 |
| ARMC10 | 2.74 | 225 | 24.7 | 7.46 | 0.002351871 |
| WNK1 | 0 | 1975 | 206.5 | 6.15 | 0.002397564 |
| HIST1H1C | 252.4 | 213 | 21.4 | 10.93 | 0.002502005 |
| CHMP2A | 2.41 | 222 | 25.1 | 5.97 | 0.002514607 |
| EGFR | 51.36 | 1091 | 120.7 | 6.37 | 0.002596 |
| CHST14 | 2.69 | 376 | 43 | 9.48 | 0.002724982 |
| PSMG2 | 37.93 | 233 | 26.3 | 7.96 | 0.003089937 |
| MT1L | 0 | 61 | 6.1 | 7.96 | 0.003122981 |
| CFDP1 | 0 | 217 | 24 | 4.49 | 0.003337637 |
| TMEM70 | 9.35 | 161 | 18.6 | 6.8 | 0.003435879 |
| SRGAP2 | 2.85 | 1071 | 120.8 | 6.7 | 0.00353 |
| P4HA2 | 0 | 533 | 60.6 | 5.71 | 0.003731734 |
| MED23 | 0 | 1359 | 155.5 | 7.4 | 0.003827892 |
| N/A | 2.4 | 355 | 38 | 5.26 | 0.003955904 |
| MYC | 366.12 | 453 | 50.4 | 5.28 | 0.004125 |
| CC2D1A | 6.9 | 950 | 103.9 | 8.09 | 0.004524658 |
| DNM1L | 24.67 | 699 | 78.1 | 6.81 | 0.004642174 |
| SNAT2 | 0 | 406 | 45.2 | 8.89 | 0.00469742 |
| ANKRD12 | 0 | 2039 | 232.9 | 7.06 | 0.004756851 |
| GIPC1 | 2 | 236 | 26.1 | 5.57 | 0.005007568 |
| CASP2 | 2.6 | 313 | 34.9 | 6.52 | 0.005205913 |
| SSR2 | 0 | 183 | 20.1 | 8.35 | 0.005363726 |
| CDC42BPA | 66.46 | 1741 | 197.8 | 6.06 | 0.005413699 |
| CD44 | 88.46 | 742 | 81.5 | 5.13 | 0.005476 |
| ZDHHC5 | 0 | 662 | 71.9 | 8.9 | 0.00567113 |
| JAK1 | 2.44 | 1154 | 133.3 | 7.48 | 0.006489315 |
| KLHL38 | 2.04 | 581 | 65.5 | 7.93 | 0.006882195 |
| TSPYL1 | 2.37 | 437 | 49.2 | 5.45 | 0.007226387 |
| GLUD1 | 106.55 | 558 | 61.4 | 7.8 | 0.007307747 |
| COPS8 | 41.34 | 160 | 17.9 | 7.25 | 0.007411608 |
| ORC3 | 4.02 | 568 | 65.9 | 7.62 | 0.007517209 |
| ABCB4 | 86.14 | 1349 | 147.8 | 8.93 | 0.00769845 |
| GUK1 | 2.44 | 197 | 21.7 | 6.55 | 0.007740704 |
| C7orf50 | 31.81 | 194 | 22.1 | 9.64 | 0.008112121 |
| PPP4R1 | 9.45 | 933 | 105.1 | 4.81 | 0.008332332 |
| NBEAL2 | 1.7 | 2570 | 282.7 | 6.54 | 0.008549896 |
| HRAS | 41.64 | 189 | 21.3 | 5.16 | 0.00864631 |
| HIST1H1E | 274.55 | 219 | 21.9 | 11.03 | 0.00866412 |
| ITGA1 | 2.25 | 1179 | 130.8 | 6.29 | 0.008819033 |
| NOL9 | 2.51 | 702 | 79.3 | 9.13 | 0.009235503 |
| CDC25A | 981.72 | 524 | 59.1 | 6.49 | 0.00924646 |
| TUBG1 | 29.12 | 451 | 51.1 | 6.14 | 0.009435855 |
| EIF2B4 | 13.08 | 522 | 57.4 | 9.38 | 0.009537342 |
| REPS1 | 2.8 | 705 | 76.8 | 5.83 | 0.009632046 |
| MRRF | 13.81 | 201 | 22.4 | 9.72 | 0.009717356 |
| MPC2 | 0 | 127 | 14.3 | 10.43 | 0.010450667 |
| EAPP | 3.16 | 285 | 32.7 | 5.12 | 0.010678783 |
| EPHB4 | 5.27 | 987 | 108.2 | 6.9 | 0.010802511 |
| ISG15 | 4.49 | 165 | 17.9 | 7.44 | 0.011356307 |
| DPH5 | 5.53 | 234 | 26.1 | 5.02 | 0.011521087 |
| PIK3CB | 2.45 | 1070 | 122.7 | 7.09 | 0.012193763 |
| FN3KRP | 2.09 | 309 | 34.4 | 7.33 | 0.013047527 |
| NT5C3A | 4.8 | 285 | 32.5 | 5.72 | 0.013547693 |
| TIMM8B | 2.91 | 83 | 9.3 | 5.12 | 0.014042304 |
| HNRNPU | 990.18 | 806 | 88.9 | 5.78 | 0.014342838 |
| ATP5G1 | 22.6 | 136 | 14.3 | 9.74 | 0.014399209 |
| RBM6 | 6.37 | 601 | 69.1 | 8.73 | 0.014502753 |
| COX20 | 9.98 | 118 | 13.3 | 8.76 | 0.014645019 |
| TFF1 | 41.5 | 84 | 9.1 | 4.29 | 0.0146845 |
| GDAP1L1 | 0 | 367 | 41.9 | 6.6 | 0.01494289 |
| RALA | 14.88 | 206 | 23.6 | 7.11 | 0.01523177 |
| ODF2 | 0 | 657 | 75.6 | 7.62 | 0.01559727 |
| ADNP | 2.29 | 1102 | 123.5 | 7.34 | 0.015793782 |
| COQ8A | 2.14 | 163 | 18.9 | 6.68 | 0.015851337 |
| TSR3 | 4.11 | 312 | 33.6 | 6.87 | 0.016448502 |
| CCNT1 | 0 | 726 | 80.6 | 8.78 | 0.016806329 |
| SFXN2 | 2.23 | 322 | 36.2 | 9.41 | 0.017593377 |
| CPNE9 | 17.28 | 553 | 61.8 | 5.34 | 0.017902367 |
| PNISR | 2.16 | 413 | 47.4 | 5.17 | 0.018067792 |
| GSTM2 | 59.01 | 191 | 22.6 | 5.31 | 0.018259347 |
| MAU2 | 0 | 189 | 21.3 | 6.71 | 0.018457189 |
| MRPS12 | 4.7 | 138 | 15.2 | 10.29 | 0.018843348 |
| MRPL2 | 5.08 | 305 | 33.3 | 11.3 | 0.019128252 |
| STAG2 | 20.99 | 1231 | 141.2 | 5.43 | 0.019238057 |
| POLK | 0 | 371 | 42.3 | 6.65 | 0.019744616 |
| C8orf82 | 3.58 | 208 | 22.8 | 11.02 | 0.019881329 |
| ORC2 | 3.75 | 577 | 65.9 | 6.51 | 0.020381248 |
| PRKCB | 4.28 | 671 | 76.8 | 7.01 | 0.021048985 |
| RDH13 | 7.91 | 331 | 35.9 | 8.1 | 0.022133596 |
| ZCCHC3 | 2.57 | 404 | 43.6 | 8.53 | 0.022136531 |
| NDUFA12 | 4.3 | 145 | 17.1 | 9.63 | 0.022181927 |
| MCFD2 | 3.63 | 94 | 10.7 | 4.41 | 0.022996253 |
| NCAPG | 52.45 | 1015 | 114.3 | 5.59 | 0.023368979 |
| CDIPT | 5.01 | 168 | 18.6 | 8.21 | 0.024080543 |
| UBE2I | 52.37 | 158 | 18 | 8.66 | 0.024225155 |
| JUN | 28.47 | 331 | 35.7 | 8.9 | 0.024569878 |
| PRSS3P2 | 10.62 | 247 | 26.5 | 6.01 | 0.024866618 |
| TOR4A | 0 | 423 | 46.9 | 9.94 | 0.025593772 |
| ECSIT | 9.55 | 296 | 33.2 | 9.13 | 0.026053659 |
| BCL2 | 272.44 | 239 | 26.3 | 6.75 | 0.02648134 |
| ULBP2 | 2.32 | 246 | 27.3 | 7.3 | 0.026689886 |
| MRPL42 | 3.84 | 142 | 16.7 | 8.35 | 0.026844358 |
| NEB | 0 | 6669 | 772.4 | 9.07 | 0.027068216 |
| CHAMP1 | 9.36 | 812 | 89 | 8.44 | 0.027123178 |
| ARG1 | 0 | 330 | 35.6 | 7.21 | 0.027831032 |
| VEGFA | 11.145 | 412 | 45.5 | 9.22 | 0.027843156 |
| TLE3 | 3.71 | 760 | 82.2 | 7.2 | 0.028231685 |
| NOL7 | 0 | 145 | 16.3 | 4.87 | 0.029154733 |
| RABEPK | 2.02 | 321 | 34.8 | 6 | 0.029549384 |
| TMEM120A | 0 | 322 | 35.9 | 8.43 | 0.030989971 |
| RIC8A | 19.42 | 530 | 59.6 | 5.33 | 0.031176344 |
| TAMM41 | 0 | 316 | 35.9 | 8.75 | 0.031337043 |
| RHBDD2 | 3.95 | 364 | 39.2 | 9.32 | 0.031882899 |
| VTI1B | 0 | 232 | 26.7 | 9.04 | 0.033986421 |
| MAPK1 | 32.14 | 360 | 41.4 | 6.5 | 0.03489 |
| MED12 | 0 | 2176 | 242.8 | 7.05 | 0.035019456 |
| STK24 | 22.58 | 431 | 47.9 | 5.43 | 0.035034747 |
| CLPTM1L | 69.81 | 502 | 58.3 | 8.82 | 0.035281401 |
| STK17A | 0 | 414 | 46.5 | 5.15 | 0.03532259 |
| RBBP6 | 0 | 1758 | 197.2 | 9.63 | 0.036421144 |
| SLC38A1 | 0 | 487 | 54 | 7.02 | 0.036549393 |
| PNPLA6 | 0 | 1300 | 143.3 | 7.74 | 0.037267578 |
| FNBP4 | 2.14 | 1017 | 110.2 | 4.74 | 0.037672853 |
| HS3ST6 | 0 | 342 | 37.2 | 10.78 | 0.037765795 |
| TIGAR | 10.89 | 270 | 30 | 7.69 | 0.040256428 |
| ATP2B4 | 1.24 | 1170 | 129.4 | 7.45 | 0.04137649 |
| BRCA1 | 2.47 | 1863 | 207.7 | 5.29 | 0.04348795 |
| LIN7A | 16.93 | 233 | 26 | 8.72 | 0.043770325 |
| TSEN34 | 4.8 | 310 | 33.6 | 8.43 | 0.044128303 |
| PRIM1 | 10.5 | 420 | 49.9 | 8.21 | 0.045157571 |
| SPART | 17.48 | 666 | 72.8 | 5.91 | 0.046267789 |
| HAUS5 | 0 | 290 | 32.6 | 7.56 | 0.046274086 |
| FOS | 19.5 | 380 | 40.7 | 4.77 | 0.047156846 |
| TP53 | 4.03 | 393 | 43.7 | 6.33 | 0.04826479 |
| MRPS14 | 6.59 | 128 | 15.1 | 11.41 | 0.048612282 |
| NME2P1 | 341.86 | 137 | 15.5 | 8.57 | 0.04866753 |
| PKMYT1 | 5.54 | 430 | 47.3 | 6.06 | 0.048877909 |
| UACA | 5.17 | 1403 | 161.4 | 6.93 | 0.048906491 |
| CDS2 | 4.18 | 445 | 51.4 | 7.09 | 0.049790732 |
| PRNP | 9.27 | 246 | 26.9 | 9.11 | 0.049963946 |

| **Supplementary** **Table 4.** Irradiation-related genes |
| --- |
| Gene |
| ABCA1 |
| ABCB4 |
| ACADM |
| ACHE |
| ACP5 |
| ACSL4 |
| ACTA1 |
| ACVRL1 |
| ACY1 |
| ACYP2 |
| ADAM12 |
| ADAM9 |
| ADH6 |
| ADORA2A |
| AGL |
| AHR |
| AHR|LASS4 |
| AHSG |
| AKAP13 |
| AKAP6 |
| ALCAM |
| ALDH3B2 |
| ALDOB |
| ALOX5 |
| ALOX5AP |
| ALPI |
| AMPD3 |
| AMPH |
| AMY1A |
| ANK1 |
| ANK2 |
| ANK3 |
| ANKHD1 |
| AOAH |
| AOF1 |
| AP3B1 |
| AP3B2 |
| APC |
| APOC2 |
| AQP5 |
| AQP9|RPAIN |
| ARFGEF1 |
| ARHGEF5 |
| ARHGEF6 |
| ARID4A |
| ARID4B |
| ARL16 |
| ARSD |
| ART3 |
| ASGR2 |
| ASPA |
| ATF3 |
| ATM |
| ATP2B1 |
| ATP2B4 |
| ATP7A |
| ATR |
| ATRX |
| ATXN7 |
| AURKA |
| AURKB |
| AVPR1A |
| AZGP1 |
| BACE1 |
| BARD1 |
| BAZ1A |
| BAZ1B |
| BCHE |
| BCKDHB |
| BCL2 |
| BDKRB2 |
| BDNF |
| BIRC2 |
| BIRC3 |
| BIRC6 |
| BLZF1 |
| BMP6 |
| BMP8A |
| BPI |
| BRAF |
| BRCA1 |
| BRPF1|N/A |
| BST1 |
| BTAF1 |
| BTG2 |
| BTK |
| BUB1 |
| BUB1B |
| C10orf10 |
| C13orf24 |
| C18orf56|CLUL1 |
| C19orf56 |
| C1orf61 |
| C1QB |
| C2 |
| C22orf31 |
| C2orf3|N/A |
| C4BPA |
| C5 |
| C6 |
| C6orf12 |
| C9orf61 |
| CA1 |
| CA6 |
| CACNA1D |
| CACNA2D2 |
| CAD |
| CAMK4 |
| CAND1 |
| CAND2 |
| CAPZA1 |
| CASP8AP2 |
| CASR |
| CAST |
| CBLB |
| CBLN1 |
| CBR4|N/A |
| CBX6 |
| CCDC6 |
| CCL15 |
| CCL4L2 |
| CCL7 |
| CCNA2 |
| CCNB1 |
| CCNB2 |
| CCND2 |
| CCNF |
| CCNT1 |
| CCNT2 |
| CCPG1 |
| CCR6 |
| CCT6A |
| CD14 |
| CD163 |
| CD1C |
| CD34 |
| CD36 |
| CD37 |
| CD3D |
| CD3G |
| CD40|N/A |
| CD44 |
| CD48 |
| CD52 |
| CD6 |
| CD79A |
| CD79B |
| CD8A |
| CDC14A |
| CDC2 |
| CDC25A |
| CDC25C |
| CDC42BPA |
| CDC5L |
| CDC6 |
| CDC7 |
| CDH3 |
| CDH5 |
| CDK6 |
| CDKL5 |
| CDKN1A |
| CDO1 |
| CEACAM1 |
| CEACAM5 |
| CENPA |
| CENPE |
| CENPF |
| CENPN|B4GALT1 |
| CENTB2 |
| CEPT1|N/A |
| CERKL|COX7B |
| CFB |
| CFH |
| CFL2 |
| CFP |
| CHD2 |
| CHGA |
| CHI3L1 |
| CHI3L2 |
| CHML|N/A |
| CHRNA4 |
| CIITA |
| CIR |
| CKS2 |
| CLDN10 |
| CLGN |
| CLOCK |
| CLPS |
| CLPTM1L |
| CNR1 |
| COCH |
| COL11A1 |
| COL11A2 |
| COL16A1 |
| COL1A1|TUBB |
| COL4A1 |
| COL4A4 |
| COL6A1 |
| COL8A2 |
| COPA|IRAK1 |
| COPS2 |
| CPEB4 |
| CPN1 |
| CPN2 |
| CPNE6 |
| CPS1 |
| CPSF1 |
| CPT1B |
| CR2 |
| CRHR1 |
| CRMP1 |
| CROP |
| CRP |
| CRYBA1 |
| CRYM |
| CSE1L |
| CSF1R |
| CSNK1G3 |
| CSTF3 |
| CTDSPL2 |
| CTNNA1 |
| CTNNA2 |
| CTNNAL1 |
| CTSD |
| CTSK |
| CTSO |
| CUL2 |
| CX3CL1 |
| CXCL1 |
| CXCL10 |
| CXCL12 |
| CXCL3 |
| CXCR7|N/A |
| CYP11B1 |
| CYP17A1 |
| CYP1A1 |
| CYP21A2 |
| CYP24A1|N/A |
| CYP27B1 |
| CYP2A6 |
| CYP2C8 |
| CYP2C9 |
| CYP2J2 |
| CYP3A7 |
| CYP4A11|SLC4A7 |
| D21S2056E |
| DAB2 |
| DAPK3 |
| DAZL |
| DCT|EGR1 |
| DDAH2 |
| DDB2 |
| DDC |
| DDX17 |
| DDX21 |
| DDX58 |
| DGKA |
| DHX9 |
| DIRAS3 |
| DLD |
| DLK1 |
| DLX4 |
| DNAH11|HIP1R |
| DNAJA2 |
| DNMT3B |
| DNTTIP2 |
| DOCK1 |
| DOPEY1 |
| DPYD |
| DSCR1L1 |
| DSP |
| DUSP3 |
| DYNC1I1 |
| ECE1 |
| EDG1 |
| EDN3 |
| EDNRA |
| EEA1 |
| EGFR |
| EGR3 |
| EIF2B1|ATG10 |
| EIF2C1 |
| EIF3S6 |
| EIF5A |
| ELA2A |
| ELA3A |
| ELF2 |
| ELK4 |
| ELL2 |
| EMR1 |
| ENG |
| ENPP1 |
| ENTPD1 |
| EPC2|PPP1R3C |
| EPHB6 |
| EPS8 |
| ERCC5 |
| EREG |
| ESM1 |
| EVI2A |
| EXO1 |
| EXOC5|N/A |
| EXTL1 |
| EYA2 |
| EYA3 |
| F11 |
| FABP1 |
| FAM107A |
| FAM48A |
| FAM82B|PTMA |
| FANCC |
| FBLN2 |
| FBN1 |
| FBXO2 |
| FCGR2B |
| FGA |
| FGB |
| FGF12 |
| FGF2 |
| FGFBP1 |
| FHIT |
| FILIP1L |
| FKBP8 |
| FLT1 |
| FMO1 |
| FMO5 |
| FOLR2 |
| FOLR3 |
| FOS |
| FOXF2 |
| FOXO1A |
| FPR1 |
| FST |
| FTCD |
| FUT4 |
| FXR1 |
| FXYD2 |
| FZD6 |
| FZR1 |
| GABPA |
| GABRA1 |
| GABRA6 |
| GABRE |
| GADD45B |
| GALC |
| GALNT3 |
| GAS1 |
| GATA3 |
| GBP1 |
| GC |
| GCDH |
| GCH1 |
| GCKR |
| GJA1 |
| GJA4 |
| GJB2 |
| GLUL |
| GMFB |
| GNRH1 |
| GNS |
| GOLGA4 |
| GOLGA8A |
| GOLGB1 |
| GP2 |
| GPC6 |
| GPD2 |
| GPLD1 |
| GPM6B |
| GPR109B |
| GPR143 |
| GPR162 |
| GPR19 |
| GRIK3 |
| GSK3A |
| GSR |
| GSTA2 |
| GTPBP1 |
| GUCY1A3|N/A |
| GYPB |
| GZMH |
| H2AFX |
| HAAO |
| HAT1 |
| HBP1 |
| HCK |
| HCLS1 |
| HCRTR1 |
| HFE |
| HIBCH |
| HIPK3 |
| HIST1H4B |
| HIVEP2 |
| HLA-A |
| HLA-DMB |
| HLA-DOA |
| HLA-DQB1 |
| HLA-DRB1 |
| HLF |
| HLTF |
| HMG2L1 |
| HMMR |
| HMMR|N/A |
| HNRPA3 |
| HNRPC |
| HOMER1 |
| HOXA2 |
| HPD |
| HPN |
| HRAS |
| HRG |
| HS2ST1 |
| HS3ST3A1|N/A |
| HSD17B3 |
| HSD17B6 |
| HSD3B1 |
| HSF2BP |
| HSP90B1 |
| HSPA1L |
| HSPA4L |
| HSPB2 |
| HSPH1 |
| HTR2B |
| HTR2C |
| HTR3A |
| HYAL1 |
| ICA1 |
| ICAM2|C17orf72 |
| ID2 |
| ID3 |
| IDE |
| IFI16 |
| IFIT2 |
| IFITM1 |
| IFT88 |
| IGF2 |
| IGHG1 |
| IGL@ |
| IGSF4 |
| IL15 |
| IL18 |
| IL1A |
| IL1B |
| IL1RAP |
| IL1RN |
| IL2RB |
| IL2RG |
| IL6ST |
| IL8|N/A |
| INDO |
| INPP4B|N/A |
| INSL4 |
| IQGAP1 |
| IQGAP2 |
| IREB2 |
| ITCH |
| ITGA2 |
| ITGA2B |
| ITGA6 |
| ITGAL |
| ITGB1 |
| ITGB2 |
| ITGB4 |
| ITGB8 |
| ITGBL1 |
| ITPR1 |
| ITPR2 |
| JAK1 |
| JMJD1C |
| JUN |
| KAL1 |
| KCNA1 |
| KCNH2 |
| KDR |
| KIAA0226 |
| KIF23 |
| KIF2C |
| KIF3A |
| KIFC1 |
| KIN |
| KLF7 |
| KLRC2 |
| KLRC3 |
| KMO |
| KNG1 |
| KRT5 |
| KYNU |
| LAD1 |
| LAMC1 |
| LARGE |
| LBR |
| LCAT |
| LCP2 |
| LGALS7 |
| LHX1 |
| LIG1 |
| LIMK1 |
| LIMK2 |
| LIPC |
| LLGL1 |
| LMO7 |
| LNPEP |
| LOC643576|PGAM1 |
| LOC647057 |
| LOC728424 |
| LOC728643 |
| LRP5 |
| LTBP2 |
| LUM |
| LYZ |
| MAL |
| MALAT1 |
| MAN1A1 |
| MAN1A2 |
| MAN2A1 |
| MAOB |
| MAP1B |
| MAP2K6 |
| MAP3K10 |
| MAP4K1 |
| MAPK1 |
| MAPK4 |
| MATN2 |
| MAX|HBA1 |
| MB |
| MDFIC |
| MDM2 |
| MEF2A |
| MEF2C |
| MET |
| METAP2 |
| MFAP2 |
| MFAP5 |
| MGA |
| MINPP1 |
| MLL2 |
| MLLT3 |
| MLLT4 |
| MME |
| MMP10 |
| MMP16 |
| MMP9 |
| MN1 |
| MNDA |
| MPDZ |
| MPP3 |
| MRC1|MRC1L1 |
| MRE11A |
| MRPS31 |
| MSH2 |
| MSH3 |
| MSX1 |
| MSX2 |
| MTM1 |
| MTMR3 |
| MUC5AC |
| MUT |
| MVK |
| MX1 |
| MX2 |
| MXD4 |
| MYC |
| MYCBP|PANX1 |
| MYF6 |
| MYH10|NDEL1 |
| MYL3 |
| MYLPF |
| MYO10|SLC23A2 |
| MYO9A |
| MYO9B |
| MYOG |
| N/A|ADAM10 |
| N/A|MEIS1 |
| N/A|N/A |
| N/A|PPARBP |
| N/A|PSG11 |
| N/A|SPRR2C |
| N/A|TRIP12 |
| NAIP |
| NAP1L3 |
| NBN |
| NCBP1 |
| NCL|N/A |
| NCOA2 |
| NDUFS6 |
| NEDD1 |
| NEDD4 |
| NEK1 |
| NEK2 |
| NEK3 |
| NF1|EVI2A |
| NF2 |
| NFIC |
| NFIX |
| NFKB2 |
| NID1 |
| NINJ1 |
| NIPBL |
| NKTR|N/A |
| NME1 |
| NOS2A |
| NOS3 |
| NPAS2|N/A |
| NPAT |
| NPY1R |
| NR1D2 |
| NR3C2 |
| NRAP |
| NRCAM |
| NT5E |
| NTRK3 |
| NUCB1 |
| NUDT1 |
| OCLN |
| OLIG2 |
| OLR1 |
| OPRK1 |
| ORC1L |
| OSMR |
| OTUD4 |
| OXTR|N/A |
| P2RY5 |
| PABPC1 |
| PAEP |
| PAX8 |
| PBEF1 |
| PBX1 |
| PCK1 |
| PCM1 |
| PCSK2 |
| PCTK3 |
| PDCD4 |
| PDE1B |
| PDE4A |
| PDE4DIP |
| PDE9A |
| PDGFRA |
| PDGFRB |
| PECAM1 |
| PF4 |
| PFAAP5|N/A |
| PFKM |
| PGGT1B |
| PGR |
| PHC1 |
| PHF6|HSD17B8 |
| PHKG2 |
| PHLDA3 |
| PIGA|C6orf142 |
| PIK3R1 |
| PIK4CA |
| PKIA |
| PLAGL1 |
| PLCB2 |
| PLCB4 |
| PLEK |
| PLK1 |
| PLK2 |
| PLK3 |
| PLN |
| PLOD2 |
| PLOD3 |
| PLXNB3 |
| PLXNC1 |
| PML |
| PMM2 |
| PMP22 |
| PMPCB|ZRF1 |
| PMS1 |
| PNN |
| POLD3 |
| POLI |
| POPDC3 |
| POU6F1 |
| PPFIBP1 |
| PPM1D |
| PPOX |
| PPP1R10 |
| PPP1R12B |
| PPP2R5A |
| PQBP1 |
| PRKAB2 |
| PRKAR2B |
| PRKCQ |
| PRKD1 |
| PRKDC |
| PROS1 |
| PROSC |
| PRPF39 |
| PRPF6 |
| PRSS8 |
| PSAP |
| PSCDBP |
| PSG1 |
| PSG9 |
| PSIP1 |
| PTEN |
| PTGDS|HN1 |
| PTGIR |
| PTGS2 |
| PTH |
| PTHR1 |
| PTPN1 |
| PTPN13 |
| PTPN7 |
| PTPRC |
| PTPRCAP |
| PTPRD |
| PTPRN2 |
| PTPRT |
| PTPRZ1 |
| PTTG1 |
| PVALB |
| PYCR2 |
| RAB22A |
| RAB40B |
| RAB7L1 |
| RAD52 |
| RANBP6 |
| RAP1GAP |
| RAPGEF2 |
| RARA |
| RARB |
| RASA1 |
| RASAL2 |
| RBBP8 |
| RBL1 |
| RBP1 |
| RCP9 |
| RDX |
| REG1B |
| RELB |
| RELN |
| RER1|N/A |
| RERE |
| REST |
| RET |
| RETSAT |
| REV3L |
| RFC1 |
| RGN |
| RGPD2|N/A |
| RGS16 |
| RGS3 |
| RGS4 |
| RHOB |
| RIPK1 |
| RLF |
| RNF6 |
| ROBO1 |
| ROCK1 |
| ROCK2 |
| RPL27 |
| RPL28 |
| RPL3 |
| RPL39L |
| RPN1 |
| RPS6KA4 |
| RSC1A1 |
| RUTBC3 |
| RY1|ACADSB |
| RYR1 |
| SACS |
| SALL2 |
| SCAP |
| SCARB2 |
| SCG2 |
| SCGB2A2 |
| SCN8A|MALAT1 |
| SCUBE2 |
| SCYE1 |
| SDC2 |
| SDCCAG10 |
| SDS |
| SEC63 |
| SERF1A |
| SERPINA5 |
| SERPINE1|N/A |
| SERPING1 |
| SFRS2IP |
| SFXN4 |
| SGCD |
| SH3BGR |
| SHROOM2 |
| SIRT4 |
| SKIL |
| SLA |
| SLC12A2 |
| SLC15A1 |
| SLC17A2 |
| SLC19A1 |
| SLC1A3 |
| SLC22A1 |
| SLC22A4|TUBA3 |
| SLC26A2 |
| SLC2A5 |
| SLC4A4 |
| SLC7A1 |
| SLC7A2 |
| SLIT3 |
| SMAD5 |
| SMARCA2 |
| SMC3|DDAH1 |
| SMC4 |
| SMG1 |
| SNRPN |
| SOD2 |
| SOS1 |
| SP1 |
| SP3 |
| SPAG5 |
| SPAG8 |
| SPIN |
| SPINK1 |
| SPP1 |
| SPTA1 |
| SSBP2 |
| SSNA1 |
| ST14|CPSF2 |
| ST8SIA2 |
| STAG1 |
| STAMBP |
| STAR |
| STK17B |
| STX6 |
| SUHW2 |
| SUOX |
| SUPT6H |
| SVIL |
| SYNJ1 |
| TACC3 |
| TAF1 |
| TAF2 |
| TAF4|N/A |
| TAS2R14 |
| TBXA2R |
| TCERG1 |
| TCL6 |
| TEC |
| TEKT2 |
| TERF1 |
| TFDP2 |
| TGFA |
| TGFBR3 |
| TGM2 |
| TGM2|STIL |
| TH |
| THBD |
| THBS1 |
| THBS2 |
| THRAP1 |
| THRAP3 |
| TIE1 |
| TIMELESS |
| TIMP3 |
| TJP1 |
| TLE4 |
| TLK2 |
| TLN1 |
| TLR2 |
| TM4SF5 |
| TMED10 |
| TMEFF1 |
| TMF1 |
| TMOD3|GYPC |
| TncRNA |
| TNFAIP3 |
| TNFRSF10B |
| TNFRSF10C |
| TNFRSF11B |
| TNFSF13B |
| TNPO1 |
| TOB2 |
| TOM1L1|COX11 |
| TOPBP1 |
| TP53 |
| TPMT |
| TPR |
| TRAF1 |
| TRAF5 |
| TRIB1 |
| TRIM22 |
| TRIM24 |
| TRIM27|DNAJC10 |
| TRIO |
| TRIP11 |
| TROVE2 |
| TSC2 |
| TSN |
| TSPAN13 |
| TSPAN7 |
| TSPAN8 |
| TTK |
| TULP2 |
| TUSC2 |
| TYR |
| TYRP1 |
| UBE3A |
| USP1 |
| USP12 |
| USP16 |
| USP32 |
| USP8 |
| USP9Y|N/A |
| UTX |
| VASP |
| VCAM1 |
| VDP |
| VEGFA |
| VIPR1 |
| VSNL1 |
| WAS |
| WEE1 |
| WNK2 |
| WT1 |
| XK |
| XPA |
| XPC |
| XRCC1 |
| XRCC4 |
| ZBTB25 |
| ZBTB26 |
| ZBTB33 |
| ZFX |
| ZMYM6 |
| ZNF124 |
| ZNF146 |
| ZNF175 |
| ZNF184 |
| ZNF273 |
| ZNF345 |
| ZNF35 |
| ZNF43 |
| ZNF559 |
| ZNF597|VCY1B |
| ZPBP |
| ZYG11BL |

| **Supplementary** **Table 5.** Mass spectrographic analysis of CLPTM1L | | | | |  |
| --- | --- | --- | --- | --- | --- |
| Gene name | Mass | Score | Matches | Sequences | emPAI |
| MYH9 | 227646 | 5189 | 212(152) | 107(82) | 5.26 |
| PS1TP5BP1 | 42052 | 4747 | 234(166) | 25(23) | 42.88 |
| ACTN4 | 105245 | 4230 | 172(134) | 62(49) | 11.32 |
| ESR2 | 55483 | 4117 | 219(149) | 103(77) | 2.36 |
| HEL-S-30 | 58470 | 3637 | 172(114) | 39(34) | 30.41 |
| HSP90AB1 | 83554 | 3405 | 183(106) | 53(36) | 9.86 |
| TUBB | 50095 | 3216 | 141(112) | 29(26) | 24.85 |
| ENO1 | 47481 | 3206 | 127(89) | 32(25) | 15.77 |
| ANXA2 | 38808 | 3049 | 125(99) | 32(28) | 49.61 |
| N/A | 46725 | 2997 | 115(92) | 23(21) | 15.40 |
| TUBA1A | 50788 | 2887 | 110(87) | 22(20) | 10.59 |
| HSPD1 | 61187 | 2842 | 103(77) | 39(30) | 12.01 |
| RXRα | 47905 | 2798 | 150(99) | 36(31) | 11.55 |
| HEL-S-53e | 55454 | 2762 | 155(112) | 37(33) | 20.20 |
| TUBB2A | 50274 | 2686 | 122(91) | 27(21) | 16.41 |
| TUBB4A | 50010 | 2613 | 121(94) | 23(22) | 12.66 |
| TUBB3 | 50856 | 2395 | 101(80) | 24(22) | 12.99 |
| TUBA4A | 50634 | 2266 | 97(74) | 22(20) | 9.99 |
| CLTC | 193703 | 1982 | 98(69) | 60(43) | 1.72 |
| EEF2 | 96246 | 1879 | 98(73) | 41(33) | 4.51 |
| HEL-S-162eP | 36201 | 1861 | 86(55) | 21(17) | 24.59 |
| ANXA1 | 38918 | 1834 | 59(48) | 24(20) | 13.74 |
| FLNB | 280157 | 1814 | 132(70) | 82(50) | 1.00 |
| KRT8 | 53671 | 1756 | 89(56) | 36(28) | 10.47 |
| HEL107 | 68519 | 1679 | 92(60) | 32(24) | 5.82 |
| KRT18 | 48029 | 1654 | 84(53) | 29(24) | 12.31 |
| G6PD | 59675 | 1635 | 91(65) | 35(29) | 9.58 |
| SPTAN1 | 282874 | 1416 | 84(50) | 66(41) | 0.79 |
| LMNA | 74380 | 1363 | 76(49) | 40(29) | 4.39 |
| AKR1B10 | 36225 | 1259 | 68(43) | 22(17) | 8.76 |
| EEF1A1 | 50433 | 1258 | 79(52) | 20(15) | 3.03 |
| RXRβ | 38516 | 1238 | 59(42) | 24(17) | 6.37 |
| KRT7 | 51411 | 1222 | 65(44) | 32(27) | 6.77 |
| LMNA | 65135 | 1219 | 69(44) | 37(26) | 4.33 |
| HEL-S-133P | 36950 | 1190 | 69(42) | 25(20) | 13.30 |
| ATP5B | 48083 | 1159 | 53(40) | 21(19) | 5.41 |
| HEL-S-123m | 59828 | 1142 | 51(38) | 23(20) | 2.81 |
| KRT1 | 66197 | 1130 | 48(35) | 28(22) | 2.53 |
| HSPA1A | 70294 | 1128 | 57(41) | 26(21) | 2.76 |
| PPARα | 52225 | 1122 | 55(34) | 27(19) | 1.94 |
| GPI | 65012 | 1075 | 60(42) | 22(21) | 4.90 |
| HEL-S-49 | 26938 | 1061 | 41(32) | 17(15) | 11.99 |
| HEL-S-87p | 39851 | 1049 | 55(37) | 20(16) | 5.26 |
| HEL70 | 67892 | 1042 | 75(39) | 35(22) | 4.74 |
| ACLY variant protein | 125389 | 1033 | 62(37) | 37(25) | 1.34 |
| FASN | 275649 | 1022 | 61(41) | 46(34) | 0.60 |
| TLN1 | 273475 | 1017 | 47(30) | 40(26) | 0.39 |
| HEL-S-68p | 44985 | 1013 | 64(38) | 29(18) | 5.31 |
| UGDH | 55674 | 1007 | 47(29) | 23(16) | 2.54 |
| HEL-S-70 | 89950 | 1003 | 61(36) | 31(21) | 1.82 |
| SPTBN1 | 275237 | 999 | 80(44) | 63(40) | 0.68 |
| NCL | 76625 | 981 | 29(20) | 16(12) | 0.88 |
| PGD | 53619 | 950 | 48(36) | 24(20) | 4.00 |
| HEL-S-100n | 57794 | 927 | 43(34) | 19(16) | 3.71 |
| HNRPA1 | 38837 | 912 | 52(34) | 21(14) | 5.56 |
| TXNRD1 | 69139 | 903 | 32(23) | 18(14) | 1.78 |
| VCL | 117220 | 902 | 42(28) | 29(20) | 0.94 |
| AHNAK | 629213 | 887 | 125(51) | 96(44) | 0.29 |
| N/A | 72641 | 861 | 38(26) | 20(15) | 1.12 |
| PLEC | 533462 | 832 | 99(38) | 83(34) | 0.28 |
| MDH2 | 35937 | 819 | 28(23) | 13(12) | 2.75 |
| YWHAZ | 27899 | 814 | 29(21) | 13(9) | 3.86 |
| HNRPK | 51472 | 797 | 33(27) | 19(15) | 2.68 |
| XRCC6 | 70084 | 776 | 41(29) | 24(19) | 2.00 |
| PLS3 | 71279 | 753 | 37(23) | 21(13) | 1.25 |
| N/A | 51577 | 743 | 36(20) | 21(12) | 2.45 |
| YWHAQ | 28032 | 741 | 25(24) | 11(11) | 4.37 |
| ERP70 | 73229 | 734 | 40(21) | 27(13) | 1.02 |
| hCG_1991735 | 189761 | 731 | 51(27) | 41(23) | 0.61 |
| P4HB | 57480 | 725 | 55(32) | 25(21) | 3.75 |
| UBE1 | 118858 | 721 | 41(20) | 22(14) | 0.77 |
| HEL-S-22 | 23569 | 700 | 24(22) | 9(8) | 3.93 |
| KRT10 | 59020 | 678 | 35(24) | 24(17) | 1.80 |
| N/A | 60183 | 676 | 33(22) | 22(16) | 1.34 |
| HEL-S-45 | 78420 | 672 | 33(18) | 23(11) | 0.85 |
| PRDX1 | 22324 | 667 | 58(37) | 16(14) | 15.43 |
| TPM3 | 29243 | 630 | 39(23) | 19(12) | 8.66 |
| EEF1A2 | 50780 | 623 | 43(28) | 11(9) | 1.27 |
| NQO1 | 22836 | 622 | 39(28) | 14(11) | 9.22 |
| PTBP1 | 62653 | 604 | 25(17) | 14(12) | 1.27 |
| HSPA4 | 95127 | 602 | 34(19) | 25(16) | 0.78 |
| HEL-S-7 | 35971 | 599 | 33(25) | 18(16) | 4.83 |
| HNRNPA2B1 | 37464 | 598 | 43(31) | 19(16) | 4.91 |
| N/A | 58479 | 597 | 36(21) | 24(14) | 1.54 |
| DHX9 | 142181 | 592 | 46(25) | 28(18) | 0.72 |
| NPM1 | 32726 | 592 | 27(21) | 10(8) | 3.26 |
| TUFM | 49852 | 590 | 36(21) | 20(12) | 1.45 |
| RPSA | 32947 | 589 | 25(13) | 13(8) | 1.61 |
| N/A | 49307 | 581 | 35(24) | 14(14) | 2.21 |
| PABPC1 | 70854 | 581 | 31(25) | 21(17) | 1.48 |
| CKAP4 | 66097 | 581 | 29(16) | 22(11) | 1.17 |
| SLC3A2 | 68230 | 579 | 45(24) | 25(18) | 1.81 |
| N/A | 98434 | 575 | 35(21) | 22(15) | 1.05 |
| AKR1C3 | 37243 | 570 | 41(28) | 16(11) | 2.90 |
| GSTP1 | 16771 | 558 | 17(16) | 5(5) | 4.23 |
| CANX | 67982 | 553 | 31(18) | 14(10) | 0.68 |
| EEF1G | 50429 | 543 | 39(24) | 20(15) | 2.78 |
| EEF1A | 24352 | 534 | 28(21) | 4(4) | 1.80 |
| TAGLN2 | 21244 | 532 | 24(16) | 13(9) | 6.83 |
| NAP1L1 | 44884 | 529 | 22(14) | 10(8) | 1.03 |
| AKR1C1 | 37221 | 527 | 43(26) | 15(11) | 2.92 |
| KYNU | 52831 | 519 | 30(23) | 15(13) | 2.35 |
| SFPQ | 76216 | 501 | 26(18) | 16(13) | 0.96 |
| KRT9 | 62255 | 501 | 17(12) | 12(8) | 0.85 |
| N/A | 52811 | 491 | 31(15) | 18(12) | 1.33 |
| AHCY | 48255 | 491 | 35(17) | 18(10) | 1.36 |
| N/A | 30868 | 491 | 19(16) | 11(9) | 1.51 |
| ALDH3A1 | 57987 | 486 | 27(18) | 15(12) | 1.29 |
| HEL-S-99n | 48283 | 482 | 24(13) | 12(8) | 0.94 |
| PYGB | 97319 | 480 | 21(15) | 18(13) | 0.64 |
| DDX5 | 69557 | 476 | 37(19) | 23(11) | 1.19 |
| STAT1 | 87850 | 476 | 27(19) | 20(16) | 0.86 |
| MYH10 | 229827 | 475 | 42(15) | 29(9) | 0.27 |
| N/A | 64939 | 471 | 19(11) | 15(9) | 0.72 |
| KPNA2 | 58182 | 467 | 17(14) | 13(11) | 0.83 |
| PHB2 | 33219 | 462 | 26(15) | 13(8) | 1.36 |
| PHB | 29871 | 462 | 23(16) | 11(8) | 1.88 |
| CSE1L | 111145 | 452 | 29(14) | 21(12) | 0.50 |
| HEL-S-128m | 25133 | 444 | 19(11) | 10(7) | 2.49 |
| ARHGDIA | 21561 | 435 | 17(16) | 6(6) | 2.68 |
| N/A | 27119 | 432 | 18(13) | 11(9) | 2.59 |
| ANXA4 | 36290 | 430 | 17(10) | 14(9) | 1.40 |
| PGAM1 | 28900 | 425 | 26(19) | 13(13) | 6.14 |
| TPM2 | 32909 | 421 | 30(16) | 16(10) | 3.67 |
| HNRNPU | 81283 | 419 | 29(18) | 17(10) | 0.61 |
| RPL7 | 30476 | 413 | 31(18) | 17(11) | 3.25 |
| N/A | 113539 | 411 | 38(18) | 26(16) | 0.87 |
| RPL7A | 30148 | 407 | 25(15) | 14(8) | 2.50 |
| AKR1C2 | 37111 | 406 | 33(19) | 13(9) | 2.31 |
| N/A | 75619 | 405 | 17(11) | 12(9) | 0.47 |
| PCBP1 | 37987 | 400 | 16(14) | 10(9) | 1.97 |
| RAB1A | 22891 | 398 | 14(10) | 5(5) | 1.27 |
| N/A | 59816 | 391 | 25(15) | 18(13) | 1.12 |
| HSPH1 | 97716 | 390 | 31(17) | 22(12) | 0.54 |
| HEL-S-34 | 21158 | 388 | 12(10) | 7(6) | 1.80 |
| TCP1 | 60819 | 385 | 22(11) | 16(8) | 0.98 |
| TPM1 | 32856 | 379 | 27(15) | 16(10) | 3.67 |
| MYOF | 236100 | 378 | 37(18) | 32(16) | 0.26 |
| IMPDH2 | 51549 | 378 | 15(11) | 9(7) | 0.75 |
| N/A | 35511 | 377 | 30(18) | 17(9) | 1.92 |
| RPL6 | 32765 | 376 | 23(13) | 13(9) | 2.87 |
| PRKDC | 473749 | 375 | 75(22) | 59(20) | 0.18 |
| N/A | 27211 | 372 | 20(16) | 11(9) | 3.50 |
| N/A | 54468 | 371 | 31(16) | 21(11) | 1.27 |
| N/A | 52814 | 367 | 12(7) | 10(7) | 0.53 |
| PCNA | 29092 | 365 | 17(12) | 13(10) | 1.96 |
| HNRNPC | 31986 | 365 | 15(10) | 10(7) | 1.69 |
| N/A | 83688 | 363 | 21(12) | 17(10) | 0.53 |
| RPS3 | 26842 | 359 | 32(17) | 15(10) | 2.62 |
| LRPPRC | 159003 | 358 | 44(20) | 31(16) | 0.47 |
| CBR1 | 30641 | 357 | 19(13) | 10(7) | 1.53 |
| HMGB1 | 25049 | 354 | 22(12) | 9(6) | 2.49 |
| PRIC295 | 294953 | 353 | 28(17) | 26(16) | 0.19 |
| MYBBP1A | 149731 | 351 | 18(12) | 16(12) | 0.30 |
| HNRNPH1 | 51482 | 351 | 16(14) | 9(8) | 0.86 |
| eIF3a | 166780 | 348 | 36(14) | 27(13) | 0.31 |
| GOT2 | 47886 | 347 | 19(12) | 13(7) | 0.82 |
| N/A | 59138 | 347 | 30(16) | 17(12) | 1.51 |
| SYNCRIP | 50847 | 347 | 18(13) | 11(9) | 0.87 |
| CCT6A | 58444 | 345 | 23(14) | 16(11) | 1.04 |
| HNRNPM | 77746 | 344 | 21(14) | 14(10) | 0.86 |
| N/A | 31760 | 342 | 10(10) | 6(6) | 1.22 |
| GAPD | 9252 | 341 | 20(12) | 8(5) | 12.04 |
| HNRNPL | 64720 | 339 | 19(12) | 9(7) | 0.64 |
| ITGB1 | 91664 | 338 | 22(11) | 12(7) | 0.42 |
| N/A | 63243 | 335 | 36(15) | 21(12) | 1.37 |
| N/A | 49353 | 334 | 22(12) | 17(9) | 0.91 |
| AP2B1 | 101953 | 323 | 18(10) | 15(9) | 0.37 |
| DRIP4 | 96646 | 316 | 26(12) | 21(10) | 0.40 |
| AARS | 107484 | 315 | 25(12) | 20(12) | 0.43 |
| N/A | 101322 | 315 | 20(12) | 16(9) | 0.46 |
| SFN | 27871 | 312 | 16(14) | 9(8) | 2.09 |
| hCG_2016482 | 23370 | 310 | 12(8) | 6(4) | 0.95 |
| XPO1 | 124447 | 308 | 22(9) | 20(9) | 0.33 |
| N/A | 69405 | 307 | 15(10) | 11(7) | 0.52 |
| RAN | 27027 | 304 | 20(10) | 12(5) | 1.01 |
| N/A | 21880 | 304 | 15(13) | 7(7) | 2.13 |
| N/A | 90271 | 300 | 14(8) | 10(6) | 0.28 |
| IGF2BP1 | 48111 | 298 | 13(6) | 11(5) | 0.39 |
| N/A | 136576 | 296 | 18(10) | 15(9) | 0.24 |
| HEL-S-10 | 22219 | 296 | 7(7) | 4(4) | 1.02 |
| PAICS | 47790 | 294 | 20(12) | 16(10) | 1.08 |
| GARS | 78166 | 293 | 19(8) | 16(7) | 0.57 |
| HEL-S-102 | 22826 | 292 | 17(11) | 10(7) | 2.42 |
| SLC25A6 | 33073 | 291 | 23(14) | 13(9) | 1.86 |
| CD44 | 23068 | 287 | 9(7) | 4(3) | 0.97 |
| SLC7A5 | 55659 | 285 | 10(9) | 5(5) | 0.41 |
| N/A | 47089 | 285 | 12(7) | 8(6) | 0.72 |
| XRCC5 | 83222 | 284 | 37(13) | 23(10) | 0.65 |
| HNRNPA3 | 39799 | 283 | 27(14) | 14(8) | 1.05 |
| RPLP0 | 34423 | 278 | 22(8) | 13(6) | 1.09 |
| hCG_2005638 | 49416 | 277 | 21(11) | 11(8) | 0.79 |
| PARP1 | 113811 | 277 | 18(10) | 14(7) | 0.29 |
| EIF3S8 | 105962 | 275 | 16(11) | 12(10) | 0.40 |
| KRT2 | 65678 | 275 | 17(11) | 12(8) | 0.55 |
| RARα | 50771 | 274 | 14(10) | 10(8) | 0.44 |
| RARS | 76129 | 274 | 16(9) | 15(9) | 0.46 |
| LMNB1 | 66653 | 273 | 15(9) | 14(8) | 0.70 |
| EIF3L | 71085 | 271 | 12(8) | 12(8) | 0.44 |
| RPN1 | 68678 | 270 | 20(12) | 15(11) | 0.75 |
| PCBP2 | 38955 | 269 | 15(9) | 10(7) | 1.26 |
| MYO1C | 119719 | 269 | 13(11) | 10(10) | 0.31 |
| PFAS | 146286 | 268 | 18(11) | 14(7) | 0.25 |
| EIF6 | 27095 | 268 | 9(8) | 6(5) | 1.01 |
| ILF2 | 39057 | 267 | 9(6) | 6(5) | 0.50 |
| N/A | 71830 | 266 | 10(6) | 6(3) | 0.20 |
| N/A | 37688 | 266 | 20(10) | 14(8) | 1.74 |
| N/A | 29242 | 266 | 8(6) | 5(4) | 0.72 |
| SND1 | 102618 | 264 | 26(14) | 20(12) | 0.50 |
| SPR | 28316 | 264 | 8(6) | 5(4) | 0.74 |
| N/A | 51868 | 264 | 19(10) | 13(7) | 0.85 |
| PYGL | 97486 | 262 | 22(10) | 18(9) | 0.35 |
| MTHFD1 | 102152 | 262 | 18(11) | 14(10) | 0.46 |
| N/A | 82974 | 261 | 17(9) | 15(8) | 0.47 |
| DKFZp686L1159 | 52189 | 261 | 16(7) | 11(6) | 0.63 |
| RPS3A | 30184 | 259 | 26(11) | 15(8) | 2.16 |
| PDIA6 | 48490 | 258 | 17(10) | 11(8) | 1.06 |
| N/A | 137941 | 257 | 18(12) | 16(12) | 0.32 |
| DDX21 | 87804 | 253 | 16(8) | 14(6) | 0.34 |
| YBX1 | 35903 | 252 | 13(11) | 8(7) | 1.22 |
| RPS4X | 29807 | 251 | 32(18) | 17(11) | 3.88 |
| RUVBL2 | 51296 | 249 | 19(11) | 12(9) | 1.11 |
| HEL-S-276 | 28041 | 249 | 19(11) | 10(7) | 2.07 |
| UCHL1 | 27108 | 249 | 25(12) | 9(6) | 1.53 |
| RPL18 | 21735 | 247 | 15(10) | 10(8) | 2.63 |
| SEPT2 | 41689 | 247 | 12(9) | 9(7) | 0.84 |
| PSME3 | 30984 | 247 | 7(6) | 6(5) | 0.84 |
| RPS6 | 28834 | 244 | 11(8) | 7(5) | 1.15 |
| MCM6 | 89803 | 243 | 14(9) | 12(9) | 0.43 |
| PSMA2 | 25996 | 242 | 9(7) | 7(6) | 1.33 |
| N/A | 28826 | 241 | 14(10) | 10(6) | 1.15 |
| UQCRC1 | 53297 | 241 | 11(6) | 9(5) | 0.43 |
| ETFA | 35400 | 241 | 12(8) | 9(7) | 1.05 |
| N/A | 41736 | 239 | 14(6) | 9(6) | 0.58 |
| ECH1 | 36136 | 238 | 11(8) | 7(6) | 0.85 |
| N/A | 69868 | 238 | 16(11) | 12(9) | 0.51 |
| N/A | 85276 | 238 | 19(9) | 15(7) | 0.35 |
| RPS7 | 22113 | 237 | 18(11) | 13(8) | 3.73 |
| ASPH | 86266 | 237 | 16(7) | 13(6) | 0.30 |
| RPS8 | 24475 | 237 | 17(11) | 9(6) | 1.79 |
| N/A | 32758 | 234 | 13(9) | 8(7) | 1.39 |
| ATP2A2 | 116336 | 233 | 21(9) | 15(8) | 0.32 |
| TOMM40 | 38211 | 232 | 8(6) | 6(5) | 0.65 |
| RPL9 | 20819 | 231 | 13(8) | 8(5) | 1.11 |
| DPYSL2 | 74027 | 229 | 22(8) | 15(7) | 0.41 |
| SLC4A1 | 102150 | 228 | 4(2) | 3(1) | 0.07 |
| DLST | 49067 | 227 | 9(7) | 7(6) | 0.48 |
| N/A | 27838 | 227 | 9(7) | 7(5) | 1.47 |
| hCG_2001986 | 50214 | 226 | 13(8) | 11(7) | 0.56 |
| IARS | 132820 | 226 | 15(10) | 14(10) | 0.31 |
| EPRS | 172080 | 226 | 26(8) | 22(8) | 0.21 |
| RPL15 | 24245 | 225 | 14(9) | 9(7) | 1.82 |
| EIF4G1 | 176807 | 225 | 19(5) | 18(5) | 0.14 |
| UQCRC2 | 44778 | 224 | 11(7) | 7(6) | 0.90 |
| FAM62A | 124439 | 223 | 10(6) | 8(5) | 0.17 |
| CS | 51908 | 222 | 13(10) | 9(8) | 0.74 |
| KRT19 | 44079 | 221 | 15(8) | 10(6) | 0.54 |
| CTPS1 | 67332 | 216 | 12(7) | 11(6) | 0.40 |
| RPL13 | 24308 | 215 | 15(10) | 7(5) | 0.90 |
| RAB5C | 23696 | 213 | 7(6) | 6(5) | 0.94 |
| HEL-S-32 | 36631 | 212 | 15(10) | 8(6) | 1.00 |
| SSB | 47009 | 211 | 17(8) | 12(7) | 0.84 |
| RPL14 | 23886 | 211 | 12(7) | 6(3) | 0.69 |
| GART | 108909 | 210 | 13(8) | 11(6) | 0.31 |
| ETFB | 28054 | 210 | 15(10) | 10(8) | 2.07 |
| IPO5 | 125319 | 209 | 17(9) | 13(7) | 0.23 |
| RAB10 | 22755 | 209 | 9(7) | 6(4) | 0.99 |
| PSME2 | 29279 | 208 | 11(6) | 9(5) | 0.91 |
| KHSRP | 73438 | 207 | 11(6) | 8(4) | 0.19 |
| N/A | 47993 | 206 | 9(5) | 8(4) | 0.30 |
| N/A | 26700 | 206 | 9(6) | 7(4) | 0.80 |
| PSMD3 | 61022 | 203 | 13(9) | 11(9) | 0.60 |
| RPS27A | 18296 | 203 | 19(12) | 6(4) | 2.27 |
| IDH1 | 42091 | 202 | 15(7) | 12(6) | 0.83 |
| TMED10 | 25131 | 200 | 7(6) | 4(4) | 0.87 |
| RUVBL1 | 50526 | 199 | 21(10) | 14(9) | 0.77 |
| N/A | 49002 | 199 | 15(5) | 12(5) | 0.68 |
| RNH1 | 51209 | 197 | 7(6) | 5(4) | 0.28 |
| N/A | 24792 | 197 | 14(8) | 8(6) | 1.14 |
| PON2 | 41672 | 196 | 6(4) | 4(3) | 0.26 |
| N/A | 77408 | 196 | 17(9) | 14(9) | 0.58 |
| N/A | 71705 | 195 | 22(10) | 15(8) | 0.50 |
| PDXK | 30790 | 193 | 5(4) | 4(3) | 0.51 |
| HNRNPA0 | 30993 | 192 | 10(10) | 4(4) | 0.66 |
| PSMD13 | 43176 | 192 | 11(6) | 10(6) | 0.56 |
| EIF3S9 | 92823 | 191 | 15(9) | 13(7) | 0.32 |
| N/A | 41544 | 191 | 8(5) | 7(4) | 0.47 |
| DKFZp686O2462 | 72627 | 191 | 11(9) | 9(7) | 0.42 |
| RNPC2 | 40744 | 191 | 6(5) | 6(5) | 0.48 |
| N/A | 37046 | 190 | 6(5) | 4(4) | 0.53 |
| MATR3 | 95078 | 189 | 17(5) | 12(4) | 0.27 |
| N/A | 26565 | 189 | 12(7) | 9(6) | 1.29 |
| SET | 32084 | 188 | 12(5) | 7(4) | 0.64 |
| PRPF8 | 274738 | 188 | 27(10) | 23(8) | 0.15 |
| CTNNA1 | 93576 | 188 | 11(5) | 11(5) | 0.23 |
| N/A | 56588 | 186 | 12(6) | 11(6) | 0.40 |
| ILF3 | 95678 | 185 | 19(10) | 15(9) | 0.40 |
| RPL10 | 23416 | 184 | 15(12) | 7(5) | 1.55 |
| N/A | 20884 | 183 | 7(6) | 4(4) | 1.11 |
| N/A | 56462 | 183 | 5(4) | 5(4) | 0.25 |
| SNRNP200 | 246006 | 183 | 13(4) | 12(4) | 0.07 |
| RPL8 | 28235 | 182 | 13(7) | 6(4) | 1.19 |
| WARS | 53474 | 180 | 6(4) | 5(4) | 0.27 |
| ELAVL1 | 36240 | 179 | 9(5) | 7(4) | 0.55 |
| PA2G4 | 44101 | 178 | 23(8) | 13(7) | 0.78 |
| NARS | 63758 | 177 | 14(9) | 13(9) | 0.57 |
| DNM1L | 88442 | 177 | 7(4) | 7(4) | 0.20 |
| DLD | 54713 | 176 | 13(6) | 8(3) | 0.26 |
| HNRPD | 36420 | 176 | 12(6) | 9(5) | 0.55 |
| STRAP | 38756 | 176 | 7(5) | 6(5) | 0.51 |
| EIF3F | 39236 | 175 | 8(8) | 6(6) | 0.63 |
| RPL5 | 34569 | 175 | 14(9) | 9(7) | 1.28 |
| RPS2 | 31590 | 174 | 15(10) | 9(7) | 1.01 |
| EPHX1 | 53143 | 174 | 11(6) | 10(5) | 0.43 |
| EIF2AK2 | 61928 | 174 | 9(7) | 8(6) | 0.44 |
| TPT1 | 22787 | 173 | 13(8) | 4(4) | 1.28 |
| VDAC2 | 35029 | 173 | 9(8) | 6(5) | 0.72 |
| PSMD1 | 106810 | 172 | 8(4) | 7(4) | 0.13 |
| ANK1 | 207334 | 172 | 16(7) | 5(2) | 0.03 |
| AKAP12 | 191937 | 172 | 11(5) | 11(5) | 0.09 |
| N/A | 90289 | 172 | 16(8) | 14(8) | 0.33 |
| N/A | 88665 | 170 | 14(6) | 13(6) | 0.29 |
| COPG1 | 98967 | 170 | 12(6) | 11(6) | 0.26 |
| KTN1 | 156464 | 170 | 21(7) | 19(7) | 0.16 |
| IPO4 | 120179 | 170 | 7(5) | 7(5) | 0.14 |
| N/A | 35615 | 170 | 8(4) | 5(3) | 0.31 |
| TPD52L2 | 23864 | 168 | 5(3) | 4(3) | 0.48 |
| N/A | 28066 | 168 | 9(5) | 6(3) | 0.40 |
| Nbla02942 | 49028 | 167 | 9(6) | 6(5) | 0.38 |
| N/A | 40234 | 167 | 15(7) | 10(6) | 0.61 |
| BAX | 21285 | 167 | 6(6) | 4(4) | 0.80 |
| RBBP7 | 48132 | 165 | 10(4) | 8(4) | 0.39 |
| N/A | 56910 | 164 | 15(7) | 13(6) | 0.48 |
| MSH2 | 103977 | 163 | 10(4) | 9(4) | 0.13 |
| EEF1B2 | 24919 | 163 | 7(5) | 4(3) | 0.46 |
| FBL | 26737 | 162 | 9(7) | 7(5) | 1.28 |
| LRRC59 | 35308 | 162 | 13(8) | 8(4) | 0.71 |
| CAD | 238097 | 162 | 12(5) | 10(4) | 0.09 |
| ANXA11 | 54697 | 162 | 7(3) | 6(3) | 0.19 |
| PGRMC1 | 21772 | 161 | 8(4) | 7(3) | 0.77 |
| DYNC1H1 | 534809 | 160 | 36(7) | 33(7) | 0.06 |
| DDX39A | 49611 | 160 | 18(7) | 10(6) | 0.57 |
| CORO1C | 53899 | 160 | 13(7) | 8(6) | 0.51 |
| STOML2 | 38624 | 160 | 9(5) | 8(5) | 0.51 |
| N/A | 17942 | 159 | 9(5) | 7(4) | 1.36 |
| N/A | 135550 | 159 | 8(4) | 8(4) | 0.10 |
| HEL-S-52 | 66836 | 159 | 18(8) | 13(6) | 0.54 |
| N/A | 81716 | 159 | 12(6) | 9(6) | 0.37 |
| N/A | 57499 | 158 | 17(7) | 13(6) | 0.40 |
| MAP4 | 246546 | 158 | 17(5) | 13(5) | 0.07 |
| TOP2A | 175017 | 158 | 17(8) | 15(7) | 0.14 |
| AP1B1 | 105482 | 156 | 12(5) | 9(4) | 0.17 |
| OLA1 | 47136 | 156 | 7(4) | 6(4) | 0.40 |
| PGRMC2 | 23861 | 155 | 8(3) | 5(2) | 0.48 |
| HSD17B10 | 27134 | 155 | 7(3) | 6(3) | 0.42 |
| N/A | 35355 | 155 | 10(5) | 5(2) | 0.43 |
| SFXN1 | 35881 | 155 | 6(4) | 6(4) | 0.56 |
| N/A | 34726 | 154 | 7(5) | 5(5) | 0.58 |
| FAM49B | 37010 | 154 | 7(4) | 6(4) | 0.41 |
| RPS9 | 22635 | 153 | 15(10) | 12(8) | 2.02 |
| HEL60 | 98850 | 152 | 11(7) | 10(7) | 0.26 |
| IPO7 | 120751 | 151 | 14(6) | 10(4) | 0.24 |
| N/A | 113791 | 150 | 12(5) | 10(4) | 0.15 |
| CLPTM1L | 62531 | 150 | 6(3) | 5(3) | 0.17 |
| VDAC3 | 30981 | 150 | 5(5) | 2(2) | 0.23 |
| AK2 | 26689 | 148 | 10(4) | 7(3) | 0.42 |
| N/A | 137715 | 148 | 12(5) | 10(4) | 0.18 |
| SSRP1 | 81367 | 147 | 17(8) | 14(6) | 0.27 |
| ALDH18A1 | 87989 | 147 | 8(5) | 8(5) | 0.25 |
| HDGF | 26886 | 147 | 11(4) | 8(4) | 0.60 |
| SERPINH1 | 46525 | 146 | 19(6) | 14(6) | 0.62 |
| PSMD11 | 47719 | 146 | 20(6) | 14(5) | 0.60 |
| ACTR3 | 47797 | 146 | 11(7) | 8(6) | 0.49 |
| FKBP4 | 52057 | 145 | 26(8) | 21(7) | 0.85 |
| DNAJA1 | 45581 | 143 | 15(7) | 8(5) | 0.52 |
| SEPT9 | 47724 | 143 | 12(6) | 9(6) | 0.49 |
| ADSS | 50465 | 143 | 4(3) | 4(3) | 0.21 |
| N/A | 51334 | 143 | 8(6) | 6(5) | 0.45 |
| RAB2 | 23702 | 142 | 4(4) | 3(3) | 0.69 |
| N/A | 25245 | 142 | 10(6) | 6(4) | 0.86 |
| CAPNS1 | 22069 | 141 | 8(5) | 6(3) | 1.03 |
| MCM7 | 81884 | 141 | 8(4) | 7(4) | 0.17 |
| PSMD12 | 53270 | 139 | 11(5) | 10(4) | 0.43 |
| N/A | 96638 | 139 | 11(5) | 11(5) | 0.18 |
| N/A | 93434 | 139 | 23(6) | 18(6) | 0.27 |
| AIFM1 | 67144 | 138 | 7(5) | 6(4) | 0.21 |
| COPB1 | 108214 | 137 | 10(6) | 9(6) | 0.23 |
| N/A | 53582 | 136 | 13(5) | 10(5) | 0.35 |
| EHD1 | 61945 | 136 | 11(4) | 10(3) | 0.23 |
| HEL-S-66p | 33095 | 135 | 9(5) | 7(4) | 0.61 |
| EIF2S1 | 36374 | 135 | 10(4) | 9(3) | 0.42 |
| N/A | 35101 | 134 | 9(5) | 8(5) | 0.57 |
| NOP58 | 60054 | 134 | 8(4) | 8(4) | 0.31 |
| N/A | 96189 | 134 | 14(5) | 12(5) | 0.18 |
| PBEF1 | 55772 | 132 | 25(9) | 13(6) | 0.68 |
| TNFAIP2 | 73015 | 132 | 9(2) | 8(2) | 0.09 |
| HEL-75 | 56791 | 132 | 12(7) | 9(5) | 0.40 |
| TIMM50 | 50946 | 131 | 4(3) | 4(3) | 0.28 |
| N/A | 41477 | 131 | 7(7) | 4(4) | 0.36 |
| PUF60 | 57533 | 131 | 10(8) | 7(5) | 0.32 |
| BZW1 | 48184 | 131 | 17(7) | 12(4) | 0.59 |
| ALB | 71177 | 130 | 7(4) | 4(1) | 0.14 |
| RBMX | 31837 | 130 | 9(5) | 6(3) | 0.49 |
| N/A | 27949 | 130 | 10(6) | 4(4) | 0.57 |
| SDHA | 73672 | 129 | 7(2) | 7(2) | 0.09 |
| THRAP3 | 108658 | 128 | 6(5) | 4(4) | 0.16 |
| N/A | 45456 | 128 | 6(3) | 6(3) | 0.23 |
| TNPO1 | 103771 | 128 | 13(5) | 11(4) | 0.20 |
| N/A | 30843 | 128 | 8(2) | 6(1) | 0.23 |
| N/A | 24906 | 127 | 8(4) | 6(4) | 0.66 |
| TJP1 | 188076 | 126 | 13(5) | 11(5) | 0.11 |
| STAT3 | 88742 | 126 | 9(4) | 8(3) | 0.16 |
| N/A | 28643 | 126 | 7(5) | 6(4) | 0.73 |
| RRBP1 | 152764 | 124 | 12(3) | 11(3) | 0.11 |
| PPP1CA | 29335 | 124 | 10(6) | 6(4) | 0.91 |
| TPR | 267530 | 123 | 14(5) | 12(5) | 0.06 |
| HEL-S-67p | 20050 | 123 | 17(9) | 10(6) | 2.45 |
| MVP | 99551 | 123 | 15(7) | 11(7) | 0.25 |
| NSUN2 | 87214 | 123 | 6(3) | 6(3) | 0.12 |
| N/A | 27528 | 122 | 6(2) | 5(2) | 0.41 |
| ALYREF | 27541 | 122 | 7(2) | 5(2) | 0.26 |
| TBCB | 27594 | 122 | 7(5) | 6(4) | 0.77 |
| PSMC6 | 46053 | 122 | 6(4) | 6(4) | 0.32 |
| AHSA1 | 38421 | 121 | 9(4) | 8(4) | 0.51 |
| NDUFS1 | 67563 | 121 | 6(4) | 6(4) | 0.21 |
| RPL29 | 17599 | 120 | 4(3) | 3(2) | 0.42 |
| EIF4H | 27425 | 120 | 8(6) | 5(4) | 0.58 |
| ANXA3 | 36524 | 120 | 9(4) | 8(4) | 0.42 |
| N/A | 137110 | 120 | 8(3) | 8(3) | 0.07 |
| PRPF19 | 55603 | 120 | 10(6) | 7(4) | 0.26 |
| CTTN | 57603 | 119 | 6(2) | 5(2) | 0.18 |
| HEL-S-129m | 28876 | 119 | 13(7) | 9(7) | 1.15 |
| RPL19 | 23347 | 119 | 4(2) | 4(2) | 0.31 |
| N/A | 60116 | 119 | 7(3) | 7(3) | 0.17 |
| RPS5 | 23033 | 119 | 12(7) | 8(6) | 1.25 |
| SURF4 | 21342 | 118 | 3(2) | 3(2) | 0.34 |
| RAB11B | 24588 | 117 | 8(5) | 6(4) | 0.89 |
| N/A | 39578 | 117 | 8(4) | 6(3) | 0.27 |
| NNMT | 30011 | 117 | 5(4) | 3(3) | 0.52 |
| SF3B1 | 146479 | 117 | 16(5) | 14(5) | 0.12 |
| LUC7L3 | 58698 | 117 | 5(3) | 5(3) | 0.18 |
| SFRS2 | 25461 | 116 | 4(3) | 4(3) | 0.45 |
| PDIA3 | 13739 | 116 | 7(3) | 5(2) | 0.56 |
| CACYBP | 26308 | 116 | 14(5) | 9(4) | 0.61 |
| APEX1 | 29457 | 116 | 7(4) | 7(4) | 0.54 |
| PRKCSH | 60228 | 115 | 12(5) | 9(5) | 0.30 |
| IPO9 | 116858 | 114 | 4(3) | 4(3) | 0.09 |
| N/A | 109456 | 114 | 10(4) | 10(4) | 0.13 |
| CAV1 | 20630 | 114 | 7(4) | 5(2) | 0.83 |
| CPNE1 | 60307 | 114 | 4(4) | 3(3) | 0.17 |
| FARSLA | 57585 | 113 | 4(2) | 4(2) | 0.12 |
| N/A | 27971 | 113 | 6(3) | 5(3) | 0.57 |
| HEL103 | 75130 | 111 | 4(3) | 4(3) | 0.14 |
| NSFL1C | 40548 | 111 | 6(2) | 6(2) | 0.17 |
| HADHB | 51547 | 111 | 13(6) | 9(5) | 0.36 |
| HSD17B4 | 80092 | 111 | 7(4) | 6(4) | 0.27 |
| CRYZ | 35356 | 111 | 7(4) | 7(4) | 0.43 |
| N/A | 26584 | 110 | 7(4) | 5(4) | 0.81 |
| TRAJ56 | 2220 | 110 | 35(15) | 2(1) | 1.81 |
| GRWD1 | 25632 | 109 | 4(3) | 3(2) | 0.28 |
| N/A | 33249 | 109 | 5(3) | 4(3) | 0.33 |
| MCM3 | 91551 | 108 | 8(5) | 7(4) | 0.15 |
| PSMA4 | 26645 | 108 | 7(6) | 6(5) | 0.80 |
| AHNAK2 | 617383 | 107 | 13(3) | 10(3) | 0.02 |
| GNAI3 | 41076 | 106 | 4(2) | 4(2) | 0.17 |
| NOP2 | 89589 | 106 | 7(3) | 7(3) | 0.11 |
| N/A | 51811 | 106 | 9(6) | 9(6) | 0.45 |
| RPL10A | 24987 | 106 | 20(6) | 7(3) | 1.12 |
| RAP1B | 21040 | 106 | 3(2) | 3(2) | 0.34 |
| GOLT1B | 8208 | 105 | 1(1) | 1(1) | 0.43 |
| TMEM113 | 35456 | 105 | 5(3) | 4(2) | 0.20 |
| N/A | 27552 | 105 | 6(2) | 4(1) | 0.12 |
| HEL-S-107 | 29032 | 105 | 7(4) | 5(3) | 0.38 |
| ECHS1 | 31823 | 104 | 4(2) | 4(2) | 0.22 |
| EFHD2 | 26794 | 104 | 10(4) | 8(4) | 0.60 |
| TMEM33 | 28302 | 104 | 3(2) | 2(1) | 0.12 |
| NIT2 | 30988 | 104 | 3(2) | 2(2) | 0.23 |
| PSMA1 | 29822 | 104 | 16(5) | 11(5) | 0.88 |
| N/A | 109111 | 104 | 15(4) | 13(4) | 0.16 |
| MCM4 | 97086 | 103 | 12(3) | 11(3) | 0.14 |
| RCC2 | 56790 | 103 | 16(7) | 10(5) | 0.57 |
| PSMC4 | 47451 | 102 | 14(6) | 12(6) | 0.71 |
| PRMT1 | 43061 | 102 | 12(6) | 8(5) | 0.56 |
| KARS | 68461 | 102 | 14(6) | 11(5) | 0.32 |
| PSMB3 | 23219 | 102 | 7(3) | 5(2) | 0.50 |
| N/A | 80883 | 102 | 11(3) | 9(3) | 0.17 |
| KIF5B-RET(NM_020630)_K22;R12 | 134971 | 102 | 14(4) | 13(3) | 0.13 |
| N/A | 142874 | 102 | 7(2) | 7(2) | 0.05 |
| MAP1B | 271651 | 101 | 12(5) | 12(5) | 0.06 |
| N/A | 28570 | 101 | 6(4) | 5(4) | 0.56 |
| DENR | 22477 | 100 | 6(3) | 3(2) | 0.52 |
| RSL1D1 | 49688 | 100 | 9(2) | 9(2) | 0.14 |
| CAPZB | 29562 | 100 | 12(5) | 8(5) | 0.71 |
| CDK1 | 34117 | 99 | 10(4) | 8(4) | 0.75 |
| N/A | 74930 | 99 | 4(3) | 4(3) | 0.14 |
| EIF3S1 | 29159 | 99 | 3(2) | 3(2) | 0.24 |
| LASP1 | 30097 | 99 | 5(2) | 4(1) | 0.37 |
| hCG_31253 | 61944 | 98 | 7(3) | 7(3) | 0.17 |
| N/A | 45423 | 98 | 9(3) | 7(3) | 0.32 |
| PPP5C | 57412 | 98 | 4(1) | 4(1) | 0.06 |
| GCLC | 69327 | 98 | 4(3) | 4(3) | 0.15 |
| FH | 54773 | 98 | 7(3) | 7(3) | 0.19 |
| SERPINB6 | 42936 | 98 | 8(6) | 5(4) | 0.45 |
| PSMB6 | 25570 | 97 | 2(2) | 2(2) | 0.28 |
| DUT | 15500 | 97 | 3(2) | 3(2) | 0.48 |
| NMT1 | 56910 | 97 | 4(2) | 4(2) | 0.12 |
| FUS | 53693 | 97 | 8(4) | 6(3) | 0.35 |
| COPA | 139797 | 97 | 15(6) | 13(5) | 0.15 |
| GSTK1 | 19429 | 96 | 5(2) | 4(2) | 0.38 |
| DPP3 | 84666 | 96 | 3(2) | 3(2) | 0.08 |
| N/A | 38365 | 96 | 8(3) | 6(3) | 0.28 |
| HDLBP | 141979 | 95 | 12(4) | 10(3) | 0.07 |
| CAPZA2 | 33157 | 95 | 4(3) | 4(3) | 0.46 |
| N/A | 49458 | 95 | 10(6) | 7(5) | 0.38 |
| ROCK2 | 161939 | 95 | 13(4) | 9(4) | 0.08 |
| KRT15 | 49409 | 95 | 6(3) | 4(3) | 0.29 |
| SRPRB | 29912 | 94 | 2(2) | 2(2) | 0.24 |
| NOP56 | 50068 | 94 | 9(4) | 8(4) | 0.29 |
| ALDH1B1 | 57626 | 94 | 6(3) | 5(3) | 0.18 |
| N/A | 50135 | 94 | 4(1) | 4(1) | 0.07 |
| SNRPN | 17706 | 93 | 5(4) | 3(3) | 0.69 |
| N/A | 32782 | 93 | 6(4) | 5(4) | 0.47 |
| VARS | 141642 | 93 | 8(3) | 8(3) | 0.07 |
| SLC25A3 | 40389 | 93 | 17(5) | 11(4) | 0.48 |
| ARPC2 | 34426 | 93 | 9(4) | 7(4) | 0.74 |
| N/A | 117367 | 93 | 7(3) | 6(2) | 0.06 |
| HEL-S-303 | 25724 | 92 | 4(3) | 3(2) | 0.28 |
| NAA15 | 102391 | 92 | 9(4) | 9(4) | 0.17 |
| NAT10 | 94217 | 92 | 5(2) | 4(2) | 0.07 |
| N/A | 91898 | 92 | 15(6) | 14(5) | 0.23 |
| APRT | 17665 | 92 | 8(6) | 5(4) | 1.40 |
| hCG_1640809 | 36049 | 91 | 4(1) | 3(1) | 0.09 |
| CBX3 | 20969 | 91 | 9(2) | 7(2) | 0.35 |
| N/A | 27806 | 91 | 10(5) | 6(3) | 0.57 |
| GLO1 | 20992 | 91 | 7(4) | 5(3) | 0.56 |
| GRHPR | 39241 | 91 | 5(2) | 5(2) | 0.18 |
| CLPTM1 | 76277 | 90 | 3(2) | 3(2) | 0.09 |
| KRT85 | 57306 | 90 | 8(4) | 6(4) | 0.25 |
| PAFAH1B3 | 25832 | 90 | 8(3) | 4(1) | 0.13 |
| U2AF2 | 53445 | 90 | 7(3) | 4(3) | 0.20 |
| ERO1A | 55213 | 90 | 3(1) | 3(1) | 0.06 |
| N/A | 45783 | 89 | 14(4) | 11(4) | 0.42 |
| N/A | 28919 | 89 | 4(2) | 4(2) | 0.24 |
| CYB5B | 15878 | 89 | 1(1) | 1(1) | 0.21 |
| APMAP | 45662 | 89 | 9(4) | 6(4) | 0.32 |
| ETF1 | 47731 | 89 | 10(5) | 8(4) | 0.40 |
| EIF5 | 49648 | 88 | 5(2) | 5(2) | 0.14 |
| SERBP1 | 49175 | 87 | 7(3) | 6(3) | 0.38 |
| NUDT21 | 26268 | 87 | 6(3) | 4(2) | 0.61 |
| N/A | 145007 | 86 | 6(3) | 6(3) | 0.07 |
| CMPK | 26180 | 86 | 4(3) | 4(3) | 0.43 |
| TARDBP | 45053 | 86 | 2(2) | 2(2) | 0.15 |
| HARS | 57944 | 86 | 9(3) | 8(3) | 0.32 |
| N/A | 61017 | 86 | 9(2) | 8(2) | 0.17 |
| HNRPH3 | 36960 | 86 | 4(2) | 4(2) | 0.19 |
| PTPA | 37637 | 86 | 3(2) | 3(2) | 0.18 |
| N/A | 42340 | 85 | 6(2) | 4(2) | 0.16 |
| PDHB | 39550 | 85 | 6(2) | 5(2) | 0.27 |
| ASNS | 64899 | 85 | 5(2) | 5(2) | 0.10 |
| ATP5O | 23377 | 85 | 7(4) | 5(4) | 0.71 |
| N/A | 67899 | 85 | 4(2) | 3(2) | 0.10 |
| HIST1H2AG | 14083 | 84 | 7(5) | 3(3) | 1.38 |
| KRT14 | 51872 | 84 | 7(4) | 6(4) | 0.28 |
| N/A | 110817 | 84 | 15(5) | 13(5) | 0.16 |
| TOMM22 | 15512 | 83 | 2(1) | 2(1) | 0.48 |
| DBN1 | 66053 | 83 | 3(2) | 3(2) | 0.10 |
| PPP1R7 | 41653 | 83 | 2(1) | 2(1) | 0.08 |
| N/A | 40658 | 83 | 1(1) | 1(1) | 0.08 |
| CHP | 22442 | 83 | 2(2) | 2(2) | 0.32 |
| PLAA | 67832 | 83 | 2(2) | 2(2) | 0.10 |
| SHMT1 | 49601 | 82 | 4(1) | 3(1) | 0.07 |
| C9orf88 | 83144 | 82 | 6(3) | 6(3) | 0.17 |
| NUDC | 38276 | 82 | 9(3) | 6(3) | 0.39 |
| SHMT2 | 53821 | 82 | 14(7) | 12(6) | 0.52 |
| CTNNB1 | 86069 | 82 | 7(2) | 5(2) | 0.12 |
| N/A | 103493 | 81 | 9(4) | 9(4) | 0.13 |
| ARCN1 | 57630 | 81 | 9(2) | 7(1) | 0.12 |
| N/A | 37870 | 81 | 5(3) | 5(3) | 0.29 |
| SRSF1 | 28426 | 80 | 9(3) | 7(2) | 0.25 |
| N/A | 59605 | 80 | 5(2) | 5(2) | 0.17 |
| N/A | 15326 | 80 | 1(1) | 1(1) | 0.22 |
| DRG1 | 40802 | 80 | 6(4) | 6(4) | 0.37 |
| ATXN10 | 54196 | 80 | 2(1) | 2(1) | 0.13 |
| SARS | 58883 | 79 | 3(2) | 2(1) | 0.11 |
| CTNND1 | 68559 | 79 | 6(3) | 6(3) | 0.15 |
| N/A | 37693 | 78 | 10(4) | 8(4) | 0.52 |
| FEN1 | 42908 | 78 | 9(3) | 8(3) | 0.25 |
| EIF3K | 24771 | 78 | 3(3) | 3(3) | 0.46 |
| SEC22B | 24806 | 78 | 5(3) | 5(3) | 0.66 |
| RPL21 | 18610 | 77 | 9(2) | 5(1) | 0.94 |
| EHD4 | 61365 | 77 | 5(3) | 5(3) | 0.17 |
| RNPEP | 73234 | 77 | 5(2) | 5(2) | 0.09 |
| PYCR1 | 33568 | 76 | 2(2) | 2(2) | 0.21 |
| N/A | 58987 | 76 | 7(3) | 7(3) | 0.18 |
| ERP44 | 47341 | 76 | 3(1) | 3(1) | 0.07 |
| N/A | 74244 | 76 | 3(1) | 3(1) | 0.04 |
| N/A | 22993 | 76 | 5(2) | 5(2) | 0.50 |
| HACD3 | 43360 | 76 | 3(2) | 3(2) | 0.25 |
| N/A | 86515 | 76 | 4(3) | 3(2) | 0.12 |
| NDUFA10 | 41067 | 76 | 1(1) | 1(1) | 0.08 |
| N/A | 32173 | 75 | 2(1) | 2(1) | 0.10 |
| TP53I3 | 26987 | 75 | 5(4) | 4(3) | 0.42 |
| ARHGEF1 | 103056 | 75 | 7(1) | 7(1) | 0.06 |
| HEL-S-106 | 56413 | 75 | 9(3) | 8(3) | 0.19 |
| GRSF1 | 36761 | 75 | 1(1) | 1(1) | 0.09 |
| NSF | 82553 | 75 | 3(2) | 3(2) | 0.08 |
| N/A | 126101 | 75 | 4(2) | 4(2) | 0.05 |
| ARHG | 21751 | 75 | 3(1) | 3(1) | 0.15 |
| CRIP2 | 21268 | 75 | 5(2) | 3(2) | 0.34 |
| AP2A2 | 102443 | 75 | 5(4) | 5(4) | 0.13 |
| N/A | 41226 | 74 | 4(1) | 4(1) | 0.08 |
| EIF2S3 | 51647 | 74 | 11(3) | 8(3) | 0.20 |
| GFPT1 | 79555 | 74 | 7(3) | 7(3) | 0.13 |
| COPS8 | 23268 | 73 | 3(2) | 3(2) | 0.50 |
| FDFT1 | 54450 | 73 | 2(1) | 2(1) | 0.06 |
| TXLNA | 62195 | 73 | 3(1) | 3(1) | 0.05 |
| IRF2BP2 | 61728 | 73 | 2(2) | 2(2) | 0.11 |
| HIST1H4H | 11364 | 73 | 4(3) | 2(2) | 0.70 |
| MCM5 | 83031 | 72 | 15(3) | 13(3) | 0.12 |
| IARS2 | 114688 | 72 | 9(2) | 9(2) | 0.09 |
| MSH6 | 154514 | 72 | 4(1) | 4(1) | 0.04 |
| PTGES3 | 18971 | 71 | 5(3) | 4(3) | 0.63 |
| ATL3 | 60960 | 71 | 5(2) | 4(1) | 0.11 |
| ATP5PD | 8910 | 71 | 5(2) | 4(2) | 0.94 |
| USP10 | 87707 | 71 | 4(1) | 4(1) | 0.12 |
| MTDH | 63856 | 71 | 2(1) | 2(1) | 0.05 |
| NUP155 | 156697 | 71 | 6(3) | 6(3) | 0.06 |
| hCG_24487 | 21611 | 71 | 4(3) | 3(3) | 0.78 |
| SNX1 | 35628 | 71 | 3(2) | 3(2) | 0.19 |
| SETD3 | 67557 | 70 | 1(1) | 1(1) | 0.05 |
| DDX18 | 75702 | 70 | 8(3) | 7(3) | 0.14 |
| GPRC5A | 40624 | 70 | 3(2) | 3(2) | 0.17 |
| EIF2S2 | 38706 | 70 | 7(5) | 6(4) | 0.39 |
| DDX46 | 117902 | 69 | 8(3) | 8(3) | 0.09 |
| RTN4 | 130250 | 69 | 4(1) | 4(1) | 0.03 |
| EIF3E | 52622 | 69 | 13(5) | 9(5) | 0.35 |
| NCBP1 | 92864 | 69 | 3(2) | 3(2) | 0.07 |
| ABCE1 | 68240 | 69 | 5(3) | 4(3) | 0.15 |
| PARVA | 46618 | 69 | 6(2) | 5(2) | 0.15 |
| DCTN2 | 29423 | 69 | 3(1) | 3(1) | 0.11 |
| KPNA4 | 19041 | 68 | 3(1) | 2(1) | 0.18 |
| JUP | 82416 | 68 | 6(1) | 5(1) | 0.04 |
| HYOU1 | 104941 | 68 | 12(3) | 11(3) | 0.10 |
| KIAA1609 | 51570 | 68 | 1(1) | 1(1) | 0.06 |
| JTV1 | 35668 | 68 | 3(2) | 3(2) | 0.19 |
| N/A | 66146 | 68 | 4(2) | 4(2) | 0.10 |
| ECI1 | 33080 | 68 | 4(1) | 4(1) | 0.21 |
| CPNE3 | 60947 | 68 | 8(4) | 6(4) | 0.23 |
| N/A | 46709 | 68 | 4(1) | 4(1) | 0.15 |
| LXRβ | 51101 | 68 | 6(4) | 5(4) | 0.29 |
| CHMP2A | 25088 | 68 | 1(1) | 1(1) | 0.13 |
| MAP2K1 | 43753 | 68 | 8(2) | 7(2) | 0.16 |
| N/A | 55311 | 67 | 4(2) | 4(2) | 0.12 |
| MAGED2 | 65085 | 67 | 2(1) | 2(1) | 0.05 |
| DTYMK | 23976 | 67 | 4(2) | 4(2) | 0.30 |
| PES1 | 68359 | 67 | 7(2) | 5(2) | 0.10 |
| RTCB | 55688 | 67 | 6(3) | 6(3) | 0.19 |
| N/A | 38087 | 67 | 2(1) | 2(1) | 0.09 |
| N/A | 99650 | 66 | 3(3) | 1(1) | 0.03 |
| HEL-S-304 | 27815 | 66 | 7(3) | 5(2) | 0.40 |
| BASP1 | 22680 | 66 | 4(3) | 4(3) | 0.51 |
| RAB7A | 23760 | 66 | 6(4) | 4(3) | 0.48 |
| CDC37 | 44938 | 65 | 7(3) | 5(3) | 0.24 |
| FDXR | 57705 | 65 | 2(2) | 2(2) | 0.12 |
| RCC1 | 47237 | 65 | 7(3) | 5(3) | 0.22 |
| RPA1 | 68723 | 65 | 5(1) | 4(1) | 0.05 |
| N/A | 102277 | 65 | 10(3) | 10(3) | 0.10 |
| hCG_1811539 | 36725 | 65 | 7(3) | 7(3) | 0.30 |
| RRAS | 23637 | 65 | 2(2) | 2(2) | 0.30 |
| TROVE2 | 61372 | 65 | 3(2) | 3(2) | 0.11 |
| EIF3M | 42932 | 65 | 6(2) | 4(2) | 0.16 |
| TEX10 | 106349 | 65 | 13(1) | 8(1) | 0.03 |
| N/A | 96323 | 65 | 7(1) | 7(1) | 0.03 |
| LONP1 | 95436 | 65 | 6(1) | 6(1) | 0.03 |
| ARL3 | 20614 | 65 | 3(3) | 3(3) | 0.57 |
| N/A | 62590 | 65 | 7(1) | 6(1) | 0.23 |
| HPGD | 19217 | 64 | 3(2) | 3(2) | 0.38 |
| SF1 | 59902 | 64 | 9(2) | 7(2) | 0.17 |
| PSMD4 | 41281 | 64 | 6(2) | 6(2) | 0.17 |
| RAB34 | 29482 | 64 | 3(1) | 3(1) | 0.11 |
| DDX23 | 95866 | 64 | 5(1) | 5(1) | 0.03 |
| CHORDC1 | 38308 | 64 | 7(3) | 5(3) | 0.39 |
| CNN2 | 19916 | 64 | 5(1) | 5(1) | 0.37 |
| DDX1 | 74898 | 63 | 10(2) | 10(2) | 0.14 |
| FLJ10842 | 47564 | 63 | 1(1) | 1(1) | 0.07 |
| STT3B | 94241 | 63 | 6(3) | 4(3) | 0.11 |
| MX1 | 73689 | 63 | 6(1) | 6(1) | 0.09 |
| NME1 | 17309 | 63 | 3(2) | 3(2) | 0.43 |
| UQCRFS1 | 29934 | 63 | 3(2) | 2(2) | 0.23 |
| UBFD1 | 32734 | 63 | 2(1) | 2(1) | 0.10 |
| N/A | 18018 | 62 | 1(1) | 1(1) | 0.19 |
| ILVBL | 68452 | 62 | 2(1) | 2(1) | 0.05 |
| GALNT2 | 67186 | 62 | 7(2) | 6(2) | 0.15 |
| EIF5B | 139053 | 62 | 5(2) | 5(2) | 0.07 |
| ACAA2 | 42032 | 62 | 8(3) | 8(3) | 0.25 |
| DDX19A | 50944 | 62 | 4(1) | 4(1) | 0.06 |
| N/A | 61818 | 62 | 1(1) | 1(1) | 0.05 |
| N/A | 31558 | 62 | 2(1) | 2(1) | 0.11 |
| HEATR1 | 244275 | 62 | 8(1) | 7(1) | 0.03 |
| SUCLG2 | 46824 | 62 | 4(2) | 4(2) | 0.15 |
| FAHD1 | 25112 | 62 | 4(1) | 3(1) | 0.13 |
| CNN3 | 36562 | 61 | 2(1) | 2(1) | 0.19 |
| N/A | 37330 | 61 | 6(2) | 6(2) | 0.19 |
| GEMIN5 | 170664 | 61 | 6(1) | 3(1) | 0.02 |
| SNX6 | 48116 | 61 | 5(1) | 5(1) | 0.07 |
| RPL13A | 24258 | 61 | 11(5) | 8(3) | 0.47 |
| MLEC | 16776 | 61 | 4(2) | 4(2) | 0.44 |
| N/A | 52071 | 61 | 4(1) | 4(1) | 0.06 |
| COPE | 37072 | 60 | 6(2) | 5(2) | 0.19 |
| ME2 | 61810 | 60 | 2(2) | 2(2) | 0.11 |
| TOR1AIP1 | 52545 | 60 | 1(1) | 1(1) | 0.06 |
| IDE | 118692 | 60 | 7(2) | 6(2) | 0.06 |
| PSMD6 | 45787 | 60 | 7(3) | 6(3) | 0.23 |
| PRMT5 | 32462 | 60 | 4(1) | 4(1) | 0.10 |
| SMC1A | 143771 | 60 | 14(2) | 11(2) | 0.05 |
| H2AFY | 39764 | 59 | 5(1) | 5(1) | 0.08 |
| N/A | 39058 | 59 | 5(2) | 5(2) | 0.18 |
| DARS2 | 74086 | 59 | 6(2) | 5(2) | 0.14 |
| N/A | 42596 | 59 | 6(3) | 6(3) | 0.25 |
| TMPO | 76016 | 59 | 6(1) | 5(1) | 0.09 |
| RPL18A | 18352 | 59 | 5(2) | 3(1) | 0.66 |
| PPT1 | 34038 | 59 | 1(1) | 1(1) | 0.10 |
| DEK | 42933 | 59 | 7(2) | 6(2) | 0.16 |
| RNPS1 | 34188 | 58 | 1(1) | 1(1) | 0.10 |
| ALDH7A1 | 58673 | 58 | 6(1) | 6(1) | 0.06 |
| LGALS3BP | 66202 | 58 | 2(1) | 2(1) | 0.05 |
| ACTR2 | 45017 | 58 | 6(1) | 5(1) | 0.07 |
| TMPO | 50696 | 58 | 3(1) | 3(1) | 0.06 |
| hCG_39634 | 21601 | 58 | 2(2) | 2(2) | 0.33 |
| HEBP1 | 21198 | 58 | 2(1) | 2(1) | 0.16 |
| N/A | 46447 | 58 | 7(2) | 7(2) | 0.15 |
| N/A | 85719 | 57 | 2(2) | 1(1) | 0.08 |
| NCKAP1 | 130018 | 57 | 4(2) | 4(2) | 0.05 |
| N/A | 130062 | 57 | 2(2) | 2(2) | 0.05 |
| CYB5R3 | 33445 | 57 | 4(1) | 4(1) | 0.21 |
| ITGA2 | 89418 | 57 | 4(1) | 4(1) | 0.04 |
| SH3KBP1 | 70572 | 57 | 5(2) | 4(1) | 0.10 |
| N/A | 33692 | 57 | 5(3) | 4(3) | 0.32 |
| N/A | 92520 | 57 | 5(2) | 4(1) | 0.04 |
| SRSF7 | 15981 | 57 | 6(2) | 5(2) | 0.47 |
| DERA | 35494 | 56 | 2(1) | 2(1) | 0.09 |
| HEL-S-84 | 38866 | 56 | 3(2) | 3(2) | 0.18 |
| FBXO22 | 45278 | 56 | 4(1) | 4(1) | 0.07 |
| SNF8 | 28960 | 56 | 1(1) | 1(1) | 0.11 |
| LMNB2 | 70020 | 56 | 8(2) | 8(2) | 0.10 |
| DAZAP1 | 43584 | 56 | 2(1) | 2(1) | 0.16 |
| NOLC1 | 74611 | 56 | 3(1) | 3(1) | 0.04 |
| HDAC1 | 55611 | 56 | 6(2) | 5(2) | 0.19 |
| EXOSC5 | 21756 | 56 | 1(1) | 1(1) | 0.15 |
| N/A | 103768 | 56 | 8(2) | 7(2) | 0.06 |
| hCG_2043376 | 22334 | 56 | 6(2) | 4(2) | 0.32 |
| HIBADH | 35705 | 55 | 3(1) | 3(1) | 0.19 |
| RANBP1 | 23467 | 55 | 8(2) | 6(2) | 0.49 |
| CSDE1 | 89684 | 55 | 7(1) | 6(1) | 0.04 |
| RANGAP1 | 63958 | 55 | 6(2) | 6(2) | 0.11 |
| ATP1B1 | 35438 | 55 | 2(1) | 2(1) | 0.09 |
| LTB4DH | 36091 | 54 | 5(1) | 5(1) | 0.19 |
| OSBPL8 | 101759 | 54 | 1(1) | 1(1) | 0.03 |
| PNPT1 | 86524 | 54 | 9(2) | 5(2) | 0.08 |
| PSMB7 | 30260 | 54 | 5(1) | 4(1) | 0.23 |
| KBTBD3 | 71220 | 54 | 5(4) | 1(1) | 0.05 |
| N/A | 15857 | 54 | 3(1) | 2(1) | 0.21 |
| N/A | 71811 | 53 | 2(1) | 2(1) | 0.05 |
| DLAT | 69438 | 53 | 1(1) | 1(1) | 0.05 |
| ATXN2L | 103919 | 53 | 5(2) | 5(2) | 0.06 |
| DDX54 | 98819 | 53 | 4(1) | 3(1) | 0.03 |
| N/A | 113662 | 53 | 3(1) | 2(1) | 0.03 |
| SUPT16H | 120409 | 53 | 10(2) | 9(2) | 0.08 |
| BLMH | 37630 | 53 | 2(1) | 2(1) | 0.09 |
| NAPA | 33667 | 53 | 5(3) | 5(3) | 0.33 |
| ANP32E | 30902 | 53 | 5(1) | 3(1) | 0.11 |
| N/A | 57810 | 53 | 5(2) | 4(2) | 0.12 |
| VPS26A | 38260 | 52 | 3(2) | 3(2) | 0.18 |
| GNL3 | 61048 | 52 | 4(1) | 4(1) | 0.11 |
| GCLM | 31050 | 52 | 6(3) | 4(3) | 0.36 |
| INF2 | 136851 | 52 | 3(1) | 3(1) | 0.02 |
| TRMT6 | 56049 | 52 | 1(1) | 1(1) | 0.06 |
| PRPF31 | 55649 | 52 | 4(1) | 3(1) | 0.12 |
| SEC24C | 119789 | 52 | 2(1) | 2(1) | 0.06 |
| N/A | 19076 | 51 | 3(2) | 3(2) | 0.38 |
| TBCD | 138346 | 51 | 4(2) | 4(2) | 0.05 |
| N/A | 36123 | 51 | 4(1) | 4(1) | 0.09 |
| ARHGAP1 | 38420 | 51 | 4(1) | 4(1) | 0.18 |
| RBM8 | 19934 | 51 | 1(1) | 1(1) | 0.17 |
| ATAD3A | 64600 | 51 | 12(2) | 8(2) | 0.10 |
| PFN1 | 11497 | 51 | 1(1) | 1(1) | 0.30 |
| TCEA1 | 34370 | 51 | 3(1) | 3(1) | 0.10 |
| PDAP1 | 4388 | 51 | 1(1) | 1(1) | 0.84 |
| N/A | 59082 | 51 | 4(2) | 4(2) | 0.11 |
| RPL7L1 | 29822 | 51 | 4(2) | 2(2) | 0.24 |
| HEL-S-64p | 52523 | 51 | 5(1) | 5(1) | 0.06 |
| N/A | 21798 | 51 | 5(1) | 4(1) | 0.33 |
| N/A | 39078 | 50 | 3(2) | 3(2) | 0.18 |
| N/A | 26623 | 50 | 9(2) | 6(2) | 0.27 |
| N/A | 34130 | 50 | 5(1) | 4(1) | 0.20 |
| RAC1 | 23851 | 50 | 9(3) | 6(3) | 0.93 |
| NUP93 | 93943 | 50 | 7(3) | 7(3) | 0.11 |
| SQSTM1-ALK | 91234 | 50 | 3(3) | 2(2) | 0.11 |
| AP2A1 | 108561 | 50 | 5(3) | 5(3) | 0.09 |
| EIF1AY | 16546 | 50 | 2(1) | 2(1) | 0.20 |
| C12orf10 | 34140 | 50 | 3(1) | 2(1) | 0.10 |
| N/A | 61742 | 50 | 1(1) | 1(1) | 0.05 |
| N/A | 147082 | 49 | 6(1) | 6(1) | 0.04 |
| CDK6 | 37257 | 49 | 4(2) | 3(2) | 0.19 |
| N/A | 40186 | 49 | 3(2) | 1(1) | 0.08 |
| EPS8L2 | 81197 | 49 | 3(1) | 2(1) | 0.04 |
| PAIP1 | 35277 | 49 | 3(2) | 3(2) | 0.20 |
| DUSP3 | 20792 | 49 | 1(1) | 1(1) | 0.16 |
| LAMB1 | 207703 | 49 | 2(1) | 2(1) | 0.02 |
| LARP4 | 21878 | 49 | 1(1) | 1(1) | 0.15 |
| SORD | 12760 | 49 | 2(1) | 2(1) | 0.27 |
| TOP1 | 91099 | 49 | 10(1) | 8(1) | 0.04 |
| DBNL | 40475 | 49 | 2(1) | 2(1) | 0.08 |
| SEC31L1 | 133900 | 49 | 3(2) | 3(2) | 0.05 |
| DHFR | 28940 | 49 | 2(1) | 2(1) | 0.11 |
| DIABLO | 18907 | 49 | 3(1) | 2(1) | 0.18 |
| LXRα | 45691 | 49 | 6(2) | 6(2) | 0.07 |
| MRRF | 37224 | 49 | 2(1) | 2(1) | 0.09 |
| CHCHD3 | 26421 | 48 | 3(1) | 3(1) | 0.13 |
| N/A | 33950 | 48 | 2(1) | 2(1) | 0.10 |
| N/A | 77022 | 48 | 4(2) | 3(2) | 0.09 |
| N/A | 59889 | 48 | 3(2) | 3(2) | 0.11 |
| RECQL | 74436 | 48 | 7(2) | 6(1) | 0.09 |
| TSR1 | 92151 | 48 | 4(2) | 4(2) | 0.07 |
| N/A | 112359 | 48 | 14(2) | 13(2) | 0.12 |
| EPHA2 | 109679 | 48 | 4(1) | 4(1) | 0.03 |
| UBTF | 58487 | 48 | 4(1) | 4(1) | 0.06 |
| N/A | 30781 | 48 | 4(1) | 2(1) | 0.11 |
| APOA1BP | 33948 | 48 | 4(2) | 4(2) | 0.32 |
| GTPBP4 | 74317 | 48 | 6(2) | 6(2) | 0.09 |
| NIPSNAP2 | 7934 | 48 | 2(1) | 2(1) | 0.45 |
| PHGDH | 56644 | 48 | 3(2) | 3(2) | 0.12 |
| N/A | 39891 | 47 | 1(1) | 1(1) | 0.08 |
| COQ8B | 22629 | 47 | 3(1) | 2(1) | 0.15 |
| RAE1 | 14945 | 47 | 1(1) | 1(1) | 0.23 |
| N/A | 32679 | 47 | 1(1) | 1(1) | 0.10 |
| IDH3A | 19292 | 47 | 3(2) | 2(2) | 0.38 |
| KLC1 | 63133 | 47 | 4(2) | 4(2) | 0.11 |
| HSD17B12 | 30456 | 47 | 2(1) | 2(1) | 0.11 |
| FAM120A | 54729 | 47 | 3(1) | 3(1) | 0.06 |
| MYO1B | 132928 | 47 | 10(3) | 9(3) | 0.10 |
| MID1 | 76913 | 47 | 2(1) | 2(1) | 0.09 |
| N/A | 26178 | 47 | 3(1) | 3(1) | 0.13 |
| ARL6IP5 | 21600 | 47 | 1(1) | 1(1) | 0.16 |
| MISP | 75482 | 46 | 3(1) | 3(1) | 0.04 |
| PSMG2 | 27078 | 46 | 2(1) | 2(1) | 0.12 |
| EBP | 26564 | 46 | 1(1) | 1(1) | 0.13 |
| N/A | 61808 | 46 | 2(1) | 2(1) | 0.11 |
| NPTN | 44702 | 46 | 2(1) | 2(1) | 0.07 |
| N/A | 42561 | 46 | 3(3) | 3(3) | 0.25 |
| N/A | 72538 | 46 | 2(1) | 2(1) | 0.05 |
| TMEM43 | 44904 | 46 | 5(1) | 4(1) | 0.07 |
| EXOSC4 | 26652 | 46 | 1(1) | 1(1) | 0.13 |
| SMARCE1 | 18417 | 46 | 2(1) | 2(1) | 0.18 |
| HAT1 | 49880 | 46 | 1(1) | 1(1) | 0.07 |
| CHMP4B | 24935 | 46 | 5(1) | 2(1) | 0.13 |
| DNPH1 | 26080 | 46 | 1(1) | 1(1) | 0.13 |
| DNM1 | 94186 | 46 | 4(1) | 4(1) | 0.03 |
| N/A | 66629 | 45 | 7(3) | 6(3) | 0.21 |
| LYPLA1 | 18197 | 45 | 1(1) | 1(1) | 0.19 |
| FLOT1 | 9553 | 45 | 1(1) | 1(1) | 0.36 |
| ANXA6 | 76168 | 45 | 9(2) | 8(2) | 0.13 |
| C9orf32 | 25770 | 45 | 3(1) | 1(1) | 0.13 |
| FXR1 | 76495 | 45 | 2(1) | 2(1) | 0.04 |
| TIGAR | 30442 | 45 | 1(1) | 1(1) | 0.11 |
| PIGT | 66567 | 45 | 2(1) | 2(1) | 0.05 |
| N/A | 48572 | 45 | 3(1) | 3(1) | 0.14 |
| POR | 77429 | 45 | 4(1) | 3(1) | 0.09 |
| EMC3 | 29932 | 45 | 5(1) | 4(1) | 0.23 |
| NEDD8 | 5863 | 45 | 2(1) | 2(1) | 0.61 |
| CPSF1 | 153087 | 45 | 3(2) | 3(2) | 0.04 |
| N/A | 53482 | 45 | 2(1) | 2(1) | 0.06 |
| TSN | 25670 | 45 | 6(2) | 3(2) | 0.28 |
| NOMO1 | 122943 | 45 | 1(1) | 1(1) | 0.03 |
| AK3 | 25605 | 45 | 4(1) | 4(1) | 0.28 |
| UNC45A | 120030 | 44 | 6(1) | 5(1) | 0.06 |
| UGP2 | 57103 | 44 | 11(2) | 9(2) | 0.12 |
| EIF3D | 53364 | 44 | 7(2) | 4(2) | 0.13 |
| DDX27 | 86980 | 44 | 2(2) | 2(2) | 0.08 |
| SLC16A3 | 50064 | 44 | 1(1) | 1(1) | 0.07 |
| SKP1 | 18879 | 44 | 2(1) | 2(1) | 0.18 |
| ELP3 | 62789 | 44 | 2(1) | 2(1) | 0.05 |
| RTN3 | 25764 | 44 | 1(1) | 1(1) | 0.13 |
| N/A | 39160 | 44 | 5(1) | 4(1) | 0.08 |
| N/A | 32616 | 44 | 4(1) | 4(1) | 0.10 |
| TMX2 | 34358 | 43 | 3(1) | 3(1) | 0.10 |
| ITGAV | 117058 | 43 | 3(1) | 3(1) | 0.03 |
| COIL | 63254 | 43 | 3(1) | 2(1) | 0.05 |
| C8orf33 | 25319 | 43 | 1(1) | 1(1) | 0.13 |
| SAE1 | 38882 | 43 | 9(2) | 6(2) | 0.18 |
| AAAS | 46290 | 43 | 5(1) | 5(1) | 0.07 |
| SMS | 41698 | 43 | 7(2) | 6(2) | 0.16 |
| STAU1 | 55140 | 42 | 2(1) | 2(1) | 0.06 |
| SNRP70 | 51583 | 42 | 7(2) | 7(2) | 0.13 |
| RDH11 | 35763 | 42 | 3(1) | 3(1) | 0.09 |
| NCEH1 | 50249 | 42 | 3(2) | 3(2) | 0.14 |
| TFG | 40780 | 42 | 4(1) | 4(1) | 0.17 |
| N/A | 56807 | 42 | 1(1) | 1(1) | 0.06 |
| AK1 | 5828 | 42 | 1(1) | 1(1) | 0.61 |
| HM13 | 41747 | 42 | 3(1) | 3(1) | 0.16 |
| SLC6A6 | 71094 | 42 | 2(1) | 1(1) | 0.05 |
| HSD17B11 | 28426 | 42 | 4(1) | 2(1) | 0.12 |
| N/A | 73566 | 42 | 3(1) | 3(1) | 0.04 |
| ADRM1 | 37652 | 41 | 2(2) | 2(2) | 0.18 |
| SF3B2 | 98223 | 41 | 15(2) | 8(2) | 0.07 |
| N/A | 84999 | 41 | 2(1) | 2(1) | 0.04 |
| BSG | 20643 | 41 | 1(1) | 1(1) | 0.16 |
| PSPC1 | 45713 | 41 | 7(2) | 6(2) | 0.15 |
| DMRTA1 | 53890 | 41 | 4(1) | 2(1) | 0.06 |
| UBLCP1 | 36838 | 41 | 3(2) | 2(2) | 0.19 |
| SRP54 | 55953 | 41 | 7(1) | 5(1) | 0.06 |
| N/A | 40520 | 41 | 5(1) | 5(1) | 0.08 |
| CSK | 51242 | 41 | 1(1) | 1(1) | 0.06 |
| N/A | 25169 | 41 | 2(1) | 2(1) | 0.13 |
| N/A | 29358 | 41 | 2(1) | 2(1) | 0.11 |
| GRB2 | 25304 | 40 | 2(1) | 2(1) | 0.13 |
| TNS3 | 156366 | 40 | 4(1) | 4(1) | 0.02 |
| N/A | 53648 | 40 | 3(1) | 3(1) | 0.06 |
| TMED7-TICAM2 | 21562 | 40 | 2(1) | 2(1) | 0.16 |
| PDHA1 | 43952 | 40 | 4(1) | 4(1) | 0.08 |
| EIF3H | 39736 | 40 | 8(1) | 6(1) | 0.17 |
| DERL1 | 28896 | 40 | 2(1) | 2(1) | 0.12 |
| APEH | 82142 | 40 | 3(2) | 2(2) | 0.08 |
| N/A | 60646 | 40 | 4(2) | 3(2) | 0.17 |
| ITGB5 | 91303 | 40 | 7(1) | 4(1) | 0.04 |
| N/A | 51189 | 40 | 9(3) | 5(2) | 0.13 |
| PSMD8 | 32702 | 40 | 7(1) | 5(1) | 0.10 |
| FAM98B | 37566 | 40 | 3(1) | 2(1) | 0.09 |
| DNAJA2 | 46344 | 40 | 3(2) | 3(2) | 0.15 |
| NT5C2 | 31162 | 40 | 2(1) | 2(1) | 0.11 |
| N/A | 46521 | 40 | 2(1) | 2(1) | 0.07 |
| TMED4 | 26097 | 39 | 6(1) | 3(1) | 0.13 |
| DPM1 | 33537 | 39 | 4(1) | 4(1) | 0.10 |
| HEL-S-39 | 22785 | 39 | 2(1) | 2(1) | 0.15 |
| DKFZp667O202 | 20551 | 39 | 2(1) | 2(1) | 0.16 |
| ASNA1 | 37551 | 39 | 2(1) | 1(1) | 0.09 |
| OAS3 | 122863 | 39 | 1(1) | 1(1) | 0.03 |
| HIST1H1B | 22566 | 39 | 3(2) | 2(2) | 0.32 |
| EIF3S2 | 36878 | 39 | 4(1) | 4(1) | 0.09 |
| FKBP10 | 64717 | 39 | 2(1) | 2(1) | 0.05 |
| SRSF3 | 10599 | 38 | 7(2) | 4(1) | 0.76 |
| CHD3 | 227989 | 38 | 10(1) | 6(1) | 0.01 |
| N/A | 57579 | 38 | 4(2) | 4(2) | 0.12 |
| CSF2RB | 11752 | 38 | 3(2) | 1(1) | 0.29 |
| N/A | 45335 | 38 | 2(1) | 2(1) | 0.07 |
| ME1 | 64679 | 38 | 5(2) | 5(2) | 0.10 |
| IST1 | 23532 | 38 | 2(1) | 2(1) | 0.14 |
| N/A | 42190 | 38 | 1(1) | 1(1) | 0.08 |
| N/A | 113035 | 38 | 2(1) | 2(1) | 0.03 |
| VDP | 108740 | 38 | 6(1) | 5(1) | 0.09 |
| N/A | 33182 | 38 | 2(1) | 2(1) | 0.10 |
| N/A | 33938 | 38 | 1(1) | 1(1) | 0.10 |
| GCS1 | 92021 | 38 | 3(2) | 3(2) | 0.07 |
| ELAC2 | 93415 | 38 | 2(1) | 2(1) | 0.04 |
| MGC11257 | 18557 | 38 | 1(1) | 1(1) | 0.18 |
| N/A | 37408 | 38 | 1(1) | 1(1) | 0.09 |
| ACOT7 | 42454 | 38 | 6(1) | 6(1) | 0.08 |
| N/A | 30313 | 37 | 2(1) | 2(1) | 0.11 |
| CYP51A1 | 57641 | 37 | 2(1) | 2(1) | 0.06 |
| N/A | 61183 | 37 | 2(1) | 2(1) | 0.05 |
| HLA-B | 40809 | 37 | 8(1) | 6(1) | 0.08 |
| FAM207A | 25441 | 37 | 2(1) | 2(1) | 0.13 |
| ATP2B4 | 139030 | 37 | 7(1) | 5(1) | 0.02 |
| EARS2 | 57246 | 37 | 2(1) | 2(1) | 0.06 |
| DDX24 | 91994 | 37 | 5(1) | 4(1) | 0.04 |
| N/A | 37468 | 37 | 3(1) | 3(1) | 0.09 |
| SRC | 60310 | 37 | 1(1) | 1(1) | 0.05 |
| MTCH2 | 33936 | 37 | 2(1) | 2(1) | 0.10 |
| TMED9 | 27374 | 37 | 9(1) | 5(1) | 0.26 |
| SNW1 | 61468 | 37 | 2(1) | 2(1) | 0.05 |
| SLC2A1 | 54361 | 37 | 6(2) | 4(2) | 0.12 |
| EWSR1 | 61662 | 37 | 3(1) | 3(1) | 0.05 |
| C8orf82 | 29093 | 37 | 1(1) | 1(1) | 0.11 |
| SYNE1 | 1011226 | 37 | 27(2) | 18(2) | 0.01 |
| P4HA1 | 61214 | 37 | 3(1) | 3(1) | 0.05 |
| SDHB | 32407 | 36 | 1(1) | 1(1) | 0.10 |
| N/A | 52013 | 36 | 2(1) | 2(1) | 0.13 |
| N/A | 31379 | 36 | 1(1) | 1(1) | 0.11 |
| TAP1 | 87733 | 36 | 1(1) | 1(1) | 0.04 |
| ZYX | 63722 | 36 | 5(1) | 4(1) | 0.05 |
| PROCR | 24551 | 36 | 4(1) | 3(1) | 0.14 |
| U2SURP | 118631 | 36 | 4(1) | 3(1) | 0.03 |
| RSN | 161632 | 36 | 7(1) | 7(1) | 0.02 |
| CALD1 | 93232 | 36 | 7(1) | 7(1) | 0.04 |
| N/A | 61519 | 36 | 3(1) | 3(1) | 0.05 |
| MKL2 | 118282 | 36 | 4(1) | 3(1) | 0.03 |
| RPS6KA5 | 90379 | 36 | 6(1) | 5(1) | 0.04 |
| ETHE1 | 27083 | 36 | 1(1) | 1(1) | 0.12 |
| N/A | 70790 | 36 | 1(1) | 1(1) | 0.05 |
| PAWR | 36659 | 36 | 4(1) | 3(1) | 0.09 |
| TMOD3 | 20946 | 36 | 3(2) | 3(2) | 0.35 |
| N/A | 42687 | 36 | 3(1) | 2(1) | 0.08 |
| BCLAF1 | 80627 | 36 | 4(1) | 4(1) | 0.04 |
| IFIT1 | 55781 | 36 | 2(1) | 2(1) | 0.06 |
| N/A | 81607 | 36 | 5(1) | 2(1) | 0.04 |
| N/A | 18225 | 36 | 1(1) | 1(1) | 0.19 |
| GPS1 | 55500 | 35 | 3(1) | 3(1) | 0.06 |
| GINS4 | 22752 | 35 | 2(1) | 2(1) | 0.15 |
| RPS16 | 5611 | 35 | 1(1) | 1(1) | 0.64 |
| N/A | 117735 | 35 | 6(1) | 5(1) | 0.03 |
| DKFZp781C0419 | 38425 | 35 | 1(1) | 1(1) | 0.09 |
| HMGB3 | 22247 | 35 | 1(1) | 1(1) | 0.15 |
| CREBBP | 268033 | 35 | 2(1) | 2(1) | 0.01 |
| CUL9 | 285440 | 35 | 1(1) | 1(1) | 0.01 |
| EPB41L2 | 104809 | 35 | 5(1) | 3(1) | 0.03 |
| PRPF6 | 107656 | 35 | 8(1) | 8(1) | 0.03 |
| CD63 | 26474 | 35 | 2(2) | 2(2) | 0.27 |
| THEM6 | 24135 | 35 | 1(1) | 1(1) | 0.14 |
| DYNC1LI2 | 54351 | 35 | 2(1) | 2(1) | 0.06 |
| N/A | 58127 | 35 | 1(1) | 1(1) | 0.06 |
| ENAH | 68765 | 35 | 4(1) | 4(1) | 0.05 |
| CPT1A | 88995 | 34 | 3(1) | 2(1) | 0.04 |
| hCG_22119 | 26193 | 34 | 2(1) | 1(1) | 0.13 |
| MOB1B | 25246 | 34 | 3(2) | 2(1) | 0.13 |
| N/A | 58612 | 34 | 6(1) | 2(1) | 0.06 |
| ND4 | 51819 | 34 | 3(1) | 3(1) | 0.06 |
| TMX1 | 32170 | 34 | 7(1) | 4(1) | 0.22 |
| SMAD3 | 48905 | 34 | 2(1) | 2(1) | 0.14 |
| CAPN1 | 82395 | 34 | 9(1) | 6(1) | 0.04 |
| ARFGAP1 | 26653 | 34 | 2(1) | 2(1) | 0.13 |
| MRE11A | 80885 | 34 | 3(1) | 3(1) | 0.04 |
| DECR1 | 36330 | 34 | 3(1) | 3(1) | 0.09 |
| RBM25 | 100467 | 34 | 5(1) | 4(1) | 0.07 |
| NPC1 | 144855 | 34 | 2(1) | 2(1) | 0.02 |
| CFL1 | 17029 | 33 | 1(1) | 1(1) | 0.20 |
| N/A | 121468 | 33 | 1(1) | 1(1) | 0.03 |
| HSPBP1 | 40190 | 33 | 2(1) | 2(1) | 0.17 |
| YKT6 | 22574 | 33 | 3(1) | 2(1) | 0.15 |
| PAFAH1B1 | 47178 | 33 | 5(1) | 5(1) | 0.07 |
| SLC25A10 | 31718 | 33 | 4(1) | 3(1) | 0.10 |
| MMS19 | 114928 | 33 | 4(1) | 3(1) | 0.03 |
| HP1BP3 | 28725 | 33 | 3(1) | 3(1) | 0.12 |
| NANS | 40738 | 33 | 7(1) | 5(1) | 0.08 |
| POLD2 | 55345 | 33 | 3(1) | 2(1) | 0.06 |
| N/A | 93124 | 33 | 2(1) | 2(1) | 0.04 |
| N/A | 46912 | 33 | 4(1) | 4(1) | 0.07 |
| DNAJB1 | 38191 | 33 | 3(1) | 3(1) | 0.09 |
| TMCO1 | 11536 | 33 | 1(1) | 1(1) | 0.30 |
| N/A | 123943 | 33 | 5(1) | 3(1) | 0.03 |
| LAMC1 | 179545 | 33 | 3(1) | 3(1) | 0.02 |
| GJB6 | 30881 | 33 | 1(1) | 1(1) | 0.11 |
| IFIT5 | 56268 | 33 | 4(1) | 3(1) | 0.06 |
| YARS | 59448 | 33 | 9(1) | 8(1) | 0.06 |
| PDLIM5 | 65132 | 33 | 1(1) | 1(1) | 0.05 |
| N/A | 107714 | 32 | 3(1) | 3(1) | 0.03 |
| AKR7A2 | 35004 | 32 | 1(1) | 1(1) | 0.09 |
| N/A | 31287 | 32 | 7(1) | 5(1) | 0.22 |
| LACTB2 | 33070 | 32 | 1(1) | 1(1) | 0.10 |
| N/A | 46544 | 32 | 4(1) | 4(1) | 0.07 |
| CAPRIN1 | 78489 | 32 | 5(1) | 4(1) | 0.04 |
| PATJ | 130799 | 32 | 4(1) | 3(1) | 0.02 |
| N/A | 19304 | 32 | 2(1) | 2(1) | 0.17 |
| N/A | 42622 | 32 | 5(1) | 4(1) | 0.08 |
| MRPS7 | 18488 | 32 | 3(1) | 2(1) | 0.18 |
| DDB1 | 128142 | 32 | 9(2) | 8(1) | 0.05 |
| TNPO3 | 105961 | 32 | 6(2) | 4(2) | 0.06 |
| N/A | 35108 | 32 | 1(1) | 1(1) | 0.09 |
| N/A | 44512 | 32 | 1(1) | 1(1) | 0.07 |
| FLVCR1-AS1 | 10299 | 32 | 5(1) | 2(1) | 0.34 |
| WDR77 | 37442 | 32 | 3(1) | 2(1) | 0.09 |
| MON2 | 192475 | 32 | 3(1) | 3(1) | 0.02 |
| PMPCA | 58729 | 32 | 2(1) | 2(1) | 0.06 |
| HEL25 | 22382 | 32 | 2(1) | 2(1) | 0.15 |
| MPDU1 | 26906 | 32 | 2(1) | 2(1) | 0.12 |
| RFC4 | 23919 | 31 | 6(1) | 5(1) | 0.14 |
| TWF1 | 40429 | 31 | 3(1) | 3(1) | 0.08 |
| MARCKS | 14850 | 31 | 1(1) | 1(1) | 0.23 |
| LARP1 | 123833 | 31 | 3(1) | 3(1) | 0.03 |
| API5 | 57867 | 31 | 6(1) | 6(1) | 0.06 |
| DIMT1 | 31109 | 31 | 3(2) | 2(2) | 0.23 |
| TRIP12 | 222234 | 31 | 5(1) | 4(1) | 0.01 |
| VPS4B | 49443 | 31 | 1(1) | 1(1) | 0.07 |
| TXN | 12015 | 31 | 1(1) | 1(1) | 0.29 |
| MYLK | 213320 | 31 | 3(1) | 3(1) | 0.02 |
| BYSL | 49798 | 31 | 3(1) | 3(1) | 0.07 |
| MAP4K5 | 96047 | 31 | 3(1) | 2(1) | 0.03 |
| COPB2 | 103278 | 31 | 7(0) | 4(0) | 0.03 |
| GNAS | 18894 | 31 | 1(1) | 1(1) | 0.18 |
| PPP1R14B | 21066 | 31 | 1(1) | 1(1) | 0.16 |
| RPL24 | 17646 | 31 | 2(1) | 2(1) | 0.19 |
| N/A | 122217 | 31 | 4(1) | 3(1) | 0.03 |
| ITPA | 4741 | 30 | 2(1) | 2(1) | 0.77 |
| GRPEL1 | 24492 | 30 | 2(1) | 2(1) | 0.14 |
| ZFR | 118081 | 30 | 4(1) | 4(1) | 0.03 |
| LRRC47 | 64004 | 30 | 2(1) | 2(1) | 0.05 |
| SAMM50 | 52342 | 30 | 1(0) | 1(0) | 0.06 |
| UCHL5 | 40853 | 30 | 1(1) | 1(1) | 0.08 |
| N/A | 101354 | 30 | 6(1) | 6(1) | 0.03 |
| N/A | 158464 | 30 | 8(0) | 8(0) | 0.02 |
| SRM | 34373 | 30 | 3(1) | 2(1) | 0.10 |
| UMPS | 33258 | 30 | 1(0) | 1(0) | 0.10 |
| N/A | 41778 | 30 | 5(1) | 5(1) | 0.08 |
| RPL11 | 20167 | 30 | 2(1) | 2(1) | 0.17 |
| HUWE1 | 484651 | 29 | 8(1) | 7(1) | 0.01 |
| N/A | 41137 | 29 | 1(1) | 1(1) | 0.08 |
| DAK | 59282 | 29 | 3(1) | 3(1) | 0.06 |
| WDHD1 | 7094 | 29 | 2(1) | 2(1) | 0.50 |
| B4GalT5 | 44255 | 29 | 5(1) | 2(1) | 0.07 |
| H3F3B | 12971 | 29 | 2(1) | 2(1) | 0.26 |
| CASP8AP2 | 224721 | 29 | 15(1) | 5(1) | 0.01 |
| NFKB2 | 92493 | 29 | 5(1) | 4(1) | 0.04 |
| N/A | 67135 | 29 | 2(1) | 2(1) | 0.05 |
| CPSF3 | 74057 | 29 | 2(1) | 2(1) | 0.04 |
| N/A | 22434 | 29 | 3(1) | 3(1) | 0.32 |
| EEA1 | 163337 | 29 | 11(1) | 10(1) | 0.02 |
| LACTB | 61111 | 29 | 1(0) | 1(0) | 0.05 |
| MRPS9 | 46034 | 29 | 2(1) | 2(1) | 0.07 |
| DHRS7 | 38673 | 29 | 5(0) | 4(0) | 0.09 |
| WDR43 | 75813 | 29 | 2(1) | 2(1) | 0.04 |
| NRDC | 140352 | 28 | 2(1) | 2(1) | 0.02 |
| INPP4A | 106389 | 28 | 6(1) | 3(1) | 0.03 |
| MAPK1 | 41762 | 28 | 1(0) | 1(0) | 0.08 |
| CHURC1-FNTB | 54005 | 28 | 2(1) | 2(1) | 0.06 |
| PTPN2 | 44989 | 28 | 1(0) | 1(0) | 0.07 |
| N/A | 94589 | 28 | 3(1) | 3(1) | 0.03 |
| N/A | 29320 | 28 | 5(1) | 4(1) | 0.11 |
| PLD3 | 55127 | 27 | 1(0) | 1(0) | 0.06 |
| YARS2 | 32279 | 27 | 1(0) | 1(0) | 0.10 |
| N/A | 65749 | 27 | 3(1) | 2(1) | 0.05 |
| N/A | 34775 | 27 | 3(1) | 2(1) | 0.10 |
| TBC1D8B | 130110 | 27 | 3(1) | 3(1) | 0.03 |
| N/A | 16544 | 27 | 1(1) | 1(1) | 0.20 |
| CLINT1 | 68273 | 27 | 3(1) | 3(1) | 0.05 |
| cICK0721Q.2 | 18086 | 27 | 3(1) | 3(1) | 0.19 |
| XPOT | 71552 | 26 | 2(1) | 2(1) | 0.05 |
| ESF1 | 99134 | 26 | 5(0) | 4(0) | 0.03 |
| SCAMP3 | 38674 | 26 | 2(0) | 2(0) | 0.09 |
| ABCA1 | 256391 | 26 | 3(1) | 3(1) | 0.01 |
| N/A | 20903 | 26 | 3(0) | 2(0) | 0.16 |
| N/A | 38805 | 26 | 1(0) | 1(0) | 0.09 |
| N/A | 26848 | 26 | 4(0) | 4(0) | 0.12 |
| hCG_1998851 | 47434 | 26 | 3(0) | 3(0) | 0.07 |
| N/A | 55035 | 26 | 10(0) | 2(0) | 0.06 |
| DENND5B | 6680 | 25 | 1(0) | 1(0) | 0.54 |
| NDUFV2 | 27617 | 25 | 2(1) | 2(1) | 0.26 |
| RAD50 | 154823 | 25 | 8(0) | 6(0) | 0.02 |
| N/A | 32441 | 25 | 1(0) | 1(0) | 0.10 |
| CPOX | 8274 | 25 | 1(0) | 1(0) | 0.42 |
| COLGALT1 | 71933 | 25 | 3(1) | 3(1) | 0.05 |
| GLG1 | 81433 | 25 | 1(0) | 1(0) | 0.04 |
| FKBP9 | 63473 | 24 | 2(0) | 2(0) | 0.05 |
| N/A | 13162 | 23 | 2(0) | 1(0) | 0.26 |
| STOM | 31882 | 23 | 8(0) | 6(0) | 0.22 |
| DOLPP1 | 27127 | 22 | 1(0) | 1(0) | 0.12 |
| VBP1 | 26690 | 22 | 2(0) | 2(0) | 0.13 |
| SPCS2 | 25359 | 22 | 3(0) | 3(0) | 0.13 |
| SAR1B | 19092 | 22 | 3(0) | 3(0) | 0.18 |
| C1orf33 | 27657 | 22 | 2(0) | 2(0) | 0.12 |
| CKAP5 | 227062 | 22 | 10(0) | 10(0) | 0.01 |
| EMP3 | 17532 | 21 | 2(0) | 2(0) | 0.19 |
| DCTPP1 | 18783 | 21 | 2(0) | 2(0) | 0.18 |
| CFAP20 | 22931 | 21 | 3(0) | 3(0) | 0.31 |
| AAMP | 47406 | 21 | 1(0) | 1(0) | 0.07 |
| FAU | 10898 | 20 | 2(0) | 1(0) | 0.32 |
| NPLOC4 | 69046 | 20 | 4(0) | 3(0) | 0.05 |
| ADSL | 55140 | 20 | 7(0) | 5(0) | 0.06 |
| N/A | 21919 | 19 | 2(0) | 2(0) | 0.15 |
| N/A | 74988 | 19 | 7(0) | 7(0) | 0.09 |
| AKT2 | 56090 | 18 | 2(0) | 2(0) | 0.06 |
| KNSTRN | 35701 | 18 | 5(0) | 3(0) | 0.09 |
| EIF2A | 65519 | 18 | 3(0) | 3(0) | 0.05 |
| AACS | 75666 | 17 | 1(0) | 1(0) | 0.04 |
| N/A | 44226 | 16 | 4(0) | 4(0) | 0.07 |
| TACR3 | 52750 | 16 | 1(0) | 1(0) | 0.06 |
| SENP6 | 66572 | 16 | 4(0) | 3(0) | 0.05 |
| N/A | 112131 | 15 | 2(0) | 2(0) | 0.03 |
| N/A | 51952 | 14 | 1(1) | 1(1) | 0.06 |

**Supplementary Table 6. ERE-containing genes (ERβ-responsive genes)**

| ID | P.Value | Gene.symbol |
| --- | --- | --- |
| 211160_x_at | 0.0000227 | ACTN1 |
| 212419_at | 0.000081 | ZCCHC24 |
| 225105_at | 0.0002478 | C12orf75 |
| 65438_at | 0.0003394 | TLDC1 |
| 212136_at | 0.000526 | ATP2B4 |
| 225847_at | 0.0009107 | NCEH1 |
| 221752_at | 0.0013661 | SSH1 |
| 221489_s_at | 0.0013804 | SPRY4 |
| 204032_at | 0.0015221 | BCAR3 |
| 224669_at | 0.0016851 | SYS1 |
| 1555832_s_at | 0.0019538 | KLF6 |
| 227599_at | 0.001965 | MB21D2 |
| 1552486_s_at | 0.0019983 | LACTB |
| 226894_at | 0.0021225 | SLC35A3 |
| 212800_at | 0.0022471 | STX6 |
| 212950_at | 0.002336 | ADGRF5 |
| 218862_at | 0.0023481 | ASB13 |
| 232169_x_at | 0.0024746 | MIR7113///MIR4691///NDUFS8 |
| 224833_at | 0.0025158 | ETS1 |
| 209681_at | 0.0027094 | SLC19A2 |
| 225368_at | 0.0027644 | HIPK2 |
| 208078_s_at | 0.0027705 | SIK1 |
| 223085_at | 0.0029939 | RNF19A |
| 209870_s_at | 0.0031633 | APBA2 |
| 209276_s_at | 0.0032994 | GLRX |
| 205074_at | 0.0034285 | SLC22A5 |
| 219045_at | 0.0034398 | RHOF |
| 235165_at | 0.0037051 | PARD6B |
| 239246_at | 0.0037725 | FARP1 |
| 212097_at | 0.003953 | CAV1 |
| 212321_at | 0.0040433 | SGPL1 |
| 226939_at | 0.0041935 | CPEB2 |
| 222504_s_at | 0.0048611 | EMC8 |
| 204185_x_at | 0.0048945 | PPID |
| 202232_s_at | 0.0048969 | EIF3M |
| 218068_s_at | 0.0051257 | ZNF672 |
| 225001_at | 0.0051902 | RAB3D |
| 225612_s_at | 0.00546 | B3GNT5 |
| 212400_at | 0.0059089 | FAM102A |
| 228601_at | 0.0059989 | HAGLR |
| 222789_at | 0.0060234 | RSBN1 |
| 213238_at | 0.0062368 | ATP10D |
| 223026_s_at | 0.0064531 | VPS29 |
| 202948_at | 0.006639 | IL1R1 |
| 212294_at | 0.0066461 | GNG12 |
| 200835_s_at | 0.0066738 | MAP4 |
| 203635_at | 0.0067039 | DSCR3 |
| 210027_s_at | 0.0067355 | APEX1 |
| 244886_at | 0.0071642 | LOC389641 |
| 221510_s_at | 0.0072805 | GLS |
| 226475_at | 0.0073688 | FAM118A |
| 203387_s_at | 0.0073745 | TBC1D4 |
| 203066_at | 0.0075574 | CHST15 |
| 235046_at | 0.0075917 | INPP4B |
| 223218_s_at | 0.0076474 | NFKBIZ |
| 236918_s_at | 0.0077043 | LRRC34 |
| 232752_at | 0.0079574 | LOXL1-AS1 |
| 217992_s_at | 0.008194 | EFHD2 |
| 222621_at | 0.0085674 | DNAJC1 |
| 217764_s_at | 0.0087462 | RAB31 |
| 216250_s_at | 0.0089171 | LPXN |
| 211168_s_at | 0.0090181 | UPF1 |
| 217941_s_at | 0.0090569 | ERBIN |
| 241370_at | 0.0091715 | LOC286052 |
| 206414_s_at | 0.0092659 | ASAP2 |
| 229394_s_at | 0.0095247 | ARHGAP35 |
| 203403_s_at | 0.0095416 | RNF6 |
| 218217_at | 0.0096556 | SCPEP1 |
| 222565_s_at | 0.0096794 | PRKD3 |
| 218276_s_at | 0.0098277 | SAV1 |
| 207075_at | 0.0098716 | NLRP3 |
| 218415_at | 0.009941 | VPS33B |
| 228099_at | 0.0099591 | ZNF550 |
| 228491_at | 0.0101253 | KRT19 |
| 200782_at | 0.010131 | ANXA5 |
| 203620_s_at | 0.0102119 | FCHSD2 |
| 212711_at | 0.0102875 | CAMSAP1 |
| 201739_at | 0.0102903 | SGK1 |
| 231810_at | 0.0102981 | THRIL///BRI3BP |
| 212399_s_at | 0.0104645 | VGLL4 |
| 228181_at | 0.0104648 | SLC30A1 |
| 206245_s_at | 0.0107935 | IVNS1ABP |
| 226099_at | 0.0109027 | ELL2 |
| 231511_at | 0.0110367 | FRAS1 |
| 218849_s_at | 0.0110555 | PPP1R13L |
| 224692_at | 0.0110888 | PPP1R15B |
| 201872_s_at | 0.0112796 | ABCE1 |
| 223567_at | 0.0113223 | SEMA6B |
| 226534_at | 0.011621 | KITLG |
| 227475_at | 0.0116365 | FOXQ1 |
| 336_at | 0.0116907 | TBXA2R |
| 213927_at | 0.011784 | MAP3K9 |
| 201818_at | 0.0117919 | LPCAT1 |
| 204473_s_at | 0.0118955 | ZNF592 |
| 230051_at | 0.0119957 | PROSER2 |
| 207181_s_at | 0.0120004 | CASP7 |
| 230569_at | 0.0123162 | CFAP97 |
| 204823_at | 0.0124248 | NAV3 |
| 225564_at | 0.0124946 | SPATA13 |
| 1568611_at | 0.0125517 | P4HA2 |
| 221840_at | 0.0126265 | PTPRE |
| 210612_s_at | 0.0127367 | SYNJ2 |
| 226829_at | 0.0128129 | AFAP1L2 |
| 201110_s_at | 0.0130896 | THBS1 |
| 208946_s_at | 0.0131952 | BECN1 |
| 202449_s_at | 0.0131991 | RXRA |
| 203616_at | 0.0132017 | POLB |
| 205891_at | 0.0132948 | ADORA2B |
| 1553956_at | 0.0134537 | TMEM237 |
| 235399_at | 0.013462 | ERCC2 |
| 226954_at | 0.0135275 | UBE2R2 |
| 205725_at | 0.0135736 | SCGB1A1 |
| 221596_s_at | 0.0136864 | RBM48 |
| 217795_s_at | 0.013757 | TMEM43 |
| 217777_s_at | 0.0138813 | HACD3 |
| 202286_s_at | 0.0139016 | TACSTD2 |
| 208620_at | 0.0139311 | PCBP1 |
| 242455_at | 0.0143399 | POU3F2 |
| 219369_s_at | 0.0143572 | OTUB2 |
| 200911_s_at | 0.0144106 | TACC1 |
| 45288_at | 0.0144268 | ABHD6 |
| 204905_s_at | 0.0144407 | EEF1E1 |
| 227198_at | 0.0144924 | AFF3 |
| 227145_at | 0.0144973 | LOXL4 |
| 1566509_s_at | 0.014554 | FBXO9 |
| 223162_s_at | 0.0145871 | KIAA1147 |
| 226733_at | 0.0147001 | PFKFB2 |
| 213935_at | 0.0147338 | ABHD5 |
| 212099_at | 0.014939 | RHOB |
| 234985_at | 0.0149488 | LDLRAD3 |
| 205895_s_at | 0.015071 | NOLC1 |
| 209025_s_at | 0.0151937 | SYNCRIP |
| 202681_at | 0.0152229 | MIR4271///C3orf62///USP4 |
| 202553_s_at | 0.0153624 | SYF2 |
| 212538_at | 0.0154244 | DOCK9 |
| 203476_at | 0.0155583 | TPBG |
| 205364_at | 0.0155667 | ACOX2 |
| 201642_at | 0.0155667 | IFNGR2 |
| 201361_at | 0.0156189 | TMEM109 |
| 205463_s_at | 0.0157595 | PDGFA |
| 201349_at | 0.0158114 | SLC9A3R1 |
| 228340_at | 0.0160672 | TLE3 |
| 213328_at | 0.0162734 | NEK1 |
| 225464_at | 0.016285 | FRMD6 |
| 223401_at | 0.0164025 | ADPRM |
| 202670_at | 0.0164034 | MAP2K1 |
| 201278_at | 0.0165506 | LOC101926921///DAB2 |
| 222126_at | 0.0165718 | AGFG2 |
| 207760_s_at | 0.0165922 | NCOR2 |
| 212660_at | 0.0165939 | JADE2 |
| 213817_at | 0.0165952 | IRAK3 |
| 212134_at | 0.0166179 | MIR6716///PHLDB1 |
| 40284_at | 0.0167486 | FOXA2 |
| 220161_s_at | 0.0168145 | EPB41L4B |
| 212124_at | 0.0170546 | ZMIZ1 |
| 225167_at | 0.017058 | FRMD4A |
| 202886_s_at | 0.0170938 | PPP2R1B |
| 207196_s_at | 0.017113 | TNIP1 |
| 1557919_a_at | 0.0171206 | ERVH-3 |
| 209626_s_at | 0.0171517 | OSBPL3 |
| 201424_s_at | 0.0174886 | CUL4A |
| 228904_at | 0.0175389 | HOXB3 |
| 227020_at | 0.0176067 | YPEL2 |
| 217877_s_at | 0.0176681 | GPBP1L1 |
| 226748_at | 0.0178082 | LYSMD2 |
| 1556178_x_at | 0.0178317 | TAF8 |
| 218248_at | 0.0179781 | FAM111A |
| 211980_at | 0.0179931 | COL4A1 |
| 218463_s_at | 0.0181428 | MUS81 |
| 227813_at | 0.0181448 | THAP6 |
| 227577_at | 0.0181823 | EXOC8 |
| 217882_at | 0.0182693 | EMC3 |
| 224477_s_at | 0.0182829 | NUDT16L1 |
| 201160_s_at | 0.0183429 | YBX3 |
| 209272_at | 0.0184468 | NAB1 |
| 212727_at | 0.0184469 | DLG3 |
| 221561_at | 0.0184736 | SOAT1 |
| 226740_x_at | 0.0184981 | NBPF26///NBPF10///NBPF14 |
| 1554577_a_at | 0.0186115 | PSMD10 |
| 222835_at | 0.0187493 | THSD4 |
| 224416_s_at | 0.018819 | MED28 |
| 208029_s_at | 0.0188358 | LAPTM4B |
| 225982_at | 0.0188489 | UBTF |
| 226184_at | 0.0188507 | FMNL2 |
| 204576_s_at | 0.0188914 | CLUAP1 |
| 213111_at | 0.0190171 | PIKFYVE |
| 229074_at | 0.0192425 | EHD4 |
| 209745_at | 0.0195702 | COQ7 |
| 212208_at | 0.0196335 | MED13L |
| 225277_at | 0.019655 | SLC39A13 |
| 231807_at | 0.0196579 | KIAA1217 |
| 207168_s_at | 0.0197017 | H2AFY |
| 218656_s_at | 0.0197162 | LHFP |
| 230968_at | 0.0198852 | HDAC9 |
| 203789_s_at | 0.019958 | SEMA3C |
| 212506_at | 0.0200356 | PICALM |
| 226955_at | 0.0200491 | AFAP1L1 |
| 203202_at | 0.0202687 | KRR1 |
| 205060_at | 0.0202704 | PARG |
| 227904_at | 0.020292 | AZI2 |
| 235508_at | 0.0203521 | PML |
| 1554155_at | 0.0203618 | MCPH1 |
| 208690_s_at | 0.0204229 | PDLIM1 |
| 206183_s_at | 0.0204493 | LOC101929134///HERC3 |
| 203646_at | 0.0204625 | FDX1 |
| 204060_s_at | 0.0205374 | PRKY///PRKX |
| 212485_at | 0.0205551 | GPATCH8 |
| 200028_s_at | 0.0205751 | STARD7 |
| 235844_at | 0.0205926 | PHTF1 |
| 223168_at | 0.0206228 | RHOU |
| 235779_at | 0.0207543 | ZNF790-AS1 |
| 201675_at | 0.0209079 | AKAP1 |
| 226834_at | 0.0211036 | CLMP |
| 224747_at | 0.0212445 | UBE2Q2 |
| 208623_s_at | 0.0213239 | EZR |
| 225798_at | 0.0213275 | JAZF1 |
| 226571_s_at | 0.0213416 | PTPRS |
| 232597_x_at | 0.0213741 | SCAF11 |
| 204131_s_at | 0.0215252 | FOXO3 |
| 209457_at | 0.0215373 | DUSP5 |
| 202627_s_at | 0.0216285 | SERPINE1 |
| 220011_at | 0.0216699 | AUNIP |
| 243606_at | 0.021773 | NXPE3 |
| 226441_at | 0.0217742 | MAP3K2 |
| 203888_at | 0.0218861 | THBD |
| 212192_at | 0.0219239 | KCTD12 |
| 227584_at | 0.0220336 | NAV1 |
| 212158_at | 0.0223163 | SDC2 |
| 202431_s_at | 0.0223562 | MYC |
| 201310_s_at | 0.0224793 | NREP |
| 207005_s_at | 0.0226923 | BCL2 |
| 209890_at | 0.0227748 | TSPAN5 |
| 209357_at | 0.0228086 | CITED2 |
| 224609_at | 0.0229677 | SLC44A2 |
| 205202_at | 0.0229709 | PCMT1 |
| 209473_at | 0.0230402 | ENTPD1 |
| 215446_s_at | 0.0230771 | LOX |
| 221936_x_at | 0.0233608 | MRPL41 |
| 1560916_a_at | 0.0234577 | DPY19L1 |
| 209485_s_at | 0.0234635 | OSBPL1A |
| 218527_at | 0.0236339 | APTX |
| 227870_at | 0.0236927 | IGDCC4 |
| 227037_at | 0.0238536 | PLD6///FLCN |
| 228638_at | 0.0239081 | FAM76A |
| 211563_s_at | 0.0239105 | URI1 |
| 227526_at | 0.0239287 | CDON |
| 211962_s_at | 0.0239521 | ZFP36L1 |
| 223288_at | 0.0240131 | USP38 |
| 227960_s_at | 0.0240294 | FAHD1 |
| 210361_s_at | 0.0240332 | ELF2 |
| 200035_at | 0.0240817 | CTDNEP1 |
| 223118_s_at | 0.0241975 | USP47 |
| 1559942_at | 0.0243257 | MDFIC |
| 200015_s_at | 0.0244484 | SEPT2 |
| 231736_x_at | 0.0244669 | MGST1 |
| 222889_at | 0.0245225 | DCLRE1B |
| 202261_at | 0.0245452 | VPS72 |
| 210047_at | 0.0248085 | SLC11A2 |
| 244308_at | 0.0248168 | LOC101927699 |
| 214384_s_at | 0.0252027 | DCTN2 |
| 200697_at | 0.0253886 | HK1 |
| 225626_at | 0.0254753 | PAG1 |
| 1557609_s_at | 0.0255915 | TBC1D12 |
| 201034_at | 0.0256794 | ADD3 |
| 220329_s_at | 0.0258027 | RMND1 |
| 204892_x_at | 0.0258172 | EEF1A1 |
| 201980_s_at | 0.0258339 | RSU1 |
| 236321_at | 0.0258513 | FAM200B |
| 213329_at | 0.025952 | SRGAP2C///SRGAP2B///SRGAP2 |
| 209688_s_at | 0.0261135 | CCDC93 |
| 202686_s_at | 0.0261409 | AXL |
| 209804_at | 0.0262003 | DCLRE1A |
| 226076_s_at | 0.0262287 | MBD6 |
| 208184_s_at | 0.0266062 | LOC102724200///TRAPPC10 |
| 219940_s_at | 0.0266406 | PCID2 |
| 201648_at | 0.0269142 | JAK1 |
| 214439_x_at | 0.0269627 | BIN1 |
| 220720_x_at | 0.0270616 | MZT2B |
| 203395_s_at | 0.0276084 | HES1 |
| 226823_at | 0.0277794 | PHACTR4 |
| 227936_at | 0.0277916 | TMEM68 |
| 211300_s_at | 0.0278865 | TP53 |
| 228495_at | 0.0281955 | GPATCH11 |
| 228066_at | 0.0282378 | C17orf96 |
| 206056_x_at | 0.0282435 | SPN |
| 1554830_a_at | 0.0284342 | STEAP3 |
| 203585_at | 0.0284729 | ZNF185 |
| 210101_x_at | 0.0285339 | SH3GLB1 |
| 209033_s_at | 0.028688 | DYRK1A |
| 223945_x_at | 0.0287319 | RP9P |
| 224570_s_at | 0.0289092 | IRF2BP2 |
| 235182_at | 0.0289293 | ISM1 |
| 201984_s_at | 0.0291254 | EGFR |
| 206669_at | 0.0292795 | GAD1 |
| 214109_at | 0.0293872 | LRBA |
| 208763_s_at | 0.0294189 | TSC22D3 |
| 204319_s_at | 0.029438 | RGS10 |
| 203044_at | 0.0294541 | CHSY1 |
| 223204_at | 0.0294911 | FAM198B |
| 219491_at | 0.0296484 | LRFN4 |
| 222592_s_at | 0.0296821 | ACSL5 |
| 202174_s_at | 0.0296831 | PCM1 |
| 213340_s_at | 0.0296876 | TP73-AS1 |
| 228652_at | 0.0297083 | ZNF776 |
| 223108_s_at | 0.0297323 | ZCCHC17 |
| 230820_at | 0.0297332 | SMURF2 |
| 227357_at | 0.0297535 | TAB3 |
| 226609_at | 0.0300118 | DCBLD1 |
| 205805_s_at | 0.0301568 | ROR1 |
| 229221_at | 0.0301934 | CD44 |
| 224858_at | 0.0303044 | ZDHHC5 |
| 1554078_s_at | 0.0305117 | DNAJA3 |
| 225943_at | 0.0306477 | NLN |
| 202862_at | 0.0306658 | FAH |
| 209939_x_at | 0.0306746 | CFLAR |
| 201325_s_at | 0.0307342 | EMP1 |
| 222430_s_at | 0.0307855 | YTHDF2 |
| 243907_at | 0.0307899 | GREB1L |
| 226565_at | 0.0307981 | TMEM99 |
| 217997_at | 0.0308703 | PHLDA1 |
| 1558942_at | 0.0310013 | ZNF765 |
| 201883_s_at | 0.0313539 | B4GALT1 |
| 218313_s_at | 0.0313608 | GALNT7 |
| 202027_at | 0.0315218 | TMEM184B |
| 231779_at | 0.0315462 | IRAK2 |
| 225813_at | 0.0316789 | RC3H2 |
| 222268_x_at | 0.0317027 | MUC5B |
| 203320_at | 0.0317521 | SH2B3 |
| 221870_at | 0.0317551 | EHD2 |
| 205432_at | 0.0319392 | OVGP1 |
| 222696_at | 0.031948 | AXIN2 |
| 200768_s_at | 0.0319599 | MAT2A |
| 201938_at | 0.0321068 | CDK2AP1 |
| 203712_at | 0.032307 | PUM3 |
| 226031_at | 0.0324246 | VPS50 |
| 202641_at | 0.0325788 | ARL3 |
| 229102_at | 0.0327025 | HTATIP2 |
| 203544_s_at | 0.0327166 | STAM |
| 209345_s_at | 0.0327825 | PI4K2A |
| 1554251_at | 0.0329065 | HP1BP3 |
| 209262_s_at | 0.03298 | NR2F6 |
| 213069_at | 0.0330006 | HEG1 |
| 200924_s_at | 0.0331163 | SLC3A2 |
| 219735_s_at | 0.033274 | TFCP2L1 |
| 228520_s_at | 0.0332759 | APLP2 |
| 238430_x_at | 0.0333373 | SLFN5 |
| 229759_s_at | 0.0333703 | VEPH1 |
| 212560_at | 0.0334388 | SORL1 |
| 202526_at | 0.0334753 | SMAD4 |
| 203306_s_at | 0.0336219 | SLC35A1 |
| 225136_at | 0.0336266 | PLEKHA2 |
| 235585_at | 0.0337191 | DR1 |
| 212184_s_at | 0.0337462 | TAB2 |
| 244052_at | 0.0338035 | CBR4 |
| 201183_s_at | 0.0338858 | CHD4 |
| 226036_x_at | 0.0339553 | CASP2 |
| 209365_s_at | 0.0339607 | ECM1 |
| 209481_at | 0.0339904 | SNRK |
| 212149_at | 0.0339987 | EFR3A |
| 202912_at | 0.0340327 | ADM |
| 226512_at | 0.0341359 | ZMYM2 |
| 207090_x_at | 0.0341735 | ZFP30 |
| 203275_at | 0.0342675 | IRF2 |
| 227236_at | 0.0343031 | TSPAN2 |
| 238761_at | 0.0344758 | ELK4 |
| 200753_x_at | 0.0345239 | MIR636///SRSF2 |
| 203373_at | 0.0345569 | SOCS2 |
| 203327_at | 0.0345807 | IDE |
| 213811_x_at | 0.0346227 | TCF3 |
| 203060_s_at | 0.0346857 | PAPSS2 |
| 225202_at | 0.0347167 | RHOBTB3 |
| 218968_s_at | 0.0349741 | ZFP64 |
| 201798_s_at | 0.0350972 | MYOF |
| 201924_at | 0.0351027 | AFF1 |
| 225267_at | 0.0351051 | KPNA4 |
| 231564_at | 0.035194 | MANEAL |
| 201366_at | 0.0352352 | ANXA7 |
| 214585_s_at | 0.0354716 | VPS52 |
| 1555272_at | 0.0354885 | RSPH10B2///RSPH10B |
| 218543_s_at | 0.0355428 | PARP12 |
| 203508_at | 0.0355729 | TNFRSF1B |
| 225530_at | 0.0358025 | MOB3A |
| 201092_at | 0.0358558 | RBBP7 |
| 223887_at | 0.0358786 | GPR132 |
| 212006_at | 0.0359028 | UBXN4 |
| 64488_at | 0.03593 | IRGQ |
| 203679_at | 0.0360728 | TMED1 |
| 1555780_a_at | 0.036084 | RHEB |
| 201905_s_at | 0.0361816 | CTDSPL |
| 201740_at | 0.0362924 | NDUFS3 |
| 224882_at | 0.0363548 | ACSS1 |
| 211997_x_at | 0.0364024 | MIR4738///H3F3B///H3F3A |
| 200059_s_at | 0.0366922 | RHOA |
| 222605_at | 0.0368174 | RCOR3 |
| 203636_at | 0.0368193 | MID1 |
| 239512_at | 0.0369219 | SRSF4 |
| 236302_at | 0.0369243 | PPM1E |
| 205327_s_at | 0.0372166 | ACVR2A |
| 226810_at | 0.0373498 | OGFRL1 |
| 218214_at | 0.037442 | ATG101 |
| 240144_at | 0.0375064 | DNASE1 |
| 205260_s_at | 0.0375523 | ACYP1 |
| 211686_s_at | 0.0375888 | MAK16 |
| 226320_at | 0.0376226 | ALYREF |
| 200762_at | 0.0376389 | DPYSL2 |
| 208897_s_at | 0.0377052 | DDX18 |
| 224873_s_at | 0.0377139 | MRPS25 |
| 201042_at | 0.0378059 | TGM2 |
| 1556346_at | 0.0378314 | COTL1 |
| 214769_at | 0.0379297 | CLCN4 |
| 226449_at | 0.0380223 | CEP120 |
| 214771_x_at | 0.0380403 | MPRIP |
| 225618_at | 0.0380479 | ARHGAP27 |
| 213629_x_at | 0.0380651 | MT1F |
| 215017_s_at | 0.0381722 | FNBP1L |
| 231067_s_at | 0.0381732 | AKAP12 |
| 200804_at | 0.0382197 | TMBIM6 |
| 200760_s_at | 0.0382453 | ARL6IP5 |
| 202368_s_at | 0.0382592 | TRAM2 |
| 213425_at | 0.0382953 | WNT5A |
| 232266_x_at | 0.0384561 | CDK13 |
| 227771_at | 0.0385146 | LIFR |
| 200746_s_at | 0.0385987 | GNB1 |
| 223151_at | 0.0386013 | DCUN1D5 |
| 242120_at | 0.0386807 | USP49 |
| 203867_s_at | 0.0386817 | NLE1 |
| 205068_s_at | 0.038722 | ARHGAP26 |
| 221211_s_at | 0.0387536 | MAP3K7CL |
| 201041_s_at | 0.0387855 | DUSP1 |
| 213075_at | 0.038786 | OLFML2A |
| 203624_at | 0.0387954 | AKAP17A |
| 208305_at | 0.0388891 | PGR |
| 224838_at | 0.038902 | FOXP1 |
| 201376_s_at | 0.0389178 | HNRNPF |
| 213698_at | 0.0389348 | TMEM35B///ZMYM6 |
| 224959_at | 0.0392107 | SLC26A2 |
| 221702_s_at | 0.0392743 | TM2D3 |
| 213419_at | 0.0393424 | APBB2 |
| 218379_at | 0.0393742 | RBM7 |
| 213182_x_at | 0.0393793 | CDKN1C |
| 226814_at | 0.0394014 | ADAMTS9 |
| 209661_at | 0.0394069 | KIFC3 |
| 224542_s_at | 0.0394719 | NFATC2 |
| 207941_s_at | 0.039718 | RBM39 |
| 200764_s_at | 0.0397342 | CTNNA1 |
| 202131_s_at | 0.0398322 | RIOK3 |
| 1553274_a_at | 0.0398481 | SNRNP48 |
| 202583_s_at | 0.0398741 | RANBP9 |
| 218107_at | 0.0401569 | WDR26 |
| 225217_s_at | 0.0405881 | BRPF3 |
| 213012_at | 0.0405997 | NEDD4 |
| 208949_s_at | 0.0406238 | LGALS3 |
| 207345_at | 0.0407467 | FST |
| 213338_at | 0.0407575 | TMEM158 |
| 241866_at | 0.0408484 | SLC16A7 |
| 202260_s_at | 0.0408611 | STXBP1 |
| 217922_at | 0.0409026 | MAN1A2 |
| 218035_s_at | 0.040909 | RBM47 |
| 200758_s_at | 0.0409287 | NFE2L1 |
| 220952_s_at | 0.0410474 | PLEKHA5 |
| 227167_s_at | 0.0410911 | RASSF3 |
| 209862_s_at | 0.0412442 | CEP57 |
| 222001_x_at | 0.0412752 | LINC00623///LINC00869 |
| 207037_at | 0.0412828 | TNFRSF11A |
| 204388_s_at | 0.0413391 | MAOA |
| 214155_s_at | 0.0414131 | LARP4 |
| 218777_at | 0.0414616 | REEP4 |
| 209225_x_at | 0.0414737 | TNPO1 |
| 218067_s_at | 0.0416036 | ARGLU1 |
| 200906_s_at | 0.0416295 | PALLD |
| 208891_at | 0.0416664 | DUSP6 |
| 224790_at | 0.0416805 | ASAP1 |
| 225069_at | 0.0417143 | PCYT1A |
| 233559_s_at | 0.0417785 | WDFY1 |
| 233748_x_at | 0.0420476 | PRKAG2 |
| 203556_at | 0.0422344 | ZHX2 |
| 203100_s_at | 0.0423867 | CDYL |
| 224280_s_at | 0.0425492 | MTFR1L |
| 237032_x_at | 0.042645 | SIPA1L1 |
| 203786_s_at | 0.0426905 | TPD52L1 |
| 1558279_a_at | 0.0428465 | KDSR |
| 217923_at | 0.0428827 | PEF1 |
| 231183_s_at | 0.0429687 | JAG1 |
| 210512_s_at | 0.0430598 | VEGFA |
| 205995_x_at | 0.043067 | IQCB1 |
| 219274_at | 0.0430767 | TSPAN12 |
| 226935_s_at | 0.0430945 | CLPTM1L |
| 213595_s_at | 0.043235 | CDC42BPA |
| 226004_at | 0.0432474 | CABLES2 |
| 200655_s_at | 0.0433109 | CALM3///CALM2///CALM1 |
| 226040_at | 0.0433242 | TRIM56 |
| 202440_s_at | 0.0433501 | ST5 |
| 1557094_at | 0.0435249 | LOC100996760 |
| 223158_s_at | 0.0435827 | NEK6 |
| 204695_at | 0.0437102 | CDC25A |
| 228908_s_at | 0.0438418 | LOC642852 |
| 217257_at | 0.0439414 | SH3BP2 |
| 217513_at | 0.0439426 | MILR1 |
| 217890_s_at | 0.0440466 | PARVA |
| 211474_s_at | 0.0440602 | SERPINB6 |
| 227107_at | 0.0441898 | PANX1 |
| 236160_at | 0.044212 | TRIP11 |
| 225199_at | 0.0442364 | C16orf72 |
| 210249_s_at | 0.0442492 | NCOA1 |
| 212612_at | 0.0444581 | RCOR1 |
| 206170_at | 0.0444742 | ADRB2 |
| 226866_at | 0.044572 | ESCO1 |
| 201761_at | 0.0446806 | MTHFD2 |
| 208718_at | 0.0448909 | DDX17 |
| 204440_at | 0.0449142 | CD83 |
| 218582_at | 0.0449187 | 43164 |
| 220655_at | 0.0450777 | TNIP3 |
| 203549_s_at | 0.0451004 | LPL |
| 222802_at | 0.0451199 | EDN1 |
| 244461_at | 0.0451785 | SPECC1 |
| 209723_at | 0.0451841 | SERPINB9 |
| 212533_at | 0.0452749 | WEE1 |
| 225117_at | 0.0453795 | KANSL1 |
| 201348_at | 0.0454222 | GPX3 |
| 1552626_a_at | 0.0455045 | TMEM163 |
| 204500_s_at | 0.0455278 | AGTPBP1 |
| 204337_at | 0.0455639 | RGS4 |
| 225532_at | 0.0455915 | CABLES1 |
| 229509_at | 0.0456019 | MFSD8 |
| 226333_at | 0.0456317 | IL6R |
| 222618_at | 0.0456953 | SMU1 |
| 212327_at | 0.0457767 | LIMCH1 |
| 1558964_at | 0.0458807 | FAT3 |
| 228496_s_at | 0.0459185 | CRIM1 |
| 212983_at | 0.0459772 | HRAS |
| 201651_s_at | 0.0459801 | PACSIN2 |
| 201751_at | 0.0460272 | JOSD1 |
| 225164_s_at | 0.0460306 | EIF2AK4 |
| 225390_s_at | 0.0460603 | KLF13 |
| 223301_s_at | 0.0460765 | CCDC82 |
| 229228_at | 0.0460867 | LOC401317///CREB5 |
| 204269_at | 0.0462059 | PIM2 |
| 213572_s_at | 0.0463442 | SERPINB1 |
| 218486_at | 0.0463829 | KLF11 |
| 234928_x_at | 0.0463844 | RUNX3 |
| 200766_at | 0.0464328 | CTSD |
| 219549_s_at | 0.046525 | RTN3 |
| 225450_at | 0.0465324 | AMOTL1 |
| 224624_at | 0.0465398 | LRRC8A |
| 208848_at | 0.0465568 | ADH5 |
| 201464_x_at | 0.0468561 | JUN |
| 201783_s_at | 0.0468936 | RELA |
| 225517_at | 0.0469313 | ZNF770 |
| 223647_x_at | 0.0469401 | HSCB |
| 216384_x_at | 0.047131 | LOC100506248 |
| 239777_at | 0.0471975 | LINC01588 |
| 1552264_a_at | 0.0473165 | MAPK1 |
| 208091_s_at | 0.047528 | VOPP1 |
| 218511_s_at | 0.0475361 | PNPO |
| 218853_s_at | 0.0475477 | MOSPD1 |
| 203277_at | 0.0475551 | DFFA |
| 203221_at | 0.0475703 | TLE1 |
| 236314_at | 0.0476547 | EPM2AIP1 |
| 226657_at | 0.0478614 | NATD1 |
| 211578_s_at | 0.0479827 | RPS6KB1 |
| 235675_at | 0.0480289 | DHFR2 |
| 226722_at | 0.0481014 | FAM20C |
| 225583_at | 0.0481547 | UXS1 |
| 203379_at | 0.0481708 | RPS6KA1 |
| 220987_s_at | 0.0483438 | NUAK2///AKIP1 |
| 237485_at | 0.0484246 | SRSF3 |
| 209362_at | 0.0486576 | LOC101928625///MED21 |
| 242543_at | 0.0488473 | SH2D6 |
| 221657_s_at | 0.0488524 | ASB6 |
| 205421_at | 0.0489184 | SLC22A3 |
| 218578_at | 0.0490484 | CDC73 |
| 209388_at | 0.04928 | PAPOLA |
| 209026_x_at | 0.0493692 | TUBB |
| 218694_at | 0.0494002 | ARMCX1 |
| 222212_s_at | 0.0495517 | CERS2 |
| 1553348_a_at | 0.0495701 | NFX1 |
| 227491_at | 0.0496664 | ELOVL6 |
| 202949_s_at | 0.0496671 | FHL2 |
| 210749_x_at | 0.0496827 | MIR4640///DDR1 |
| 209189_at | 0.0499037 | FOS |
| 235498_at | 0.0499386 | LRRIQ3 |
| 212196_at | 0.0499912 | IL6ST |

| **Supplementary** **Table 7.** Combined analysis of CLPTM1L-modulated genes, IR-related genes, and ERE containing genes |
| --- |
| CLPTM1L Merged ERE genes |
| ATP2B4 |
| BCL2 |
| BIN1 |
| CASP2 |
| CD44 |
| CDC25A |
| CDC42BPA |
| CLPTM1L |
| DR1 |
| EGFR |
| FOS |
| HRAS |
| JAK1 |
| JUN |
| MAPK1 |
| MTFR1L |
| MYC |
| NOLC1 |
| P4HA2 |
| PGR |
| TLE3 |
| TP53 |
| VEGFA |
| ZDHHC5 |
| CLPTM1L Merged Radiation |
| ABCB4 |
| ATP2B4 |
| BCL2 |
| BRCA1 |
| BTAF1 |
| CCNT1 |
| CD14 |
| CD44 |
| CDC25A |
| CDC42BPA |
| CLPTM1L |
| EGFR |
| FOS |
| HRAS |
| JAK1 |
| JUN |
| MAPK1 |
| MYC |
| NUDT1 |
| PGR |
| RPL27 |
| TP53 |
| TRIO |
| VEGFA |
| Irradiation Merged ERE genes |
| ATP2B4 |
| BCL2 |
| CD44 |
| CDC25A |
| CDC42BPA |
| CLPTM1L |
| COL4A1 |
| CTNNA1 |
| CTSD |
| DDX17 |
| EGFR |
| ELF2 |
| ELK4 |
| ELL2 |
| ENTPD1 |
| FOS |
| FST |
| HRAS |
| IDE |
| IL6ST |
| JAK1 |
| JUN |
| MAN1A2 |
| MAPK1 |
| MDFIC |
| MYC |
| NEDD4 |
| NEK1 |
| PCM1 |
| PGR |
| PML |
| RGS4 |
| RHOB |
| RNF6 |
| SDC2 |
| SLC26A2 |
| STX6 |
| TBXA2R |
| TGM2 |
| THBD |
| THBS1 |
| TNPO1 |
| TP53 |
| TRIP11 |
| VEGFA |
| WEE1 |
| Triple Merged |
| ATP2B4 |
| BCL2 |
| CD44 |
| CDC25A |
| CDC42BPA |
| CLPTM1L |
| EGFR |
| FOS |
| HRAS |
| JAK1 |
| JUN |
| MAPK1 |
| MYC |
| PGR |
| TP53 |
| VEGFA |

**Supplementary** **Table 8.** Cross tabulation analysis of CLPTM1L and CDC25A in NSCLC tissues

| NSCLC | |  | CDC25A | | | Total |
| --- | --- | --- | --- | --- | --- | --- |
|  |  |  | - | + | ++ |  |
| CLPTM1L | - | Count | 16 | 1 | 2 | 19 |
|  |  | % within CDC25A | 84.2 | 5.3 | 10.5 | 100 |
|  | + | Count | 3 | 44 | 5 | 52 |
|  |  | % within CDC25A | 5.8 | 84.6 | 9.6 | 100 |
|  | ++ | Count | 0 | 9 | 30 | 39 |
|  |  | % within CDC25A | 0 | 23.1 | 76.9 | 100 |
| Total | | Count | 19 | 54 | 37 | 110 |
|  | | % within CDC25A | 17.3 | 49.1 | 33.6 | 100 |

χ^2^=81.217, *P*<0.001, n=110

**Supplementary** **Table 9.** Cross tabulation analysis of CLPTM1L and c-Jun in NSCLC tissues

| NSCLC | |  | c-Jun | | | Total |
| --- | --- | --- | --- | --- | --- | --- |
|  |  |  | - | + | ++ |  |
| CLPTM1L | - | Count | 14 | 4 | 1 | 19 |
|  |  | % within c-Jun | 73.7 | 21.0 | 5.3 | 100 |
|  | + | Count | 6 | 42 | 4 | 52 |
|  |  | % within c-Jun | 11.5 | 80.8 | 7.7 | 100 |
|  | ++ | Count | 1 | 9 | 29 | 39 |
|  |  | % within c-Jun | 2.6 | 23.1 | 74.3 | 100 |
| Total | | Count | 21 | 55 | 34 | 110 |
|  | | % within c-Jun | 19.1 | 50.0 | 30.9 | 100 |

χ^2^=74.326, *P*<0.001, n=110

**Supplementary** **Table 10.** Cross tabulation analysis of CLPTM1L and BCL2 in NSCLC tissues

| NSCLC | |  | BCL2 | | | Total |
| --- | --- | --- | --- | --- | --- | --- |
|  |  |  | - | + | ++ |  |
| CLPTM1L | - | Count | 15 | 4 | 0 | 19 |
|  |  | % within BCL2 | 78.9 | 21.1 | 0 | 100 |
|  | + | Count | 6 | 37 | 9 | 52 |
|  |  | % within BCL2 | 11.5 | 71.2 | 17.3 | 100 |
|  | ++ | Count | 2 | 11 | 26 | 39 |
|  |  | % within BCL2 | 5.1 | 28.2 | 66.7 | 100 |
| Total | | Count | 23 | 52 | 35 | 110 |
|  | | % within BCL2 | 20.9 | 47.3 | 31.8 | 100 |

χ^2^=71.693, *P*<0.001, n=110

**
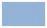

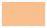

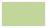

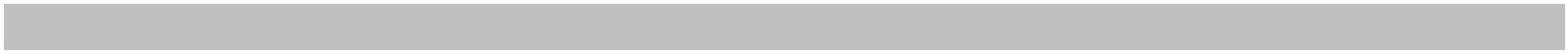

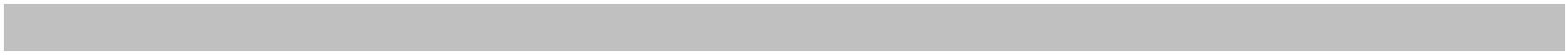

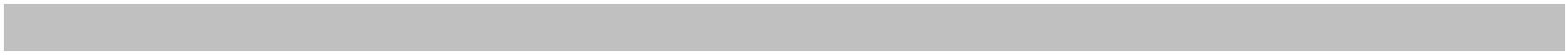

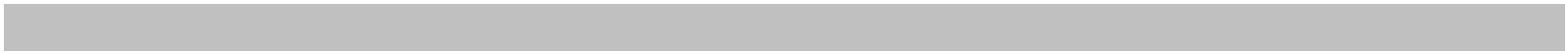

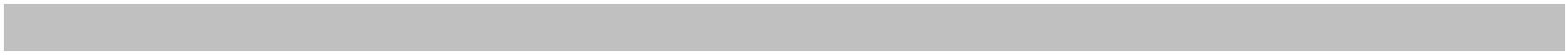

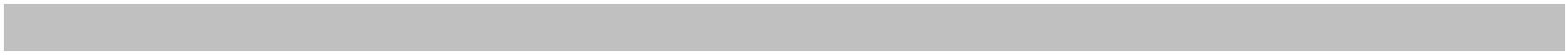

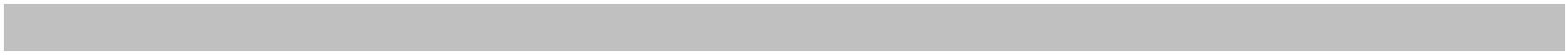

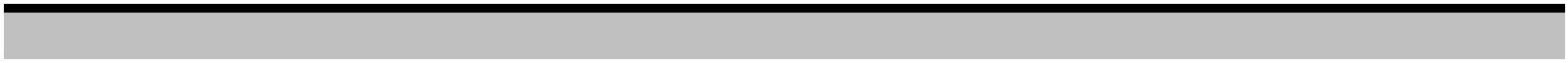

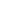

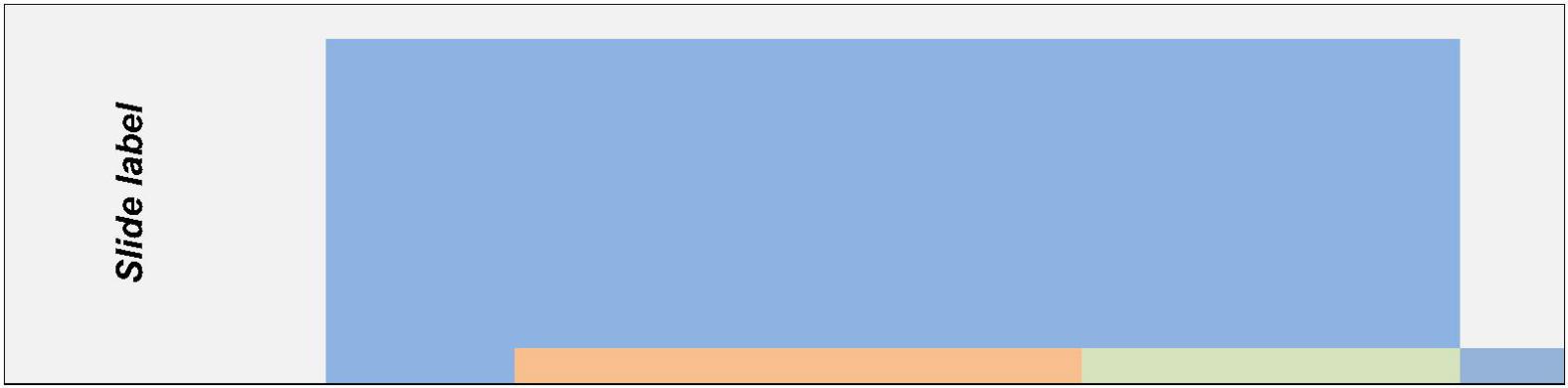

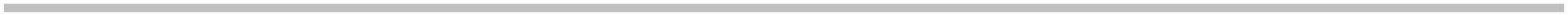

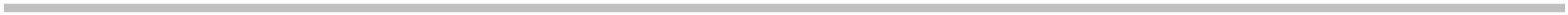

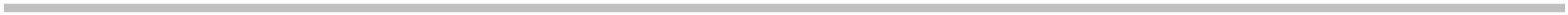

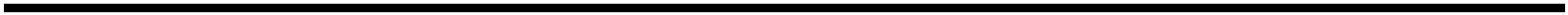
**

**Supplementary** **Table 11.** Non-small cell lung carcinoma & Normal TMA

Catalog #: TC0219

Availability: By order

|  | Description: |  | Human non-small cell lung carcinoma tissue microarray, containing 20 cases of  lung squamous cell carcinoma, 37 cases of lung large cell carcinoma, 53 cases of  lung adenocarcinoma, 10 cases of adjacent normal lung tissue or normal lung  tissue. |
| --- | --- | --- | --- |

Species Human

Fixative: Formalin

Total cases 120

Total cores: 120

Layout: 10×12

Core size: 1.0 mm

Section thickness: 3-5 μm

Validation: Validated for Immunohistochemistry and In Situ Hybridization.

A B C D E F G H I J K L M

1 Lun Lun Lun Lun Lun Lun Lun Lun Lun Lun Lun Lun

2 Lun Lun Lun Lun Lun Lun Lun Lun Lun Lun Lun Lun

3 Lun Lun Lun Lun Lun Lun Lun Lun Lun Lun Lun Lun

4 Lun Lun Lun Lun Lun Lun Lun Lun Lun Lun Lun Lun

5 Lun Lun Lun Lun Lun Lun Lun Lun Lun Lun Lun Lun

6 Lun Lun Lun Lun Lun Lun Lun Lun Lun Lun Lun Lun

7 Lun Lun Lun Lun Lun Lun Lun Lun Lun Lun Lun Lun

8 Lun Lun Lun Lun Lun Lun Lun Lun Lun Lun Lun Lun

9 Lun Lun Lun Lun Lun Lun Lun Lun Lun Lun Lun Lun

10 Lun Lun Lun Lun Lun Lun Lun Lun Lun Lun Lun Lun Mark


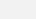
Malignant tumor Normal adjacent tissue Normal tissue

**# Pos Sex Age Tissue Pathological Diagnosis Grade Stage TNM**

1 1A F 61 Lung Squamous cell carcinoma 3 IIIA T3N1M0

2 1B M 65 Lung Squamous cell carcinoma 3 IIIA T2N2M0

3 1C M 64 Lung Squamous cell carcinoma 3 IIIA T2N2M0

4 1D M 62 Lung Squamous cell carcinoma with necrosis – IIIA T3N1M0

5 1E F 65 Lung Squamous cell carcinoma 3 IIIA T2N2M0

6 1F M 67 Lung Squamous cell carcinoma 3 IIIA T2N2M0

7 1G M 61 Lung Squamous cell carcinoma 3 IIIB T4N0M0

8 1H M 68 Lung Squamous cell carcinoma 3 IIIA T2N2M0

9 1I M 70 Lung Squamous cell carcinoma 3 IIIA T2N2M0

10 1J M 63 Lung Squamous cell carcinoma 3 IIIA T2N2M0

11 1K M 53 Lung Squamous cell carcinoma 3 IIIA T3N2M0

12 1L M 72 Lung Squamous cell carcinoma 3 IIIB T4N2M0

13 2A M 74 Lung Squamous cell carcinoma 3 IIIB T4N1M0

14 2B F 54 Lung Squamous cell carcinoma 2 IIIB T4N0M0

15 2C M 55 Lung Squamous cell carcinoma 3 IIIB T4N0M0


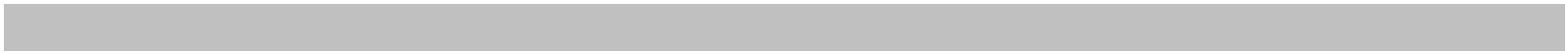

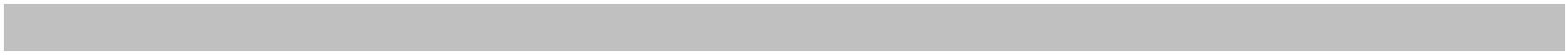

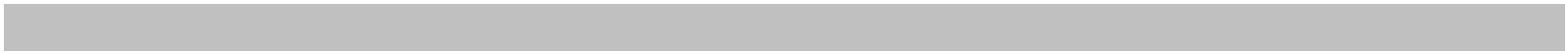

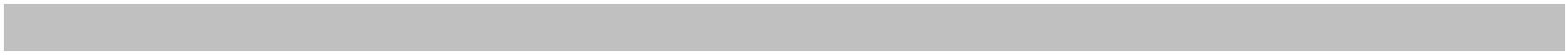

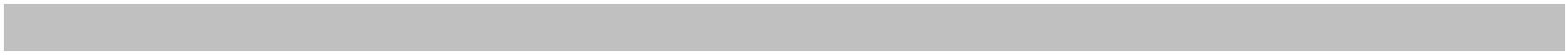

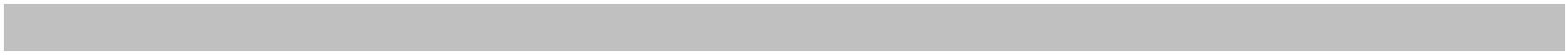

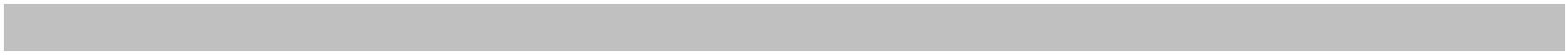

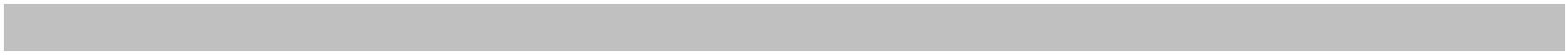

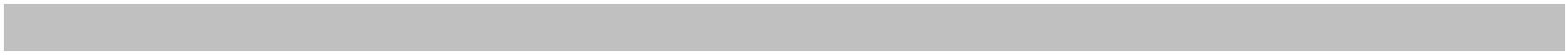

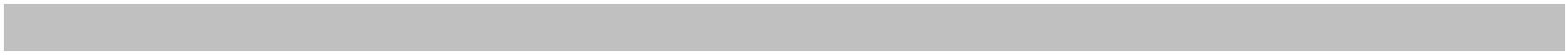

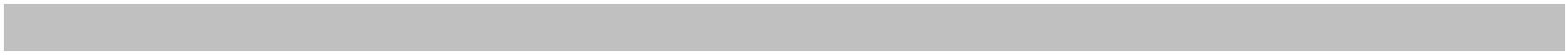

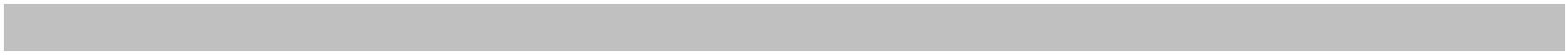

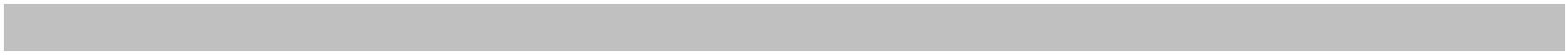

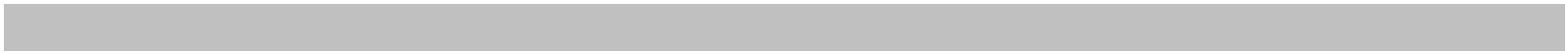

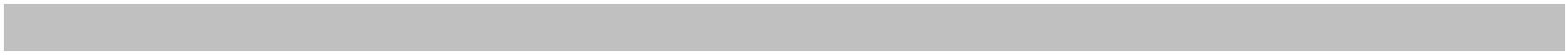

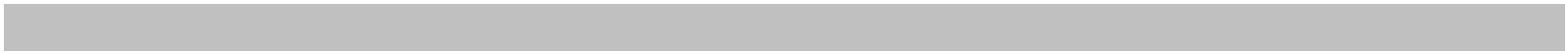

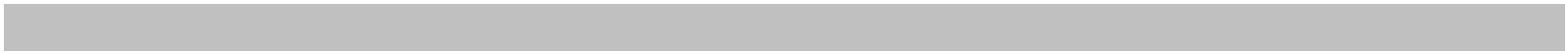

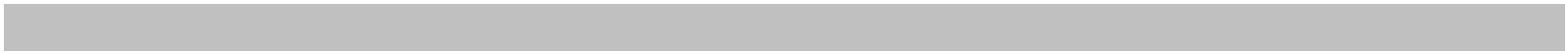

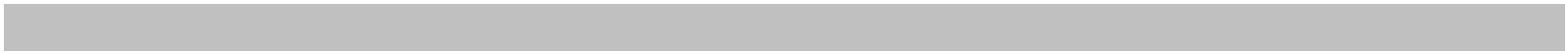

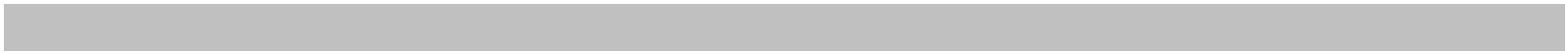

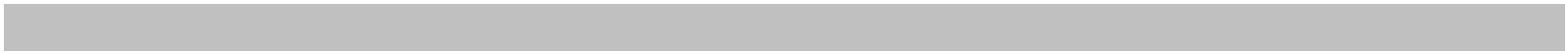

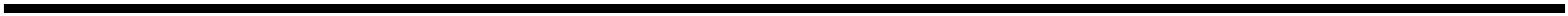


**# Pos Sex Age Tissue Pathological Diagnosis Grade Stage TNM**

16 2D M 62 Lung Squamous cell carcinoma 3 IIIA T3N2M0

17 2E M 48 Lung Squamous cell carcinoma 3 IIIA T2N0M0

18 2F M 63 Lung Squamous cell carcinoma – IIIB T4N0M0

19 2G M 44 Lung Squamous cell carcinoma 3 IIIA T3N1M0

20 2H F 60 Lung Squamous cell carcinoma 3 IIIA T2N2M0

21 2I F 54 Lung Large cell carcinoma – IIB T2N1M0

22 2J F 72 Lung Large cell carcinoma – IIB T3N0M0

23 2K F 48 Lung Large cell carcinoma – IB T2N0M0

24 2L M 55 Lung Large cell carcinoma – IB T2N0M0

25 3A M 45 Lung Large cell carcinoma – I T1N0M0

26 3B M 63 Lung Large cell carcinoma – IIIB T4N0M0

27 3C M 64 Lung Large cell carcinoma – IB T2N0M0

28 3D M 61 Lung Large cell carcinoma – IB T2N0M0

29 3E M 64 Lung Large cell carcinoma – IIIB T4N0M0

30 3F M 33 Lung Large cell carcinoma – IB T2N0M0

31 3G M 58 Lung Large cell carcinoma – IIB T2N1M0

32 3H M 62 Lung Large cell carcinoma – IB T2N0M0

33 3I M 62 Lung Large cell carcinoma with necrosis – IIIA T3N2M0

34 3J M 43 Lung Large cell carcinoma – IB T2N0M0

35 3K M 62 Lung Large cell carcinoma – IIB T2N1M0

36 3L M 51 Lung Large cell carcinoma – IIB T3N0M0

37 4A F 68 Lung Large cell carcinoma – IIB T2N1M0

38 4B M 64 Lung Large cell carcinoma – IB T2N0M0

39 4C F 45 Lung Large cell carcinoma – IB T2N0M0

40 4D M 48 Lung Large cell carcinoma – IA T1N0M0

41 4E F 66 Lung Large cell carcinoma with necrosis – IB T2N0M0

42 4F F 65 Lung Large cell carcinoma with necrosis – I T2N0M0

43 4G F 59 Lung Large cell carcinoma – IB T2N0M0

44 4H M 64 Lung Large cell carcinoma – IB T2N0M0

45 4I M 57 Lung Large cell carcinoma – IB T2N0M0

46 4J M 42 Lung Large cell carcinoma – IB T2N0M0

47 4K M 44 Lung Large cell carcinoma – IB T2N0M0

48 4L M 70 Lung Large cell carcinoma – IIIA T3N1M0

49 5A M 55 Lung Large cell carcinoma – IIB T2N1M0

50 5B M 56 Lung Large cell carcinoma with necrosis – IIB T2N1M0

51 5C M 53 Lung Large cell carcinoma – IB T2N0M0

52 5D M 62 Lung Large cell carcinoma – I T2N0M0

53 5E F 30 Lung Large cell carcinoma – IIB T2N1M0

54 5F F 49 Lung Large cell carcinoma – IIIA T2N2M0

55 5G M 71 Lung Large cell carcinoma – IIIA T3N1M0

56 5H M 54 Lung Large cell carcinoma – IIIB T4N0M0

57 5I M 64 Lung Adenocarcinoma 3 IIIA T2N2M0

58 5J M 51 Lung Adenocarcinoma 2 IB T2N0M0


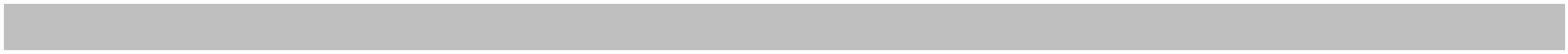

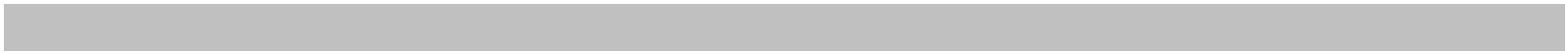

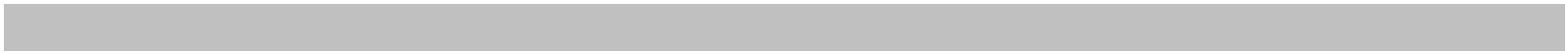

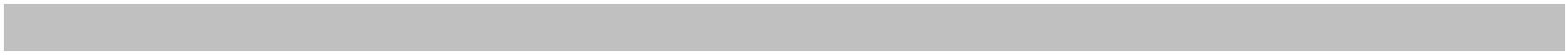

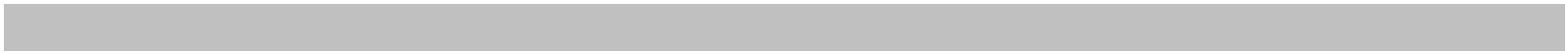

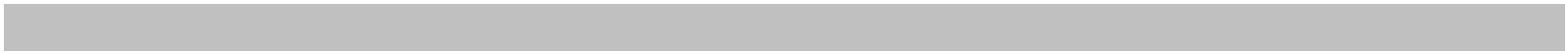

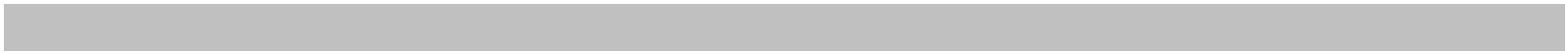

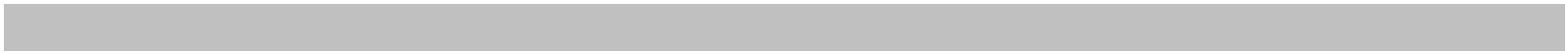

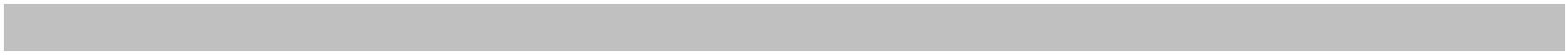

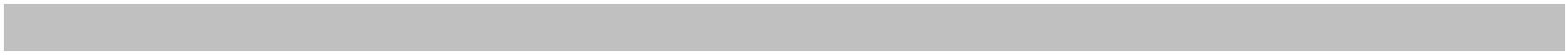

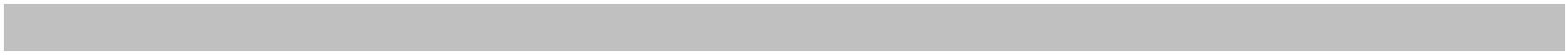

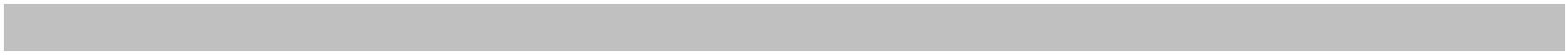

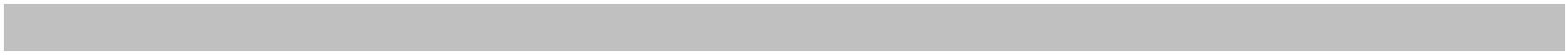

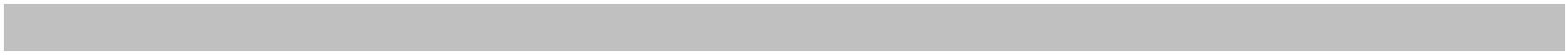

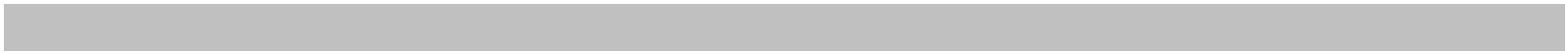

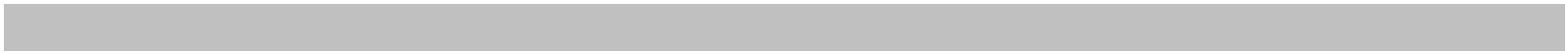

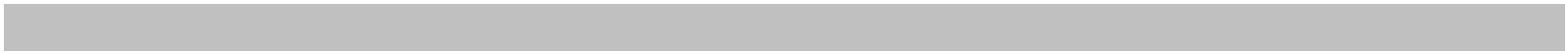

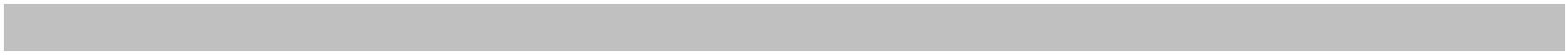

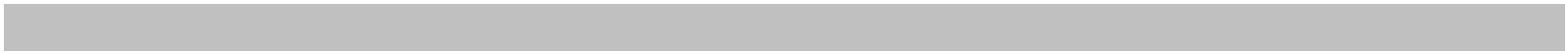

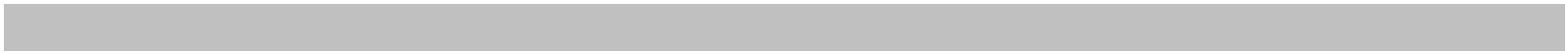

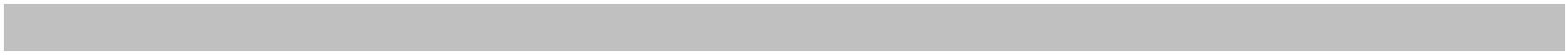

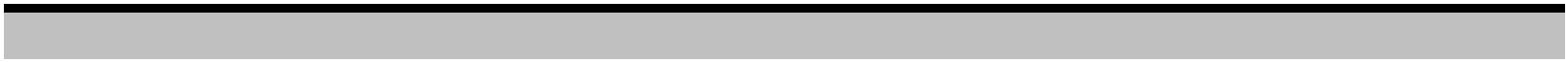


**# Pos Sex Age Tissue Pathological Diagnosis Grade Stage TNM**

59 5K M 53 Lung Adenocarcinoma 3 IIIA T3N1M0

60 5L F 45 Lung Adenocarcinoma 3 IIIB T4N0M0

61 6A F 64 Lung Adenocarcinoma 3 IIIB T4N0M0

62 6B M 56 Lung Adenocarcinoma 3 IIIA T2N2M0

63 6C F 57 Lung Adenocarcinoma with necrosis – IIB T3N0M0

64 6D M 63 Lung Adenocarcinoma 3 IIIA T2N2M0

65 6E M 64 Lung Adenocarcinoma 3 IIB T3N0M0

66 6F F 39 Lung Adenocarcinoma 3 IIIA T2N2M0

67 6G M 43 Lung Adenocarcinoma with necrosis 3 IIIB T4N0M0

68 6H M 66 Lung Adenocarcinoma 3 IIIA T2N2M0

69 6I M 65 Lung Adenocarcinoma 3 IIIA T3N1M0

70 6J F 71 Lung Adenocarcinoma 3 IIIA T2N2M0

71 6K M 62 Lung Adenocarcinoma 3 IB T2N0M0

72 6L M 42 Lung Adenocarcinoma 3 IIIA T3N1M0

73 7A M 38 Lung Adenocarcinoma with necrosis 3 IIIA T3N2M0

74 7B M 65 Lung Adenocarcinoma 3 IIIA T3N1M0

75 7C F 62 Lung Adenocarcinoma 3 IIIA T3N1M0

76 7D F 56 Lung Adenocarcinoma 3 IIIA T3N1M0

77 7E M 43 Lung Adenocarcinoma 3 IIIA T3N2M0

78 7F M 75 Lung Adenocarcinoma 3 IIIA T2N2M0

79 7G M 52 Lung Adenocarcinoma 3 IIIA T4N0M0

80 7H F 34 Lung Adenocarcinoma 3 IV T4N1M1

81 7I M 45 Lung Adenocarcinoma 3 IB T2N0M0

82 7J F 49 Lung Adenocarcinoma 3 IB T2N0M0

83 7K M 71 Lung Adenocarcinoma 2 IB T2N0M0

84 7L M 63 Lung Adenocarcinoma with necrosis 3 IIB T3N0M0

85 8A F 62 Lung Adenocarcinoma 3 IIB T3N0M0

86 8B F 24 Lung Adenocarcinoma 3 IIB T3N0M0

87 8C F 51 Lung Adenocarcinoma 3 IB T2N0M0

88 8D F 45 Lung Adenocarcinoma 3 IB T2N0M0

89 8E F 53 Lung Adenocarcinoma 3 IIB T3N0M0

90 8F F 51 Lung Adenocarcinoma 3 IB T2N0M0

91 8G F 47 Lung Adenocarcinoma 3 IB T2N0M0

92 8H M 53 Lung Adenocarcinoma 3 IIB T2N1M0

93 8I M 79 Lung Adenocarcinoma 3 IIIB T4N1M0

94 8J F 51 Lung Adenocarcinoma 3 IIIB T4N1M0

95 8K M 38 Lung Adenocarcinoma with necrosis 2 IIIB T4N1M0

96 8L M 55 Lung Adenocarcinoma 2 IIIB T4N0M0

97 9A M 44 Lung Adenocarcinoma 2 IIIA T3N1M0

98 9B M 62 Lung Adenocarcinoma 2 IIIA T3N2M0

99 9C M 61 Lung Adenocarcinoma 2 IIIC T4N3M0

100 9D M 64 Lung Adenocarcinoma 3 IIIB T4N0M0

101 9E F 50 Lung Adenocarcinoma 3 IB T2N0M0


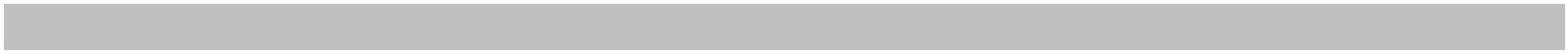

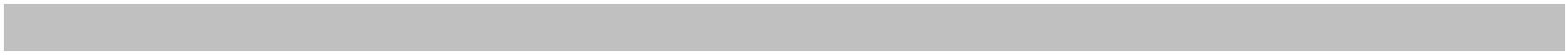

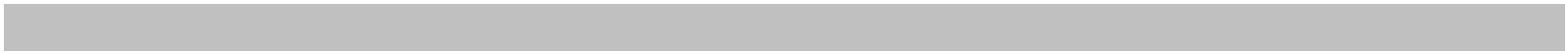

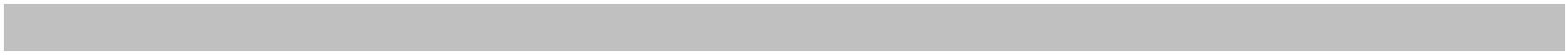

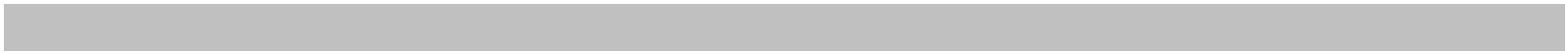

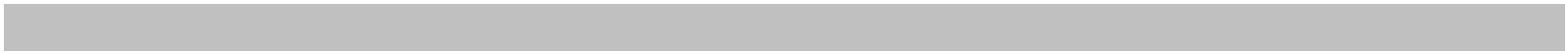

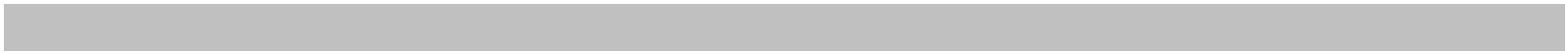

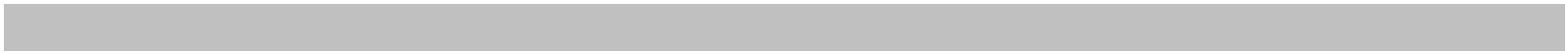

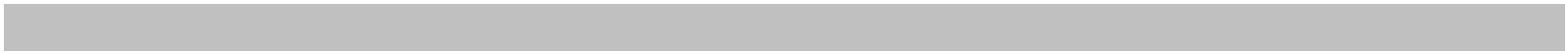

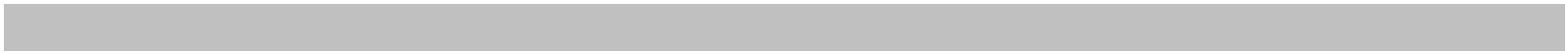

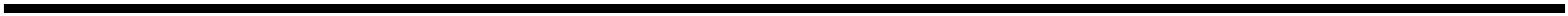


**# Pos Sex Age Tissue Pathological Diagnosis Grade Stage TNM**

102 9F M 39 Lung Adenocarcinoma 2 IIIB T4N0M0

103 9G F 44 Lung Adenocarcinoma 2 IIIA T3N1M0

104 9H F 57 Lung Adenocarcinoma 2 IIIB T4N0M0

105 9I F 45 Lung Adenocarcinoma 2 IIIB T4N1M0

106 9J F 51 Lung Adenocarcinoma 3 IB T2N0M0

107 9K M 52 Lung Papillary adenocarcinoma 2 IIIA T3N1M0

108 9L M 54 Lung Papillary adenocarcinoma 2 IB T2N0M0

109 10A F 43 Lung Papillary adenocarcinoma 2 IIIA T3N1M0

110 10B F 51 Lung Papillary adenocarcinoma 2 IB T2N0M0

111 10C M 52 Lung Cancer adjacent normal lung tissue – – –

112 10D M 57 Lung Cancer adjacent normal lung tissue – – –

113 10E F 54 Lung Cancer adjacent normal lung tissue – – –

114 10F M 65 Lung Cancer adjacent normal lung tissue – – –

115 10G F 57 Lung Cancer adjacent normal lung tissue – – –

116 10H M 46 Lung Cancer adjacent normal lung tissue – – –

117 10I M 30 Lung Normal lung tissue – – –

118 10J F 17 Lung Normal lung tissue – – –

119 10K M 45 Lung Normal lung tissue – – –

120 10L F 18 Lung Normal lung tissue – – –

121 10M - - - Array marker – –
